# Global Gradients of Coral Exposure to Environmental Stresses and Implications for Local Management

**DOI:** 10.1371/journal.pone.0023064

**Published:** 2011-08-10

**Authors:** Joseph Maina, Tim R. McClanahan, Valentijn Venus, Mebrahtu Ateweberhan, Joshua Madin

**Affiliations:** 1 Computational Ecology Group, Department of Biological Sciences, Macquarie University, Sydney, New South Wales, Australia; 2 Marine Programs, Wildlife Conservation Society, Bronx, New York, United States of America; 3 Faculty of Geo-Information Science and Earth Observation, University of Twente, Enschede, The Netherlands; 4 Department of Biological Sciences, University of Warwick, Coventry, United Kingdom; Biodiversity Insitute of Ontario - University of Guelph, Canada

## Abstract

**Background:**

The decline of coral reefs globally underscores the need for a spatial assessment of their exposure to multiple environmental stressors to estimate vulnerability and evaluate potential counter-measures.

**Methodology/Principal Findings:**

This study combined global spatial gradients of coral exposure to radiation stress factors (temperature, UV light and doldrums), stress-reinforcing factors (sedimentation and eutrophication), and stress-reducing factors (temperature variability and tidal amplitude) to produce a global map of coral exposure and identify areas where exposure depends on factors that can be locally managed. A systems analytical approach was used to define interactions between radiation stress variables, stress reinforcing variables and stress reducing variables. Fuzzy logic and spatial ordinations were employed to quantify coral exposure to these stressors. Globally, corals are exposed to radiation and reinforcing stress, albeit with high spatial variability within regions. Based on ordination of exposure grades, regions group into two clusters. The first cluster was composed of severely exposed regions with high radiation and low reducing stress scores (South East Asia, Micronesia, Eastern Pacific and the central Indian Ocean) or alternatively high reinforcing stress scores (the Middle East and the Western Australia). The second cluster was composed of moderately to highly exposed regions with moderate to high scores in both radiation and reducing factors (Caribbean, Great Barrier Reef (GBR), Central Pacific, Polynesia and the western Indian Ocean) where the GBR was strongly associated with reinforcing stress.

**Conclusions/Significance:**

Despite radiation stress being the most dominant stressor, the exposure of coral reefs could be reduced by locally managing chronic human impacts that act to reinforce radiation stress. Future research and management efforts should focus on incorporating the factors that mitigate the effect of coral stressors until long-term carbon reductions are achieved through global negotiations.

## Introduction

Corals globally are exposed to diverse and often interacting physico-chemical and biological disturbances [1], [2]. The diversity, spatio-temporal heterogeneity, and interactions of these disturbances have complicated the understanding of the response of coral assemblages to multiple stressors [1], and reduced the potential for spatially targeted coral reef management strategies. To counteract species extinctions predicted by many [e.g. 3], [4], [5], corals would have to adapt to temperatures of more than 2°C above normal thresholds by the turn of the century [6], [7], in addition to coping with a suite of other stressors [8]. For example, local stressors such as eutrophication from coastal watersheds exacerbate coral stress by changing the oligotrophic conditions where coral reefs function optimally [9], [10], [11], [12], while overfishing and removal of grazers is accelerating a shift towards algal dominance [9], [13], [14].

Given the bleak view of the status and prognosis for coral reefs globally, timely identification of spatial gradients of their exposure to global and local stressors is needed so that appropriate counter-measures can be formulated and implemented. The management strategies proposed include among others: (i) protecting coral reef locations with biological and environmental conditions that render them less exposed or vulnerable to stress [15], [16], [17], [18]; and (ii) reducing anthropogenic disturbances such as overfishing and pollution, which are likely to reduce the resistance and tolerance of corals to radiation (temperature and ultraviolet light) stress [19], [20], [18]. Understanding of where, when and how global and local stressors affect corals can strengthen the decision support needed for appropriate coral reef management [7], [21], [22], [23], [24]. The two important considerations that have arisen from these multidisciplinary studies are: (i) assessment of the degree of exposure to multiple interacting stressors at different scales; and (ii) understanding how the environment interacts with the coral community structure and coral-algal symbiosis in influencing their sensitivity, vulnerability and adaptability to thermal, radiation and other physiological and biomechanical disturbances. The first of these two metrics are evaluated here as one of the important considerations that underpins the concepts of the resilience and vulnerability of coral reefs more generally [25].

Ecosystem vulnerability, although defined in different ways, is most often conceptualized as a function of the exposure, sensitivity and adaptive capacity of the perturbed organisms or ecosystems [26]. Sensitivity is a property of a system that is difficult to estimate and is dependent on the interaction between the biological and ecological characteristics of a system as well as on the attributes of the environmental stimulus [27]. Unlike sensitivity and adaptive capacity, exposure is an attribute of the relationship between the system and perturbations, rather than of the system itself [26]. These three metrics of vulnerability overlap and the environmental and biological processes that drive them are frequently interdependent [27]. For instance, many of the determinants of coral sensitivity (e.g. acclimatization) are similar to those that influence or constrain a system's adaptive capacity (e.g. genetic and species diversity, dispersal, and connectivity).

In this study, we derive a generic exposure metric and translate it into fuzzy logic mathematical expressions. The modelling of coral exposure, like many reef processes, is often hindered by poor knowledge of the physiology of corals complicated by contradicting theories on coral-environment interactions [18], sparse data, and poor precision [28]. Frequently, important observations are lacking and potentially valuable information may be non quantitative [29], which may limit the usefulness of these models. For example, the ability of corals to adapt or acclimatize to abnormal conditions is not well understood [30]. Fuzzy logic, first introduced by Zadeh [31], offers a methodology for dealing with these problems and provides an alternative approach to modelling complex systems. For example, translating data layers to fuzzy measures results in standardised measures of the possibility of belonging to a given set along a continuous scale from 0 to 1 [32]. This approach is more realistic than a binary set membership rule as is used in Boolean analyses, especially when there is uncertainty inherent in the input data [29].

### Stressor interactions, coral response and environmental thresholds

In benthic aquatic habitats, the light and temperature environment is highly dynamic and is primarily a function of hydrodynamics (tidal regime, currents, and stratification), cloud cover, and turbidity among other factors [33], [34], [35]. For instance, extreme tides in turbid waters causes a much greater increase in benthic irradiance than in clear water [34], [36], which has been shown to cause significant coral mortality [34], [37], [38], [39]. Moreover, as wind speed falls, vertical-mixing decreases, resulting in decreased evaporative cooling and transfer of deeper cool water, which increases the likelihood of thermal stress on corals [6], [33], [40]. Based on published hypotheses and conceptual deductions about the likely response of corals to a given stressor (Appendix S1), we use a systems analytical approach to idealize the coral-environment relationships. We considered a series of composite stressors derived from combinations of sea surface temperature (SST), UV irradiance, wind speed, tidal range, and chlorophyll a concentration data. SST, UV, wind magnitude and consistency (together referred here as radiation) are considered to be the primary climatic drivers of coral reef exposure. Tides and SST variability are considered to be stress antagonistic or reducing variables that mitigate the primary climatic stressors. Sedimentation and eutrophication are stress reinforcing or exacerbating interactive stressors because they can undermine the resilience of the coral reef ecosystem through either undermining physiological homeostasis or the recovery processes after disturbance [12], [41]. Coral exposure is a function of derived stressors that interact with radiation having either reinforcing (additive or multiplicative) or reducing affects (antagonistic) [2], [42], [43]. It is this combination of reinforcing and reducing effects that causes the complex and sometimes surprising behavior of composite coral-environmental systems that is not well predicted by simple models that consider one or few coral-environmental variables [44].

**Figure 1:**
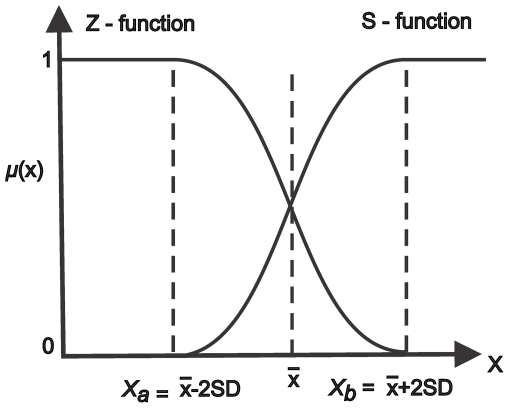
Increasing (S curve) and decreasing (Z curve) sigmoidal membership functions, which were used to standardize the environmental data. *x*_a_ and *x*_b_ are the control points for the lower and upper bounds along the stressor gradient; SD is the standard deviation, while 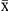 is the mean.

Most methods for estimating thresholds of environmental attributes, such as thermal and sediment levels, above which stress responses such as coral bleaching, diseases and mortality are likely to occur [6], [45], [46] mostly rely on availability of response observations (e.g., [47]). There are limited insights for identifying when thresholds may be crossed, in a setting with interactive, and cumulative impacts of multiple stressors, which often result in spurious and confounding effects [2], [24]. In addition, a system's response to stressors can adopt various linear and non-linear complex behaviour patterns, which for modelling purposes can be represented in many forms of fuzzy logic membership functions including trapezoidal, sinusoidal, logistic, Gaussian etc [2], [48]. In this study, we estimate environmental limits of corals (*x*_a_ and *x*_b_) based on the distribution of global environmental data for locations where corals are found. We assume that geophysical variables in coral reef areas are distributed normally, where *x*_a_ and *x_b_* are two standard deviations from the mean on the lower and upper tail. For simplicity, we assign a normal cumulative function (represented as logistic curve in fuzzy logic membership function) as the response of the interaction between coral and environment, where coral exposure is a function of the environmental variables considered, and initially increases or decreases exponentially along the environmental gradient respectively above or below the user defined minimum threshold (*x*_a_), before levelling off at a user defined maximum threshold (*x_b_*) [2] (Fig. 1).

Because coral bleaching and mortality is driven by factors such as temperature and their interactions with other stressors like pollution and sedimentation, it may be possible to prevent some damage by reducing the impact of stressors that are not related to climate change [18], [19]. Additionally, fishing can influence grazers and algae and subsequently influence the overall recovery rates and resilience to climate disturbances [9]. This study aims to identify the global spatial gradients of thermal and eutrophication stressors and of the key factors that reduce these stressors to develop a broad-scale metric of environmental exposure for coral reefs. In addition, we address the questions: (i) which of these stressors are corals most exposed to in their respective locations, (ii) which reef locations are least and most exposed to thermal and UV radiation and sedimentation stress, and (iii) how do these stress and reinforcing and reducing variables interact globally?

## Materials and Methods

We used environmental data from satellite observations and model outputs to derive variables that represent temperature and UV light (radiation), reinforcing and reducing stress.

### Sea surface temperature

Sea surface temperature derived variables were obtained from the second version of the coral reef temperature anomaly database (CoRTAD) [23]. This database contains global SST and related thermal stress metrics at an approximately 4-km resolution weekly from 1982 through 2008, derived from measurements from the Advanced Very High Resolution Radiometer onboard NOAA suite of polar orbiting satellites. The global accuracy of the retrieval algorithm based on comparisons with in situ buoys indicates values of 0.02–0.5°C [49]. When compared with in situ temperature from data loggers at shallow depth in the western Indian Ocean, RMSE of 0.87°C were reported [16]. The CoRTAD reanalysis database has also been evaluated using *in situ* observations from different coral reef locations globally and at depths ranging from 0–9 m, which corresponds to depths of most coral reef habitats [23]. This evaluation reported RMSEs ranging from 0.49–0.81°C, and a coefficient of determination (R^2^) of 0.72–0.96 [23]. Overall, the performance of this data for global coastal applications is adequate, notwithstanding the fact that radiometers measure the temperature at the sea surface while most *in situ* measurements are based on bulk temperature at shallow depths.

We downloaded time series of weekly SST anomalies (WSSTAs), defined as the weekly averaged temperature in excess of 1°C or more above that week's long term average value; and thermal stress anomalies (TSAs), defined as the temperature excess of 1°C or more above the climatologically (long-term average) warmest week of the year (the warmest week of the 52 climatologically weeks averaged over 27 years) [23], from the National Oceanographic Data Center website (http://www.nodc.noaa.gov/sog/Cortad). Two different cumulative estimates of thermal stress were computed from each of these metrics: TSAs and WSSTAs were summed for each year and averaged over 27 years; and for each year, a maximum duration (in weeks) that WSSTA and TSA were greater than or equal to 1°C were computed and averaged over 27 years. These two metrics, the mean annual cumulative and mean yearly maximum duration, represent the characteristic magnitude and duration of the anomalies at a given location, which are important predictors of coral stress [23], [45]. Mean SST and the coefficient of variation for the 27-year monthly mean time series were also computed.

### Chlorophyll and suspended solids

Oceanic satellite observations in the visible and near-infrared bands allow for the measurement of a variety of ocean color information including phytoplankton chlorophyll-*a*, total suspended matter (TSM), and colored dissolved organic matter (CDOM) [50], [51]. For modeling purposes, ocean waters are commonly described as being of Case I or case II types [52], [53]. The former type are those waters whose optical properties are determined primarily by phytoplankton and related colored dissolved organic matter (CDOM) and detritus degradation products; while the later represents the turbid coastal zones influenced by land drainage or sediment re-suspension, with optical properties mainly influenced by CDOM of terrestrial origin, mineral particles, various suspended sediments, urban discharges and industrial wastes [52].

The application of ocean color data in coral reef areas is limited by the complexity of the water's optical properties in shallow coastal environments where they are found. The standard Case I algorithm for deriving chlorophyll concentration fail in turbid coastal waters resulting in over estimation of chlorophyll along most coastal areas [53], even if due to terrestrial influence considerable enhancements of the algal biomass in these shallow zones is expected. Further, the standard algorithms for both water types were developed on the assumption of optically deep waters. Therefore in clear shallow bottoms that are highly complex or reflective as with the case in coral reefs and atolls, bottom reflection can induce an increase in marine reflectance, which is wrongly interpreted as ocean color constituents [54]. Given these problems, until special algorithms that take into account the complexity in coral reef areas are developed and incorporated in the standard processing chains of the current ocean color satellites, the usefulness of ocean color data for coral reef applications will remain limited [54], [55].

To derive chlorophyll estimates taking into account these problems we carried out a series of analyses with ocean color observations from the Sea-viewing Wide Field-of-view Sensor (SeaWiFS), Moderate Resolution Imaging Spectro-radiometer (MODIS), and Medium Resolution Imaging Spectrometer Instrument (MERIS) sensors (Appendix S2). The GlobColour processor at the European Space Agency's GlobColour project (http://hermes.acri.fr/GlobColour) was used to process Level 2 data from the three sensors to derive monthly level-3 binned products, including case I and case II chlorophyll concentrations with their respective flags, at a resolution 4.63 km at the equator (http://www.GlobColour.info/products_description.html). Data from all the three sensors were merged to derive case I Chlorophyll, while MERIS Case II algorithm was used to retrieve case II chlorophyll [56]. These Level 3 outputs do not spatially differentiate the regions where each of the water types are relevant; therefore further analysis using turbidity flags is required to discern and merge regions with the different water types into a homogenous continuous layer [53]. To achieve this, we used turbidity and depth flags (<30 m) derived from the processing of level 2 products, in a logical expression designed to merge respective case I and case II regions in a given month, and further to exclude shallow water (<30 m) pixels. Having masked shallower depths using the depth flags, we assumed similar water column properties in masked areas to those found in adjacent deeper (>30 m) water pixels, and extrapolated the deeper water pixels to these areas. To achieve this for each layer, we applied 3×3 spatial interpolator, which calculates the median value of 8 pixels adjacent to the pixel being considered. In effect, pixels adjacent to the missing value maintained their original values while the missing pixel was assigned the resulting value from the interpolator [16]. These monthly mean layers were then temporally aggregated for the long-term average.

### Doldrums

Global sea surface wind speed (m s^−1^) estimates for 10 m above sea level at a 28-km resolution are available from the National Climatic Data Center (NCDC, ftp://eclipse.ncdc.noaa.gov/raid1b/seawinds). NCDC wind data is based on the blended observations from multiple sensors, with reduced spatial and temporal gaps of individual satellite samplings, and reduced sub-sampling aliases and random errors [57]. Despite the coastal application of this data by the Coral Reef Watch, inter-comparisons with other products have not been performed because sparse in-situ measurements over the vast ocean surface make errors difficult to quantify [57]. Nonetheless, measurements from each sensor are passed through quality control prior to blending and gridding. Additionally, the blending of cross-calibrated multiple satellite observations is known to increase accuracy and resolution [57], [58].

Daily averaged wind speeds (2000–2009) and the averaged 10-year mean monthly wind speeds (1995–2004) were downloaded. The National Oceanic and Atmospheric Administration (NOAA) coral reef watch defines doldrums as wind conditions with a daily mean of less than 3 m s^−1^. To estimate the magnitude and consistency of wind regimes in a given location, a doldrums metric was computed by taking the annual average maximum number of days that wind speeds were greater than 3 m s^−1^ over 10 years (2000–2009) and multiplying this by the 10-year mean monthly average.

### Tidal model

Over the last decade, the tidal research group of Le Provost and collaborators have produced a series of finite element solution (FES) tidal atlases; FES-2004 is the latest release. Data are computed from the tidal hydrodynamic equations and tide gauges and altimeter data assimilation [59]. When cross-validated with other tidal products, the FES-2004 atlas was found to be the most accurate, with improved performance in shelf and coastal areas and moderately deeper areas [59], [60]. The accuracy of the 15 tidal components used in the model ranges from 2–12 cm and varies by region [60]. Therefore, local applications would require calibration with tidal observations at the same scale.

The digital FES-2004 tidal model and the associated extraction software were downloaded from the Laboratoire d'Etudes en Géophysique et Océanographie Spatiales website (http://www.legos.obs-mip.fr/en/soa) [59], [60]. The software in C++ was modified to enable gridding of the tidal predictions for a user defined spatial and temporal extents. To minimize the computer processing time, the model's temporal resolution was degraded from hourly to 6-hr interval. These predictions were then aggregated for average, minimum, and maximum heights over seven day intervals and gridded at the model's spatial resolution of roughly 14-km. To capture the long-term conditions and variability, the model was run for 8 years from 1987 with a three-year interval, including 1987, 1990, 1993, 1996, 1999, 2002, 2005, and 2008. Tidal ranges were computed as the long term averaged difference between the weekly maximum and minimum simulated tidal heights.

### Ultraviolet radiation

Daily global maps of UV-erythemal (biologically damaging) irradiance at the Earth's surface (for the spectral range 290 to 400 nm and in the units of milli-watts m-^2^) in a 1 by 1.25 degree grid were retrieved for 1996 to 2001 from the NASA website (http://toms.gsfc.nasa.gov) [61], [62]. This data is derived from the total ozone mapping spectrometer (TOMS) on-board Earth Probe-TOMS satellite. Erythemal radiation is a weighted average of UVA (315–400 nm) and UVB (280 to 315 nm) used as a measure of skin irritation caused by exposure sunlight [63]. Errors associated with this data have not been ascertained for many parts of the world, however evaluations in Canada using a ground-based spectrometer reported absolute accuracy of 6% under normal conditions and 12% under conditions of UV absorbing aerosol plumes [61]. These uncertainties are mostly influenced by the amount of ozone, clouds and aerosols, and terrain height. In the ocean, depth attenuation and the optical properties of the seawater influence the amount of radiation below water surface [61], [64]. Radiative transfer modeling that includes the ocean system has been performed to estimate in-water radiation field [60], [65]. Here we use Erythermal UV with no correction for the seawater optical properties. Previous reports have shown a good correlation of this data with coral bleaching where observations were made at varying depth [16].

The current online values of UV irradiance and Erythemal exposure from EP-TOMS have errors after 2001, and therefore can not be used for UV changes as these are more prone to time-dependent errors from cloud cover and aerosols. The application of this data here is limited to global mean, where the overall error is expected to be relatively small, as the mainly negative cloud-height errors and other positive errors usually partly cancel, leading to an overall smaller error [66]. Consequently, UV average from 1997 to the end of 2001 was computed to represent local conditions in each grid square.

### Coral exposure

Environmental variables were grouped into three categories based on the role that they play as coral stressors: (1) radiation variables, consisting of variables derived from temperature (mean SST, TSA and WSSTA magnitude and duration), UV-erythermal and wind speed data (doldrums index); (2) stress reinforcing variable (TSM and chlorophyll-*a*), representing sedimentation and eutrophication; and (3) stress reducing variables, consisting of SST variability and tidal range. Values of each variable that correspond with the approximately 4000 reef locations were extracted, and examined for normality and log10-transformations applied where necessary (Appendix S3). For each variable, a membership function with similar behavior pattern to a normal cumulative distribution function was used to characterize the relationship between coral exposure and a stress variable. Membership functions capture the degree to which the variable x is a member of a fuzzy set *A* using a suitably chosen function *μ*(x) [48]. Here we used spline-based logistic functions:
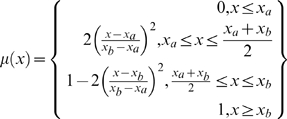
(1)

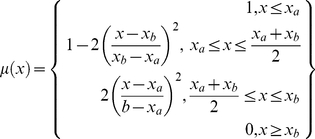
(2)where *x_a_* and *x_b_* are control values and correspond to the lower and upper bound of a stressor values, respectively (Table 1). These were calculated for each variable as the mean value of minus or plus two standard deviations, respectively. Radiation and reinforcing variables were normalized using an increasing curve (Eq. 1) and stress reducing variables were normalized using a decreasing curve (Eq. 2) (Fig. 1).

**Table 1:** Summary statistics for the variables used in the analyses based on (a) coral reef location points, and (b) all pixels within the image spatial boundaries (35N&S, 180E&W); (c) is control values *x_a_* & *x_b_* used in standardizing the variable layers.

		Radiation							Reducing		Reinforcing	
		Mean SST	Mean sum SSTA	Mean sum TSA	Mean SSTA duration	Mean TSA duration	UV	Doldrum index	Tidal range	SST coeff. of var.	Chlorophyll	TSM
**(a)**	**Coral reef areas**											
	N	3822	3901	3901	3901	3901	3958	3963	3914	3822	3274	3325
	Average	26.9	16.0	3.9	2.0	0.8	250.0	20.1	0.7	5.6	0.7	0.8
	Std dev	1.3	1.2	1.4	1.4	1.6	22.9	1.5	2.3	1.6	1.2	1.5
	Min	20.8	11.0	1.0	0.7	0.1	136.6	1.0	0.1	1.5	0.0	0.1
	Max	29.6	52.0	24.3	10.6	5.7	322.4	111.5	3.3	21.9	13.8	44.9
**(b)**	**Global values**											
	Average	22.1	21.0	13.9	5.7	4.0	244.3	21.5	0.7	20.9	0.2	0.8
	Std dev	4.4	1.5	2.6	2.6	2.6	45.6	1.7	2.2	2.2	0.6	0.8
	Min	14.5	0.9	0.0	0.0	0.0	124.5	0.0	0.0	1.3	0.0	0.1
	Max	29.7	90.3	84.7	16.5	12.9	419.6	134.8	4.9	51.8	27.2	45.2
**(c)**	**Control values**											
	Xa	24.3	10.8	1.9	1.0	0.3	204.2	9.0	0.1	2.2	0.1	0.2
	Xb	29.6	23.7	8.2	4.1	2.1	295.8	45.1	3.6	14.4	2.4	2.0

Mean SST and UV were not log transformed.

Spatial Principal Component Analyses (SPCA) was used to combine the standardized variables within each category. Principal Component Analysis transforms each variable into a linear combination of orthogonal common components (output layers), or latent variables with decreasing variation. The linear transformation assumes the components will explain all of the variance in each variable. Hence, for each output the latent component layer carries different information, which is uncorrelated with other components. This enables a reduction of output maps because the last transformed map(s) may be discarded as they have little or no variation left and may be virtually constant. The component weightings were calculated using coefficients of linear correlation to weigh the contribution of factors in spatial principal component analysis [67]. SPCA was performed to synthesize the standardized variables within radiation, stress reducing, and stress reinforcing categories. A final composite map from each of these three groups was computed by summing PC's with contribution ratio >1, weighted by their respective contribution ratio (Equation 3; [68], [16]).
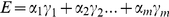
(3)where *Y_i_* is the *i^th^* principal component, while *α_i_* is its corresponding contribution ratio.

The output maps were standardized between zero and one, representing low and high exposure respectively. To combine the stress reducing and radiation variables, SPCA procedure described above was repeated with standardized radiation and reducing variables as the input variables. The output PC's were synthesized using a weighted sum equation (Eq. 3) to yield a layer with estimates of exposure to radiation taking into account the contribution from reducing variables. Fuzzy-integration-based approach was used to integrate the output from this procedure with the reinforcing variables into a single composite layer. [69] lists five fuzzy operators that are most useful for combining fuzzy data (AND, OR, sum, product and gamma). Given two fuzzy sets (standardized layers) *A* and *B*, the fuzzy sum operator produces a layer whose values are equal to or greater than each of the input layers *A* and *B* and results in an increased effect [69]. We therefore used fuzzy sum operator to reflect the reinforcing behaviour of sediment and eutrophication to radiation stress:
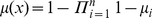
(4)where 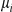 .is the membership value for *i*-th map, and *i* = *A, B*, *n* maps.

**Figure 2:**
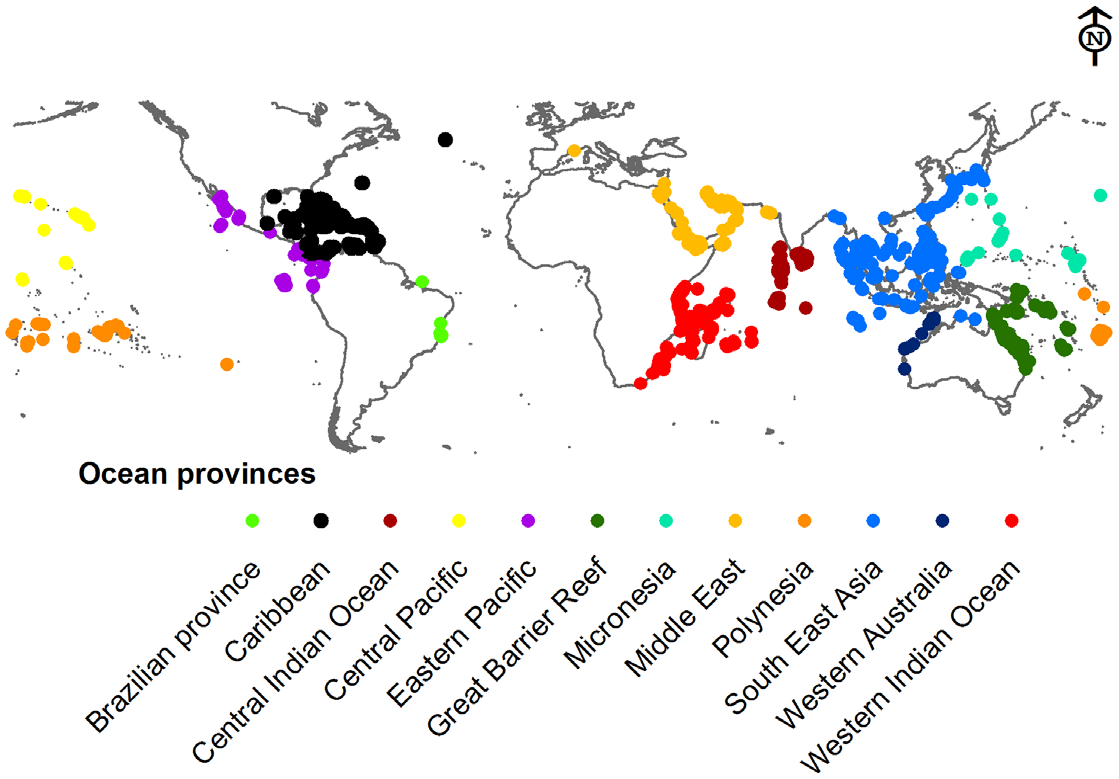
Coral reef locations grouped into eleven oceanic provinces after Donner (2009). Coral reef locations were obtained from Reefbase (http://reefgis.reefbase.org/), WCS coral monitoring sites in the western Indian Ocean, and from Ateweberhan & McClanahan (2010).

Coral reef location data was obtained from the Reef Base website (http://reefgis.reefbase.org/) and the Wildlife Conservation Society monitoring sites in the western Indian Ocean [70]. The location data were grouped into eleven oceanic provinces [9] (Fig. 2). For the respective locations, exposure metrics as described above were extracted for the corresponding locations. Box plots of exposure metrics by stressors against the coral reef provinces were plotted.

### Exposure gradation

Exposure gradation, also termed “defuzzification,” is a process where fuzzy application outputs are converted into a crisp output to facilitate their interpretation [48]. We used an iso-cluster (clustering) approach to partition exposure membership grades map into 4 user-defined clusters of statistically homogenous classes (i.e. low, moderate, high and severe).

Data for the three stress categories and for the final model were extracted for the sample reef locations. Correspondence analyses [71] were performed to detect the structural relationships among the oceanic provinces based on the three stress groups and on the exposure classes. The results of correspondence analysis were presented on a bi-plot that represents the configurations of points in projection planes formed by the first two principal axes [71]. To determine the distribution of sampled locations by region on the basis of their respective partial exposure scores, exposure space bi-plots of reinforcing against radiation and reducing stresses were generated. Contours were also drawn on these exposure space bi-plots based on the break points of final model exposure classes.

## Results

### Global patterns

**Figure 3:**
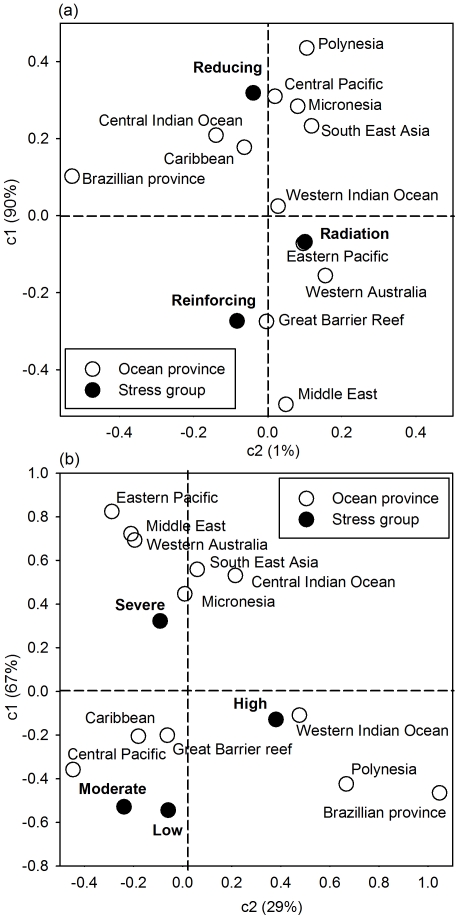
Correspondence bi-plots of the oceanic provinces based on the three stress groups (radiation, reducing, and reinforcing) and based on exposure severity class (a & b respectively.

**Figure 4:**
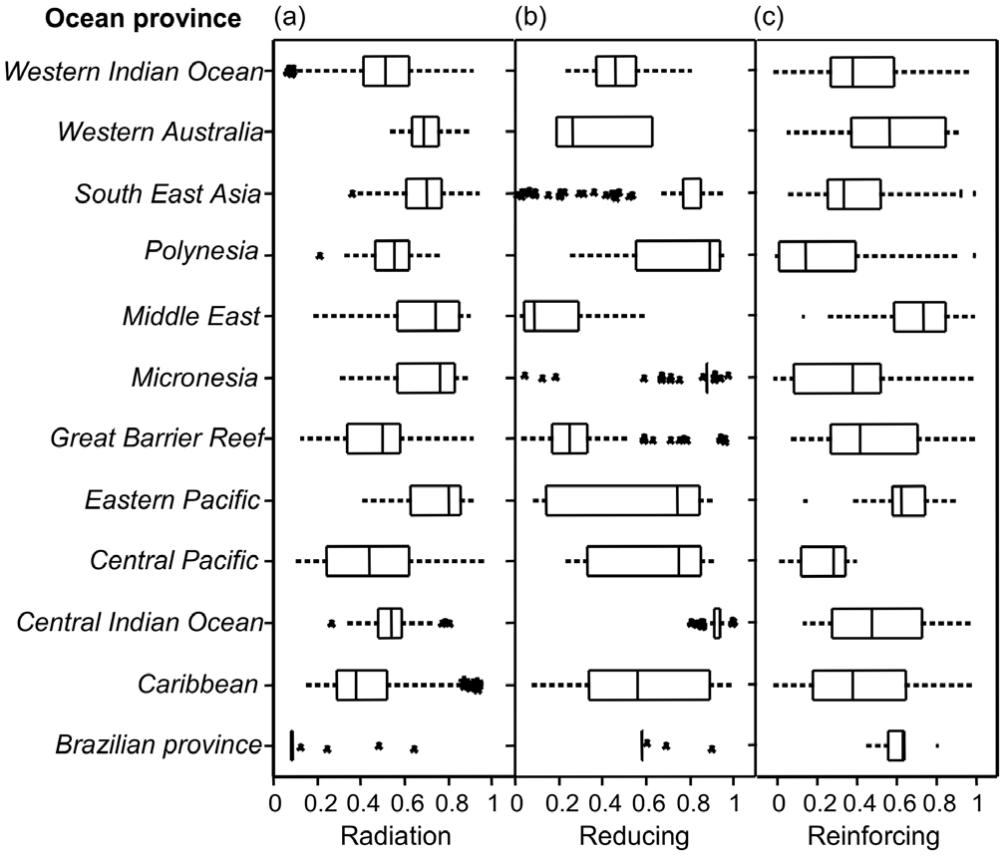
Box plots of distribution of, radiation, reducing, and reinforcing stress (a, b, & c respectively).

**Figure 5:**
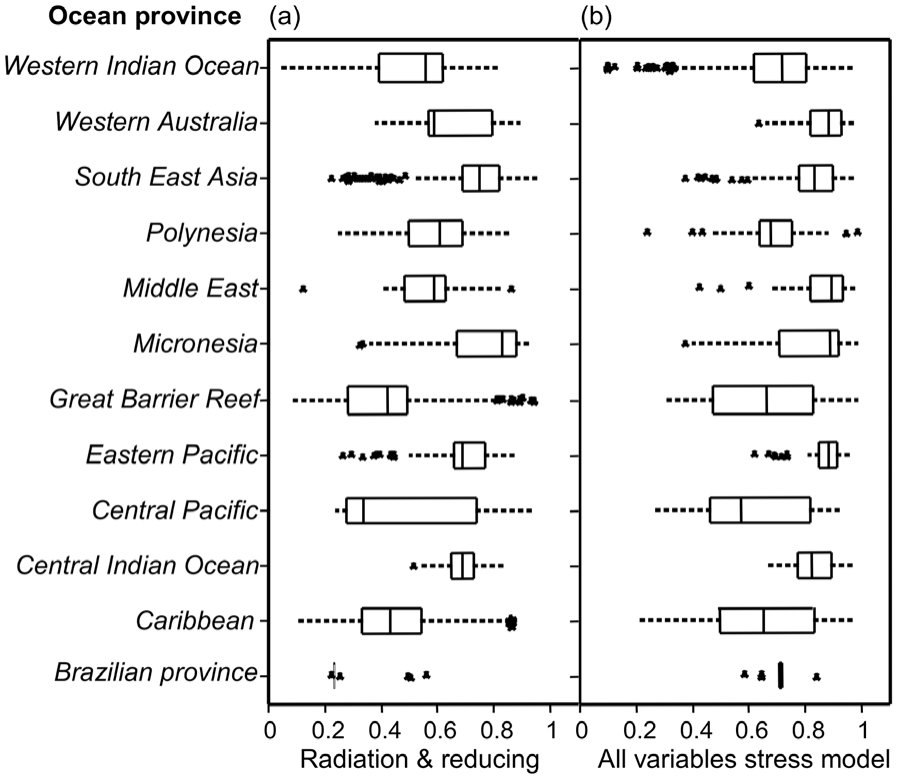
Box plots of distribution of combined radiation stress and stress reducing variables, and the overall exposure model (a & b respectively).

Analyses of the partial and overall exposure from the three stress groups indicate that corals at locations in all the 12 oceanic provinces were evaluated as highly exposed to radiation and reinforcing stress, albeit with spatial variability within the regions (Figs. 3, 4, 5). Ordination of the oceanic provinces by their respective exposure scores in each of the three stress groups in a correspondence analyses showed that 90% of the variation was captured by the first principal axis (c1) (Fig. 3a). The marginal variances explained by the stress categories and their relative position on the correspondence bi-plot indicates that reinforcing variables were most influential (negative in c1), and in descending order radiation and reducing (lack of); radiation stress was neutral among all regions; and the reducing stress had the lowest influence on the first axis (Fig. 3a).

**Figure 6:**
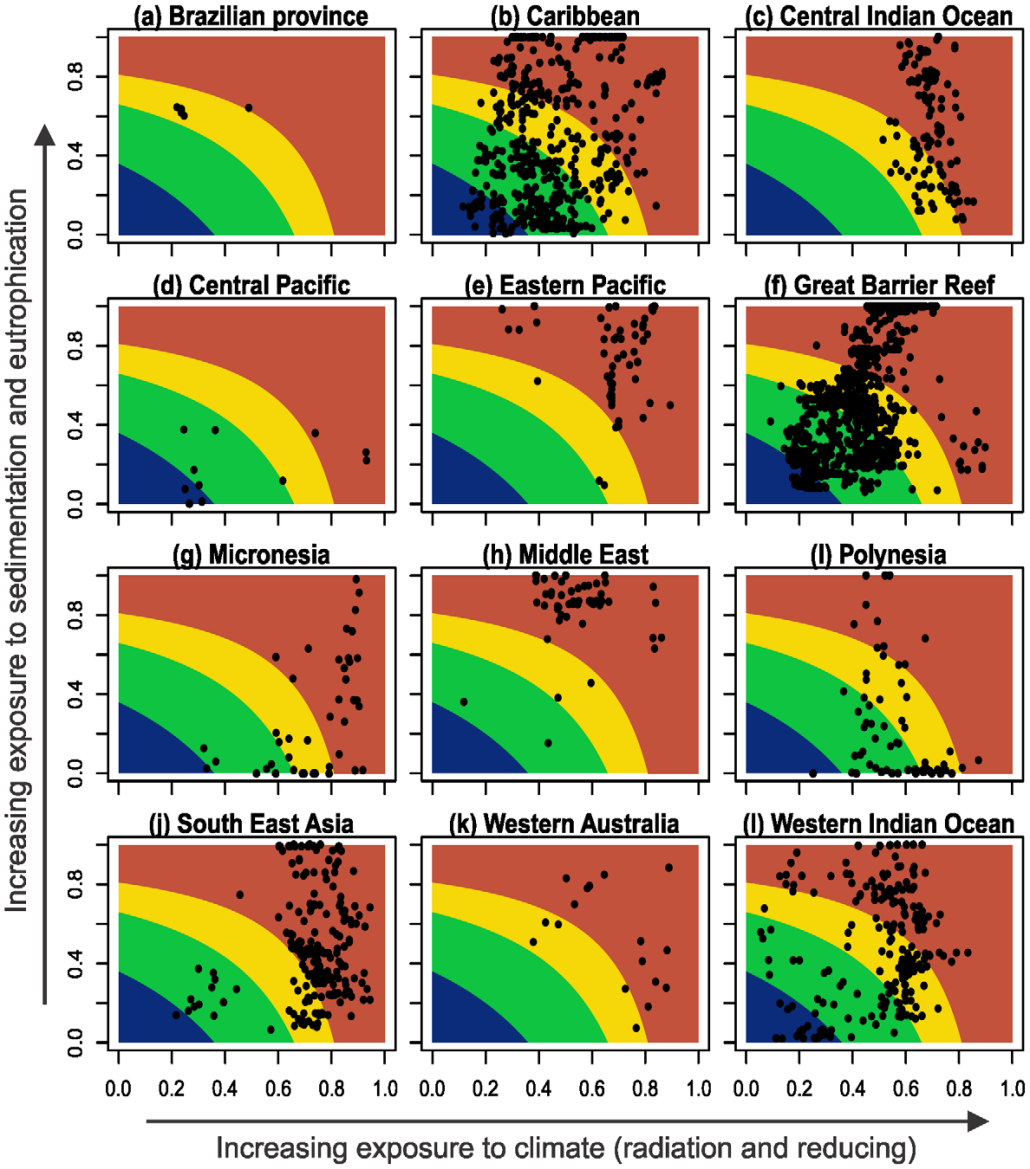
Exposure space bi-plots of reinforcing against combined radiation and reducing variables, with contours showing exposure grades (i.e. low, moderate, high, severe) based on the final exposure model.

When the regions were grouped based on their assigned exposure grades (Fig. 3b), a pattern emerged where regions clustered around two exposure extremes as follows: South East Asia, Eastern Pacific, Micronesia, and the central Indian ocean grouped on the severe exposure extreme, primarily due to low reducing (high reducing scores) and high radiation stress scores (Fig. 3a, b), the Middle East and Western Australia were also in this group primarily due to high scores from reinforcing stress (Fig. 3a). The second cluster of regions strongly associated with moderate-high exposure included the Caribbean, Great Barrier Reef (GBR), Central Pacific, Polynesia and the western Indian Ocean, all with moderate-high scores from radiation and GBR strongly associated with reinforcing stress; while the Brazilian province with low exposure did not conform well to any of these groups (Table 2, Fig. 3a, b). Partial exposure scores from the three stress groups indicate that the Caribbean, GBR, South East Asia, and the western Indian Ocean were highly variable as depicted by the outliers in the lower and higher extremes of the whiskers (Fig. 4) and by the distribution of sample points in the exposure space bi-plots (Fig. 6b, f, j, l).

**Table 2:** Regional statistics for all three-stress groups, for radiation and reducing composite, and for the stress model.

Ocean province		Radiation		Reducing		Reinforcing		Radiation & Reducing		Stress model	
	N	Mean	SE	Mean	SE	Mean	SE	Mean	SE	Mean	SE
Western Indian Ocean	407	0.5	0.0	0.5	0.0	0.4	0.0	0.5	0.0	0.7	0.0
Western Australia	26	0.7	0.0	0.4	0.0	0.6	0.1	0.7	0.0	0.9	0.0
South East Asia	412	0.7	0.0	0.8	0.0	0.4	0.0	0.7	0.0	0.8	0.0
Polynesia	123	0.5	0.0	0.8	0.0	0.3	0.0	0.6	0.0	0.7	0.0
Middle East	96	0.7	0.0	0.2	0.0	0.7	0.0	0.6	0.0	0.9	0.0
Micronesia	62	0.7	0.0	0.8	0.0	0.4	0.0	0.8	0.0	0.8	0.0
Great Barrier Reef	1530	0.5	0.0	0.3	0.0	0.5	0.0	0.4	0.0	0.7	0.0
Eastern Pacific	103	0.7	0.0	0.5	0.0	0.7	0.0	0.7	0.0	0.9	0.0
Central Pacific	17	0.5	0.1	0.7	0.1	0.3	0.0	0.5	0.1	0.6	0.1
Central Indian Ocean	169	0.5	0.0	0.9	0.0	0.5	0.0	0.7	0.0	0.8	0.0
Caribbean	1035	0.4	0.0	0.6	0.0	0.4	0.0	0.4	0.0	0.7	0.0
Brazilian province	18	0.1	0.0	0.6	0.0	0.6	0.0	0.3	0.0	0.7	0.0

The GBR, Middle East and Western Australia were, in relative terms, exposed to high stress reducing effect (thus low exposure scores) from tidal movement and high temperature variability, while the central Indian Ocean, Central and Eastern Pacific, Polynesia, and South East Asia were relatively exposed to low reducing effect as shown by the high partial exposure scores attributed to low stress reducing conditions (Fig. 4b). Western Indian Ocean and the Caribbean reefs were moderately exposed to reducing conditions with the later province being highly variable (Table 2, Fig. 3a; Fig. 4). In the Middle East, high reinforcing stress was mainly in the Persian Gulf (Bahrain and Iran) and the Gulf of Oman, Southern Red Sea and Gulf of Aden, and Southern Asia on the Gulf of Kutch on the Northerly Gujarat coast among other locations.

### Regional patterns

**Figure 7:**
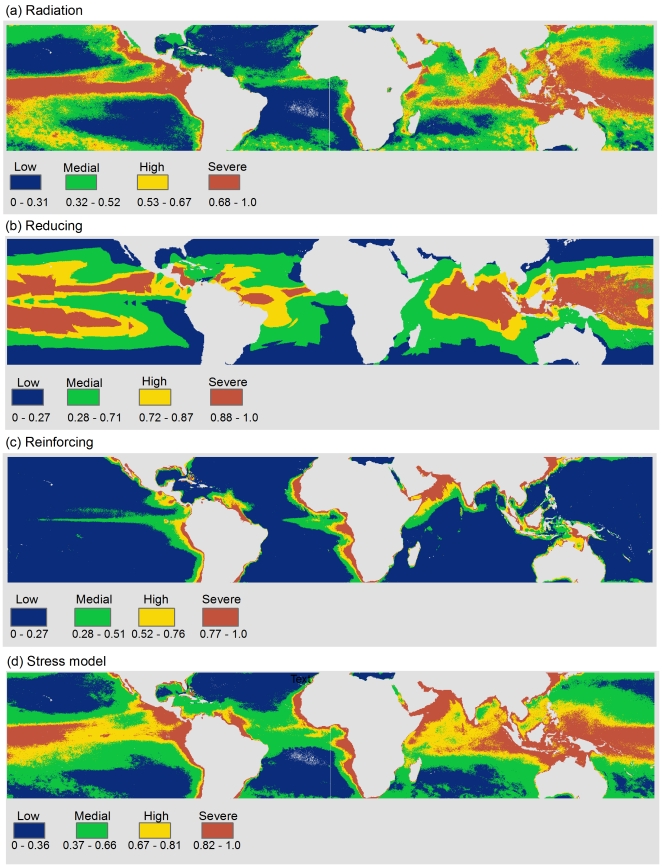
Composite layers for radiation, reducing, reinforcing stress categories, and the overall stress model (a, b, c & d respectively).

The Central Pacific, Micronesia and Polynesia oceanic provinces were weakly exposed to reinforcing variables, and the overall exposure was largely due to high exposure to radiation stress (top and bottom right distribution of sample points in the exposure space bi-plots of Fig. 6 d,g,i). These regions were also exposed to relatively low stress-reducing effects alongside the central Indian Ocean and a more variable eastern pacific (Figs. 4, 5, 6). In South East Asia, all locations with low to moderate overall exposure grades (on the bottom left of the exposure space bi-plots) were in the Far East in the coral reefs of Japan, while the rest of the region was mostly high to severely exposed primarily from radiation stress and a low stress reducing effect (Fig. 6-j). The reinforcing effect was generally low to moderate, but some locations were highly exposed to reinforcing effect including Kagoshima Bay and Western Shikoku in Japan, Polillo Islands and Bolinao in the Philippines, Pari Island, East Kalimantan, and Tanjong Berakit in Indonesia, and several locations in Thailand, Cambodia and Malaysia (Fig. 7, See Appendix S4). Reefs in South East Asia, including the Islands of Peghu, and Peru in Taiwan and Indonesia respectively, Honcau and Holong Bay in Vietnam, and Shikoku in Japan are overall severely exposed, although they have low to moderate exposure to radiation they have high to severe exposure to reinforcing stress (Table 2, Fig. 7, and See Appendix S4).

Western Australia is exposed to severe conditions due to reinforcing stress. Despite the high exposure to doldrums, the tidal variability was high and likely to mitigate radiation stress, for example in Tantabiddi, mangrove islands, and Onslow reef, all had low to moderate radiation stress. Ningaloo and Abrolhos Islands have low radiation but were severely exposed to reinforcing stress alongside Dampier Archipelago, and in the South of Conzine and Withnell Bays. The reefs in the offshore islands off NW Australia in Seringapatam Reef, Hibernia, Timor Sea Reefs, Scott Reef, and Rowley Shoals were not exposed to high reinforcing stress, but were highly exposed to radiation stress due to high doldrums and low tide variability.

The Central Indian Ocean had many reefs with high exposure primarily due to radiation stress (Fig. 6c). These reefs are generally exposed to relatively low reinforcing stress, with the exception of reefs in Sri Lanka and India (Fig. 6c, Fig. 7). The GBR was moderately exposed to radiation and highly exposed to reinforcing stress, and to relatively high stress reducing effects of winds and tides. However, reefs in Kimbe Bay in Papua New Guinea and Solomon Islands in South-west Pacific were highly exposed to radiation stress.

The western Indian Ocean reefs were mostly ranked high to severe in overall exposure, except for some reefs mainly in South Africa, Mauritius, Reunion, Rodrigues, and Torres Reef in Mozambique, which were least exposed (Fig. 6-l, see Appendix S4). Although highly variable, the main stress contributor in this region was radiation and reinforcing stress. In most coral reef locations where exposure grade was severe, radiation and reinforcing stress were both high (Fig. 6-l). These reefs included Malindi and Kiunga in Kenya, reefs in southern Tanzania, most of western Madagascar, and Berreira and Vamizi reefs in Mozambique among others. Reefs at Grand River South East in Mauritius, Lagoa Pinnacle and Coral Gardens in Mozambique had low exposure to radiation stress but were severely exposed to reinforcing stress (Fig. 6-l, See Appendix S4).

In the Eastern Pacific, reef locations in the south were mostly severely exposed to radiation stress and included Inguana, Saboga, Uraba, Taboga, Contadora Islands, and the Gulf of Chiriqui in Panama, several reefs in Gorgona Island in Colombia, Culera Bay in Costa Rica, and the Galapagos Archipelago in Ecuador (Fig. 6-e, 7, and See Appendix S4). The northern part of the Eastern Pacific, including along the Gulf of California, had high reducing and reinforcing effects.

Overall the Caribbean region was moderate to highly exposed but also with high spatial heterogeneity in the exposure variables (Figs. 4, 5, 6, 7). The overall exposure of Caribbean reefs to radiation stress was moderate but several locations were outliers and highly exposed to radiation stress. Reef locations in high and severe exposure grades, with reinforcing as the major stress contributors, included reef locations in eastern Panama, Belize, the Bahamas, Cuba, eastern Mexico, and the Florida Keys (Fig. 7; See Appendix S4).

## Discussion

Coral reefs globally are highly dependent on radiation, but are also exposed to radiation stress when values exceed normal seasonal and inter-annual ranges [72]. Stress, as used here, is the environmental exposure and does not distinguish the physiological acclimatization or genetic adaptation that determines the corals and other organisms' sensitivity to these forces. The degree of sensitivity will determine how organisms counter these stresses and therefore our metric is only a comparative baseline of the forces that are exogenous to the reef organisms. This exposure measure alone will not have predictive power in determining responses to the environment, which requires the sensitivity and adaptive capacity of the organisms, but does provide a basis for understanding the forces that these organisms face.

The results suggest considerable spatial heterogeneity globally but also some clear groupings based on our metrics of radiation stress and reinforcing and reducing variables. The spatial heterogeneity of coral stressors and their influence on coral physiology provide a basis to tailor management strategies that can address locally relevant threats [20], [73]. Determining the specific spatial locations with lower or higher cumulative stress and with significant non-climate change related stressors can assist this prioritization process. Despite the difficulties of discriminating among stressors [74], the results of this study demonstrate the utility of disaggregating stress into various components to emphasize management strategies and to effectively reduce the degradation of coral reefs [75]. The implications of this variability are discussed below in terms of the classification of reefs based on these variables and potential management recommendations.

There is increasing concern globally that enhanced runoff from human land uses is leading to the degradation of coral reefs [41]. It has been argued from studies on the inshore reefs of GBR that poor water quality lowers the radiation tolerance of scleractinian corals [12]. It has also been shown that the bioerosion, growth, and recovery rates of coral reefs are often slowed by high nutrient concentrations [41], [76]. Low water quality can reduce the stress of light and its interaction with temperature to increase bleaching response [72], [77]. However, corals stressed by sedimentation and eutrophication may have a lower capacity to tolerate the effects of other stressors and recover slower, making these factors as overall reinforcing variables [41], [78]. Consequently, if these studies are relevant globally, sedimentation and eutrophication reinforce coral reef stress and improved water quality will increase regional-scale resilience to global climate change.

Our results indicate that sedimentation and eutrophication (reinforcing stresses) are common in all regions, but differ in their intensity and co-occurrence with radiation and reducing stressors.

In the western Indian Ocean, coral locations exposed to high reinforcing stress correspond to those areas with high river runoff and sedimentation [79]–[82] (Fig. 6-l, See Appendix S4). These locations are exposed to moderate radiation stress but overall are severely exposed to high reinforcing effect of water quality from highland runoff. Local management of the coastal watershed in these areas is expected to shift the overall exposure towards lower severity grades. On the GBR, eutrophication is increasing principally due to land use in the adjacent coastal catchment area [83]–[86]. From our 1520 sample points in GBR, there is great variability but the majority of coral locations are moderately to highly exposed to water quality reinforcing stress (Fig. 4, 7). Given that the exposure of GBR reefs to radiation stresses are relatively moderate ((Fig. 6-f), a management strategy that improves water quality is predicted to increase reef resiliency [13], [78].

The central Indian Ocean lies within a different domain of exposure, where corals are exposed to high radiation stress but have little reinforcing stress, except in Sri Lanka and off India. Despite most of this region having small direct human impacts, synergistic effect of increased temperature and UV is the dominant stressor and has led to current significant coral declines associated with climatic anomalies [87]. In the most remote areas of the Chagos Islands, there is also evidence for fast reef recovery after these disturbances, which may arise from the low reinforcing stresses [7], [88]. Our results indicate that this region has low stress reducing effect from temperature variability and tidal amplitude, making it one of the most exposed to climate change alongside Micronesia and South East Asia.

In the Middle East, there was moderate to high radiation stress, with recent reports indicating exposure to high thermal anomalies [23] and similar conditions for the future [7]. Corals in the Middle East are also exposed to high levels of natural eutrophication, along with Western Australia, Eastern Pacific, and the GBR. Despite their exposure to extreme environments that are close to the limits of their thermal distribution [19], less frequent bleaching disturbances have been predicted in the future [7]. As a result, managing the highly eutrophic conditions and the chronic human impacts in these regions could possibly reduce coral decline.

In the Caribbean, coastal development—among other disturbances such as diseases and bleaching—has been associated with mortality of corals and the increase in macroalgae [74], [89]. Our study shows that coral reefs in the south western and the western boundary of the Caribbean, including Belize, reefs off Panama, Costa Rica, Colombia and Venezuela, are severely exposed to stresses, primarily due to reinforcing stress and moderate radiation stress and compounded by a low reducing effect. In Belize for example, there has been reports of high coral decline due to nutrification, bleaching, and diseases among other factors [74], [90], in agreement to our results indicating a high-severe exposure primarily due to reinforcing and radiation stresses (Fig. 2, See Appendix S4). Declines continue despite the integrated adaptive approach to marine protected area management currently in place since the late 1990's. This scenario provides an example of the difficulties of managing for both large-scale climate disturbances and the regulation of land-based sources of pollution and siltation in areas where the main sources of pollution are far away from the reefs [91], [92].

While these results are largely expected to correspond to the observed degree or extent to which coral reefs are subject to the perturbations, including the proximity to river discharges, coastal cities and agricultural areas, they may not necessarily correlate with the current reef status and observed changes in the respective regions or specific coral reef locations. Internal elements of biological and ecological adaptive capacity (i.e. genetic and species diversity, dispersal and connectivity) and sensitivity (e.g. acclimatization, overall health) that are critical to such predictions are not considered here and may explain mismatches between exposure and vulnerability. Recent model predictions are indicating that adaptations of corals through physiological and genetic changes of corals and zooxanthellae will not match the rate of temperature increase from climate change under the business-as-usual scenarios [30]. Environmental factors that counter the effects of radiation stressors or reduction of the reinforcing stress factors may play a greater role in the maintenance of the health of coral reefs.

### Management implications

The global variability in coral exposure to stresses, as evidenced by the distribution of coral locations by region in the exposure space (Fig. 6) portrays the degree to which various management strategies are locally relevant. For example, the variability of exposure among coral reef locations in the Caribbean, GBR, South East Asia, and western Indian Ocean indicate the potential for a high within-region dynamics (Fig. 6). This offers an opportunity for spatially targeted management strategies to possibly reverse the well-documented significant decline of coral reefs in these regions (e.g. [45]). While management can act to reduce the exposure to anthropogenic pressures, few if any practical large-scale options exist for reducing climate related stress. Under this framework, effective local management needs to target moving reef locations, especially those that are moderately exposed to climate related stress, towards low reinforcing conditions through improved water quality.

### Model limitations

The outputs of this study are constrained, among others, by the uncertainty conferred on the results of the membership functions and standardization algorithms. Insufficient or contradictory knowledge on the response of corals to environmental stimuli in the field and the local adaptation and species-specific responses to stress is the main limitation to creating predictive models. In addition, the use of proxies as a substitute for unavailable environment data, may limit the validity of the assumptions because of potential weak causation associated with correlation-based studies. For example, sedimentation and eutrophication proxy is used as a reinforcing variable and defined using a monotonically increasing sigmoid function, as suggested by some field studies [78]. This however contradicts other findings that suggest increased turbidity, which may result from increased chlorophyll, reduces the depth penetration of harmful UVB [36], thereby protecting corals. Similarly, high nutrients and heterotrophy associated with rich plankton and high chlorophyll may prevent the severity and impact of coral bleaching [93], [94]. In addition, the results of localized studies may not necessarily scale to an entire region [95]. The multiple interactive roles of turbidity is an example of the complex nature of multiple stressors, where even a single variable can be viewed mechanistically as multiple stressors with impacts of varying scales [1].

Our model assumes a negative linear relationship between thermal stress and SST variability whereas the relationship may be more complex [70]. Other studies evaluating large variability areas have indicated large thermal stress values in regions with the largest SST variability [9], [70]. This could result in uncertainties in areas of high SST variability in the Arabian Sea, Arabian Gulf, Eastern Pacific, Western Australia and the coast of Brazil. Further, the boundaries of our study preclude several other factors that affect coral health and an ideal systems analysis with unlimited global data for multiple threats would consider. These include: ocean acidification; fishery exploitation; hydrodynamic disturbances; abundance of bio-eroders and corallivores; and coral community structure, among others.

While the low-moderate resolution remote sensing data used in this study demonstrates sufficient variability for explaining large-scale biological processes [16], [23], a coarse grid ignores significant sub-grid details, and very often introduces approximations and uncertainties into model results [96]. The spatial and temporal aggregation, interpolation and integration of data from different spatial and temporal scales contribute to the errors from mismatch in spatial and temporal correlation structure [48].

### Conclusions

Despite the limitations described above, these results can be applied to specific reefs if they are downscaled to incorporate indicators of resilience at reef scale [21], [97]. Through the framework presented, integrating many sources of spatially explicit data and scientific knowledge has identified global spatial gradients of radiation, sedimentation and eutrophication stressors and of the key factors that reduce these stresses. This provides a better understand how coral reefs might be managed better under conditions of environmental uncertainty and complexity.

There is high spatial variability of the relative exposures of corals to radiation and reinforcing stressors. Despite radiation stress being dominant, most reef locations identified as severely exposed due to radiation and reinforcing stress are expected to have a lower severity grade if the reinforcing effect from sedimentation and eutrophication were managed. Future studies should focus on incorporating additional coral threats such as acidification, the removal of grazers, and multiple interacting stresses. Enhancement of the knowledge base of the physiological response of corals to environmental stimulus can help improve future models.

## Supporting Information

Appendix S1 **A summary of conceptual deductions of reef coral responses to environmental variables (adopted from** [**16**] **).**
(DOC)

Appendix S2 **A conceptual framework adopted for the analysis of ocean color data.**
(TIF)

Appendix S3 **Normal cumulative density functions fitted on respective environmental parameters (log transformed except for SST and UV).**
(TIF)

Appendix S4 **A table of coral exposure indices i.e. radiation, reducing, reinforcing, each set of coordinates represents coral reef location within respective ocean provinces.**
(DOC)
