# Supplementary material for: Global Gradients of Coral Exposure to Environmental Stresses and Implications for Local Management
Source: PLoS One. 2011 Aug 10;6(8):e23064. doi: 10.1371/journal.pone.0023064 (PMC3156087; doi:10.1371/journal.pone.0023064)
Supplement: Appendix S1 — A summary of conceptual deductions of reef coral responses to environmental variables (adopted from [16] ). (DOC) [file pone.0023064.s001.doc]

| **Variable** | **Proxy/derived variable** | **Response and direction** | **Reference** | **Proxy data Source** |
| --- | --- | --- | --- | --- |
| Sea surface temperature | Thermal Stress Anomaly | Positive correlation with coral bleaching and diseases | Bruno et al. 2007; Selig and Bruno 2010; Bruno | NOAA SST |
|  | Sea surface temperature anomaly | Positive correlation with coral bleaching and diseases | Bruno et al. 2007; Selig and Bruno 2010 | (CoRTAD database) |
|  | Long term mean | Positive correlation with bleaching | Maina et al. 2008 | NOAA AVHRR |
|  | SST variability | Negative correlation with bleaching; non-linear relationship; non linear relationship with coral growth and survival. | Potts and Swart 1984; McClanahan et al. 2007; Maina et al. 2008; Ateweberhan and McClanahan 2010 | NOAA AVHRR, JCOMM,HADSST |
| Tides | Tidal variability | Large tidal variability may create stress tolerance in corals on shallow reefs. | Brown 1997; Anthony et al. 2004; Storlazzi et al. 2004; Anthony et al. 2007 | FES2004 model |
| UV | Erythermal exposure | Tissue damage, stress on coral and zooxanthellae, reduced photosynthetic efficiency particularly in combination with elevated temperatures | Dunne and Brown 2001; Fitt et al. 2001; Jokiel et al. 2004; Lesser and Farrell 2004; Ferrier-Pages et al. 2007 | TOMS |
| Wind speed | Wind speed and low wind (doldrums) duration | as wind speed falls, vertical mixing decreases, resulting in decreased evaporative cooling and transfer of deeper cool water, which increases the likelihood of thermal stress on corals | Hoegh-Guldberg 1999; Dunne and Brown 2001; Mumby et al. 2001 | Sea winds Scaterometers |
| Particulate matter | Chlorophyll a | High chlorophyll levels may represent stressful eutrophic environments; high chlorophyll a concentration also may shade corals from the effects strong irradiation levels. High chlorophyll is also associated with turbid coastal zones influenced by land drainage or sediment re-suspension. Here chlorophyll is used as an indicator of sedimentation and eutrophication. | Szmant and Forrester 1996; Fabricius 2005; Weber et al. 2006; Cooper et al. 2007; Wooldridge 2009 | Merged MODIS, MERIS, SeaWIFS |
|  | Total Suspended Matter (TSM) | Stresses corals; causes mortality to corals | Cortes and Risk 1985; Rogers 1990; Nugues and Roberts 2003; Fabricius 2005; Fabricius et al. 2005 | MERIS |

Appendix S1. A summary of conceptual deductions of reef coral responses to environmental variables (adopted from Maina et al., 2008)..
