# Supplementary figures and images for: Global Gradients of Coral Exposure to Environmental Stresses and Implications for Local Management
Source: PLoS One. 2011 Aug 10;6(8):e23064. doi: 10.1371/journal.pone.0023064 (PMC3156087; doi:10.1371/journal.pone.0023064)

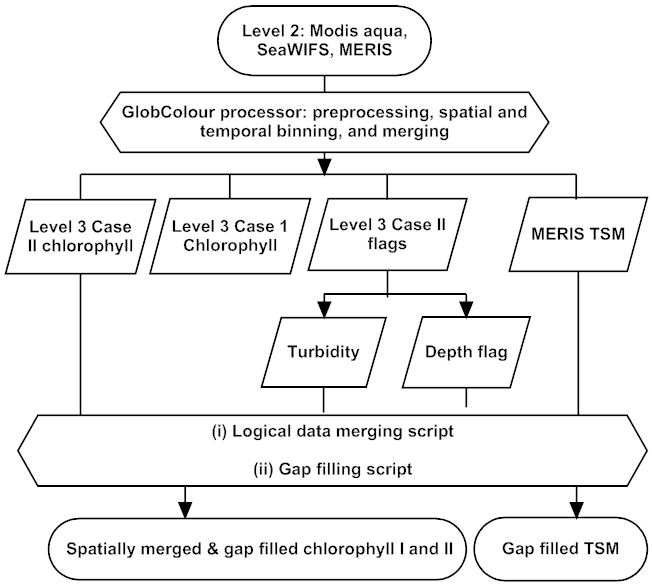

Supplement: Appendix S2 — A conceptual framework adopted for the analysis of ocean color data. (TIF) [file pone.0023064.s002.tif]

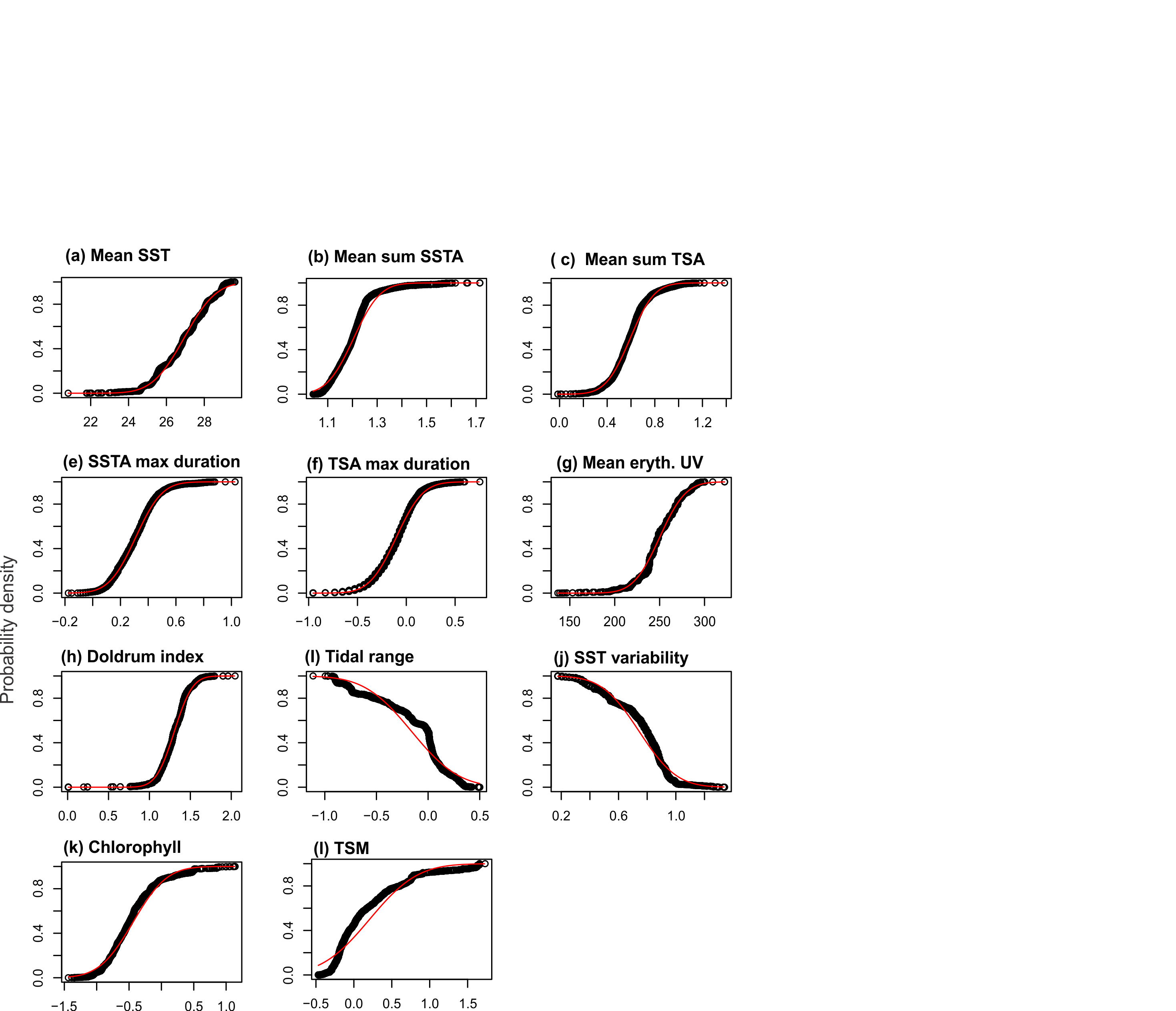

Supplement: Appendix S3 — Normal cumulative density functions fitted on respective environmental parameters (log transformed except for SST and UV). (TIF) [file pone.0023064.s003.tif]
