# Supplementary material for: Global Gradients of Coral Exposure to Environmental Stresses and Implications for Local Management
Source: PLoS One. 2011 Aug 10;6(8):e23064. doi: 10.1371/journal.pone.0023064 (PMC3156087; doi:10.1371/journal.pone.0023064)
Supplement: Appendix S4 — A table of coral exposure indices i.e. radiation, reducing, reinforcing, each set of coordinates represents coral reef location within respective ocean provinces. (DOC) [file pone.0023064.s004.doc]

| Region | Lat | Lon | Radiation | Reducing | Radiation&reducing | Reinforcing | model | VulnerabilityClass |
| --- | --- | --- | --- | --- | --- | --- | --- | --- |
| Western Indian Ocean | -30.27 | 30.84 | 0.3 | 0.3 | 0.2 | 1.0 | 1.0 | Severe |
| Western Indian Ocean | -30.27 | 30.83 | 0.3 | 0.3 | 0.2 | 1.0 | 1.0 | Severe |
| Western Indian Ocean | -30.10 | 31.12 | 0.2 | 0.3 | 0.1 | 0.8 | 0.9 | Severe |
| Western Indian Ocean | -28.87 | 34.45 | 0.2 | 0.3 | 0.2 | 0.2 | 0.3 | Low |
| Western Australia | -28.68 | 113.83 | 0.5 | 0.3 | 0.4 | 0.5 | 0.7 | High |
| Great Barrier Reef | -28.66 | 153.63 | 0.4 | 0.2 | 0.3 | 0.8 | 0.9 | Severe |
| Western Indian Ocean | -28.50 | 33.50 | 0.1 | 0.3 | 0.1 | 0.2 | 0.3 | Low |
| Western Indian Ocean | -27.87 | 32.61 | 0.1 | 0.3 | 0.1 | 0.3 | 0.4 | Medial |
| Western Indian Ocean | -27.59 | 32.74 | 0.1 | 0.3 | 0.1 | 0.4 | 0.5 | Medial |
| Western Indian Ocean | -27.54 | 34.69 | 0.2 | 0.3 | 0.2 | 0.2 | 0.3 | Low |
| Polynesia | -27.17 | -109.33 | 0.4 | 0.2 | 0.3 | 0.0 | 0.3 | Low |
| Western Indian Ocean | -26.93 | 32.89 | 0.4 | 0.2 | 0.3 | 0.4 | 0.5 | Medial |
| Western Indian Ocean | -26.84 | 32.91 | 0.5 | 0.2 | 0.3 | 0.4 | 0.6 | Medial |
| Western Indian Ocean | -26.82 | 32.90 | 0.5 | 0.2 | 0.3 | 0.4 | 0.6 | Medial |
| Great Barrier Reef | -26.65 | 153.18 | 0.2 | 0.2 | 0.1 | 0.6 | 0.6 | Medial |
| Great Barrier Reef | -26.65 | 153.33 | 0.2 | 0.2 | 0.1 | 0.4 | 0.5 | Medial |
| Great Barrier Reef | -26.17 | 154.88 | 0.6 | 0.2 | 0.4 | 0.1 | 0.4 | Medial |
| Western Indian Ocean | -26.10 | 32.88 | 0.4 | 0.2 | 0.3 | 0.8 | 0.9 | Severe |
| Western Indian Ocean | -26.05 | 32.89 | 0.3 | 0.2 | 0.2 | 0.8 | 0.9 | Severe |
| Western Indian Ocean | -26.04 | 33.01 | 0.1 | 0.2 | 0.1 | 0.6 | 0.6 | Medial |
| Western Indian Ocean | -26.02 | 32.90 | 0.3 | 0.2 | 0.2 | 0.8 | 0.9 | Severe |
| Western Indian Ocean | -26.00 | 32.92 | 0.2 | 0.2 | 0.1 | 0.8 | 0.9 | Severe |
| Western Indian Ocean | -26.00 | 33.50 | 0.1 | 0.2 | 0.1 | 0.5 | 0.6 | Medial |
| Western Indian Ocean | -25.96 | 33.01 | 0.1 | 0.2 | 0.1 | 0.7 | 0.7 | High |
| Western Indian Ocean | -25.91 | 33.05 | 0.1 | 0.2 | 0.1 | 0.6 | 0.6 | Medial |
| Western Indian Ocean | -25.90 | 33.05 | 0.1 | 0.2 | 0.1 | 0.6 | 0.6 | Medial |
| Western Indian Ocean | -25.44 | 33.17 | 0.2 | 0.2 | 0.2 | 0.8 | 0.8 | Severe |
| Great Barrier Reef | -24.93 | 152.50 | 0.7 | 0.1 | 0.5 | 0.7 | 0.9 | Severe |
| Western Indian Ocean | -24.61 | 35.27 | 0.2 | 0.3 | 0.2 | 0.8 | 0.8 | High |
| Great Barrier Reef | -24.11 | 152.72 | 0.4 | 0.2 | 0.2 | 0.3 | 0.5 | Medial |
| Great Barrier Reef | -24.01 | 151.73 | 0.6 | 0.1 | 0.4 | 0.6 | 0.8 | High |
| Great Barrier Reef | -24.01 | 151.72 | 0.6 | 0.1 | 0.4 | 0.6 | 0.8 | High |
| Great Barrier Reef | -24.00 | 151.78 | 0.6 | 0.1 | 0.4 | 0.6 | 0.8 | High |
| Great Barrier Reef | -24.00 | 151.60 | 0.7 | 0.1 | 0.5 | 0.7 | 0.8 | Severe |
| Great Barrier Reef | -23.98 | 151.77 | 0.5 | 0.1 | 0.4 | 0.6 | 0.8 | High |
| Great Barrier Reef | -23.98 | 151.66 | 0.5 | 0.1 | 0.4 | 0.7 | 0.8 | High |
| Great Barrier Reef | -23.97 | 151.48 | 0.7 | 0.1 | 0.5 | 0.7 | 0.9 | Severe |
| Great Barrier Reef | -23.97 | 151.63 | 0.5 | 0.1 | 0.4 | 0.9 | 0.9 | Severe |
| Great Barrier Reef | -23.96 | 151.48 | 0.7 | 0.1 | 0.5 | 0.7 | 0.9 | Severe |
| Great Barrier Reef | -23.94 | 151.47 | 0.6 | 0.1 | 0.4 | 0.7 | 0.8 | Severe |
| Great Barrier Reef | -23.91 | 152.41 | 0.2 | 0.1 | 0.2 | 0.3 | 0.4 | Medial |
| Great Barrier Reef | -23.91 | 152.41 | 0.2 | 0.1 | 0.2 | 0.3 | 0.4 | Medial |
| Great Barrier Reef | -23.90 | 152.41 | 0.2 | 0.1 | 0.2 | 0.3 | 0.4 | Medial |
| Great Barrier Reef | -23.86 | 152.37 | 0.2 | 0.1 | 0.2 | 0.4 | 0.5 | Medial |
| Great Barrier Reef | -23.86 | 152.37 | 0.2 | 0.1 | 0.2 | 0.4 | 0.5 | Medial |
| Great Barrier Reef | -23.81 | 152.29 | 0.3 | 0.1 | 0.2 | 0.4 | 0.5 | Medial |
| Great Barrier Reef | -23.81 | 152.29 | 0.3 | 0.1 | 0.2 | 0.4 | 0.5 | Medial |
| Great Barrier Reef | -23.76 | 152.27 | 0.3 | 0.1 | 0.2 | 0.5 | 0.6 | Medial |
| Great Barrier Reef | -23.76 | 152.27 | 0.3 | 0.1 | 0.2 | 0.4 | 0.5 | Medial |
| Great Barrier Reef | -23.70 | 152.18 | 0.4 | 0.1 | 0.2 | 0.5 | 0.6 | Medial |
| Western Indian Ocean | -23.70 | 43.66 | 0.8 | 0.2 | 0.6 | 0.9 | 1.0 | Severe |
| Great Barrier Reef | -23.70 | 152.18 | 0.4 | 0.1 | 0.2 | 0.5 | 0.6 | Medial |
| Great Barrier Reef | -23.63 | 152.15 | 0.3 | 0.1 | 0.2 | 0.5 | 0.6 | Medial |
| Great Barrier Reef | -23.60 | 152.04 | 0.5 | 0.1 | 0.3 | 0.5 | 0.7 | High |
| Great Barrier Reef | -23.60 | 152.05 | 0.5 | 0.1 | 0.3 | 0.5 | 0.7 | High |
| Great Barrier Reef | -23.56 | 151.68 | 0.5 | 0.1 | 0.3 | 0.5 | 0.7 | Medial |
| Great Barrier Reef | -23.54 | 151.74 | 0.5 | 0.1 | 0.3 | 0.5 | 0.7 | Medial |
| Great Barrier Reef | -23.54 | 151.74 | 0.5 | 0.1 | 0.3 | 0.5 | 0.7 | Medial |
| Great Barrier Reef | -23.54 | 151.27 | 0.5 | 0.1 | 0.4 | 0.6 | 0.7 | High |
| Great Barrier Reef | -23.53 | 151.28 | 0.5 | 0.1 | 0.4 | 0.6 | 0.7 | High |
| Great Barrier Reef | -23.50 | 151.77 | 0.5 | 0.1 | 0.3 | 0.5 | 0.7 | High |
| Great Barrier Reef | -23.50 | 151.77 | 0.5 | 0.1 | 0.3 | 0.5 | 0.7 | High |
| Great Barrier Reef | -23.50 | 152.06 | 0.3 | 0.1 | 0.2 | 0.5 | 0.6 | Medial |
| Western Indian Ocean | -23.50 | 35.50 | 0.5 | 0.3 | 0.4 | 0.6 | 0.8 | High |
| Great Barrier Reef | -23.50 | 152.06 | 0.3 | 0.1 | 0.2 | 0.5 | 0.6 | Medial |
| Western Indian Ocean | -23.49 | 43.75 | 0.7 | ? | 0.5 | 0.8 | 0.9 | Severe |
| Great Barrier Reef | -23.49 | 151.23 | 0.5 | 0.1 | 0.3 | 0.6 | 0.8 | High |
| Great Barrier Reef | -23.49 | 152.09 | 0.3 | 0.1 | 0.2 | 0.5 | 0.6 | Medial |
| Great Barrier Reef | -23.47 | 151.88 | 0.4 | 0.1 | 0.3 | 0.5 | 0.7 | High |
| Great Barrier Reef | -23.47 | 151.88 | 0.4 | 0.1 | 0.3 | 0.5 | 0.7 | High |
| Great Barrier Reef | -23.47 | 151.88 | 0.4 | 0.1 | 0.3 | 0.5 | 0.7 | High |
| Great Barrier Reef | -23.45 | 151.96 | 0.4 | 0.1 | 0.3 | 0.5 | 0.7 | Medial |
| Great Barrier Reef | -23.45 | 151.96 | 0.4 | 0.1 | 0.3 | 0.5 | 0.7 | Medial |
| Great Barrier Reef | -23.45 | 151.97 | 0.4 | 0.1 | 0.3 | 0.5 | 0.7 | Medial |
| Great Barrier Reef | -23.45 | 151.91 | 0.3 | 0.1 | 0.2 | 0.5 | 0.6 | Medial |
| Great Barrier Reef | -23.44 | 151.93 | 0.4 | 0.1 | 0.3 | 0.5 | 0.7 | Medial |
| Great Barrier Reef | -23.44 | 152.03 | 0.3 | 0.1 | 0.2 | 0.5 | 0.6 | Medial |
| Western Indian Ocean | -23.42 | 43.65 | 0.7 | 0.2 | 0.5 | 0.8 | 0.9 | Severe |
| Great Barrier Reef | -23.42 | 151.17 | 0.6 | 0.1 | 0.4 | 0.7 | 0.8 | High |
| Western Indian Ocean | -23.42 | 43.67 | 0.7 | 0.2 | 0.5 | 0.8 | 0.9 | Severe |
| Great Barrier Reef | -23.42 | 151.17 | 0.6 | 0.1 | 0.4 | 0.7 | 0.8 | High |
| Great Barrier Reef | -23.41 | 151.18 | 0.5 | 0.1 | 0.4 | 0.6 | 0.8 | High |
| Great Barrier Reef | -23.41 | 151.18 | 0.5 | 0.1 | 0.4 | 0.6 | 0.8 | High |
| Western Indian Ocean | -23.40 | 43.64 | 0.7 | 0.2 | 0.5 | 0.8 | 0.9 | Severe |
| Great Barrier Reef | -23.40 | 151.15 | 0.6 | 0.1 | 0.4 | 0.7 | 0.8 | High |
| Great Barrier Reef | -23.40 | 151.15 | 0.6 | 0.1 | 0.4 | 0.7 | 0.8 | High |
| Western Indian Ocean | -23.38 | 43.72 | 0.7 | ? | 0.6 | 1.0 | 1.0 | Severe |
| Great Barrier Reef | -23.35 | 150.94 | 0.6 | 0.1 | 0.4 | 0.9 | 0.9 | Severe |
| Great Barrier Reef | -23.33 | 151.96 | 0.3 | 0.1 | 0.2 | 0.5 | 0.6 | Medial |
| Great Barrier Reef | -23.33 | 151.97 | 0.3 | 0.1 | 0.2 | 0.5 | 0.6 | Medial |
| Great Barrier Reef | -23.32 | 151.98 | 0.3 | 0.1 | 0.2 | 0.5 | 0.6 | Medial |
| Western Indian Ocean | -23.32 | 36.72 | 0.3 | 0.3 | 0.3 | 0.1 | 0.4 | Medial |
| Great Barrier Reef | -23.30 | 150.92 | 0.6 | 0.1 | 0.4 | 0.9 | 0.9 | Severe |
| Great Barrier Reef | -23.30 | 151.77 | 0.4 | 0.1 | 0.3 | 0.5 | 0.7 | High |
| Great Barrier Reef | -23.29 | 151.89 | 0.3 | 0.1 | 0.2 | 0.5 | 0.6 | Medial |
| Great Barrier Reef | -23.29 | 151.89 | 0.3 | 0.1 | 0.2 | 0.5 | 0.6 | Medial |
| Great Barrier Reef | -23.27 | 151.93 | 0.3 | 0.1 | 0.2 | 0.5 | 0.6 | Medial |
| Great Barrier Reef | -23.25 | 151.79 | 0.4 | 0.1 | 0.3 | 0.5 | 0.7 | Medial |
| Great Barrier Reef | -23.22 | 150.96 | 0.7 | 0.1 | 0.4 | 0.9 | 0.9 | Severe |
| Great Barrier Reef | -23.21 | 150.97 | 0.7 | 0.1 | 0.4 | 0.9 | 0.9 | Severe |
| Great Barrier Reef | -23.21 | 150.97 | 0.7 | 0.1 | 0.4 | 0.9 | 0.9 | Severe |
| Great Barrier Reef | -23.21 | 150.97 | 0.7 | 0.1 | 0.4 | 0.9 | 0.9 | Severe |
| Great Barrier Reef | -23.20 | 151.10 | 0.6 | 0.1 | 0.4 | 0.6 | 0.7 | High |
| Great Barrier Reef | -23.20 | 151.10 | 0.6 | 0.1 | 0.4 | 0.6 | 0.7 | High |
| Great Barrier Reef | -23.20 | 151.10 | 0.6 | 0.1 | 0.4 | 0.6 | 0.7 | High |
| Great Barrier Reef | -23.20 | 151.10 | 0.6 | 0.1 | 0.4 | 0.6 | 0.7 | High |
| Great Barrier Reef | -23.19 | 150.93 | 0.7 | 0.1 | 0.4 | 0.9 | 0.9 | Severe |
| Great Barrier Reef | -23.19 | 150.91 | 0.7 | 0.1 | 0.4 | 0.9 | 0.9 | Severe |
| Great Barrier Reef | -23.18 | 151.91 | 0.3 | 0.1 | 0.2 | 0.5 | 0.6 | Medial |
| Western Indian Ocean | -23.18 | 43.59 | 0.6 | ? | 0.5 | 1.0 | 1.0 | Severe |
| Great Barrier Reef | -23.17 | 150.91 | 0.7 | 0.1 | 0.4 | 0.9 | 0.9 | Severe |
| Great Barrier Reef | -23.17 | 150.91 | 0.7 | 0.1 | 0.4 | 0.9 | 0.9 | Severe |
| Great Barrier Reef | -23.17 | 150.92 | 0.7 | 0.1 | 0.4 | 0.9 | 0.9 | Severe |
| Western Indian Ocean | -23.17 | 43.59 | 0.6 | ? | 0.5 | 1.0 | 1.0 | Severe |
| Great Barrier Reef | -23.16 | 151.08 | 0.6 | 0.1 | 0.4 | 0.7 | 0.8 | High |
| Great Barrier Reef | -23.16 | 151.07 | 0.6 | 0.1 | 0.4 | 0.7 | 0.8 | High |
| Great Barrier Reef | -23.16 | 151.07 | 0.6 | 0.1 | 0.4 | 0.7 | 0.8 | High |
| Great Barrier Reef | -23.16 | 151.08 | 0.6 | 0.1 | 0.4 | 0.7 | 0.8 | High |
| Great Barrier Reef | -23.16 | 151.07 | 0.6 | 0.1 | 0.4 | 0.7 | 0.8 | High |
| Great Barrier Reef | -23.16 | 150.92 | 0.7 | 0.1 | 0.4 | 0.9 | 0.9 | Severe |
| Great Barrier Reef | -23.16 | 150.96 | 0.6 | 0.1 | 0.4 | 0.8 | 0.9 | Severe |
| Great Barrier Reef | -23.16 | 150.96 | 0.6 | 0.1 | 0.4 | 0.8 | 0.9 | Severe |
| Great Barrier Reef | -23.16 | 150.96 | 0.6 | 0.1 | 0.4 | 0.8 | 0.9 | Severe |
| Western Indian Ocean | -23.16 | 43.58 | 0.6 | ? | 0.5 | 1.0 | 1.0 | Severe |
| Great Barrier Reef | -23.16 | 151.08 | 0.6 | 0.1 | 0.4 | 0.6 | 0.8 | High |
| Great Barrier Reef | -23.15 | 151.09 | 0.5 | 0.1 | 0.3 | 0.6 | 0.7 | High |
| Great Barrier Reef | -23.15 | 150.97 | 0.6 | 0.1 | 0.4 | 0.8 | 0.9 | Severe |
| Great Barrier Reef | -23.15 | 150.90 | 0.6 | 0.1 | 0.4 | 0.9 | 0.9 | Severe |
| Great Barrier Reef | -23.15 | 150.90 | 0.6 | 0.1 | 0.4 | 0.9 | 0.9 | Severe |
| Great Barrier Reef | -23.15 | 150.98 | 0.6 | 0.1 | 0.4 | 0.8 | 0.9 | Severe |
| Great Barrier Reef | -23.15 | 150.91 | 0.6 | 0.1 | 0.4 | 0.9 | 0.9 | Severe |
| Western Indian Ocean | -23.15 | 43.59 | 0.6 | ? | 0.5 | 1.0 | 1.0 | Severe |
| Great Barrier Reef | -23.12 | 150.99 | 0.6 | 0.1 | 0.4 | 0.8 | 0.9 | Severe |
| Western Indian Ocean | -23.11 | 35.57 | 0.5 | 0.3 | 0.4 | 0.5 | 0.7 | High |
| Great Barrier Reef | -23.10 | 150.90 | 0.7 | 0.1 | 0.4 | 0.9 | 0.9 | Severe |
| Great Barrier Reef | -23.10 | 150.90 | 0.7 | 0.1 | 0.4 | 0.9 | 0.9 | Severe |
| Great Barrier Reef | -23.10 | 150.88 | 0.7 | 0.1 | 0.4 | 0.9 | 0.9 | Severe |
| Great Barrier Reef | -23.10 | 150.88 | 0.7 | 0.1 | 0.4 | 0.9 | 0.9 | Severe |
| Great Barrier Reef | -23.09 | 150.90 | 0.7 | 0.1 | 0.4 | 0.9 | 0.9 | Severe |
| Great Barrier Reef | -23.08 | 150.89 | 0.7 | 0.1 | 0.4 | 0.9 | 0.9 | Severe |
| Great Barrier Reef | -23.08 | 150.89 | 0.7 | 0.1 | 0.4 | 0.9 | 0.9 | Severe |
| Great Barrier Reef | -23.07 | 150.96 | 0.6 | 0.1 | 0.4 | 0.7 | 0.8 | High |
| Great Barrier Reef | -23.05 | 150.88 | 0.6 | 0.1 | 0.4 | 0.8 | 0.9 | Severe |
| Great Barrier Reef | -23.05 | 150.88 | 0.6 | 0.1 | 0.4 | 0.8 | 0.9 | Severe |
| Great Barrier Reef | -23.04 | 150.88 | 0.6 | 0.1 | 0.4 | 0.8 | 0.9 | Severe |
| Great Barrier Reef | -23.04 | 150.88 | 0.6 | 0.1 | 0.4 | 0.8 | 0.9 | Severe |
| Western Indian Ocean | -22.96 | 43.35 | 0.5 | 0.2 | 0.4 | 0.6 | 0.7 | High |
| Great Barrier Reef | -22.65 | 166.30 | 0.6 | 0.3 | 0.5 | 0.1 | 0.5 | Medial |
| Great Barrier Reef | -22.56 | 166.33 | 0.6 | 0.3 | 0.5 | 0.1 | 0.5 | Medial |
| Great Barrier Reef | -22.49 | 166.44 | 0.6 | 0.3 | 0.4 | 0.7 | 0.8 | High |
| Great Barrier Reef | -22.43 | 166.37 | 0.6 | 0.3 | 0.4 | 0.7 | 0.8 | High |
| Great Barrier Reef | -22.42 | 166.33 | 0.6 | 0.3 | 0.4 | 0.7 | 0.8 | High |
| Great Barrier Reef | -22.39 | 166.53 | 0.6 | 0.3 | 0.4 | 0.8 | 0.9 | Severe |
| Great Barrier Reef | -22.39 | 152.63 | 0.3 | 0.2 | 0.2 | 0.3 | 0.5 | Medial |
| Great Barrier Reef | -22.38 | 152.56 | 0.3 | 0.2 | 0.3 | 0.3 | 0.5 | Medial |
| Great Barrier Reef | -22.36 | 152.72 | 0.3 | 0.3 | 0.2 | 0.1 | 0.3 | Low |
| Great Barrier Reef | -22.36 | 152.72 | 0.3 | 0.3 | 0.2 | 0.1 | 0.3 | Low |
| Great Barrier Reef | -22.36 | 152.69 | 0.2 | 0.2 | 0.2 | 0.1 | 0.3 | Low |
| Great Barrier Reef | -22.35 | 152.69 | 0.2 | 0.2 | 0.2 | 0.1 | 0.3 | Low |
| Great Barrier Reef | -22.35 | 152.73 | 0.3 | 0.3 | 0.2 | 0.1 | 0.3 | Low |
| Great Barrier Reef | -22.35 | 152.73 | 0.3 | 0.3 | 0.2 | 0.1 | 0.3 | Low |
| Great Barrier Reef | -22.34 | 166.42 | 0.6 | 0.3 | 0.4 | 0.8 | 0.9 | Severe |
| Western Indian Ocean | -22.34 | 40.46 | 0.6 | 0.3 | 0.5 | 0.1 | 0.5 | Medial |
| Great Barrier Reef | -22.33 | 152.72 | 0.3 | 0.3 | 0.2 | 0.1 | 0.3 | Low |
| Great Barrier Reef | -22.32 | 152.72 | 0.3 | 0.3 | 0.2 | 0.1 | 0.3 | Low |
| Great Barrier Reef | -22.30 | 152.73 | 0.3 | 0.3 | 0.2 | 0.1 | 0.3 | Low |
| Great Barrier Reef | -22.30 | 166.02 | 0.7 | 0.3 | 0.5 | 0.1 | 0.5 | Medial |
| Great Barrier Reef | -22.30 | 152.73 | 0.3 | 0.3 | 0.2 | 0.1 | 0.3 | Low |
| Great Barrier Reef | -22.29 | 166.19 | 0.6 | 0.3 | 0.4 | 0.4 | 0.7 | High |
| Great Barrier Reef | -22.27 | 152.73 | 0.2 | 0.3 | 0.2 | 0.1 | 0.3 | Low |
| Great Barrier Reef | -22.27 | 152.73 | 0.2 | 0.3 | 0.2 | 0.1 | 0.3 | Low |
| Great Barrier Reef | -22.26 | 152.40 | 0.4 | 0.2 | 0.3 | 0.2 | 0.4 | Medial |
| Western Indian Ocean | -22.25 | 43.19 | 0.4 | 0.2 | 0.4 | 0.9 | 0.9 | Severe |
| Great Barrier Reef | -22.25 | 166.27 | 0.6 | 0.3 | 0.4 | 0.7 | 0.9 | Severe |
| Western Indian Ocean | -22.23 | 43.22 | 0.5 | ? | 0.5 | 0.8 | 0.9 | Severe |
| Great Barrier Reef | -22.23 | 150.31 | 0.5 | 0.1 | 0.3 | 0.5 | 0.7 | Medial |
| Western Indian Ocean | -22.22 | 43.18 | 0.5 | 0.2 | 0.4 | 0.7 | 0.8 | Severe |
| Great Barrier Reef | -22.22 | 166.32 | 0.6 | 0.2 | 0.5 | 0.8 | 0.9 | Severe |
| Western Australia | -22.22 | 113.82 | 0.5 | 0.3 | 0.4 | 0.6 | 0.8 | High |
| Great Barrier Reef | -22.21 | 152.75 | 0.2 | 0.3 | 0.2 | 0.1 | 0.3 | Low |
| Great Barrier Reef | -22.21 | 152.75 | 0.2 | 0.3 | 0.2 | 0.1 | 0.3 | Low |
| Great Barrier Reef | -22.20 | 152.49 | 0.3 | 0.2 | 0.3 | 0.2 | 0.4 | Medial |
| Western Indian Ocean | -22.20 | 43.21 | 0.5 | ? | 0.5 | 0.8 | 0.9 | Severe |
| Great Barrier Reef | -22.19 | 152.76 | 0.2 | 0.3 | 0.2 | 0.1 | 0.3 | Low |
| Great Barrier Reef | -22.19 | 152.76 | 0.2 | 0.3 | 0.2 | 0.1 | 0.3 | Low |
| Great Barrier Reef | -22.18 | 150.36 | 0.4 | 0.1 | 0.3 | 0.5 | 0.6 | Medial |
| Great Barrier Reef | -22.18 | 152.70 | 0.3 | 0.3 | 0.2 | 0.1 | 0.3 | Low |
| Great Barrier Reef | -22.17 | 152.44 | 0.3 | 0.2 | 0.2 | 0.2 | 0.4 | Medial |
| Great Barrier Reef | -22.16 | 152.55 | 0.3 | 0.3 | 0.3 | 0.1 | 0.4 | Medial |
| Great Barrier Reef | -22.15 | 152.76 | 0.2 | 0.3 | 0.2 | 0.1 | 0.3 | Low |
| Great Barrier Reef | -22.15 | 152.76 | 0.2 | 0.3 | 0.2 | 0.1 | 0.3 | Low |
| Great Barrier Reef | -22.15 | 152.53 | 0.3 | 0.3 | 0.2 | 0.1 | 0.4 | Medial |
| Western Indian Ocean | -22.12 | 43.20 | 0.4 | 0.2 | 0.4 | 0.9 | 0.9 | Severe |
| Great Barrier Reef | -22.12 | 152.74 | 0.3 | 0.3 | 0.2 | 0.1 | 0.3 | Low |
| Great Barrier Reef | -22.12 | 152.74 | 0.3 | 0.3 | 0.2 | 0.1 | 0.3 | Low |
| Great Barrier Reef | -22.11 | 152.53 | 0.3 | 0.3 | 0.2 | 0.1 | 0.4 | Medial |
| Great Barrier Reef | -22.10 | 152.74 | 0.2 | 0.3 | 0.2 | 0.1 | 0.3 | Low |
| Great Barrier Reef | -22.10 | 152.74 | 0.2 | 0.3 | 0.2 | 0.1 | 0.3 | Low |
| Great Barrier Reef | -22.08 | 150.67 | 0.6 | 0.1 | 0.4 | 0.5 | 0.7 | High |
| Great Barrier Reef | -22.08 | 152.52 | 0.2 | 0.3 | 0.2 | 0.1 | 0.3 | Low |
| Great Barrier Reef | -22.06 | 152.50 | 0.2 | 0.3 | 0.2 | 0.1 | 0.3 | Low |
| Great Barrier Reef | -22.06 | 152.74 | 0.2 | 0.3 | 0.2 | 0.1 | 0.3 | Low |
| Great Barrier Reef | -22.06 | 152.74 | 0.2 | 0.3 | 0.2 | 0.1 | 0.3 | Low |
| Great Barrier Reef | -22.03 | 150.30 | 0.5 | 0.1 | 0.3 | 0.5 | 0.7 | Medial |
| Great Barrier Reef | -22.03 | 150.65 | 0.6 | 0.1 | 0.4 | 0.5 | 0.7 | High |
| Western Indian Ocean | -22.03 | 43.19 | 0.4 | 0.3 | 0.4 | 0.9 | 0.9 | Severe |
| Great Barrier Reef | -22.02 | 150.37 | 0.5 | 0.1 | 0.3 | 0.5 | 0.7 | Medial |
| Great Barrier Reef | -22.01 | 152.45 | 0.3 | 0.3 | 0.3 | 0.1 | 0.4 | Medial |
| Great Barrier Reef | -22.01 | 152.73 | 0.2 | 0.3 | 0.2 | 0.1 | 0.3 | Low |
| Great Barrier Reef | -22.01 | 152.73 | 0.2 | 0.3 | 0.2 | 0.1 | 0.3 | Low |
| Great Barrier Reef | -22.01 | 150.38 | 0.4 | 0.1 | 0.3 | 0.5 | 0.7 | High |
| Great Barrier Reef | -22.00 | 152.67 | 0.3 | 0.3 | 0.2 | 0.1 | 0.3 | Low |
| Great Barrier Reef | -21.99 | 150.17 | 0.5 | 0.1 | 0.3 | 0.5 | 0.7 | High |
| Great Barrier Reef | -21.98 | 152.48 | 0.3 | 0.3 | 0.3 | 0.1 | 0.4 | Medial |
| Great Barrier Reef | -21.98 | 150.19 | 0.5 | 0.1 | 0.3 | 0.5 | 0.7 | High |
| Great Barrier Reef | -21.98 | 152.48 | 0.3 | 0.3 | 0.3 | 0.1 | 0.4 | Medial |
| Great Barrier Reef | -21.97 | 152.70 | 0.2 | 0.3 | 0.2 | 0.1 | 0.3 | Low |
| Great Barrier Reef | -21.97 | 152.70 | 0.2 | 0.3 | 0.2 | 0.1 | 0.3 | Low |
| Great Barrier Reef | -21.96 | 150.68 | 0.6 | 0.1 | 0.4 | 0.5 | 0.7 | High |
| Great Barrier Reef | -21.95 | 150.13 | 0.5 | 0.1 | 0.3 | 0.5 | 0.7 | High |
| Great Barrier Reef | -21.95 | 152.43 | 0.3 | 0.3 | 0.3 | 0.2 | 0.4 | Medial |
| Great Barrier Reef | -21.94 | 152.68 | 0.2 | 0.3 | 0.2 | 0.1 | 0.3 | Low |
| Great Barrier Reef | -21.94 | 152.46 | 0.3 | 0.3 | 0.3 | 0.1 | 0.4 | Low |
| Great Barrier Reef | -21.94 | 152.69 | 0.2 | 0.3 | 0.2 | 0.1 | 0.3 | Low |
| Great Barrier Reef | -21.93 | 152.49 | 0.3 | 0.3 | 0.2 | 0.1 | 0.3 | Low |
| Great Barrier Reef | -21.93 | 150.10 | 0.5 | 0.1 | 0.3 | 0.5 | 0.7 | High |
| Great Barrier Reef | -21.92 | 150.23 | 0.5 | 0.1 | 0.3 | 0.5 | 0.7 | High |
| Great Barrier Reef | -21.91 | 152.67 | 0.2 | 0.3 | 0.2 | 0.1 | 0.3 | Low |
| Great Barrier Reef | -21.91 | 152.67 | 0.2 | 0.3 | 0.2 | 0.1 | 0.3 | Low |
| Western Indian Ocean | -21.90 | 35.52 | 0.2 | 0.3 | 0.2 | 0.4 | 0.5 | Medial |
| Great Barrier Reef | -21.90 | 152.66 | 0.2 | 0.3 | 0.2 | 0.1 | 0.3 | Low |
| Great Barrier Reef | -21.90 | 152.66 | 0.2 | 0.3 | 0.2 | 0.1 | 0.3 | Low |
| Great Barrier Reef | -21.88 | 152.45 | 0.3 | 0.3 | 0.3 | 0.1 | 0.4 | Medial |
| Great Barrier Reef | -21.87 | 152.19 | 0.3 | 0.3 | 0.2 | 0.2 | 0.4 | Medial |
| Western Australia | -21.85 | 113.96 | 0.6 | 0.2 | 0.5 | 0.6 | 0.8 | High |
| Great Barrier Reef | -21.85 | 152.64 | 0.3 | 0.3 | 0.2 | 0.1 | 0.3 | Low |
| Great Barrier Reef | -21.85 | 152.64 | 0.3 | 0.3 | 0.2 | 0.1 | 0.3 | Low |
| Great Barrier Reef | -21.84 | 152.47 | 0.3 | 0.3 | 0.2 | 0.1 | 0.3 | Low |
| Great Barrier Reef | -21.83 | 152.63 | 0.3 | 0.3 | 0.2 | 0.1 | 0.3 | Low |
| Great Barrier Reef | -21.82 | 152.63 | 0.3 | 0.3 | 0.2 | 0.1 | 0.3 | Low |
| Great Barrier Reef | -21.81 | 152.45 | 0.3 | 0.3 | 0.2 | 0.1 | 0.3 | Low |
| Great Barrier Reef | -21.80 | 152.48 | 0.3 | 0.3 | 0.2 | 0.1 | 0.3 | Low |
| Great Barrier Reef | -21.80 | 152.39 | 0.3 | 0.3 | 0.3 | 0.1 | 0.4 | Medial |
| Western Indian Ocean | -21.80 | 35.51 | 0.1 | 0.3 | 0.2 | 0.4 | 0.5 | Medial |
| Great Barrier Reef | -21.80 | 152.38 | 0.3 | 0.3 | 0.3 | 0.1 | 0.4 | Medial |
| Great Barrier Reef | -21.79 | 152.59 | 0.2 | 0.3 | 0.2 | 0.1 | 0.3 | Low |
| Great Barrier Reef | -21.79 | 152.59 | 0.2 | 0.3 | 0.2 | 0.1 | 0.3 | Low |
| Great Barrier Reef | -21.79 | 152.45 | 0.3 | 0.3 | 0.2 | 0.1 | 0.3 | Low |
| Great Barrier Reef | -21.76 | 152.42 | 0.3 | 0.3 | 0.2 | 0.1 | 0.3 | Low |
| Great Barrier Reef | -21.74 | 152.43 | 0.3 | 0.3 | 0.2 | 0.1 | 0.3 | Low |
| Great Barrier Reef | -21.73 | 150.32 | 0.5 | 0.1 | 0.4 | 0.5 | 0.7 | High |
| Great Barrier Reef | -21.72 | 152.56 | 0.2 | 0.3 | 0.2 | 0.1 | 0.3 | Low |
| Great Barrier Reef | -21.71 | 152.45 | 0.3 | 0.3 | 0.2 | 0.1 | 0.3 | Low |
| Western Australia | -21.68 | 114.93 | 0.6 | 0.3 | 0.5 | 0.7 | 0.9 | Severe |
| Great Barrier Reef | -21.68 | 152.44 | 0.2 | 0.3 | 0.2 | 0.1 | 0.3 | Low |
| Great Barrier Reef | -21.67 | 152.40 | 0.3 | 0.3 | 0.2 | 0.1 | 0.3 | Low |
| Great Barrier Reef | -21.67 | 152.54 | 0.2 | 0.3 | 0.2 | 0.1 | 0.3 | Low |
| Great Barrier Reef | -21.67 | 152.54 | 0.2 | 0.3 | 0.2 | 0.1 | 0.3 | Low |
| Great Barrier Reef | -21.67 | 152.45 | 0.2 | 0.3 | 0.2 | 0.1 | 0.3 | Low |
| Great Barrier Reef | -21.66 | 150.32 | 0.5 | 0.1 | 0.3 | 0.5 | 0.7 | Medial |
| Great Barrier Reef | -21.65 | 150.22 | 0.5 | 0.1 | 0.3 | 0.5 | 0.7 | Medial |
| Great Barrier Reef | -21.65 | 152.48 | 0.2 | 0.3 | 0.2 | 0.1 | 0.3 | Low |
| Great Barrier Reef | -21.64 | 152.27 | 0.2 | 0.3 | 0.2 | 0.2 | 0.4 | Low |
| Great Barrier Reef | -21.63 | 152.40 | 0.3 | 0.3 | 0.2 | 0.1 | 0.3 | Low |
| Great Barrier Reef | -21.61 | 152.21 | 0.2 | 0.3 | 0.2 | 0.2 | 0.4 | Low |
| Great Barrier Reef | -21.61 | 152.47 | 0.2 | 0.3 | 0.2 | 0.1 | 0.3 | Low |
| Great Barrier Reef | -21.61 | 152.37 | 0.2 | 0.3 | 0.2 | 0.1 | 0.3 | Low |
| Great Barrier Reef | -21.61 | 149.78 | 0.6 | 0.1 | 0.4 | 0.6 | 0.7 | High |
| Great Barrier Reef | -21.61 | 152.19 | 0.2 | 0.3 | 0.2 | 0.2 | 0.4 | Low |
| Great Barrier Reef | -21.60 | 152.08 | 0.2 | 0.3 | 0.2 | 0.2 | 0.4 | Medial |
| Great Barrier Reef | -21.58 | 151.93 | 0.2 | 0.1 | 0.2 | 0.3 | 0.4 | Medial |
| Great Barrier Reef | -21.58 | 152.53 | 0.2 | 0.3 | 0.2 | 0.1 | 0.3 | Low |
| Great Barrier Reef | -21.58 | 152.54 | 0.2 | 0.3 | 0.2 | 0.1 | 0.3 | Low |
| Great Barrier Reef | -21.57 | 152.01 | 0.2 | 0.3 | 0.2 | 0.2 | 0.3 | Low |
| Great Barrier Reef | -21.56 | 152.54 | 0.2 | 0.3 | 0.2 | 0.1 | 0.3 | Low |
| Great Barrier Reef | -21.55 | 152.54 | 0.2 | 0.3 | 0.2 | 0.1 | 0.3 | Low |
| Great Barrier Reef | -21.53 | 151.78 | 0.2 | 0.1 | 0.2 | 0.3 | 0.5 | Medial |
| Great Barrier Reef | -21.53 | 151.92 | 0.2 | 0.1 | 0.2 | 0.3 | 0.4 | Medial |
| Great Barrier Reef | -21.53 | 150.15 | 0.5 | 0.1 | 0.3 | 0.5 | 0.7 | High |
| Great Barrier Reef | -21.52 | 150.26 | 0.5 | 0.1 | 0.3 | 0.5 | 0.7 | Medial |
| Western Indian Ocean | -21.52 | 35.48 | 0.2 | 0.3 | 0.2 | 0.9 | 0.9 | Severe |
| Great Barrier Reef | -21.52 | 151.81 | 0.2 | 0.2 | 0.2 | 0.3 | 0.4 | Medial |
| Western Indian Ocean | -21.50 | 35.50 | 0.2 | 0.3 | 0.2 | 0.9 | 0.9 | Severe |
| Great Barrier Reef | -21.50 | 149.91 | 0.5 | 0.1 | 0.3 | 0.5 | 0.7 | High |
| Western Indian Ocean | -21.49 | 35.51 | 0.1 | 0.3 | 0.1 | 0.8 | 0.8 | Severe |
| Great Barrier Reef | -21.49 | 149.88 | 0.5 | 0.1 | 0.4 | 0.5 | 0.7 | High |
| Great Barrier Reef | -21.49 | 152.56 | 0.2 | 0.3 | 0.2 | 0.1 | 0.3 | Low |
| Great Barrier Reef | -21.49 | 152.56 | 0.2 | 0.3 | 0.2 | 0.1 | 0.3 | Low |
| Great Barrier Reef | -21.48 | 149.26 | 0.7 | 0.1 | 0.5 | 0.7 | 0.9 | Severe |
| Great Barrier Reef | -21.48 | 149.91 | 0.5 | 0.1 | 0.3 | 0.5 | 0.7 | High |
| Great Barrier Reef | -21.47 | 149.88 | 0.6 | 0.1 | 0.4 | 0.5 | 0.7 | High |
| Great Barrier Reef | -21.47 | 152.52 | 0.3 | 0.3 | 0.2 | 0.1 | 0.3 | Low |
| Great Barrier Reef | -21.46 | 152.52 | 0.3 | 0.3 | 0.2 | 0.1 | 0.3 | Low |
| Great Barrier Reef | -21.46 | 149.87 | 0.6 | 0.1 | 0.4 | 0.5 | 0.7 | High |
| Great Barrier Reef | -21.45 | 151.67 | 0.3 | 0.1 | 0.2 | 0.3 | 0.5 | Medial |
| Western Australia | -21.45 | 115.37 | 0.6 | 0.2 | 0.5 | 0.8 | 0.9 | Severe |
| Great Barrier Reef | -21.45 | 149.71 | 0.5 | 0.1 | 0.3 | 0.6 | 0.7 | High |
| Great Barrier Reef | -21.45 | 151.59 | 0.2 | 0.1 | 0.2 | 0.3 | 0.5 | Medial |
| Great Barrier Reef | -21.44 | 149.83 | 0.5 | 0.1 | 0.4 | 0.5 | 0.7 | High |
| Great Barrier Reef | -21.43 | 149.82 | 0.5 | 0.1 | 0.4 | 0.5 | 0.7 | High |
| Great Barrier Reef | -21.43 | 151.42 | 0.3 | 0.1 | 0.2 | 0.3 | 0.4 | Medial |
| Great Barrier Reef | -21.43 | 151.42 | 0.3 | 0.1 | 0.2 | 0.3 | 0.4 | Medial |
| Great Barrier Reef | -21.42 | 151.65 | 0.2 | 0.1 | 0.2 | 0.3 | 0.5 | Medial |
| Great Barrier Reef | -21.42 | 152.49 | 0.3 | 0.3 | 0.3 | 0.1 | 0.3 | Low |
| Great Barrier Reef | -21.40 | 151.42 | 0.3 | 0.1 | 0.2 | 0.3 | 0.4 | Medial |
| Great Barrier Reef | -21.40 | 151.42 | 0.3 | 0.1 | 0.2 | 0.3 | 0.4 | Medial |
| Great Barrier Reef | -21.40 | 152.51 | 0.3 | 0.3 | 0.2 | 0.1 | 0.3 | Low |
| Great Barrier Reef | -21.40 | 152.51 | 0.3 | 0.3 | 0.2 | 0.1 | 0.3 | Low |
| Great Barrier Reef | -21.40 | 151.33 | 0.3 | 0.1 | 0.2 | 0.2 | 0.4 | Medial |
| Great Barrier Reef | -21.39 | 151.33 | 0.3 | 0.1 | 0.2 | 0.2 | 0.4 | Medial |
| Great Barrier Reef | -21.39 | 151.28 | 0.3 | 0.1 | 0.2 | 0.2 | 0.4 | Medial |
| Great Barrier Reef | -21.39 | 151.28 | 0.3 | 0.1 | 0.2 | 0.2 | 0.4 | Medial |
| Great Barrier Reef | -21.39 | 151.42 | 0.2 | 0.1 | 0.2 | 0.3 | 0.4 | Medial |
| Great Barrier Reef | -21.39 | 151.42 | 0.2 | 0.1 | 0.2 | 0.3 | 0.4 | Medial |
| Great Barrier Reef | -21.39 | 152.50 | 0.3 | 0.3 | 0.2 | 0.1 | 0.3 | Low |
| Great Barrier Reef | -21.38 | 152.53 | 0.3 | 0.3 | 0.2 | 0.1 | 0.3 | Low |
| Great Barrier Reef | -21.38 | 151.25 | 0.3 | 0.1 | 0.2 | 0.2 | 0.4 | Medial |
| Great Barrier Reef | -21.37 | 151.37 | 0.2 | 0.1 | 0.2 | 0.2 | 0.4 | Medial |
| Great Barrier Reef | -21.37 | 151.37 | 0.2 | 0.1 | 0.2 | 0.2 | 0.4 | Medial |
| Great Barrier Reef | -21.37 | 149.83 | 0.5 | 0.1 | 0.3 | 0.5 | 0.7 | High |
| Great Barrier Reef | -21.36 | 152.48 | 0.3 | 0.3 | 0.2 | 0.1 | 0.3 | Low |
| Great Barrier Reef | -21.36 | 152.52 | 0.3 | 0.3 | 0.2 | 0.1 | 0.3 | Low |
| Great Barrier Reef | -21.36 | 152.48 | 0.3 | 0.3 | 0.2 | 0.1 | 0.3 | Low |
| Great Barrier Reef | -21.36 | 152.52 | 0.3 | 0.3 | 0.3 | 0.1 | 0.3 | Low |
| Great Barrier Reef | -21.35 | 151.48 | 0.2 | 0.1 | 0.2 | 0.3 | 0.5 | Medial |
| Great Barrier Reef | -21.35 | 152.25 | 0.2 | 0.3 | 0.2 | 0.1 | 0.3 | Low |
| Great Barrier Reef | -21.34 | 151.28 | 0.3 | 0.1 | 0.2 | 0.2 | 0.4 | Medial |
| Great Barrier Reef | -21.34 | 151.28 | 0.3 | 0.1 | 0.2 | 0.2 | 0.4 | Medial |
| Great Barrier Reef | -21.34 | 151.83 | 0.2 | 0.3 | 0.2 | 0.3 | 0.4 | Medial |
| Great Barrier Reef | -21.33 | 149.69 | 0.6 | 0.1 | 0.4 | 0.5 | 0.7 | High |
| Western Indian Ocean | -21.33 | 55.46 | 0.3 | 0.4 | 0.3 | 0.1 | 0.3 | Low |
| Great Barrier Reef | -21.32 | 151.17 | 0.3 | 0.1 | 0.3 | 0.2 | 0.4 | Medial |
| Great Barrier Reef | -21.32 | 151.17 | 0.3 | 0.1 | 0.3 | 0.2 | 0.4 | Medial |
| Great Barrier Reef | -21.32 | 151.12 | 0.4 | 0.1 | 0.3 | 0.3 | 0.5 | Medial |
| Great Barrier Reef | -21.32 | 152.49 | 0.3 | 0.3 | 0.2 | 0.1 | 0.3 | Low |
| Great Barrier Reef | -21.32 | 152.49 | 0.3 | 0.3 | 0.2 | 0.1 | 0.3 | Low |
| Great Barrier Reef | -21.26 | 151.13 | 0.3 | 0.1 | 0.2 | 0.2 | 0.4 | Medial |
| Great Barrier Reef | -21.24 | 151.85 | 0.2 | 0.3 | 0.2 | 0.2 | 0.4 | Medial |
| Great Barrier Reef | -21.24 | 151.85 | 0.2 | 0.3 | 0.2 | 0.2 | 0.4 | Medial |
| Great Barrier Reef | -21.23 | 152.50 | 0.2 | 0.3 | 0.2 | 0.1 | 0.3 | Low |
| Great Barrier Reef | -21.23 | 152.50 | 0.2 | 0.3 | 0.2 | 0.1 | 0.3 | Low |
| Great Barrier Reef | -21.23 | 151.99 | 0.3 | 0.3 | 0.2 | 0.2 | 0.4 | Medial |
| Great Barrier Reef | -21.22 | 151.99 | 0.3 | 0.3 | 0.2 | 0.2 | 0.4 | Medial |
| Great Barrier Reef | -21.22 | 152.01 | 0.2 | 0.3 | 0.2 | 0.2 | 0.4 | Low |
| Western Indian Ocean | -21.22 | 55.22 | 0.1 | 0.4 | 0.1 | 0.0 | 0.2 | Low |
| Great Barrier Reef | -21.22 | 152.01 | 0.2 | 0.3 | 0.2 | 0.2 | 0.4 | Low |
| Great Barrier Reef | -21.21 | 151.01 | 0.3 | 0.1 | 0.3 | 0.2 | 0.5 | Medial |
| Great Barrier Reef | -21.21 | 151.01 | 0.3 | 0.1 | 0.3 | 0.2 | 0.5 | Medial |
| Great Barrier Reef | -21.21 | 151.99 | 0.3 | 0.3 | 0.2 | 0.2 | 0.4 | Medial |
| Great Barrier Reef | -21.21 | 151.99 | 0.3 | 0.3 | 0.2 | 0.2 | 0.4 | Medial |
| Great Barrier Reef | -21.21 | 152.50 | 0.2 | 0.3 | 0.2 | 0.1 | 0.3 | Low |
| Great Barrier Reef | -21.20 | 152.52 | 0.2 | 0.3 | 0.2 | 0.1 | 0.3 | Low |
| Great Barrier Reef | -21.20 | 151.83 | 0.2 | 0.3 | 0.2 | 0.2 | 0.4 | Medial |
| Great Barrier Reef | -21.20 | 152.52 | 0.2 | 0.3 | 0.2 | 0.1 | 0.3 | Low |
| Great Barrier Reef | -21.20 | 152.10 | 0.3 | 0.3 | 0.2 | 0.1 | 0.3 | Low |
| Polynesia | -21.20 | -159.78 | 0.5 | 0.6 | 0.4 | 0.0 | 0.4 | Medial |
| Great Barrier Reef | -21.20 | 152.10 | 0.3 | 0.3 | 0.2 | 0.1 | 0.3 | Low |
| Western Indian Ocean | -21.20 | 43.19 | 0.3 | 0.3 | 0.4 | 0.3 | 0.5 | Medial |
| Great Barrier Reef | -21.19 | 150.94 | 0.3 | 0.1 | 0.2 | 0.3 | 0.5 | Medial |
| Great Barrier Reef | -21.19 | 150.87 | 0.3 | 0.1 | 0.3 | 0.4 | 0.5 | Medial |
| Great Barrier Reef | -21.19 | 151.82 | 0.2 | 0.3 | 0.2 | 0.2 | 0.4 | Medial |
| Great Barrier Reef | -21.18 | 151.82 | 0.2 | 0.3 | 0.2 | 0.2 | 0.4 | Medial |
| Great Barrier Reef | -21.18 | 152.52 | 0.3 | 0.3 | 0.2 | 0.1 | 0.3 | Low |
| Great Barrier Reef | -21.18 | 152.52 | 0.3 | 0.3 | 0.2 | 0.1 | 0.3 | Low |
| Great Barrier Reef | -21.17 | 151.04 | 0.3 | 0.1 | 0.3 | 0.2 | 0.4 | Medial |
| Great Barrier Reef | -21.17 | 151.04 | 0.3 | 0.1 | 0.3 | 0.2 | 0.4 | Medial |
| Western Indian Ocean | -21.17 | 55.28 | 0.1 | 0.4 | 0.1 | 0.0 | 0.1 | Low |
| Western Indian Ocean | -21.17 | 55.28 | 0.1 | 0.4 | 0.1 | 0.0 | 0.1 | Low |
| Polynesia | -21.17 | -175.17 | 0.6 | 0.4 | 0.5 | 0.3 | 0.6 | Medial |
| Great Barrier Reef | -21.17 | 150.66 | 0.4 | 0.1 | 0.3 | 0.5 | 0.7 | High |
| Great Barrier Reef | -21.17 | 151.80 | 0.2 | 0.3 | 0.2 | 0.2 | 0.4 | Low |
| Great Barrier Reef | -21.16 | 151.80 | 0.2 | 0.3 | 0.2 | 0.2 | 0.4 | Low |
| Great Barrier Reef | -21.16 | 150.93 | 0.3 | 0.1 | 0.3 | 0.3 | 0.5 | Medial |
| Great Barrier Reef | -21.16 | 152.15 | 0.2 | 0.3 | 0.2 | 0.1 | 0.3 | Low |
| Western Indian Ocean | -21.16 | 55.28 | 0.1 | 0.4 | 0.1 | 0.0 | 0.1 | Low |
| Great Barrier Reef | -21.16 | 151.70 | 0.2 | 0.3 | 0.2 | 0.3 | 0.4 | Medial |
| Great Barrier Reef | -21.16 | 152.31 | 0.3 | 0.3 | 0.3 | 0.1 | 0.4 | Low |
| Great Barrier Reef | -21.16 | 150.85 | 0.3 | 0.1 | 0.3 | 0.5 | 0.6 | Medial |
| Great Barrier Reef | -21.16 | 151.78 | 0.2 | 0.3 | 0.2 | 0.3 | 0.4 | Medial |
| Western Indian Ocean | -21.15 | 55.27 | 0.1 | 0.4 | 0.1 | 0.0 | 0.1 | Low |
| Great Barrier Reef | -21.15 | 151.78 | 0.2 | 0.3 | 0.2 | 0.3 | 0.4 | Medial |
| Great Barrier Reef | -21.15 | 151.76 | 0.2 | 0.3 | 0.2 | 0.3 | 0.4 | Medial |
| Great Barrier Reef | -21.14 | 152.50 | 0.3 | 0.3 | 0.2 | 0.1 | 0.3 | Low |
| Great Barrier Reef | -21.14 | 152.19 | 0.2 | 0.3 | 0.2 | 0.1 | 0.3 | Low |
| Great Barrier Reef | -21.14 | 152.19 | 0.2 | 0.3 | 0.2 | 0.1 | 0.3 | Low |
| Great Barrier Reef | -21.14 | 150.76 | 0.3 | 0.1 | 0.3 | 0.5 | 0.7 | Medial |
| Great Barrier Reef | -21.14 | 150.79 | 0.3 | 0.1 | 0.3 | 0.5 | 0.6 | Medial |
| Great Barrier Reef | -21.14 | 152.49 | 0.3 | 0.3 | 0.3 | 0.1 | 0.3 | Low |
| Great Barrier Reef | -21.13 | 152.49 | 0.3 | 0.3 | 0.3 | 0.1 | 0.3 | Low |
| Great Barrier Reef | -21.13 | 152.47 | 0.3 | 0.3 | 0.3 | 0.1 | 0.3 | Low |
| Great Barrier Reef | -21.13 | 151.08 | 0.3 | 0.1 | 0.2 | 0.2 | 0.4 | Medial |
| Great Barrier Reef | -21.13 | 150.48 | 0.4 | 0.1 | 0.3 | 0.4 | 0.6 | Medial |
| Great Barrier Reef | -21.12 | 150.48 | 0.4 | 0.1 | 0.3 | 0.4 | 0.6 | Medial |
| Great Barrier Reef | -21.12 | 152.39 | 0.2 | 0.3 | 0.2 | 0.1 | 0.3 | Low |
| Great Barrier Reef | -21.12 | 151.77 | 0.2 | 0.3 | 0.2 | 0.2 | 0.4 | Medial |
| Great Barrier Reef | -21.12 | 151.78 | 0.2 | 0.3 | 0.2 | 0.2 | 0.4 | Medial |
| Great Barrier Reef | -21.12 | 152.32 | 0.3 | 0.3 | 0.3 | 0.1 | 0.3 | Low |
| Western Indian Ocean | -21.12 | 55.83 | 0.3 | 0.4 | 0.3 | 0.0 | 0.3 | Low |
| Western Indian Ocean | -21.12 | 55.50 | 0.2 | 0.4 | 0.3 | 0.0 | 0.3 | Low |
| Great Barrier Reef | -21.12 | 151.76 | 0.2 | 0.3 | 0.2 | 0.2 | 0.4 | Medial |
| Great Barrier Reef | -21.11 | 151.76 | 0.2 | 0.3 | 0.2 | 0.2 | 0.4 | Medial |
| Great Barrier Reef | -21.11 | 151.74 | 0.2 | 0.3 | 0.2 | 0.3 | 0.4 | Medial |
| Great Barrier Reef | -21.11 | 151.74 | 0.2 | 0.3 | 0.2 | 0.3 | 0.4 | Medial |
| Great Barrier Reef | -21.11 | 152.08 | 0.3 | 0.3 | 0.2 | 0.1 | 0.3 | Low |
| Great Barrier Reef | -21.11 | 152.31 | 0.3 | 0.3 | 0.2 | 0.1 | 0.3 | Low |
| Great Barrier Reef | -21.10 | 152.31 | 0.3 | 0.3 | 0.2 | 0.1 | 0.3 | Low |
| Great Barrier Reef | -21.10 | 152.16 | 0.2 | 0.3 | 0.2 | 0.1 | 0.3 | Low |
| Great Barrier Reef | -21.10 | 152.20 | 0.3 | 0.3 | 0.2 | 0.1 | 0.3 | Low |
| Great Barrier Reef | -21.10 | 152.35 | 0.3 | 0.3 | 0.3 | 0.1 | 0.3 | Low |
| Great Barrier Reef | -21.10 | 152.20 | 0.3 | 0.3 | 0.2 | 0.1 | 0.3 | Low |
| Great Barrier Reef | -21.10 | 152.35 | 0.3 | 0.3 | 0.3 | 0.1 | 0.3 | Low |
| Great Barrier Reef | -21.10 | 151.72 | 0.2 | 0.3 | 0.2 | 0.3 | 0.4 | Medial |
| Great Barrier Reef | -21.10 | 152.26 | 0.3 | 0.3 | 0.2 | 0.1 | 0.3 | Low |
| Great Barrier Reef | -21.10 | 152.49 | 0.3 | 0.3 | 0.3 | 0.1 | 0.3 | Low |
| Western Indian Ocean | -21.10 | 55.24 | 0.2 | 0.4 | 0.2 | 0.0 | 0.2 | Low |
| Great Barrier Reef | -21.10 | 151.72 | 0.2 | 0.3 | 0.2 | 0.3 | 0.4 | Medial |
| Great Barrier Reef | -21.10 | 152.17 | 0.2 | 0.3 | 0.2 | 0.1 | 0.3 | Low |
| Great Barrier Reef | -21.09 | 152.14 | 0.2 | 0.3 | 0.2 | 0.1 | 0.3 | Low |
| Great Barrier Reef | -21.09 | 152.24 | 0.2 | 0.3 | 0.2 | 0.1 | 0.3 | Low |
| Great Barrier Reef | -21.09 | 152.50 | 0.3 | 0.3 | 0.2 | 0.1 | 0.3 | Low |
| Great Barrier Reef | -21.09 | 152.28 | 0.2 | 0.3 | 0.2 | 0.1 | 0.3 | Low |
| Great Barrier Reef | -21.09 | 151.69 | 0.2 | 0.3 | 0.2 | 0.3 | 0.4 | Medial |
| Great Barrier Reef | -21.09 | 152.24 | 0.2 | 0.3 | 0.2 | 0.1 | 0.3 | Low |
| Great Barrier Reef | -21.09 | 152.50 | 0.3 | 0.3 | 0.2 | 0.1 | 0.3 | Low |
| Great Barrier Reef | -21.08 | 151.65 | 0.2 | 0.3 | 0.2 | 0.3 | 0.4 | Medial |
| Great Barrier Reef | -21.08 | 150.45 | 0.4 | 0.1 | 0.3 | 0.4 | 0.6 | Medial |
| Great Barrier Reef | -21.08 | 152.28 | 0.2 | 0.3 | 0.2 | 0.1 | 0.3 | Low |
| Great Barrier Reef | -21.08 | 151.69 | 0.2 | 0.3 | 0.2 | 0.3 | 0.4 | Medial |
| Great Barrier Reef | -21.08 | 151.65 | 0.2 | 0.3 | 0.2 | 0.3 | 0.4 | Medial |
| Great Barrier Reef | -21.08 | 150.45 | 0.4 | 0.1 | 0.3 | 0.4 | 0.6 | Medial |
| Great Barrier Reef | -21.08 | 152.21 | 0.2 | 0.3 | 0.2 | 0.1 | 0.3 | Low |
| Great Barrier Reef | -21.07 | 151.67 | 0.2 | 0.3 | 0.2 | 0.3 | 0.4 | Medial |
| Great Barrier Reef | -21.07 | 151.62 | 0.2 | 0.3 | 0.2 | 0.3 | 0.4 | Medial |
| Great Barrier Reef | -21.07 | 151.67 | 0.2 | 0.3 | 0.2 | 0.3 | 0.4 | Medial |
| Great Barrier Reef | -21.07 | 150.26 | 0.5 | 0.1 | 0.4 | 0.2 | 0.5 | Medial |
| Great Barrier Reef | -21.07 | 152.23 | 0.2 | 0.3 | 0.2 | 0.1 | 0.3 | Low |
| Great Barrier Reef | -21.07 | 152.19 | 0.2 | 0.3 | 0.2 | 0.1 | 0.3 | Low |
| Great Barrier Reef | -21.07 | 152.46 | 0.3 | 0.3 | 0.3 | 0.1 | 0.3 | Low |
| Great Barrier Reef | -21.07 | 152.23 | 0.2 | 0.3 | 0.2 | 0.1 | 0.3 | Low |
| Great Barrier Reef | -21.06 | 152.47 | 0.3 | 0.3 | 0.3 | 0.1 | 0.3 | Low |
| Great Barrier Reef | -21.05 | 150.51 | 0.5 | 0.1 | 0.3 | 0.4 | 0.6 | Medial |
| Great Barrier Reef | -21.05 | 152.21 | 0.2 | 0.3 | 0.2 | 0.1 | 0.3 | Low |
| Great Barrier Reef | -21.05 | 150.51 | 0.5 | 0.1 | 0.3 | 0.4 | 0.6 | Medial |
| Great Barrier Reef | -21.05 | 152.21 | 0.2 | 0.3 | 0.2 | 0.1 | 0.3 | Low |
| Great Barrier Reef | -21.05 | 151.58 | 0.2 | 0.3 | 0.2 | 0.3 | 0.4 | Medial |
| Great Barrier Reef | -21.05 | 151.58 | 0.2 | 0.3 | 0.2 | 0.3 | 0.4 | Medial |
| Great Barrier Reef | -21.04 | 151.56 | 0.2 | 0.3 | 0.2 | 0.3 | 0.5 | Medial |
| Great Barrier Reef | -21.04 | 151.48 | 0.2 | 0.3 | 0.2 | 0.4 | 0.5 | Medial |
| Great Barrier Reef | -21.04 | 151.56 | 0.2 | 0.3 | 0.2 | 0.3 | 0.5 | Medial |
| Great Barrier Reef | -21.04 | 151.57 | 0.2 | 0.3 | 0.2 | 0.3 | 0.4 | Medial |
| Great Barrier Reef | -21.03 | 149.55 | 0.5 | 0.1 | 0.3 | 0.5 | 0.7 | High |
| Great Barrier Reef | -21.03 | 151.52 | 0.2 | 0.3 | 0.2 | 0.4 | 0.5 | Medial |
| Great Barrier Reef | -21.03 | 150.92 | 0.2 | 0.1 | 0.2 | 0.2 | 0.4 | Medial |
| Great Barrier Reef | -21.03 | 150.08 | 0.4 | 0.1 | 0.3 | 0.5 | 0.6 | Medial |
| Great Barrier Reef | -21.02 | 149.90 | 0.4 | 0.1 | 0.3 | 0.5 | 0.6 | Medial |
| Great Barrier Reef | -21.02 | 149.90 | 0.4 | 0.1 | 0.3 | 0.5 | 0.6 | Medial |
| Great Barrier Reef | -21.01 | 150.52 | 0.3 | 0.1 | 0.2 | 0.5 | 0.6 | Medial |
| Great Barrier Reef | -21.01 | 151.45 | 0.2 | 0.3 | 0.2 | 0.4 | 0.5 | Medial |
| Great Barrier Reef | -21.00 | 152.47 | 0.3 | 0.3 | 0.3 | 0.1 | 0.4 | Medial |
| Great Barrier Reef | -20.99 | 149.78 | 0.4 | 0.1 | 0.3 | 0.5 | 0.7 | Medial |
| Great Barrier Reef | -20.99 | 152.39 | 0.3 | 0.3 | 0.3 | 0.1 | 0.3 | Low |
| Great Barrier Reef | -20.98 | 149.78 | 0.4 | 0.1 | 0.3 | 0.5 | 0.7 | Medial |
| Great Barrier Reef | -20.98 | 150.53 | 0.4 | 0.1 | 0.3 | 0.5 | 0.7 | High |
| Great Barrier Reef | -20.98 | 150.53 | 0.4 | 0.1 | 0.3 | 0.5 | 0.7 | High |
| Great Barrier Reef | -20.97 | 151.25 | 0.1 | 0.2 | 0.2 | 0.3 | 0.4 | Medial |
| Great Barrier Reef | -20.97 | 151.40 | 0.2 | 0.3 | 0.2 | 0.4 | 0.5 | Medial |
| Great Barrier Reef | -20.97 | 151.40 | 0.2 | 0.3 | 0.2 | 0.4 | 0.5 | Medial |
| Great Barrier Reef | -20.96 | 151.36 | 0.1 | 0.3 | 0.2 | 0.4 | 0.5 | Medial |
| Great Barrier Reef | -20.96 | 150.08 | 0.4 | 0.1 | 0.3 | 0.4 | 0.6 | Medial |
| Great Barrier Reef | -20.96 | 151.36 | 0.2 | 0.3 | 0.2 | 0.4 | 0.5 | Medial |
| Great Barrier Reef | -20.96 | 150.08 | 0.4 | 0.1 | 0.3 | 0.4 | 0.6 | Medial |
| Great Barrier Reef | -20.95 | 149.74 | 0.5 | 0.1 | 0.3 | 0.5 | 0.7 | High |
| Great Barrier Reef | -20.95 | 150.66 | 0.4 | 0.1 | 0.3 | 0.5 | 0.7 | High |
| Great Barrier Reef | -20.95 | 149.74 | 0.5 | 0.1 | 0.3 | 0.5 | 0.7 | High |
| Great Barrier Reef | -20.95 | 150.66 | 0.4 | 0.1 | 0.3 | 0.5 | 0.7 | High |
| Great Barrier Reef | -20.94 | 151.32 | 0.2 | 0.3 | 0.2 | 0.4 | 0.5 | Medial |
| Great Barrier Reef | -20.94 | 151.32 | 0.2 | 0.3 | 0.2 | 0.4 | 0.5 | Medial |
| Great Barrier Reef | -20.94 | 150.54 | 0.4 | 0.1 | 0.3 | 0.6 | 0.7 | High |
| Great Barrier Reef | -20.93 | 149.45 | 0.6 | 0.1 | 0.4 | 0.5 | 0.7 | High |
| Great Barrier Reef | -20.93 | 149.43 | 0.6 | 0.1 | 0.4 | 0.5 | 0.7 | High |
| Great Barrier Reef | -20.93 | 149.43 | 0.6 | 0.1 | 0.4 | 0.5 | 0.7 | High |
| Great Barrier Reef | -20.93 | 149.47 | 0.5 | 0.1 | 0.4 | 0.5 | 0.7 | High |
| Great Barrier Reef | -20.93 | 151.24 | 0.1 | 0.2 | 0.1 | 0.3 | 0.4 | Medial |
| Great Barrier Reef | -20.92 | 149.41 | 0.5 | 0.1 | 0.4 | 0.6 | 0.7 | High |
| Great Barrier Reef | -20.91 | 150.03 | 0.4 | 0.1 | 0.3 | 0.4 | 0.6 | Medial |
| Great Barrier Reef | -20.91 | 150.03 | 0.4 | 0.1 | 0.3 | 0.4 | 0.6 | Medial |
| Great Barrier Reef | -20.91 | 149.39 | 0.5 | 0.1 | 0.4 | 0.5 | 0.7 | High |
| Great Barrier Reef | -20.91 | 149.44 | 0.5 | 0.1 | 0.4 | 0.5 | 0.7 | High |
| Great Barrier Reef | -20.90 | 150.62 | 0.4 | 0.1 | 0.3 | 0.6 | 0.7 | High |
| Great Barrier Reef | -20.89 | 151.30 | 0.2 | 0.2 | 0.2 | 0.3 | 0.5 | Medial |
| Great Barrier Reef | -20.87 | 151.25 | 0.2 | 0.3 | 0.2 | 0.3 | 0.4 | Medial |
| Great Barrier Reef | -20.87 | 151.25 | 0.2 | 0.3 | 0.2 | 0.3 | 0.4 | Medial |
| Great Barrier Reef | -20.87 | 149.60 | 0.6 | 0.1 | 0.4 | 0.5 | 0.7 | High |
| Great Barrier Reef | -20.86 | 149.60 | 0.6 | 0.1 | 0.4 | 0.5 | 0.7 | High |
| Great Barrier Reef | -20.86 | 150.38 | 0.4 | 0.1 | 0.3 | 0.4 | 0.6 | Medial |
| Great Barrier Reef | -20.86 | 151.30 | 0.2 | 0.3 | 0.2 | 0.4 | 0.5 | Medial |
| Great Barrier Reef | -20.84 | 151.23 | 0.2 | 0.3 | 0.2 | 0.3 | 0.4 | Medial |
| Great Barrier Reef | -20.82 | 150.38 | 0.5 | 0.1 | 0.4 | 0.5 | 0.7 | High |
| Great Barrier Reef | -20.82 | 149.27 | 0.6 | 0.1 | 0.4 | 0.6 | 0.8 | High |
| Great Barrier Reef | -20.82 | 149.27 | 0.6 | 0.1 | 0.4 | 0.6 | 0.8 | High |
| Great Barrier Reef | -20.81 | 149.27 | 0.6 | 0.1 | 0.4 | 0.6 | 0.8 | High |
| Great Barrier Reef | -20.81 | 151.09 | 0.2 | 0.2 | 0.2 | 0.2 | 0.4 | Medial |
| Great Barrier Reef | -20.81 | 151.07 | 0.2 | 0.2 | 0.2 | 0.2 | 0.4 | Medial |
| Great Barrier Reef | -20.80 | 151.16 | 0.2 | 0.2 | 0.2 | 0.3 | 0.4 | Medial |
| Great Barrier Reef | -20.80 | 151.16 | 0.2 | 0.2 | 0.2 | 0.3 | 0.4 | Medial |
| Great Barrier Reef | -20.80 | 149.28 | 0.6 | 0.1 | 0.4 | 0.6 | 0.8 | High |
| Great Barrier Reef | -20.80 | 149.28 | 0.6 | 0.1 | 0.4 | 0.6 | 0.8 | High |
| Great Barrier Reef | -20.80 | 150.45 | 0.4 | 0.1 | 0.3 | 0.6 | 0.7 | High |
| Great Barrier Reef | -20.78 | 150.48 | 0.4 | 0.1 | 0.3 | 0.6 | 0.7 | High |
| Great Barrier Reef | -20.78 | 149.39 | 0.5 | 0.1 | 0.3 | 0.5 | 0.7 | High |
| Great Barrier Reef | -20.77 | 151.04 | 0.2 | 0.2 | 0.2 | 0.2 | 0.4 | Medial |
| Great Barrier Reef | -20.77 | 149.43 | 0.5 | 0.1 | 0.4 | 0.5 | 0.7 | High |
| Great Barrier Reef | -20.77 | 150.96 | 0.2 | 0.2 | 0.2 | 0.2 | 0.4 | Medial |
| Great Barrier Reef | -20.77 | 149.61 | 0.5 | 0.1 | 0.4 | 0.5 | 0.7 | High |
| Great Barrier Reef | -20.76 | 149.28 | 0.6 | 0.1 | 0.4 | 0.6 | 0.8 | High |
| Great Barrier Reef | -20.75 | 149.16 | 0.6 | 0.1 | 0.4 | 0.6 | 0.8 | High |
| Great Barrier Reef | -20.75 | 151.02 | 0.2 | 0.2 | 0.2 | 0.2 | 0.4 | Medial |
| Great Barrier Reef | -20.74 | 149.48 | 0.5 | 0.1 | 0.4 | 0.5 | 0.7 | High |
| Great Barrier Reef | -20.74 | 149.15 | 0.5 | 0.1 | 0.4 | 0.6 | 0.8 | High |
| Great Barrier Reef | -20.73 | 150.89 | 0.2 | 0.2 | 0.2 | 0.2 | 0.4 | Medial |
| Great Barrier Reef | -20.72 | 151.05 | 0.2 | 0.2 | 0.2 | 0.2 | 0.4 | Medial |
| Great Barrier Reef | -20.72 | 150.47 | 0.3 | 0.1 | 0.3 | 0.6 | 0.7 | High |
| Great Barrier Reef | -20.72 | 150.47 | 0.3 | 0.1 | 0.3 | 0.6 | 0.7 | High |
| Great Barrier Reef | -20.72 | 151.05 | 0.2 | 0.2 | 0.2 | 0.2 | 0.4 | Medial |
| Great Barrier Reef | -20.72 | 150.47 | 0.4 | 0.1 | 0.3 | 0.6 | 0.7 | High |
| Great Barrier Reef | -20.72 | 165.22 | 0.4 | 0.3 | 0.4 | 0.1 | 0.5 | Medial |
| Great Barrier Reef | -20.71 | 150.82 | 0.2 | 0.2 | 0.2 | 0.2 | 0.4 | Medial |
| Great Barrier Reef | -20.71 | 149.14 | 0.5 | 0.1 | 0.4 | 0.6 | 0.8 | High |
| Great Barrier Reef | -20.70 | 150.18 | 0.4 | 0.1 | 0.3 | 0.2 | 0.5 | Medial |
| Great Barrier Reef | -20.70 | 149.21 | 0.5 | 0.1 | 0.4 | 0.6 | 0.8 | High |
| Great Barrier Reef | -20.70 | 149.20 | 0.5 | 0.1 | 0.4 | 0.6 | 0.8 | High |
| Great Barrier Reef | -20.70 | 150.83 | 0.3 | 0.2 | 0.3 | 0.2 | 0.4 | Medial |
| Great Barrier Reef | -20.69 | 149.14 | 0.6 | 0.1 | 0.4 | 0.6 | 0.8 | High |
| Great Barrier Reef | -20.68 | 149.15 | 0.6 | 0.1 | 0.4 | 0.6 | 0.8 | High |
| Great Barrier Reef | -20.68 | 150.45 | 0.3 | 0.2 | 0.3 | 0.6 | 0.7 | High |
| Great Barrier Reef | -20.68 | 149.10 | 0.5 | 0.1 | 0.4 | 0.6 | 0.8 | High |
| Great Barrier Reef | -20.68 | 150.23 | 0.4 | 0.1 | 0.3 | 0.3 | 0.6 | Medial |
| Great Barrier Reef | -20.68 | 150.23 | 0.4 | 0.1 | 0.3 | 0.3 | 0.6 | Medial |
| Great Barrier Reef | -20.67 | 149.14 | 0.6 | 0.1 | 0.4 | 0.6 | 0.8 | High |
| Great Barrier Reef | -20.67 | 149.16 | 0.6 | 0.1 | 0.4 | 0.6 | 0.8 | High |
| Great Barrier Reef | -20.67 | 150.88 | 0.2 | 0.2 | 0.2 | 0.2 | 0.4 | Medial |
| Great Barrier Reef | -20.66 | 150.29 | 0.4 | 0.1 | 0.3 | 0.4 | 0.6 | Medial |
| Great Barrier Reef | -20.66 | 150.29 | 0.4 | 0.1 | 0.3 | 0.4 | 0.6 | Medial |
| Great Barrier Reef | -20.66 | 149.15 | 0.6 | 0.1 | 0.4 | 0.6 | 0.8 | High |
| Great Barrier Reef | -20.66 | 149.15 | 0.6 | 0.1 | 0.4 | 0.6 | 0.8 | High |
| Great Barrier Reef | -20.65 | 150.46 | 0.3 | 0.2 | 0.2 | 0.6 | 0.7 | High |
| Great Barrier Reef | -20.65 | 150.46 | 0.3 | 0.2 | 0.2 | 0.6 | 0.7 | High |
| Great Barrier Reef | -20.65 | 149.08 | 0.5 | 0.1 | 0.4 | 0.7 | 0.8 | High |
| Great Barrier Reef | -20.64 | 149.05 | 0.5 | 0.1 | 0.4 | 0.7 | 0.8 | High |
| Western Australia | -20.63 | 116.65 | 0.7 | 0.2 | 0.6 | 0.8 | 0.9 | Severe |
| Great Barrier Reef | -20.63 | 150.80 | 0.2 | 0.2 | 0.2 | 0.2 | 0.4 | Medial |
| Great Barrier Reef | -20.62 | 150.85 | 0.2 | 0.2 | 0.2 | 0.2 | 0.4 | Medial |
| Great Barrier Reef | -20.62 | 149.07 | 0.5 | 0.1 | 0.4 | 0.7 | 0.8 | High |
| Western Australia | -20.61 | 116.76 | 0.7 | 0.2 | 0.6 | 0.8 | 0.9 | Severe |
| Western Australia | -20.60 | 116.76 | 0.7 | 0.2 | 0.6 | 0.8 | 0.9 | Severe |
| Great Barrier Reef | -20.60 | 150.38 | 0.3 | 0.2 | 0.2 | 0.6 | 0.7 | High |
| Western Australia | -20.60 | 116.76 | 0.7 | 0.2 | 0.6 | 0.8 | 0.9 | Severe |
| Great Barrier Reef | -20.59 | 149.11 | 0.6 | 0.1 | 0.4 | 0.6 | 0.8 | High |
| Great Barrier Reef | -20.57 | 150.13 | 0.4 | 0.1 | 0.3 | 0.3 | 0.5 | Medial |
| Great Barrier Reef | -20.57 | 150.05 | 0.4 | 0.1 | 0.3 | 0.2 | 0.5 | Medial |
| Great Barrier Reef | -20.57 | 150.77 | 0.2 | 0.2 | 0.2 | 0.2 | 0.4 | Medial |
| Great Barrier Reef | -20.56 | 150.56 | 0.2 | 0.2 | 0.2 | 0.4 | 0.5 | Medial |
| Western Australia | -20.56 | 116.79 | 0.7 | 0.2 | 0.6 | 0.8 | 0.9 | Severe |
| Great Barrier Reef | -20.56 | 149.93 | 0.4 | 0.1 | 0.3 | 0.2 | 0.5 | Medial |
| Great Barrier Reef | -20.54 | 149.95 | 0.4 | 0.1 | 0.3 | 0.2 | 0.4 | Medial |
| Great Barrier Reef | -20.54 | 149.95 | 0.4 | 0.1 | 0.3 | 0.2 | 0.4 | Medial |
| Great Barrier Reef | -20.54 | 149.11 | 0.6 | 0.2 | 0.4 | 0.6 | 0.8 | High |
| Great Barrier Reef | -20.54 | 149.12 | 0.6 | 0.2 | 0.4 | 0.6 | 0.8 | High |
| Great Barrier Reef | -20.54 | 150.38 | 0.3 | 0.2 | 0.3 | 0.6 | 0.7 | High |
| Great Barrier Reef | -20.54 | 150.38 | 0.3 | 0.2 | 0.3 | 0.6 | 0.7 | High |
| Great Barrier Reef | -20.53 | 149.09 | 0.6 | 0.2 | 0.4 | 0.7 | 0.8 | Severe |
| Great Barrier Reef | -20.53 | 150.76 | 0.2 | 0.2 | 0.2 | 0.2 | 0.4 | Medial |
| Great Barrier Reef | -20.52 | 150.24 | 0.3 | 0.2 | 0.3 | 0.5 | 0.6 | Medial |
| Great Barrier Reef | -20.51 | 149.05 | 0.6 | 0.2 | 0.4 | 0.7 | 0.8 | Severe |
| Western Indian Ocean | -20.50 | 57.39 | 0.2 | 0.4 | 0.2 | 0.1 | 0.3 | Low |
| Western Indian Ocean | -20.50 | 55.00 | 0.2 | 0.4 | 0.3 | 0.0 | 0.3 | Low |
| Western Indian Ocean | -20.50 | 57.50 | 0.2 | 0.4 | 0.2 | 0.1 | 0.3 | Low |
| Great Barrier Reef | -20.49 | 149.06 | 0.6 | 0.2 | 0.4 | 0.7 | 0.8 | Severe |
| Great Barrier Reef | -20.48 | 150.21 | 0.3 | 0.2 | 0.3 | 0.5 | 0.6 | Medial |
| Great Barrier Reef | -20.48 | 150.21 | 0.3 | 0.2 | 0.3 | 0.6 | 0.7 | High |
| Great Barrier Reef | -20.48 | 149.05 | 0.5 | 0.2 | 0.4 | 0.7 | 0.8 | High |
| Great Barrier Reef | -20.48 | 149.06 | 0.7 | 0.2 | 0.5 | 0.6 | 0.8 | High |
| Great Barrier Reef | -20.47 | 150.29 | 0.3 | 0.2 | 0.3 | 0.6 | 0.7 | High |
| Great Barrier Reef | -20.47 | 149.13 | 0.6 | 0.2 | 0.4 | 0.6 | 0.8 | High |
| Great Barrier Reef | -20.47 | 150.29 | 0.3 | 0.2 | 0.3 | 0.6 | 0.7 | High |
| Great Barrier Reef | -20.47 | 149.04 | 0.5 | 0.2 | 0.4 | 0.7 | 0.8 | High |
| Great Barrier Reef | -20.46 | 149.10 | 0.7 | 0.2 | 0.5 | 0.6 | 0.8 | High |
| Western Indian Ocean | -20.46 | 57.31 | 0.1 | 0.4 | 0.2 | 0.0 | 0.2 | Low |
| Great Barrier Reef | -20.46 | 149.08 | 0.7 | 0.2 | 0.5 | 0.6 | 0.8 | High |
| Great Barrier Reef | -20.46 | 149.05 | 0.5 | 0.2 | 0.4 | 0.7 | 0.8 | High |
| Western Indian Ocean | -20.45 | 57.71 | 0.5 | 0.4 | 0.4 | 0.1 | 0.5 | Medial |
| Great Barrier Reef | -20.45 | 150.24 | 0.3 | 0.2 | 0.3 | 0.6 | 0.7 | High |
| Great Barrier Reef | -20.45 | 150.24 | 0.3 | 0.2 | 0.3 | 0.6 | 0.7 | High |
| Western Indian Ocean | -20.44 | 57.71 | 0.5 | 0.4 | 0.4 | 0.1 | 0.5 | Medial |
| Great Barrier Reef | -20.44 | 149.03 | 0.5 | 0.2 | 0.4 | 0.7 | 0.8 | High |
| Great Barrier Reef | -20.43 | 149.08 | 0.7 | 0.2 | 0.5 | 0.6 | 0.8 | High |
| Western Indian Ocean | -20.43 | 57.34 | 0.2 | ? | 0.2 | 0.1 | 0.3 | Low |
| Great Barrier Reef | -20.42 | 149.03 | 0.6 | 0.2 | 0.4 | 0.7 | 0.8 | High |
| Great Barrier Reef | -20.42 | 149.01 | 0.6 | 0.2 | 0.4 | 0.7 | 0.8 | High |
| Great Barrier Reef | -20.42 | 149.02 | 0.6 | 0.2 | 0.4 | 0.7 | 0.8 | High |
| Great Barrier Reef | -20.40 | 150.24 | 0.3 | 0.2 | 0.3 | 0.6 | 0.7 | High |
| Great Barrier Reef | -20.40 | 150.24 | 0.3 | 0.2 | 0.3 | 0.6 | 0.7 | High |
| Great Barrier Reef | -20.39 | 149.03 | 0.6 | 0.2 | 0.4 | 0.7 | 0.8 | High |
| Great Barrier Reef | -20.38 | 150.76 | 0.2 | 0.3 | 0.2 | 0.2 | 0.4 | Medial |
| Great Barrier Reef | -20.37 | 148.96 | 0.6 | 0.2 | 0.4 | 0.7 | 0.8 | High |
| Great Barrier Reef | -20.37 | 148.95 | 0.6 | 0.2 | 0.4 | 0.7 | 0.8 | High |
| Great Barrier Reef | -20.37 | 148.97 | 0.6 | 0.2 | 0.4 | 0.7 | 0.8 | High |
| Great Barrier Reef | -20.36 | 148.96 | 0.6 | 0.2 | 0.4 | 0.7 | 0.8 | High |
| Great Barrier Reef | -20.35 | 150.07 | 0.4 | 0.2 | 0.3 | 0.2 | 0.5 | Medial |
| Great Barrier Reef | -20.35 | 150.10 | 0.4 | 0.2 | 0.3 | 0.4 | 0.6 | Medial |
| Great Barrier Reef | -20.35 | 150.07 | 0.4 | 0.2 | 0.3 | 0.2 | 0.5 | Medial |
| Great Barrier Reef | -20.35 | 150.10 | 0.4 | 0.2 | 0.3 | 0.4 | 0.6 | Medial |
| Great Barrier Reef | -20.35 | 150.65 | 0.2 | 0.2 | 0.2 | 0.2 | 0.3 | Low |
| Great Barrier Reef | -20.35 | 150.71 | 0.2 | 0.2 | 0.2 | 0.2 | 0.4 | Medial |
| Great Barrier Reef | -20.35 | 150.20 | 0.2 | 0.2 | 0.2 | 0.6 | 0.7 | High |
| Great Barrier Reef | -20.34 | 150.20 | 0.2 | 0.2 | 0.2 | 0.6 | 0.7 | High |
| Great Barrier Reef | -20.34 | 150.13 | 0.3 | 0.2 | 0.3 | 0.6 | 0.7 | High |
| Great Barrier Reef | -20.34 | 150.13 | 0.3 | 0.2 | 0.3 | 0.6 | 0.7 | High |
| Great Barrier Reef | -20.32 | 148.93 | 0.6 | 0.2 | 0.5 | 0.6 | 0.8 | High |
| Great Barrier Reef | -20.30 | 150.61 | 0.2 | 0.2 | 0.2 | 0.2 | 0.3 | Low |
| Great Barrier Reef | -20.30 | 149.07 | 0.5 | 0.2 | 0.4 | 0.5 | 0.7 | High |
| Western Indian Ocean | -20.30 | 57.80 | 0.3 | 0.4 | 0.3 | 0.2 | 0.4 | Medial |
| Great Barrier Reef | -20.30 | 150.10 | 0.3 | 0.2 | 0.3 | 0.5 | 0.7 | Medial |
| Great Barrier Reef | -20.30 | 150.10 | 0.3 | 0.2 | 0.3 | 0.5 | 0.7 | Medial |
| Western Indian Ocean | -20.29 | 57.80 | 0.3 | 0.4 | 0.3 | 0.2 | 0.4 | Medial |
| Western Indian Ocean | -20.29 | 57.82 | 0.3 | 0.4 | 0.3 | 0.2 | 0.4 | Medial |
| Great Barrier Reef | -20.28 | 150.56 | 0.2 | 0.2 | 0.2 | 0.2 | 0.4 | Low |
| Western Indian Ocean | -20.28 | 57.55 | 0.1 | 0.4 | 0.2 | 0.0 | 0.2 | Low |
| Great Barrier Reef | -20.28 | 149.10 | 0.5 | 0.2 | 0.4 | 0.5 | 0.7 | High |
| Great Barrier Reef | -20.28 | 149.11 | 0.5 | 0.2 | 0.4 | 0.5 | 0.7 | High |
| Great Barrier Reef | -20.27 | 149.08 | 0.5 | 0.2 | 0.4 | 0.5 | 0.7 | High |
| Great Barrier Reef | -20.26 | 149.11 | 0.5 | 0.2 | 0.4 | 0.5 | 0.7 | High |
| Great Barrier Reef | -20.26 | 148.81 | 0.6 | 0.2 | 0.4 | 0.6 | 0.8 | High |
| Great Barrier Reef | -20.26 | 148.81 | 0.6 | 0.2 | 0.4 | 0.6 | 0.8 | High |
| Great Barrier Reef | -20.26 | 149.08 | 0.5 | 0.2 | 0.4 | 0.5 | 0.7 | High |
| Great Barrier Reef | -20.26 | 148.84 | 0.6 | 0.2 | 0.4 | 0.6 | 0.8 | High |
| Great Barrier Reef | -20.26 | 148.84 | 0.6 | 0.2 | 0.4 | 0.6 | 0.8 | High |
| Great Barrier Reef | -20.26 | 148.84 | 0.6 | 0.2 | 0.4 | 0.6 | 0.8 | High |
| Great Barrier Reef | -20.26 | 150.51 | 0.2 | 0.2 | 0.2 | 0.2 | 0.4 | Medial |
| Great Barrier Reef | -20.25 | 148.82 | 0.6 | 0.2 | 0.4 | 0.7 | 0.8 | High |
| Great Barrier Reef | -20.25 | 148.83 | 0.6 | 0.2 | 0.4 | 0.7 | 0.8 | High |
| Great Barrier Reef | -20.25 | 149.18 | 0.5 | 0.2 | 0.4 | 0.5 | 0.7 | High |
| Great Barrier Reef | -20.25 | 148.83 | 0.6 | 0.2 | 0.4 | 0.7 | 0.8 | High |
| Western Indian Ocean | -20.25 | 57.80 | 0.4 | 0.4 | 0.3 | 0.2 | 0.5 | Medial |
| Great Barrier Reef | -20.24 | 149.15 | 0.5 | 0.2 | 0.4 | 0.5 | 0.7 | High |
| Great Barrier Reef | -20.24 | 149.16 | 0.5 | 0.2 | 0.4 | 0.5 | 0.7 | High |
| Great Barrier Reef | -20.23 | 149.15 | 0.5 | 0.2 | 0.4 | 0.5 | 0.7 | High |
| Great Barrier Reef | -20.22 | 148.81 | 0.6 | 0.2 | 0.4 | 0.7 | 0.8 | High |
| Great Barrier Reef | -20.22 | 148.81 | 0.6 | 0.2 | 0.4 | 0.7 | 0.8 | High |
| Great Barrier Reef | -20.20 | 149.11 | 0.6 | 0.2 | 0.4 | 0.5 | 0.7 | High |
| Great Barrier Reef | -20.19 | 150.08 | 0.2 | 0.2 | 0.2 | 0.6 | 0.7 | High |
| Great Barrier Reef | -20.19 | 150.47 | 0.2 | 0.3 | 0.2 | 0.2 | 0.4 | Medial |
| Great Barrier Reef | -20.19 | 150.08 | 0.2 | 0.2 | 0.2 | 0.6 | 0.7 | High |
| Great Barrier Reef | -20.18 | 149.03 | 0.5 | 0.2 | 0.4 | 0.5 | 0.7 | High |
| Great Barrier Reef | -20.18 | 150.37 | 0.2 | 0.2 | 0.2 | 0.4 | 0.5 | Medial |
| Great Barrier Reef | -20.17 | 149.03 | 0.5 | 0.2 | 0.4 | 0.5 | 0.7 | High |
| Great Barrier Reef | -20.17 | 149.03 | 0.5 | 0.2 | 0.4 | 0.5 | 0.7 | High |
| Great Barrier Reef | -20.17 | 149.03 | 0.5 | 0.2 | 0.4 | 0.5 | 0.7 | High |
| Great Barrier Reef | -20.17 | 149.01 | 0.6 | 0.2 | 0.4 | 0.5 | 0.7 | High |
| Great Barrier Reef | -20.17 | 149.04 | 0.5 | 0.2 | 0.4 | 0.5 | 0.7 | High |
| Great Barrier Reef | -20.17 | 149.02 | 0.6 | 0.2 | 0.4 | 0.5 | 0.7 | High |
| Western Indian Ocean | -20.16 | 57.45 | 0.3 | 0.4 | 0.3 | 0.1 | 0.3 | Low |
| Great Barrier Reef | -20.16 | 149.03 | 0.5 | 0.2 | 0.4 | 0.5 | 0.7 | High |
| Great Barrier Reef | -20.16 | 149.03 | 0.5 | 0.2 | 0.4 | 0.5 | 0.7 | High |
| Great Barrier Reef | -20.16 | 149.07 | 0.5 | 0.1 | 0.4 | 0.5 | 0.7 | High |
| Great Barrier Reef | -20.16 | 149.07 | 0.5 | 0.1 | 0.4 | 0.5 | 0.7 | High |
| Great Barrier Reef | -20.15 | 148.61 | 0.6 | 0.1 | 0.4 | 0.6 | 0.7 | High |
| Great Barrier Reef | -20.15 | 148.61 | 0.6 | 0.1 | 0.4 | 0.6 | 0.7 | High |
| Great Barrier Reef | -20.15 | 149.07 | 0.5 | 0.1 | 0.4 | 0.5 | 0.7 | High |
| Great Barrier Reef | -20.14 | 150.44 | 0.2 | 0.3 | 0.2 | 0.2 | 0.4 | Medial |
| Western Indian Ocean | -20.13 | 57.47 | 0.3 | 0.4 | 0.3 | 0.1 | 0.3 | Low |
| Great Barrier Reef | -20.13 | 149.94 | 0.3 | 0.2 | 0.3 | 0.2 | 0.4 | Medial |
| Great Barrier Reef | -20.13 | 149.94 | 0.3 | 0.2 | 0.3 | 0.2 | 0.4 | Medial |
| Great Barrier Reef | -20.13 | 148.61 | 0.6 | 0.1 | 0.4 | 0.6 | 0.7 | High |
| Great Barrier Reef | -20.13 | 149.94 | 0.3 | 0.2 | 0.3 | 0.2 | 0.4 | Medial |
| Great Barrier Reef | -20.13 | 148.61 | 0.6 | 0.1 | 0.4 | 0.6 | 0.7 | High |
| Great Barrier Reef | -20.12 | 150.01 | 0.2 | 0.2 | 0.2 | 0.4 | 0.5 | Medial |
| Great Barrier Reef | -20.12 | 148.66 | 0.5 | 0.2 | 0.4 | 0.6 | 0.7 | High |
| Great Barrier Reef | -20.11 | 148.65 | 0.5 | 0.2 | 0.4 | 0.6 | 0.7 | High |
| Great Barrier Reef | -20.11 | 148.65 | 0.5 | 0.2 | 0.4 | 0.6 | 0.7 | High |
| Great Barrier Reef | -20.11 | 148.71 | 0.5 | 0.2 | 0.4 | 0.5 | 0.7 | High |
| Great Barrier Reef | -20.11 | 150.32 | 0.2 | 0.2 | 0.2 | 0.4 | 0.5 | Medial |
| Great Barrier Reef | -20.10 | 148.32 | 0.7 | 0.1 | 0.5 | 0.6 | 0.8 | High |
| Great Barrier Reef | -20.10 | 149.93 | 0.2 | 0.2 | 0.2 | 0.2 | 0.4 | Medial |
| Great Barrier Reef | -20.10 | 148.61 | 0.6 | 0.2 | 0.4 | 0.6 | 0.8 | High |
| Western Indian Ocean | -20.10 | 57.50 | 0.6 | 0.4 | 0.6 | 0.0 | 0.6 | Medial |
| Great Barrier Reef | -20.10 | 148.61 | 0.6 | 0.2 | 0.4 | 0.6 | 0.8 | High |
| Great Barrier Reef | -20.09 | 148.88 | 0.6 | 0.2 | 0.4 | 0.5 | 0.7 | High |
| Western Indian Ocean | -20.09 | 57.51 | 0.3 | 0.4 | 0.3 | 0.0 | 0.3 | Low |
| Western Indian Ocean | -20.09 | 57.51 | 0.3 | 0.4 | 0.3 | 0.0 | 0.3 | Low |
| Great Barrier Reef | -20.08 | 148.53 | 0.6 | 0.1 | 0.4 | 0.6 | 0.8 | High |
| Great Barrier Reef | -20.08 | 148.89 | 0.6 | 0.2 | 0.4 | 0.5 | 0.7 | High |
| Great Barrier Reef | -20.08 | 148.88 | 0.5 | 0.2 | 0.4 | 0.5 | 0.7 | High |
| Great Barrier Reef | -20.08 | 148.96 | 0.6 | 0.2 | 0.4 | 0.5 | 0.7 | High |
| Great Barrier Reef | -20.08 | 148.49 | 0.7 | 0.1 | 0.5 | 0.6 | 0.8 | High |
| Great Barrier Reef | -20.08 | 148.49 | 0.7 | 0.1 | 0.5 | 0.6 | 0.8 | High |
| Great Barrier Reef | -20.07 | 148.93 | 0.6 | 0.2 | 0.4 | 0.5 | 0.7 | High |
| Great Barrier Reef | -20.07 | 148.93 | 0.6 | 0.2 | 0.4 | 0.5 | 0.7 | High |
| Great Barrier Reef | -20.07 | 148.90 | 0.6 | 0.2 | 0.4 | 0.5 | 0.7 | High |
| Great Barrier Reef | -20.07 | 148.54 | 0.6 | 0.1 | 0.4 | 0.6 | 0.8 | High |
| Great Barrier Reef | -20.07 | 148.54 | 0.6 | 0.1 | 0.4 | 0.6 | 0.8 | High |
| Great Barrier Reef | -20.07 | 149.93 | 0.2 | 0.2 | 0.2 | 0.2 | 0.4 | Medial |
| Great Barrier Reef | -20.07 | 148.96 | 0.6 | 0.2 | 0.4 | 0.5 | 0.7 | High |
| Great Barrier Reef | -20.07 | 148.95 | 0.6 | 0.2 | 0.4 | 0.5 | 0.7 | High |
| Great Barrier Reef | -20.07 | 148.89 | 0.6 | 0.2 | 0.4 | 0.5 | 0.7 | High |
| Great Barrier Reef | -20.07 | 148.47 | 0.7 | 0.1 | 0.5 | 0.6 | 0.8 | High |
| Great Barrier Reef | -20.07 | 148.89 | 0.6 | 0.2 | 0.4 | 0.5 | 0.7 | High |
| Great Barrier Reef | -20.06 | 148.95 | 0.6 | 0.2 | 0.4 | 0.5 | 0.7 | High |
| Great Barrier Reef | -20.06 | 148.89 | 0.6 | 0.2 | 0.4 | 0.5 | 0.7 | High |
| Great Barrier Reef | -20.06 | 148.90 | 0.6 | 0.2 | 0.4 | 0.5 | 0.7 | High |
| Great Barrier Reef | -20.05 | 148.47 | 0.7 | 0.1 | 0.5 | 0.6 | 0.8 | High |
| Western Indian Ocean | -20.05 | 57.53 | 0.1 | 0.4 | 0.2 | 0.1 | 0.2 | Low |
| Great Barrier Reef | -20.03 | 149.69 | 0.3 | 0.2 | 0.3 | 0.2 | 0.4 | Medial |
| Great Barrier Reef | -20.03 | 149.95 | 0.3 | 0.2 | 0.2 | 0.4 | 0.5 | Medial |
| Great Barrier Reef | -20.03 | 149.69 | 0.3 | 0.2 | 0.3 | 0.2 | 0.4 | Medial |
| Great Barrier Reef | -20.03 | 148.63 | 0.5 | 0.1 | 0.4 | 0.6 | 0.7 | High |
| Great Barrier Reef | -20.02 | 148.62 | 0.5 | 0.1 | 0.4 | 0.6 | 0.7 | High |
| Great Barrier Reef | -20.02 | 148.62 | 0.5 | 0.1 | 0.4 | 0.6 | 0.7 | High |
| Great Barrier Reef | -20.01 | 149.72 | 0.3 | 0.2 | 0.3 | 0.2 | 0.4 | Medial |
| Great Barrier Reef | -20.01 | 150.32 | 0.2 | 0.3 | 0.2 | 0.3 | 0.4 | Medial |
| Polynesia | -20.00 | -175.00 | 0.5 | 0.4 | 0.4 | 0.1 | 0.5 | Medial |
| Western Indian Ocean | -20.00 | 57.50 | 0.1 | 0.4 | 0.2 | 0.1 | 0.2 | Low |
| Great Barrier Reef | -20.00 | 148.56 | 0.5 | 0.1 | 0.4 | 0.6 | 0.7 | High |
| Western Indian Ocean | -19.99 | 57.64 | 0.5 | 0.4 | 0.5 | 0.1 | 0.5 | Medial |
| Great Barrier Reef | -19.98 | 148.36 | 0.6 | 0.1 | 0.4 | 0.6 | 0.8 | High |
| Great Barrier Reef | -19.98 | 148.36 | 0.5 | 0.1 | 0.4 | 0.6 | 0.7 | High |
| Western Indian Ocean | -19.98 | 57.64 | 0.5 | 0.4 | 0.5 | 0.1 | 0.5 | Medial |
| Great Barrier Reef | -19.88 | 147.97 | 0.6 | 0.1 | 0.5 | 0.9 | 1.0 | Severe |
| Great Barrier Reef | -19.88 | 149.58 | 0.3 | 0.2 | 0.3 | 0.2 | 0.4 | Medial |
| Great Barrier Reef | -19.88 | 149.58 | 0.3 | 0.2 | 0.3 | 0.2 | 0.4 | Medial |
| Great Barrier Reef | -19.88 | 147.93 | 0.6 | 0.1 | 0.5 | 0.6 | 0.8 | Severe |
| Great Barrier Reef | -19.86 | 147.89 | 0.7 | 0.1 | 0.5 | 0.6 | 0.8 | Severe |
| Great Barrier Reef | -19.85 | 147.90 | 0.7 | 0.1 | 0.5 | 0.6 | 0.8 | Severe |
| Great Barrier Reef | -19.85 | 149.90 | 0.3 | 0.3 | 0.2 | 0.2 | 0.4 | Medial |
| Western Indian Ocean | -19.83 | 63.50 | 0.2 | 0.4 | 0.3 | 0.2 | 0.4 | Medial |
| Great Barrier Reef | -19.82 | 149.12 | 0.3 | 0.2 | 0.3 | 0.2 | 0.4 | Medial |
| Great Barrier Reef | -19.81 | 149.07 | 0.4 | 0.2 | 0.3 | 0.2 | 0.4 | Medial |
| Great Barrier Reef | -19.81 | 149.07 | 0.4 | 0.2 | 0.3 | 0.2 | 0.4 | Medial |
| Great Barrier Reef | -19.81 | 149.43 | 0.3 | 0.2 | 0.3 | 0.2 | 0.4 | Medial |
| Great Barrier Reef | -19.81 | 149.08 | 0.4 | 0.2 | 0.3 | 0.2 | 0.4 | Medial |
| Great Barrier Reef | -19.80 | 149.42 | 0.3 | 0.2 | 0.3 | 0.2 | 0.4 | Medial |
| Great Barrier Reef | -19.80 | 149.20 | 0.4 | 0.2 | 0.4 | 0.2 | 0.5 | Medial |
| Great Barrier Reef | -19.80 | 150.15 | 0.2 | 0.3 | 0.2 | 0.3 | 0.4 | Medial |
| Great Barrier Reef | -19.80 | 149.20 | 0.4 | 0.2 | 0.4 | 0.2 | 0.5 | Medial |
| Western Indian Ocean | -19.80 | 63.30 | 0.2 | 0.3 | 0.3 | 0.8 | 0.8 | Severe |
| Polynesia | -19.78 | -174.45 | 0.5 | 0.5 | 0.5 | 0.2 | 0.6 | Medial |
| Great Barrier Reef | -19.77 | 149.38 | 0.3 | 0.2 | 0.3 | 0.2 | 0.4 | Medial |
| Great Barrier Reef | -19.76 | 149.23 | 0.4 | 0.2 | 0.4 | 0.2 | 0.4 | Medial |
| Great Barrier Reef | -19.76 | 149.23 | 0.4 | 0.2 | 0.4 | 0.2 | 0.4 | Medial |
| Great Barrier Reef | -19.76 | 149.40 | 0.3 | 0.2 | 0.3 | 0.2 | 0.4 | Medial |
| Great Barrier Reef | -19.76 | 149.37 | 0.4 | 0.2 | 0.3 | 0.2 | 0.4 | Medial |
| Great Barrier Reef | -19.76 | 149.23 | 0.4 | 0.2 | 0.4 | 0.2 | 0.4 | Medial |
| Great Barrier Reef | -19.75 | 150.08 | 0.2 | 0.3 | 0.2 | 0.3 | 0.4 | Medial |
| Great Barrier Reef | -19.75 | 149.31 | 0.4 | 0.2 | 0.3 | 0.2 | 0.4 | Medial |
| Western Indian Ocean | -19.75 | 63.42 | 0.4 | 0.4 | 0.4 | 1.0 | 1.0 | Severe |
| Great Barrier Reef | -19.75 | 149.49 | 0.4 | 0.2 | 0.3 | 0.2 | 0.4 | Medial |
| Great Barrier Reef | -19.74 | 149.96 | 0.2 | 0.3 | 0.2 | 0.2 | 0.4 | Medial |
| Great Barrier Reef | -19.73 | 149.17 | 0.3 | 0.2 | 0.3 | 0.2 | 0.4 | Medial |
| Great Barrier Reef | -19.73 | 148.36 | 0.5 | 0.2 | 0.4 | 0.5 | 0.7 | High |
| Great Barrier Reef | -19.73 | 148.36 | 0.5 | 0.2 | 0.4 | 0.5 | 0.7 | High |
| Great Barrier Reef | -19.73 | 148.36 | 0.5 | 0.2 | 0.4 | 0.5 | 0.7 | High |
| Great Barrier Reef | -19.72 | 148.36 | 0.5 | 0.2 | 0.4 | 0.5 | 0.7 | High |
| Great Barrier Reef | -19.72 | 149.20 | 0.4 | 0.2 | 0.3 | 0.2 | 0.4 | Medial |
| Great Barrier Reef | -19.72 | 149.98 | 0.2 | 0.3 | 0.2 | 0.2 | 0.4 | Medial |
| Great Barrier Reef | -19.71 | 149.37 | 0.3 | 0.2 | 0.3 | 0.2 | 0.4 | Medial |
| Western Indian Ocean | -19.70 | 63.51 | 0.3 | 0.4 | 0.3 | 0.1 | 0.4 | Medial |
| Great Barrier Reef | -19.69 | 149.98 | 0.2 | 0.3 | 0.2 | 0.2 | 0.4 | Medial |
| Great Barrier Reef | -19.69 | 149.19 | 0.3 | 0.2 | 0.3 | 0.2 | 0.4 | Medial |
| Great Barrier Reef | -19.69 | 149.19 | 0.3 | 0.2 | 0.3 | 0.2 | 0.4 | Medial |
| Western Indian Ocean | -19.68 | 63.58 | 0.3 | 0.4 | 0.3 | 0.1 | 0.3 | Low |
| Western Indian Ocean | -19.67 | 63.48 | 0.2 | 0.3 | 0.3 | 0.1 | 0.3 | Low |
| Western Indian Ocean | -19.67 | 63.41 | 0.3 | 0.3 | 0.3 | 0.0 | 0.3 | Low |
| Western Indian Ocean | -19.67 | 63.41 | 0.3 | 0.3 | 0.3 | 0.0 | 0.3 | Low |
| Western Indian Ocean | -19.67 | 63.41 | 0.3 | 0.3 | 0.3 | 0.0 | 0.3 | Low |
| Great Barrier Reef | -19.67 | 149.93 | 0.2 | 0.3 | 0.2 | 0.2 | 0.4 | Medial |
| Western Indian Ocean | -19.66 | 63.44 | 0.2 | 0.3 | 0.3 | 0.0 | 0.3 | Low |
| Western Indian Ocean | -19.64 | 63.40 | 0.2 | 0.3 | 0.3 | 0.0 | 0.3 | Low |
| Great Barrier Reef | -19.63 | 149.67 | 0.2 | 0.3 | 0.2 | 0.2 | 0.4 | Medial |
| Great Barrier Reef | -19.63 | 149.67 | 0.2 | 0.3 | 0.2 | 0.2 | 0.4 | Medial |
| Great Barrier Reef | -19.63 | 149.88 | 0.3 | 0.3 | 0.3 | 0.2 | 0.4 | Medial |
| Great Barrier Reef | -19.62 | 149.85 | 0.2 | 0.3 | 0.2 | 0.2 | 0.4 | Medial |
| Great Barrier Reef | -19.61 | 149.63 | 0.2 | 0.3 | 0.2 | 0.2 | 0.4 | Medial |
| Great Barrier Reef | -19.61 | 149.73 | 0.2 | 0.3 | 0.2 | 0.2 | 0.4 | Medial |
| Great Barrier Reef | -19.60 | 149.77 | 0.2 | 0.3 | 0.2 | 0.2 | 0.4 | Medial |
| Great Barrier Reef | -19.56 | 148.93 | 0.2 | 0.2 | 0.3 | 0.2 | 0.4 | Medial |
| Great Barrier Reef | -19.56 | 149.60 | 0.3 | 0.3 | 0.2 | 0.2 | 0.4 | Medial |
| Great Barrier Reef | -19.55 | 149.36 | 0.2 | 0.3 | 0.2 | 0.2 | 0.4 | Low |
| Great Barrier Reef | -19.55 | 149.28 | 0.2 | 0.3 | 0.2 | 0.2 | 0.3 | Low |
| Great Barrier Reef | -19.54 | 148.94 | 0.3 | 0.2 | 0.3 | 0.2 | 0.4 | Medial |
| Great Barrier Reef | -19.54 | 148.94 | 0.3 | 0.2 | 0.3 | 0.2 | 0.4 | Medial |
| Great Barrier Reef | -19.53 | 149.37 | 0.2 | 0.3 | 0.2 | 0.2 | 0.4 | Low |
| Great Barrier Reef | -19.53 | 148.88 | 0.4 | 0.3 | 0.3 | 0.2 | 0.4 | Medial |
| Great Barrier Reef | -19.53 | 149.40 | 0.2 | 0.3 | 0.2 | 0.2 | 0.3 | Low |
| Great Barrier Reef | -19.53 | 148.98 | 0.2 | 0.2 | 0.2 | 0.2 | 0.4 | Medial |
| Great Barrier Reef | -19.53 | 148.98 | 0.2 | 0.2 | 0.2 | 0.2 | 0.4 | Medial |
| Great Barrier Reef | -19.52 | 149.58 | 0.3 | 0.3 | 0.3 | 0.2 | 0.4 | Medial |
| Great Barrier Reef | -19.50 | 149.12 | 0.2 | 0.3 | 0.3 | 0.1 | 0.4 | Medial |
| Great Barrier Reef | -19.50 | 148.74 | 0.3 | 0.3 | 0.3 | 0.1 | 0.4 | Medial |
| Great Barrier Reef | -19.50 | 148.78 | 0.3 | 0.3 | 0.3 | 0.1 | 0.4 | Medial |
| Great Barrier Reef | -19.50 | 148.74 | 0.3 | 0.3 | 0.3 | 0.1 | 0.4 | Medial |
| Western Indian Ocean | -19.50 | 57.50 | 0.2 | 0.4 | 0.3 | 0.0 | 0.3 | Low |
| Western Indian Ocean | -19.50 | 63.50 | 0.2 | 0.4 | 0.3 | 0.0 | 0.3 | Low |
| Great Barrier Reef | -19.49 | 149.05 | 0.3 | 0.3 | 0.3 | 0.2 | 0.4 | Medial |
| Great Barrier Reef | -19.49 | 148.90 | 0.3 | 0.3 | 0.3 | 0.2 | 0.4 | Medial |
| Great Barrier Reef | -19.49 | 148.90 | 0.3 | 0.3 | 0.3 | 0.2 | 0.4 | Medial |
| Great Barrier Reef | -19.48 | 148.82 | 0.3 | 0.3 | 0.3 | 0.1 | 0.4 | Medial |
| Great Barrier Reef | -19.47 | 149.17 | 0.2 | 0.3 | 0.2 | 0.1 | 0.3 | Low |
| Great Barrier Reef | -19.47 | 149.17 | 0.2 | 0.3 | 0.2 | 0.1 | 0.3 | Low |
| Great Barrier Reef | -19.44 | 148.85 | 0.3 | 0.3 | 0.3 | 0.2 | 0.4 | Medial |
| Great Barrier Reef | -19.43 | 148.66 | 0.3 | 0.3 | 0.3 | 0.2 | 0.4 | Medial |
| Great Barrier Reef | -19.43 | 148.66 | 0.3 | 0.3 | 0.3 | 0.2 | 0.4 | Medial |
| Great Barrier Reef | -19.42 | 148.85 | 0.3 | 0.3 | 0.3 | 0.2 | 0.4 | Medial |
| Great Barrier Reef | -19.40 | 148.78 | 0.3 | 0.3 | 0.3 | 0.1 | 0.4 | Medial |
| Great Barrier Reef | -19.40 | 148.87 | 0.3 | 0.3 | 0.3 | 0.2 | 0.4 | Medial |
| Great Barrier Reef | -19.39 | 146.72 | 0.8 | 0.1 | 0.6 | 1.0 | 1.0 | Severe |
| Great Barrier Reef | -19.39 | 148.09 | 0.5 | 0.3 | 0.4 | 0.3 | 0.6 | Medial |
| Great Barrier Reef | -19.39 | 148.09 | 0.5 | 0.3 | 0.4 | 0.3 | 0.6 | Medial |
| Great Barrier Reef | -19.38 | 148.09 | 0.5 | 0.3 | 0.4 | 0.3 | 0.6 | Medial |
| Great Barrier Reef | -19.37 | 148.34 | 0.4 | 0.3 | 0.4 | 0.3 | 0.5 | Medial |
| Great Barrier Reef | -19.36 | 148.64 | 0.2 | 0.3 | 0.3 | 0.1 | 0.4 | Medial |
| Great Barrier Reef | -19.34 | 148.63 | 0.2 | 0.3 | 0.3 | 0.1 | 0.4 | Medial |
| Great Barrier Reef | -19.33 | 149.05 | 0.2 | 0.3 | 0.2 | 0.1 | 0.3 | Low |
| Great Barrier Reef | -19.32 | 148.43 | 0.3 | 0.3 | 0.3 | 0.3 | 0.5 | Medial |
| Great Barrier Reef | -19.32 | 148.43 | 0.3 | 0.3 | 0.3 | 0.3 | 0.5 | Medial |
| Great Barrier Reef | -19.30 | 148.08 | 0.4 | 0.3 | 0.3 | 0.3 | 0.6 | Medial |
| Great Barrier Reef | -19.30 | 148.08 | 0.4 | 0.3 | 0.3 | 0.3 | 0.6 | Medial |
| Great Barrier Reef | -19.28 | 148.35 | 0.3 | 0.3 | 0.3 | 0.3 | 0.5 | Medial |
| Great Barrier Reef | -19.28 | 148.35 | 0.3 | 0.3 | 0.3 | 0.3 | 0.5 | Medial |
| Great Barrier Reef | -19.26 | 148.55 | 0.3 | 0.3 | 0.3 | 0.1 | 0.4 | Medial |
| Great Barrier Reef | -19.26 | 148.63 | 0.2 | 0.3 | 0.2 | 0.1 | 0.3 | Low |
| Great Barrier Reef | -19.26 | 148.63 | 0.2 | 0.3 | 0.2 | 0.1 | 0.3 | Low |
| Great Barrier Reef | -19.22 | 148.90 | 0.3 | 0.3 | 0.3 | 0.2 | 0.4 | Medial |
| Great Barrier Reef | -19.21 | 148.54 | 0.2 | 0.3 | 0.3 | 0.1 | 0.4 | Medial |
| Great Barrier Reef | -19.21 | 148.17 | 0.4 | 0.3 | 0.3 | 0.3 | 0.5 | Medial |
| Great Barrier Reef | -19.21 | 148.54 | 0.2 | 0.3 | 0.3 | 0.1 | 0.4 | Medial |
| Great Barrier Reef | -19.20 | 148.82 | 0.4 | 0.3 | 0.3 | 0.2 | 0.4 | Medial |
| Great Barrier Reef | -19.20 | 146.81 | 0.8 | 0.2 | 0.6 | 1.0 | 1.0 | Severe |
| Great Barrier Reef | -19.20 | 146.81 | 0.8 | 0.2 | 0.6 | 1.0 | 1.0 | Severe |
| Great Barrier Reef | -19.20 | 146.82 | 0.8 | 0.2 | 0.6 | 1.0 | 1.0 | Severe |
| Great Barrier Reef | -19.18 | 147.06 | 0.5 | 0.2 | 0.4 | 0.6 | 0.7 | High |
| Great Barrier Reef | -19.18 | 147.02 | 0.5 | 0.2 | 0.4 | 0.6 | 0.8 | High |
| Great Barrier Reef | -19.18 | 148.38 | 0.3 | 0.3 | 0.3 | 0.3 | 0.5 | Medial |
| Great Barrier Reef | -19.18 | 146.81 | 0.8 | 0.2 | 0.6 | 1.0 | 1.0 | Severe |
| Great Barrier Reef | -19.18 | 148.38 | 0.3 | 0.3 | 0.3 | 0.3 | 0.5 | Medial |
| Great Barrier Reef | -19.17 | 146.81 | 0.8 | 0.2 | 0.6 | 1.0 | 1.0 | Severe |
| Great Barrier Reef | -19.16 | 146.87 | 0.6 | 0.2 | 0.4 | 0.8 | 0.9 | Severe |
| Great Barrier Reef | -19.14 | 148.31 | 0.3 | 0.3 | 0.3 | 0.3 | 0.5 | Medial |
| Great Barrier Reef | -19.13 | 148.47 | 0.3 | 0.3 | 0.3 | 0.1 | 0.4 | Medial |
| Great Barrier Reef | -19.12 | 146.88 | 0.6 | 0.2 | 0.4 | 0.8 | 0.9 | Severe |
| Great Barrier Reef | -19.12 | 146.88 | 0.6 | 0.2 | 0.4 | 0.8 | 0.9 | Severe |
| Great Barrier Reef | -19.12 | 146.76 | 0.6 | 0.2 | 0.5 | 0.9 | 0.9 | Severe |
| Great Barrier Reef | -19.11 | 146.83 | 0.6 | 0.2 | 0.5 | 0.7 | 0.9 | Severe |
| Great Barrier Reef | -19.11 | 146.86 | 0.6 | 0.2 | 0.5 | 0.7 | 0.9 | Severe |
| Great Barrier Reef | -19.10 | 148.29 | 0.4 | 0.3 | 0.3 | 0.3 | 0.6 | Medial |
| Great Barrier Reef | -19.10 | 146.56 | 0.7 | 0.2 | 0.5 | 0.8 | 0.9 | Severe |
| Great Barrier Reef | -19.07 | 148.08 | 0.4 | 0.3 | 0.4 | 0.2 | 0.5 | Medial |
| Great Barrier Reef | -19.07 | 148.18 | 0.4 | 0.3 | 0.3 | 0.3 | 0.5 | Medial |
| Great Barrier Reef | -19.05 | 148.31 | 0.5 | 0.3 | 0.4 | 0.3 | 0.6 | Medial |
| Great Barrier Reef | -19.04 | 146.61 | 0.6 | 0.2 | 0.5 | 0.8 | 0.9 | Severe |
| Great Barrier Reef | -19.04 | 147.93 | 0.4 | 0.3 | 0.3 | 0.6 | 0.7 | High |
| Great Barrier Reef | -19.03 | 146.59 | 0.6 | 0.2 | 0.5 | 0.8 | 0.9 | Severe |
| Great Barrier Reef | -19.03 | 146.62 | 0.6 | 0.2 | 0.5 | 0.8 | 0.9 | Severe |
| Great Barrier Reef | -19.03 | 146.57 | 0.6 | 0.2 | 0.5 | 0.8 | 0.9 | Severe |
| Great Barrier Reef | -19.03 | 146.61 | 0.6 | 0.2 | 0.5 | 0.8 | 0.9 | Severe |
| Great Barrier Reef | -19.03 | 146.63 | 0.6 | 0.2 | 0.5 | 0.8 | 0.9 | Severe |
| Great Barrier Reef | -19.02 | 148.32 | 0.4 | 0.3 | 0.4 | 0.2 | 0.5 | Medial |
| Great Barrier Reef | -19.02 | 148.11 | 0.4 | 0.3 | 0.4 | 0.3 | 0.5 | Medial |
| Great Barrier Reef | -19.02 | 148.33 | 0.4 | 0.3 | 0.4 | 0.2 | 0.5 | Medial |
| Great Barrier Reef | -19.02 | 148.11 | 0.4 | 0.3 | 0.4 | 0.3 | 0.5 | Medial |
| Western Indian Ocean | -19.00 | 46.56 | 0.5 | 0.4 | 0.5 | 0.7 | 0.9 | Severe |
| Great Barrier Reef | -18.98 | 146.62 | 0.5 | 0.2 | 0.4 | 0.8 | 0.9 | Severe |
| Great Barrier Reef | -18.97 | 147.70 | 0.5 | 0.3 | 0.4 | 0.4 | 0.6 | Medial |
| Great Barrier Reef | -18.97 | 146.63 | 0.5 | 0.2 | 0.4 | 0.8 | 0.9 | Severe |
| Great Barrier Reef | -18.96 | 146.63 | 0.5 | 0.2 | 0.4 | 0.8 | 0.9 | Severe |
| Great Barrier Reef | -18.95 | 148.08 | 0.4 | 0.3 | 0.3 | 0.2 | 0.5 | Medial |
| Great Barrier Reef | -18.95 | 148.08 | 0.4 | 0.3 | 0.3 | 0.2 | 0.5 | Medial |
| Great Barrier Reef | -18.92 | 147.74 | 0.4 | 0.3 | 0.3 | 0.4 | 0.6 | Medial |
| Great Barrier Reef | -18.91 | 147.74 | 0.4 | 0.3 | 0.3 | 0.4 | 0.6 | Medial |
| Great Barrier Reef | -18.91 | 147.75 | 0.4 | 0.3 | 0.3 | 0.3 | 0.6 | Medial |
| Great Barrier Reef | -18.90 | 147.96 | 0.2 | 0.3 | 0.2 | 0.2 | 0.4 | Medial |
| Great Barrier Reef | -18.90 | 147.96 | 0.2 | 0.3 | 0.2 | 0.2 | 0.4 | Medial |
| Polynesia | -18.90 | 178.10 | 0.4 | 0.4 | 0.4 | 0.1 | 0.5 | Medial |
| Great Barrier Reef | -18.87 | 147.70 | 0.4 | 0.3 | 0.4 | 0.6 | 0.8 | High |
| Polynesia | -18.87 | -159.75 | 0.6 | 0.8 | 0.6 | 0.0 | 0.6 | Medial |
| Great Barrier Reef | -18.87 | 147.72 | 0.3 | 0.3 | 0.3 | 0.4 | 0.6 | Medial |
| Polynesia | -18.86 | -159.80 | 0.6 | 0.8 | 0.6 | 0.2 | 0.6 | Medial |
| Great Barrier Reef | -18.86 | 147.89 | 0.3 | 0.3 | 0.3 | 0.2 | 0.5 | Medial |
| Great Barrier Reef | -18.86 | 147.89 | 0.3 | 0.3 | 0.3 | 0.2 | 0.5 | Medial |
| Great Barrier Reef | -18.84 | 146.54 | 0.5 | 0.2 | 0.4 | 0.8 | 0.9 | Severe |
| Great Barrier Reef | -18.84 | 146.54 | 0.5 | 0.2 | 0.4 | 0.8 | 0.9 | Severe |
| Great Barrier Reef | -18.83 | 146.52 | 0.5 | 0.2 | 0.4 | 0.8 | 0.9 | Severe |
| Great Barrier Reef | -18.83 | 146.53 | 0.5 | 0.2 | 0.4 | 0.8 | 0.9 | Severe |
| Great Barrier Reef | -18.82 | 147.65 | 0.3 | 0.3 | 0.3 | 0.4 | 0.6 | Medial |
| Great Barrier Reef | -18.82 | 147.65 | 0.3 | 0.3 | 0.3 | 0.4 | 0.6 | Medial |
| Great Barrier Reef | -18.81 | 146.43 | 0.5 | 0.2 | 0.4 | 0.8 | 0.9 | Severe |
| Great Barrier Reef | -18.81 | 146.43 | 0.5 | 0.2 | 0.4 | 0.8 | 0.9 | Severe |
| Great Barrier Reef | -18.81 | 146.43 | 0.5 | 0.2 | 0.4 | 0.8 | 0.9 | Severe |
| Great Barrier Reef | -18.80 | 147.53 | 0.4 | 0.3 | 0.4 | 0.5 | 0.7 | High |
| Great Barrier Reef | -18.80 | 147.53 | 0.4 | 0.3 | 0.4 | 0.5 | 0.7 | High |
| Great Barrier Reef | -18.78 | 146.53 | 0.5 | 0.2 | 0.4 | 0.8 | 0.9 | Severe |
| Great Barrier Reef | -18.78 | 146.54 | 0.5 | 0.2 | 0.4 | 0.8 | 0.9 | Severe |
| Great Barrier Reef | -18.78 | 147.87 | 0.4 | 0.3 | 0.4 | 0.2 | 0.5 | Medial |
| Great Barrier Reef | -18.78 | 146.52 | 0.5 | 0.2 | 0.4 | 0.8 | 0.9 | Severe |
| Great Barrier Reef | -18.78 | 146.67 | 0.6 | 0.1 | 0.4 | 0.7 | 0.8 | High |
| Great Barrier Reef | -18.77 | 147.71 | 0.3 | 0.3 | 0.3 | 0.3 | 0.5 | Medial |
| Great Barrier Reef | -18.77 | 146.52 | 0.5 | 0.2 | 0.4 | 0.8 | 0.9 | Severe |
| Great Barrier Reef | -18.77 | 146.55 | 0.5 | 0.2 | 0.4 | 0.8 | 0.9 | Severe |
| Great Barrier Reef | -18.77 | 146.55 | 0.5 | 0.2 | 0.4 | 0.8 | 0.9 | Severe |
| Great Barrier Reef | -18.77 | 146.63 | 0.6 | 0.1 | 0.5 | 0.7 | 0.8 | Severe |
| Great Barrier Reef | -18.77 | 146.55 | 0.5 | 0.2 | 0.4 | 0.8 | 0.9 | Severe |
| Great Barrier Reef | -18.77 | 146.25 | 0.8 | 0.2 | 0.6 | 0.9 | 0.9 | Severe |
| Great Barrier Reef | -18.77 | 146.53 | 0.5 | 0.2 | 0.4 | 0.8 | 0.9 | Severe |
| Polynesia | -18.76 | 178.53 | 0.5 | 0.4 | 0.4 | 0.4 | 0.7 | Medial |
| Polynesia | -18.75 | 178.47 | 0.5 | 0.4 | 0.5 | 0.6 | 0.8 | Severe |
| Great Barrier Reef | -18.75 | 147.27 | 0.5 | 0.3 | 0.4 | 0.3 | 0.6 | Medial |
| Great Barrier Reef | -18.75 | 146.25 | 0.8 | 0.2 | 0.6 | 0.9 | 0.9 | Severe |
| Great Barrier Reef | -18.75 | 147.27 | 0.5 | 0.3 | 0.4 | 0.3 | 0.6 | Medial |
| Great Barrier Reef | -18.74 | 146.69 | 0.4 | 0.2 | 0.4 | 0.5 | 0.7 | High |
| Great Barrier Reef | -18.74 | 146.64 | 0.6 | 0.1 | 0.4 | 0.7 | 0.8 | Severe |
| Great Barrier Reef | -18.73 | 147.27 | 0.5 | 0.3 | 0.4 | 0.3 | 0.6 | Medial |
| Great Barrier Reef | -18.73 | 147.84 | 0.4 | 0.3 | 0.3 | 0.2 | 0.5 | Medial |
| Great Barrier Reef | -18.73 | 147.54 | 0.4 | 0.3 | 0.4 | 0.3 | 0.6 | Medial |
| Great Barrier Reef | -18.72 | 147.54 | 0.4 | 0.3 | 0.4 | 0.3 | 0.6 | Medial |
| Great Barrier Reef | -18.72 | 146.58 | 0.5 | 0.1 | 0.4 | 0.7 | 0.8 | Severe |
| Great Barrier Reef | -18.72 | 146.58 | 0.5 | 0.1 | 0.4 | 0.7 | 0.8 | Severe |
| Great Barrier Reef | -18.70 | 147.10 | 0.6 | 0.3 | 0.5 | 0.4 | 0.7 | High |
| Great Barrier Reef | -18.70 | 147.10 | 0.6 | 0.3 | 0.5 | 0.4 | 0.7 | High |
| Great Barrier Reef | -18.70 | 146.60 | 0.5 | 0.1 | 0.4 | 0.7 | 0.8 | Severe |
| Great Barrier Reef | -18.70 | 147.10 | 0.6 | 0.3 | 0.5 | 0.4 | 0.7 | High |
| Great Barrier Reef | -18.68 | 146.58 | 0.5 | 0.1 | 0.4 | 0.7 | 0.8 | Severe |
| Polynesia | -18.68 | 178.53 | 0.5 | 0.4 | 0.5 | 0.8 | 0.9 | Severe |
| Great Barrier Reef | -18.68 | 146.51 | 0.6 | 0.2 | 0.5 | 0.8 | 0.9 | Severe |
| Great Barrier Reef | -18.68 | 146.51 | 0.6 | 0.2 | 0.5 | 0.8 | 0.9 | Severe |
| Great Barrier Reef | -18.68 | 146.55 | 0.6 | 0.2 | 0.5 | 0.8 | 0.9 | Severe |
| Great Barrier Reef | -18.67 | 146.56 | 0.5 | 0.2 | 0.4 | 0.7 | 0.8 | Severe |
| Great Barrier Reef | -18.67 | 146.56 | 0.5 | 0.2 | 0.4 | 0.7 | 0.8 | Severe |
| Great Barrier Reef | -18.66 | 146.49 | 0.6 | 0.2 | 0.5 | 0.8 | 0.9 | Severe |
| Great Barrier Reef | -18.66 | 146.49 | 0.6 | 0.2 | 0.5 | 0.8 | 0.9 | Severe |
| Great Barrier Reef | -18.66 | 147.71 | 0.4 | 0.3 | 0.4 | 0.3 | 0.6 | Medial |
| Great Barrier Reef | -18.66 | 147.60 | 0.4 | 0.3 | 0.4 | 0.1 | 0.5 | Medial |
| Great Barrier Reef | -18.66 | 146.49 | 0.6 | 0.2 | 0.5 | 0.8 | 0.9 | Severe |
| Great Barrier Reef | -18.66 | 146.48 | 0.6 | 0.2 | 0.5 | 0.8 | 0.9 | Severe |
| Great Barrier Reef | -18.65 | 146.55 | 0.6 | 0.2 | 0.5 | 0.8 | 0.9 | Severe |
| Great Barrier Reef | -18.64 | 147.59 | 0.4 | 0.3 | 0.4 | 0.1 | 0.5 | Medial |
| Great Barrier Reef | -18.64 | 146.50 | 0.6 | 0.2 | 0.5 | 0.8 | 0.9 | Severe |
| Great Barrier Reef | -18.64 | 146.50 | 0.6 | 0.2 | 0.5 | 0.8 | 0.9 | Severe |
| Great Barrier Reef | -18.64 | 146.49 | 0.6 | 0.2 | 0.5 | 0.8 | 0.9 | Severe |
| Great Barrier Reef | -18.64 | 146.49 | 0.6 | 0.2 | 0.5 | 0.8 | 0.9 | Severe |
| Great Barrier Reef | -18.63 | 147.05 | 0.4 | 0.3 | 0.4 | 0.4 | 0.6 | Medial |
| Great Barrier Reef | -18.63 | 147.05 | 0.4 | 0.3 | 0.4 | 0.4 | 0.6 | Medial |
| Great Barrier Reef | -18.63 | 147.05 | 0.5 | 0.3 | 0.4 | 0.4 | 0.7 | High |
| Great Barrier Reef | -18.63 | 147.29 | 0.5 | 0.3 | 0.4 | 0.2 | 0.6 | Medial |
| Great Barrier Reef | -18.62 | 147.05 | 0.5 | 0.3 | 0.4 | 0.4 | 0.7 | High |
| Polynesia | -18.62 | 178.56 | 0.6 | 0.4 | 0.5 | 0.1 | 0.6 | Medial |
| Great Barrier Reef | -18.62 | 147.08 | 0.5 | 0.3 | 0.4 | 0.4 | 0.7 | High |
| Great Barrier Reef | -18.62 | 147.61 | 0.4 | 0.3 | 0.4 | 0.2 | 0.5 | Medial |
| Great Barrier Reef | -18.61 | 146.48 | 0.6 | 0.2 | 0.5 | 0.8 | 0.9 | Severe |
| Great Barrier Reef | -18.60 | 146.49 | 0.6 | 0.2 | 0.5 | 0.8 | 0.9 | Severe |
| Great Barrier Reef | -18.60 | 146.49 | 0.6 | 0.2 | 0.5 | 0.8 | 0.9 | Severe |
| Great Barrier Reef | -18.60 | 146.49 | 0.6 | 0.2 | 0.5 | 0.8 | 0.9 | Severe |
| Polynesia | -18.60 | 178.50 | 0.5 | 0.4 | 0.5 | 0.3 | 0.6 | Medial |
| Great Barrier Reef | -18.59 | 146.50 | 0.6 | 0.2 | 0.5 | 0.8 | 0.9 | Severe |
| Great Barrier Reef | -18.59 | 146.50 | 0.6 | 0.2 | 0.5 | 0.8 | 0.9 | Severe |
| Polynesia | -18.59 | 178.10 | 0.4 | 0.4 | 0.4 | 0.1 | 0.5 | Medial |
| Western Indian Ocean | -18.59 | 43.97 | 0.5 | 0.4 | 0.5 | 0.7 | 0.9 | Severe |
| Western Indian Ocean | -18.59 | 43.93 | 0.5 | 0.4 | 0.5 | 0.8 | 0.9 | Severe |
| Western Indian Ocean | -18.59 | 43.93 | 0.5 | 0.4 | 0.5 | 0.8 | 0.9 | Severe |
| Great Barrier Reef | -18.58 | 146.50 | 0.6 | 0.2 | 0.5 | 0.8 | 0.9 | Severe |
| Western Indian Ocean | -18.57 | 43.89 | 0.5 | 0.4 | 0.5 | 1.0 | 1.0 | Severe |
| Great Barrier Reef | -18.57 | 147.49 | 0.4 | 0.3 | 0.4 | 0.1 | 0.4 | Medial |
| Great Barrier Reef | -18.56 | 147.50 | 0.4 | 0.3 | 0.4 | 0.1 | 0.4 | Medial |
| Great Barrier Reef | -18.56 | 146.49 | 0.6 | 0.2 | 0.5 | 0.8 | 0.9 | Severe |
| Great Barrier Reef | -18.56 | 146.49 | 0.6 | 0.2 | 0.5 | 0.8 | 0.9 | Severe |
| Great Barrier Reef | -18.56 | 146.49 | 0.6 | 0.2 | 0.5 | 0.8 | 0.9 | Severe |
| Western Indian Ocean | -18.55 | 43.86 | 0.5 | 0.4 | 0.5 | 1.0 | 1.0 | Severe |
| Western Indian Ocean | -18.55 | 43.90 | 0.5 | 0.4 | 0.5 | 1.0 | 1.0 | Severe |
| Western Indian Ocean | -18.53 | 43.78 | 0.4 | 0.4 | 0.5 | 0.7 | 0.9 | Severe |
| Great Barrier Reef | -18.53 | 147.14 | 0.5 | 0.3 | 0.4 | 0.3 | 0.6 | Medial |
| Great Barrier Reef | -18.52 | 147.14 | 0.5 | 0.3 | 0.4 | 0.3 | 0.6 | Medial |
| Polynesia | -18.52 | 178.01 | 0.4 | 0.4 | 0.4 | 0.3 | 0.6 | Medial |
| Great Barrier Reef | -18.51 | 147.54 | 0.4 | 0.3 | 0.4 | 0.1 | 0.5 | Medial |
| Great Barrier Reef | -18.51 | 147.54 | 0.4 | 0.3 | 0.4 | 0.1 | 0.5 | Medial |
| Polynesia | -18.50 | 177.90 | 0.5 | 0.5 | 0.5 | 0.5 | 0.7 | High |
| Polynesia | -18.50 | 178.00 | 0.4 | 0.4 | 0.4 | 0.8 | 0.9 | Severe |
| Great Barrier Reef | -18.50 | 147.44 | 0.5 | 0.3 | 0.4 | 0.1 | 0.5 | Medial |
| Western Indian Ocean | -18.49 | 43.81 | 0.5 | 0.4 | 0.5 | 0.7 | 0.9 | Severe |
| Great Barrier Reef | -18.49 | 147.10 | 0.4 | 0.3 | 0.4 | 0.3 | 0.6 | Medial |
| Great Barrier Reef | -18.48 | 146.87 | 0.6 | 0.3 | 0.5 | 0.5 | 0.7 | High |
| Great Barrier Reef | -18.48 | 146.87 | 0.6 | 0.3 | 0.5 | 0.5 | 0.7 | High |
| Great Barrier Reef | -18.48 | 146.87 | 0.6 | 0.3 | 0.5 | 0.5 | 0.7 | High |
| Great Barrier Reef | -18.47 | 146.99 | 0.5 | 0.3 | 0.4 | 0.5 | 0.7 | High |
| Great Barrier Reef | -18.47 | 146.88 | 0.6 | 0.3 | 0.5 | 0.5 | 0.7 | High |
| Great Barrier Reef | -18.47 | 146.99 | 0.5 | 0.3 | 0.4 | 0.5 | 0.7 | High |
| Great Barrier Reef | -18.47 | 146.98 | 0.5 | 0.3 | 0.4 | 0.5 | 0.7 | High |
| Polynesia | -18.46 | 178.10 | 0.5 | 0.5 | 0.5 | 0.5 | 0.7 | High |
| Great Barrier Reef | -18.44 | 147.52 | 0.5 | 0.3 | 0.4 | 0.1 | 0.5 | Medial |
| Great Barrier Reef | -18.43 | 146.99 | 0.5 | 0.3 | 0.5 | 0.5 | 0.7 | High |
| Great Barrier Reef | -18.43 | 146.99 | 0.5 | 0.3 | 0.5 | 0.5 | 0.7 | High |
| Great Barrier Reef | -18.43 | 146.99 | 0.5 | 0.3 | 0.5 | 0.5 | 0.7 | High |
| Polynesia | -18.43 | 178.07 | 0.5 | 0.5 | 0.5 | 0.6 | 0.8 | Severe |
| Polynesia | -18.43 | 178.09 | 0.5 | 0.5 | 0.5 | 0.6 | 0.8 | Severe |
| Great Barrier Reef | -18.42 | 147.35 | 0.5 | 0.3 | 0.4 | 0.1 | 0.5 | Medial |
| Great Barrier Reef | -18.42 | 147.35 | 0.5 | 0.3 | 0.4 | 0.1 | 0.5 | Medial |
| Polynesia | -18.42 | 178.02 | 0.3 | 0.4 | 0.4 | 0.4 | 0.6 | Medial |
| Great Barrier Reef | -18.41 | 147.45 | 0.5 | 0.3 | 0.4 | 0.1 | 0.5 | Medial |
| Great Barrier Reef | -18.41 | 147.45 | 0.5 | 0.3 | 0.4 | 0.1 | 0.5 | Medial |
| Great Barrier Reef | -18.41 | 146.71 | 0.6 | 0.3 | 0.5 | 0.5 | 0.7 | High |
| Great Barrier Reef | -18.40 | 146.83 | 0.5 | 0.3 | 0.4 | 0.5 | 0.7 | High |
| Polynesia | -18.39 | 178.20 | 0.5 | 0.4 | 0.5 | 0.3 | 0.6 | Medial |
| Great Barrier Reef | -18.36 | 147.20 | 0.5 | 0.3 | 0.4 | 0.2 | 0.5 | Medial |
| Great Barrier Reef | -18.35 | 146.83 | 0.5 | 0.3 | 0.4 | 0.5 | 0.7 | High |
| Great Barrier Reef | -18.35 | 146.83 | 0.5 | 0.3 | 0.4 | 0.5 | 0.7 | High |
| Great Barrier Reef | -18.32 | 146.74 | 0.5 | 0.3 | 0.5 | 0.5 | 0.7 | High |
| Great Barrier Reef | -18.32 | 146.74 | 0.5 | 0.3 | 0.5 | 0.5 | 0.7 | High |
| Great Barrier Reef | -18.32 | 146.75 | 0.5 | 0.3 | 0.5 | 0.5 | 0.7 | High |
| Polynesia | -18.29 | 178.08 | 0.6 | 0.5 | 0.5 | 0.6 | 0.8 | High |
| Great Barrier Reef | -18.27 | 147.39 | 0.5 | 0.3 | 0.5 | 0.1 | 0.5 | Medial |
| Great Barrier Reef | -18.27 | 147.39 | 0.5 | 0.3 | 0.5 | 0.1 | 0.5 | Medial |
| Great Barrier Reef | -18.27 | 147.37 | 0.5 | 0.3 | 0.5 | 0.1 | 0.5 | Medial |
| Great Barrier Reef | -18.24 | 146.32 | 0.6 | 0.1 | 0.5 | 0.8 | 0.9 | Severe |
| Great Barrier Reef | -18.23 | 146.77 | 0.5 | 0.3 | 0.4 | 0.5 | 0.7 | High |
| Great Barrier Reef | -18.23 | 146.73 | 0.5 | 0.3 | 0.4 | 0.5 | 0.7 | High |
| Great Barrier Reef | -18.23 | 146.73 | 0.5 | 0.3 | 0.4 | 0.5 | 0.7 | High |
| Great Barrier Reef | -18.23 | 146.73 | 0.5 | 0.3 | 0.4 | 0.5 | 0.7 | High |
| Great Barrier Reef | -18.21 | 147.03 | 0.3 | 0.3 | 0.3 | 0.3 | 0.5 | Medial |
| Polynesia | -18.20 | 178.30 | 0.4 | 0.5 | 0.5 | 1.0 | 1.0 | Severe |
| Great Barrier Reef | -18.17 | 146.15 | 0.5 | 0.2 | 0.4 | 0.9 | 0.9 | Severe |
| Polynesia | -18.16 | 178.40 | 0.6 | ? | 0.5 | 1.0 | 1.0 | Severe |
| Great Barrier Reef | -18.15 | 146.29 | 0.6 | 0.3 | 0.4 | 0.8 | 0.9 | Severe |
| Great Barrier Reef | -18.15 | 146.29 | 0.6 | 0.3 | 0.4 | 0.8 | 0.9 | Severe |
| Polynesia | -18.15 | 178.45 | 0.6 | 0.4 | 0.5 | 1.0 | 1.0 | Severe |
| Great Barrier Reef | -18.15 | 146.29 | 0.6 | 0.3 | 0.5 | 0.8 | 0.9 | Severe |
| Great Barrier Reef | -18.14 | 146.97 | 0.3 | 0.3 | 0.3 | 0.2 | 0.5 | Medial |
| Great Barrier Reef | -18.14 | 146.97 | 0.3 | 0.3 | 0.3 | 0.2 | 0.5 | Medial |
| Great Barrier Reef | -18.12 | 146.76 | 0.5 | 0.3 | 0.4 | 0.5 | 0.7 | High |
| Great Barrier Reef | -18.12 | 146.76 | 0.5 | 0.3 | 0.4 | 0.5 | 0.7 | High |
| Great Barrier Reef | -18.10 | 146.78 | 0.4 | 0.3 | 0.4 | 0.5 | 0.7 | High |
| Great Barrier Reef | -18.10 | 146.80 | 0.4 | 0.3 | 0.4 | 0.5 | 0.7 | High |
| Great Barrier Reef | -18.08 | 146.45 | 0.5 | 0.3 | 0.4 | 0.6 | 0.8 | High |
| Great Barrier Reef | -18.08 | 146.57 | 0.6 | 0.3 | 0.5 | 0.5 | 0.7 | High |
| Great Barrier Reef | -18.08 | 146.80 | 0.4 | 0.3 | 0.4 | 0.5 | 0.7 | High |
| Great Barrier Reef | -18.06 | 146.86 | 0.4 | 0.3 | 0.4 | 0.4 | 0.6 | Medial |
| Great Barrier Reef | -18.05 | 146.56 | 0.6 | 0.3 | 0.5 | 0.5 | 0.8 | High |
| Great Barrier Reef | -18.05 | 146.56 | 0.6 | 0.3 | 0.5 | 0.5 | 0.8 | High |
| Great Barrier Reef | -18.05 | 146.20 | 0.6 | 0.3 | 0.5 | 0.8 | 0.9 | Severe |
| Great Barrier Reef | -18.05 | 146.56 | 0.5 | 0.3 | 0.5 | 0.5 | 0.7 | High |
| Great Barrier Reef | -18.05 | 146.21 | 0.5 | 0.3 | 0.4 | 0.8 | 0.9 | Severe |
| Great Barrier Reef | -18.04 | 146.20 | 0.6 | 0.3 | 0.5 | 0.8 | 0.9 | Severe |
| Great Barrier Reef | -18.04 | 146.79 | 0.5 | 0.3 | 0.4 | 0.4 | 0.6 | Medial |
| Great Barrier Reef | -18.02 | 146.17 | 0.6 | 0.3 | 0.5 | 0.8 | 0.9 | Severe |
| Great Barrier Reef | -18.02 | 146.78 | 0.5 | 0.3 | 0.4 | 0.4 | 0.7 | Medial |
| Great Barrier Reef | -18.01 | 146.84 | 0.4 | 0.3 | 0.3 | 0.3 | 0.6 | Medial |
| Great Barrier Reef | -18.01 | 146.15 | 0.7 | 0.3 | 0.6 | 0.8 | 0.9 | Severe |
| Great Barrier Reef | -18.00 | 146.18 | 0.7 | 0.3 | 0.5 | 0.8 | 0.9 | Severe |
| Great Barrier Reef | -18.00 | 146.17 | 0.7 | 0.3 | 0.5 | 0.8 | 0.9 | Severe |
| Great Barrier Reef | -18.00 | 146.77 | 0.4 | 0.3 | 0.4 | 0.5 | 0.7 | High |
| Great Barrier Reef | -18.00 | 146.78 | 0.4 | 0.3 | 0.4 | 0.5 | 0.7 | High |
| Brazilian province | -17.99 | -38.67 | 0.1 | 0.6 | 0.2 | 0.6 | 0.7 | High |
| Brazilian province | -17.98 | -38.67 | 0.1 | 0.6 | 0.2 | 0.6 | 0.7 | High |
| Brazilian province | -17.98 | -38.70 | 0.1 | 0.6 | 0.2 | 0.6 | 0.7 | High |
| Great Barrier Reef | -17.98 | 146.13 | 0.7 | 0.3 | 0.6 | 0.8 | 0.9 | Severe |
| Brazilian province | -17.97 | -38.66 | 0.1 | 0.6 | 0.2 | 0.6 | 0.7 | High |
| Brazilian province | -17.97 | -38.69 | 0.1 | 0.6 | 0.2 | 0.6 | 0.7 | High |
| Great Barrier Reef | -17.97 | 146.14 | 0.7 | 0.3 | 0.6 | 0.8 | 0.9 | Severe |
| Brazilian province | -17.97 | -38.70 | 0.1 | 0.6 | 0.2 | 0.6 | 0.7 | High |
| Brazilian province | -17.96 | -38.70 | 0.1 | 0.6 | 0.2 | 0.6 | 0.7 | High |
| Brazilian province | -17.96 | -38.70 | 0.1 | 0.6 | 0.2 | 0.6 | 0.7 | High |
| Brazilian province | -17.96 | -38.71 | 0.1 | 0.6 | 0.2 | 0.6 | 0.7 | High |
| Brazilian province | -17.96 | -38.70 | 0.1 | 0.6 | 0.2 | 0.6 | 0.7 | High |
| Brazilian province | -17.96 | -38.70 | 0.1 | 0.6 | 0.2 | 0.6 | 0.7 | High |
| Great Barrier Reef | -17.96 | 146.15 | 0.7 | 0.3 | 0.6 | 0.8 | 0.9 | Severe |
| Brazilian province | -17.96 | -38.65 | 0.1 | 0.6 | 0.2 | 0.6 | 0.7 | High |
| Great Barrier Reef | -17.95 | 146.80 | 0.5 | 0.3 | 0.4 | 0.4 | 0.7 | Medial |
| Brazilian province | -17.94 | -38.66 | 0.1 | 0.6 | 0.2 | 0.6 | 0.7 | High |
| Polynesia | -17.92 | 177.20 | 0.4 | 0.5 | 0.4 | 0.9 | 0.9 | Severe |
| Great Barrier Reef | -17.89 | 146.59 | 0.5 | 0.3 | 0.5 | 0.5 | 0.7 | High |
| Great Barrier Reef | -17.89 | 146.59 | 0.5 | 0.3 | 0.4 | 0.5 | 0.7 | High |
| Great Barrier Reef | -17.88 | 146.59 | 0.5 | 0.3 | 0.4 | 0.5 | 0.7 | High |
| Great Barrier Reef | -17.85 | 146.49 | 0.4 | 0.3 | 0.4 | 0.5 | 0.7 | High |
| Great Barrier Reef | -17.85 | 146.49 | 0.4 | 0.3 | 0.4 | 0.5 | 0.7 | High |
| Great Barrier Reef | -17.85 | 146.49 | 0.4 | 0.3 | 0.4 | 0.5 | 0.7 | High |
| Great Barrier Reef | -17.83 | 146.55 | 0.5 | 0.3 | 0.5 | 0.5 | 0.7 | High |
| Great Barrier Reef | -17.79 | 146.52 | 0.4 | 0.3 | 0.4 | 0.5 | 0.7 | High |
| Great Barrier Reef | -17.79 | 146.52 | 0.4 | 0.3 | 0.4 | 0.5 | 0.7 | High |
| Brazilian province | -17.79 | -39.00 | 0.1 | 0.6 | 0.2 | 0.6 | 0.7 | High |
| Great Barrier Reef | -17.77 | 146.12 | 0.7 | 0.3 | 0.6 | 0.8 | 0.9 | Severe |
| Great Barrier Reef | -17.77 | 146.65 | 0.4 | 0.3 | 0.4 | 0.5 | 0.7 | Medial |
| Great Barrier Reef | -17.77 | 146.44 | 0.5 | 0.3 | 0.4 | 0.5 | 0.7 | High |
| Great Barrier Reef | -17.77 | 146.44 | 0.5 | 0.3 | 0.4 | 0.5 | 0.7 | High |
| Great Barrier Reef | -17.74 | 146.50 | 0.5 | 0.3 | 0.5 | 0.5 | 0.7 | High |
| Polynesia | -17.73 | 177.14 | 0.6 | 0.6 | 0.6 | 0.6 | 0.8 | Severe |
| Great Barrier Reef | -17.73 | 148.46 | 0.4 | 0.3 | 0.4 | 0.1 | 0.5 | Medial |
| Great Barrier Reef | -17.72 | 146.39 | 0.6 | 0.3 | 0.5 | 0.5 | 0.7 | High |
| Great Barrier Reef | -17.72 | 146.39 | 0.6 | 0.3 | 0.5 | 0.5 | 0.7 | High |
| Great Barrier Reef | -17.72 | 146.40 | 0.6 | 0.3 | 0.5 | 0.5 | 0.7 | High |
| Great Barrier Reef | -17.70 | 146.54 | 0.5 | 0.3 | 0.4 | 0.5 | 0.7 | High |
| Polynesia | -17.70 | 177.31 | 0.4 | 0.6 | 0.5 | 0.2 | 0.6 | Medial |
| Great Barrier Reef | -17.69 | 146.57 | 0.5 | 0.3 | 0.5 | 0.5 | 0.7 | High |
| Great Barrier Reef | -17.68 | 146.18 | 0.7 | 0.3 | 0.6 | 0.8 | 0.9 | Severe |
| Great Barrier Reef | -17.68 | 146.17 | 0.7 | 0.3 | 0.6 | 0.8 | 0.9 | Severe |
| Great Barrier Reef | -17.67 | 146.17 | 0.7 | 0.3 | 0.6 | 0.8 | 0.9 | Severe |
| Brazilian province | -17.67 | -38.50 | 0.1 | 0.6 | 0.2 | 0.6 | 0.7 | High |
| Great Barrier Reef | -17.66 | 146.47 | 0.5 | 0.3 | 0.4 | 0.5 | 0.7 | High |
| Great Barrier Reef | -17.66 | 146.47 | 0.5 | 0.3 | 0.4 | 0.5 | 0.7 | High |
| Polynesia | -17.64 | -149.24 | 0.4 | 0.9 | 0.5 | 0.0 | 0.5 | Medial |
| Polynesia | -17.63 | -149.26 | 0.4 | 0.9 | 0.5 | 0.0 | 0.5 | Medial |
| Great Barrier Reef | -17.61 | 146.45 | 0.5 | 0.3 | 0.4 | 0.5 | 0.7 | High |
| Great Barrier Reef | -17.61 | 146.45 | 0.5 | 0.3 | 0.4 | 0.5 | 0.7 | High |
| Polynesia | -17.60 | -149.30 | 0.3 | 0.9 | 0.5 | 0.0 | 0.5 | Medial |
| Polynesia | -17.58 | 178.97 | 0.6 | 0.6 | 0.6 | 0.4 | 0.8 | High |
| Polynesia | -17.58 | -149.78 | 0.2 | 0.9 | 0.4 | 0.0 | 0.4 | Medial |
| Polynesia | -17.57 | 177.30 | 0.3 | 0.6 | 0.4 | 0.2 | 0.6 | Medial |
| Great Barrier Reef | -17.53 | 146.38 | 0.5 | 0.3 | 0.5 | 0.5 | 0.7 | High |
| Great Barrier Reef | -17.53 | 146.38 | 0.5 | 0.3 | 0.5 | 0.5 | 0.7 | High |
| Great Barrier Reef | -17.53 | 146.38 | 0.5 | 0.3 | 0.5 | 0.5 | 0.7 | High |
| Great Barrier Reef | -17.53 | 146.52 | 0.5 | 0.3 | 0.5 | 0.4 | 0.7 | High |
| Great Barrier Reef | -17.52 | 146.53 | 0.5 | 0.3 | 0.5 | 0.4 | 0.7 | High |
| Great Barrier Reef | -17.50 | 147.00 | 0.5 | 0.3 | 0.4 | 0.1 | 0.5 | Medial |
| Great Barrier Reef | -17.49 | 146.39 | 0.5 | 0.3 | 0.4 | 0.5 | 0.7 | High |
| Great Barrier Reef | -17.48 | 146.39 | 0.5 | 0.3 | 0.4 | 0.5 | 0.7 | High |
| Great Barrier Reef | -17.48 | 146.46 | 0.5 | 0.3 | 0.5 | 0.5 | 0.7 | High |
| Polynesia | -17.46 | -149.08 | 0.4 | 0.9 | 0.5 | 0.0 | 0.6 | Medial |
| Great Barrier Reef | -17.43 | 146.54 | 0.6 | 0.3 | 0.5 | 0.4 | 0.7 | High |
| Great Barrier Reef | -17.43 | 146.55 | 0.6 | 0.3 | 0.5 | 0.4 | 0.7 | High |
| Great Barrier Reef | -17.40 | 146.40 | 0.5 | 0.3 | 0.5 | 0.5 | 0.7 | High |
| Great Barrier Reef | -17.38 | 146.42 | 0.5 | 0.3 | 0.5 | 0.5 | 0.7 | High |
| Great Barrier Reef | -17.35 | 146.53 | 0.5 | 0.3 | 0.5 | 0.4 | 0.7 | Medial |
| Polynesia | -17.33 | 179.00 | 0.6 | 0.6 | 0.6 | 0.2 | 0.6 | Medial |
| Polynesia | -17.33 | 178.54 | 0.5 | 0.6 | 0.5 | 0.4 | 0.7 | High |
| Great Barrier Reef | -17.31 | 146.54 | 0.5 | 0.3 | 0.5 | 0.4 | 0.6 | Medial |
| Great Barrier Reef | -17.30 | 146.36 | 0.5 | 0.3 | 0.5 | 0.5 | 0.7 | High |
| Great Barrier Reef | -17.29 | 146.37 | 0.5 | 0.3 | 0.5 | 0.5 | 0.7 | High |
| Great Barrier Reef | -17.27 | 146.47 | 0.5 | 0.3 | 0.5 | 0.4 | 0.7 | High |
| Great Barrier Reef | -17.27 | 146.36 | 0.5 | 0.3 | 0.5 | 0.4 | 0.7 | High |
| Polynesia | -17.25 | 178.57 | 0.5 | 0.6 | 0.6 | 0.5 | 0.8 | High |
| Western Australia | -17.25 | 119.33 | 0.8 | 0.5 | 0.8 | 0.1 | 0.8 | High |
| Great Barrier Reef | -17.23 | 146.48 | 0.5 | 0.3 | 0.4 | 0.3 | 0.6 | Medial |
| Great Barrier Reef | -17.23 | 146.09 | 0.6 | 0.3 | 0.5 | 0.6 | 0.8 | Severe |
| Great Barrier Reef | -17.22 | 146.09 | 0.6 | 0.3 | 0.5 | 0.6 | 0.8 | Severe |
| Great Barrier Reef | -17.22 | 146.08 | 0.6 | 0.3 | 0.5 | 0.6 | 0.8 | Severe |
| Great Barrier Reef | -17.21 | 146.08 | 0.6 | 0.3 | 0.5 | 0.6 | 0.8 | Severe |
| Great Barrier Reef | -17.21 | 146.08 | 0.6 | 0.3 | 0.5 | 0.6 | 0.8 | Severe |
| Great Barrier Reef | -17.20 | 146.37 | 0.5 | 0.3 | 0.5 | 0.4 | 0.7 | High |
| Great Barrier Reef | -17.20 | 146.37 | 0.5 | 0.3 | 0.5 | 0.4 | 0.7 | High |
| Great Barrier Reef | -17.19 | 146.29 | 0.5 | 0.3 | 0.5 | 0.5 | 0.7 | High |
| Great Barrier Reef | -17.18 | 146.29 | 0.5 | 0.3 | 0.5 | 0.5 | 0.7 | High |
| Great Barrier Reef | -17.15 | 146.01 | 0.5 | 0.3 | 0.5 | 0.8 | 0.9 | Severe |
| Polynesia | -17.13 | 178.27 | 0.6 | 0.6 | 0.6 | 0.5 | 0.8 | High |
| Great Barrier Reef | -17.10 | 146.34 | 0.6 | 0.3 | 0.5 | 0.4 | 0.7 | High |
| Great Barrier Reef | -17.07 | 146.18 | 0.6 | 0.3 | 0.5 | 0.5 | 0.7 | High |
| Great Barrier Reef | -17.07 | 146.19 | 0.6 | 0.3 | 0.5 | 0.5 | 0.7 | High |
| Great Barrier Reef | -17.02 | 146.22 | 0.5 | 0.3 | 0.4 | 0.4 | 0.7 | High |
| Great Barrier Reef | -17.01 | 146.22 | 0.5 | 0.3 | 0.4 | 0.4 | 0.7 | High |
| Polynesia | -17.00 | -151.00 | 0.5 | 0.9 | 0.6 | 0.0 | 0.6 | Medial |
| Polynesia | -17.00 | -143.00 | 0.3 | 0.8 | 0.5 | 0.0 | 0.5 | Medial |
| Western Indian Ocean | -17.00 | 43.50 | 0.5 | 0.4 | 0.5 | 0.4 | 0.7 | High |
| Great Barrier Reef | -17.00 | 146.00 | 0.5 | 0.3 | 0.4 | 0.7 | 0.9 | Severe |
| Polynesia | -17.00 | 178.00 | 0.6 | 0.6 | 0.6 | 0.2 | 0.7 | High |
| Great Barrier Reef | -16.95 | 146.22 | 0.5 | 0.3 | 0.5 | 0.4 | 0.7 | High |
| Great Barrier Reef | -16.94 | 146.21 | 0.5 | 0.3 | 0.5 | 0.4 | 0.7 | High |
| Great Barrier Reef | -16.94 | 146.21 | 0.5 | 0.3 | 0.5 | 0.4 | 0.7 | High |
| Great Barrier Reef | -16.92 | 146.00 | 0.5 | 0.3 | 0.5 | 0.6 | 0.8 | High |
| Great Barrier Reef | -16.92 | 146.00 | 0.5 | 0.3 | 0.5 | 0.6 | 0.8 | High |
| Great Barrier Reef | -16.92 | 145.99 | 0.5 | 0.3 | 0.5 | 0.6 | 0.8 | High |
| Great Barrier Reef | -16.92 | 146.25 | 0.5 | 0.3 | 0.5 | 0.4 | 0.7 | High |
| Great Barrier Reef | -16.92 | 146.25 | 0.5 | 0.3 | 0.5 | 0.4 | 0.7 | High |
| Great Barrier Reef | -16.92 | 146.25 | 0.5 | 0.3 | 0.5 | 0.4 | 0.7 | High |
| Great Barrier Reef | -16.88 | 146.40 | 0.5 | 0.3 | 0.4 | 0.2 | 0.6 | Medial |
| Great Barrier Reef | -16.87 | 146.21 | 0.5 | 0.3 | 0.4 | 0.4 | 0.7 | High |
| Great Barrier Reef | -16.87 | 146.21 | 0.5 | 0.3 | 0.4 | 0.4 | 0.7 | High |
| Great Barrier Reef | -16.87 | 146.22 | 0.5 | 0.3 | 0.4 | 0.4 | 0.7 | High |
| Great Barrier Reef | -16.80 | 146.18 | 0.5 | 0.3 | 0.4 | 0.5 | 0.7 | High |
| Great Barrier Reef | -16.80 | 146.18 | 0.5 | 0.3 | 0.4 | 0.5 | 0.7 | High |
| Great Barrier Reef | -16.79 | 146.27 | 0.4 | 0.3 | 0.5 | 0.4 | 0.7 | Medial |
| Great Barrier Reef | -16.79 | 146.27 | 0.4 | 0.3 | 0.5 | 0.4 | 0.7 | Medial |
| Great Barrier Reef | -16.77 | 145.98 | 0.5 | 0.3 | 0.5 | 0.5 | 0.7 | High |
| Great Barrier Reef | -16.77 | 145.99 | 0.6 | 0.3 | 0.5 | 0.5 | 0.7 | High |
| Great Barrier Reef | -16.77 | 145.99 | 0.6 | 0.3 | 0.5 | 0.5 | 0.7 | High |
| Great Barrier Reef | -16.77 | 145.99 | 0.6 | 0.3 | 0.5 | 0.5 | 0.7 | High |
| Great Barrier Reef | -16.76 | 147.73 | 0.5 | 0.4 | 0.5 | 0.1 | 0.5 | Medial |
| Western Indian Ocean | -16.74 | 63.40 | 0.3 | 0.4 | 0.4 | 0.0 | 0.4 | Medial |
| Great Barrier Reef | -16.74 | 146.27 | 0.5 | 0.3 | 0.5 | 0.3 | 0.6 | Medial |
| Great Barrier Reef | -16.73 | 145.68 | 0.6 | 0.3 | 0.5 | 0.6 | 0.8 | High |
| Great Barrier Reef | -16.71 | 146.01 | 0.6 | 0.3 | 0.5 | 0.4 | 0.7 | High |
| Great Barrier Reef | -16.70 | 146.05 | 0.6 | 0.3 | 0.5 | 0.3 | 0.7 | High |
| Great Barrier Reef | -16.70 | 146.05 | 0.6 | 0.3 | 0.5 | 0.3 | 0.7 | High |
| Great Barrier Reef | -16.69 | 146.25 | 0.4 | 0.3 | 0.4 | 0.2 | 0.5 | Medial |
| Great Barrier Reef | -16.67 | 145.94 | 0.6 | 0.3 | 0.5 | 0.5 | 0.7 | High |
| Great Barrier Reef | -16.67 | 145.94 | 0.6 | 0.3 | 0.5 | 0.5 | 0.7 | High |
| Great Barrier Reef | -16.65 | 145.99 | 0.5 | 0.3 | 0.5 | 0.4 | 0.7 | Medial |
| Great Barrier Reef | -16.65 | 146.03 | 0.5 | 0.3 | 0.5 | 0.3 | 0.6 | Medial |
| Great Barrier Reef | -16.65 | 145.98 | 0.4 | 0.3 | 0.4 | 0.4 | 0.6 | Medial |
| Great Barrier Reef | -16.64 | 146.02 | 0.5 | 0.3 | 0.5 | 0.4 | 0.7 | Medial |
| Great Barrier Reef | -16.64 | 145.93 | 0.5 | 0.3 | 0.4 | 0.5 | 0.7 | High |
| Great Barrier Reef | -16.62 | 146.04 | 0.5 | 0.3 | 0.5 | 0.3 | 0.6 | Medial |
| Great Barrier Reef | -16.62 | 145.95 | 0.4 | 0.3 | 0.4 | 0.4 | 0.6 | Medial |
| Great Barrier Reef | -16.58 | 146.02 | 0.5 | 0.3 | 0.4 | 0.4 | 0.6 | Medial |
| Great Barrier Reef | -16.58 | 145.98 | 0.5 | 0.3 | 0.4 | 0.4 | 0.6 | Medial |
| Great Barrier Reef | -16.57 | 146.00 | 0.5 | 0.3 | 0.4 | 0.3 | 0.6 | Medial |
| Great Barrier Reef | -16.56 | 145.55 | 0.7 | 0.3 | 0.6 | 0.7 | 0.9 | Severe |
| Great Barrier Reef | -16.55 | 146.05 | 0.5 | 0.3 | 0.4 | 0.3 | 0.6 | Medial |
| Great Barrier Reef | -16.55 | 145.95 | 0.5 | 0.3 | 0.5 | 0.4 | 0.7 | High |
| Western Indian Ocean | -16.52 | 44.00 | 0.5 | 0.4 | 0.6 | 0.6 | 0.8 | Severe |
| Great Barrier Reef | -16.52 | 146.01 | 0.5 | 0.3 | 0.4 | 0.3 | 0.6 | Medial |
| Great Barrier Reef | -16.50 | 146.03 | 0.5 | 0.3 | 0.4 | 0.3 | 0.6 | Medial |
| Great Barrier Reef | -16.47 | 145.80 | 0.7 | 0.3 | 0.6 | 0.5 | 0.8 | High |
| Great Barrier Reef | -16.47 | 145.99 | 0.6 | 0.3 | 0.5 | 0.3 | 0.6 | Medial |
| Western Indian Ocean | -16.44 | 39.96 | 0.5 | 0.5 | 0.5 | 0.8 | 0.9 | Severe |
| Great Barrier Reef | -16.43 | 146.00 | 0.5 | 0.3 | 0.5 | 0.3 | 0.6 | Medial |
| Great Barrier Reef | -16.42 | 145.77 | 0.7 | 0.3 | 0.5 | 0.5 | 0.8 | High |
| Great Barrier Reef | -16.41 | 145.77 | 0.6 | 0.3 | 0.5 | 0.5 | 0.7 | High |
| Great Barrier Reef | -16.40 | 145.70 | 0.6 | 0.3 | 0.5 | 0.5 | 0.8 | High |
| Great Barrier Reef | -16.39 | 145.57 | 0.5 | 0.3 | 0.5 | 0.7 | 0.8 | High |
| Great Barrier Reef | -16.39 | 145.56 | 0.5 | 0.3 | 0.5 | 0.7 | 0.8 | High |
| Great Barrier Reef | -16.39 | 145.56 | 0.5 | 0.3 | 0.5 | 0.7 | 0.8 | High |
| Western Indian Ocean | -16.33 | 40.00 | 0.3 | 0.5 | 0.4 | 0.8 | 0.9 | Severe |
| Western Indian Ocean | -16.33 | 49.85 | 0.6 | 0.4 | 0.5 | 0.6 | 0.8 | High |
| Great Barrier Reef | -16.32 | 145.81 | 0.6 | 0.3 | 0.6 | 0.4 | 0.7 | High |
| Great Barrier Reef | -16.30 | 145.50 | 0.8 | 0.3 | 0.6 | 0.7 | 0.9 | Severe |
| Great Barrier Reef | -16.30 | 145.68 | 0.6 | 0.3 | 0.5 | 0.5 | 0.7 | High |
| Great Barrier Reef | -16.25 | 145.49 | 0.7 | 0.3 | 0.6 | 0.8 | 0.9 | Severe |
| Polynesia | -16.24 | 179.30 | 0.5 | 0.7 | 0.6 | 0.3 | 0.7 | High |
| Great Barrier Reef | -16.22 | 145.89 | 0.6 | 0.3 | 0.5 | 0.3 | 0.7 | High |
| Great Barrier Reef | -16.21 | 145.79 | 0.5 | 0.3 | 0.5 | 0.3 | 0.7 | High |
| Great Barrier Reef | -16.21 | 145.91 | 0.6 | 0.3 | 0.5 | 0.3 | 0.6 | Medial |
| Western Indian Ocean | -16.20 | 40.14 | 0.3 | 0.5 | 0.5 | 0.8 | 0.9 | Severe |
| Great Barrier Reef | -16.19 | 145.70 | 0.6 | 0.3 | 0.5 | 0.4 | 0.7 | High |
| Great Barrier Reef | -16.19 | 145.70 | 0.6 | 0.3 | 0.5 | 0.4 | 0.7 | High |
| Great Barrier Reef | -16.13 | 145.67 | 0.7 | 0.3 | 0.6 | 0.4 | 0.8 | High |
| Great Barrier Reef | -16.12 | 145.69 | 0.6 | 0.3 | 0.5 | 0.4 | 0.7 | High |
| Great Barrier Reef | -16.12 | 145.69 | 0.6 | 0.3 | 0.5 | 0.4 | 0.7 | High |
| Great Barrier Reef | -16.12 | 145.78 | 0.6 | 0.3 | 0.5 | 0.3 | 0.7 | Medial |
| Great Barrier Reef | -16.12 | 145.78 | 0.6 | 0.3 | 0.5 | 0.3 | 0.7 | Medial |
| Great Barrier Reef | -16.11 | 145.84 | 0.5 | 0.3 | 0.4 | 0.3 | 0.6 | Medial |
| Great Barrier Reef | -16.10 | 145.47 | 0.6 | 0.3 | 0.5 | 0.7 | 0.9 | Severe |
| Great Barrier Reef | -16.10 | 167.43 | 0.7 | 0.6 | 0.7 | 0.1 | 0.7 | High |
| Great Barrier Reef | -16.08 | 145.72 | 0.6 | 0.3 | 0.5 | 0.3 | 0.7 | High |
| Great Barrier Reef | -16.08 | 145.76 | 0.6 | 0.3 | 0.5 | 0.3 | 0.7 | High |
| Great Barrier Reef | -16.07 | 145.85 | 0.5 | 0.3 | 0.5 | 0.3 | 0.6 | Medial |
| Western Indian Ocean | -16.07 | 50.18 | 0.4 | 0.4 | 0.4 | 0.7 | 0.9 | Severe |
| Great Barrier Reef | -16.05 | 145.86 | 0.5 | 0.3 | 0.4 | 0.2 | 0.6 | Medial |
| Great Barrier Reef | -16.05 | 145.65 | 0.5 | 0.3 | 0.5 | 0.4 | 0.7 | High |
| Great Barrier Reef | -16.04 | 145.65 | 0.6 | 0.3 | 0.5 | 0.4 | 0.7 | High |
| Great Barrier Reef | -16.04 | 145.87 | 0.5 | 0.3 | 0.5 | 0.2 | 0.6 | Medial |
| Great Barrier Reef | -16.03 | 145.71 | 0.5 | 0.3 | 0.4 | 0.3 | 0.6 | Medial |
| Great Barrier Reef | -16.03 | 145.71 | 0.5 | 0.3 | 0.4 | 0.3 | 0.6 | Medial |
| Great Barrier Reef | -16.02 | 145.85 | 0.5 | 0.3 | 0.5 | 0.3 | 0.6 | Medial |
| Great Barrier Reef | -16.02 | 145.70 | 0.5 | 0.3 | 0.4 | 0.3 | 0.6 | Medial |
| Western Indian Ocean | -16.02 | 40.13 | 0.6 | 0.5 | 0.6 | 0.8 | 0.9 | Severe |
| Western Indian Ocean | -16.01 | 50.16 | 0.5 | 0.4 | 0.5 | 0.9 | 0.9 | Severe |
| Western Indian Ocean | -16.00 | 50.20 | 0.6 | 0.4 | 0.5 | 0.9 | 0.9 | Severe |
| Western Indian Ocean | -16.00 | 50.16 | 0.5 | 0.4 | 0.5 | 0.9 | 0.9 | Severe |
| Western Indian Ocean | -16.00 | 50.50 | 0.4 | 0.4 | 0.4 | 0.1 | 0.5 | Medial |
| Polynesia | -16.00 | 178.00 | 0.6 | 0.7 | 0.7 | 0.0 | 0.7 | Medial |
| Great Barrier Reef | -15.99 | 145.83 | 0.4 | 0.3 | 0.4 | 0.2 | 0.6 | Medial |
| Western Indian Ocean | -15.98 | 50.17 | 0.6 | 0.4 | 0.6 | 0.9 | 1.0 | Severe |
| Great Barrier Reef | -15.97 | 145.67 | 0.6 | 0.3 | 0.5 | 0.4 | 0.7 | High |
| Great Barrier Reef | -15.95 | 145.82 | 0.5 | 0.3 | 0.5 | 0.2 | 0.6 | Medial |
| Great Barrier Reef | -15.94 | 145.40 | 0.7 | 0.3 | 0.6 | 0.6 | 0.8 | Severe |
| Great Barrier Reef | -15.91 | 145.66 | 0.6 | 0.3 | 0.5 | 0.3 | 0.7 | High |
| Great Barrier Reef | -15.91 | 145.66 | 0.6 | 0.3 | 0.5 | 0.3 | 0.7 | High |
| Great Barrier Reef | -15.88 | 145.79 | 0.6 | 0.3 | 0.5 | 0.2 | 0.7 | Medial |
| Great Barrier Reef | -15.88 | 145.79 | 0.6 | 0.3 | 0.5 | 0.2 | 0.7 | Medial |
| Great Barrier Reef | -15.88 | 145.57 | 0.6 | 0.3 | 0.5 | 0.4 | 0.7 | High |
| Great Barrier Reef | -15.87 | 145.57 | 0.6 | 0.3 | 0.5 | 0.4 | 0.7 | High |
| Great Barrier Reef | -15.81 | 145.78 | 0.5 | 0.3 | 0.5 | 0.2 | 0.6 | Medial |
| Great Barrier Reef | -15.79 | 145.80 | 0.5 | 0.3 | 0.5 | 0.2 | 0.6 | Medial |
| Great Barrier Reef | -15.79 | 145.80 | 0.5 | 0.3 | 0.5 | 0.2 | 0.6 | Medial |
| Great Barrier Reef | -15.78 | 145.58 | 0.6 | 0.3 | 0.5 | 0.4 | 0.7 | High |
| Great Barrier Reef | -15.78 | 145.58 | 0.6 | 0.3 | 0.5 | 0.4 | 0.7 | High |
| Great Barrier Reef | -15.78 | 145.58 | 0.6 | 0.3 | 0.5 | 0.4 | 0.7 | High |
| Great Barrier Reef | -15.75 | 145.78 | 0.5 | 0.4 | 0.5 | 0.2 | 0.6 | Medial |
| Great Barrier Reef | -15.75 | 145.78 | 0.5 | 0.4 | 0.5 | 0.2 | 0.6 | Medial |
| Great Barrier Reef | -15.74 | 145.44 | 0.6 | 0.3 | 0.6 | 0.5 | 0.8 | High |
| Great Barrier Reef | -15.74 | 145.46 | 0.6 | 0.3 | 0.6 | 0.5 | 0.8 | High |
| Great Barrier Reef | -15.72 | 145.80 | 0.4 | 0.4 | 0.4 | 0.2 | 0.5 | Medial |
| Great Barrier Reef | -15.71 | 145.39 | 0.7 | 0.3 | 0.6 | 0.5 | 0.8 | High |
| Great Barrier Reef | -15.69 | 145.50 | 0.6 | 0.3 | 0.5 | 0.4 | 0.7 | High |
| Great Barrier Reef | -15.65 | 145.81 | 0.4 | 0.4 | 0.4 | 0.2 | 0.6 | Medial |
| Great Barrier Reef | -15.65 | 145.72 | 0.5 | 0.4 | 0.5 | 0.2 | 0.6 | Medial |
| Great Barrier Reef | -15.63 | 168.12 | 0.6 | 0.7 | 0.6 | 0.1 | 0.6 | Medial |
| Great Barrier Reef | -15.61 | 145.51 | 0.4 | 0.3 | 0.5 | 0.3 | 0.6 | Medial |
| Great Barrier Reef | -15.61 | 145.63 | 0.5 | 0.4 | 0.4 | 0.2 | 0.6 | Medial |
| Great Barrier Reef | -15.61 | 145.63 | 0.5 | 0.4 | 0.4 | 0.2 | 0.6 | Medial |
| Great Barrier Reef | -15.61 | 145.80 | 0.4 | 0.4 | 0.4 | 0.2 | 0.5 | Medial |
| Great Barrier Reef | -15.58 | 145.78 | 0.5 | 0.4 | 0.5 | 0.2 | 0.6 | Medial |
| Great Barrier Reef | -15.56 | 145.65 | 0.4 | 0.3 | 0.4 | 0.2 | 0.6 | Medial |
| Great Barrier Reef | -15.56 | 145.65 | 0.4 | 0.3 | 0.4 | 0.2 | 0.6 | Medial |
| Great Barrier Reef | -15.55 | 145.53 | 0.5 | 0.3 | 0.4 | 0.2 | 0.6 | Medial |
| Great Barrier Reef | -15.53 | 145.35 | 0.7 | 0.3 | 0.6 | 0.5 | 0.8 | High |
| Great Barrier Reef | -15.51 | 145.54 | 0.5 | 0.3 | 0.4 | 0.2 | 0.6 | Medial |
| Great Barrier Reef | -15.51 | 145.55 | 0.5 | 0.4 | 0.4 | 0.2 | 0.6 | Medial |
| Great Barrier Reef | -15.50 | 145.62 | 0.4 | 0.4 | 0.4 | 0.2 | 0.6 | Medial |
| Great Barrier Reef | -15.50 | 145.62 | 0.4 | 0.4 | 0.4 | 0.2 | 0.6 | Medial |
| Great Barrier Reef | -15.50 | 145.33 | 0.7 | 0.3 | 0.6 | 0.5 | 0.8 | High |
| Great Barrier Reef | -15.50 | 145.62 | 0.4 | 0.4 | 0.4 | 0.2 | 0.6 | Medial |
| Great Barrier Reef | -15.49 | 145.80 | 0.4 | 0.4 | 0.4 | 0.2 | 0.5 | Medial |
| Great Barrier Reef | -15.49 | 145.65 | 0.4 | 0.4 | 0.4 | 0.2 | 0.6 | Medial |
| Great Barrier Reef | -15.49 | 145.66 | 0.4 | 0.4 | 0.4 | 0.2 | 0.6 | Medial |
| Great Barrier Reef | -15.47 | 145.42 | 0.4 | 0.3 | 0.4 | 0.3 | 0.6 | Medial |
| Great Barrier Reef | -15.47 | 145.42 | 0.4 | 0.3 | 0.4 | 0.3 | 0.6 | Medial |
| Great Barrier Reef | -15.46 | 145.68 | 0.4 | 0.4 | 0.4 | 0.2 | 0.6 | Medial |
| Great Barrier Reef | -15.46 | 145.68 | 0.4 | 0.4 | 0.4 | 0.2 | 0.6 | Medial |
| Great Barrier Reef | -15.43 | 145.80 | 0.3 | 0.4 | 0.4 | 0.2 | 0.5 | Medial |
| Great Barrier Reef | -15.43 | 145.51 | 0.5 | 0.3 | 0.5 | 0.2 | 0.6 | Medial |
| Great Barrier Reef | -15.43 | 145.61 | 0.4 | 0.4 | 0.4 | 0.2 | 0.6 | Medial |
| Great Barrier Reef | -15.43 | 145.51 | 0.5 | 0.3 | 0.5 | 0.2 | 0.6 | Medial |
| Great Barrier Reef | -15.41 | 145.43 | 0.4 | 0.3 | 0.5 | 0.3 | 0.6 | Medial |
| Great Barrier Reef | -15.41 | 145.43 | 0.4 | 0.3 | 0.5 | 0.3 | 0.6 | Medial |
| Great Barrier Reef | -15.37 | 145.78 | 0.4 | 0.4 | 0.4 | 0.2 | 0.5 | Medial |
| Great Barrier Reef | -15.37 | 145.78 | 0.4 | 0.4 | 0.4 | 0.2 | 0.5 | Medial |
| Great Barrier Reef | -15.36 | 145.58 | 0.6 | 0.4 | 0.5 | 0.2 | 0.6 | Medial |
| Great Barrier Reef | -15.36 | 145.59 | 0.6 | 0.4 | 0.5 | 0.2 | 0.6 | Medial |
| Great Barrier Reef | -15.33 | 145.65 | 0.5 | 0.4 | 0.5 | 0.2 | 0.6 | Medial |
| Great Barrier Reef | -15.32 | 145.64 | 0.5 | 0.4 | 0.5 | 0.2 | 0.6 | Medial |
| Great Barrier Reef | -15.31 | 145.76 | 0.4 | 0.4 | 0.4 | 0.2 | 0.6 | Medial |
| Great Barrier Reef | -15.29 | 145.58 | 0.6 | 0.4 | 0.6 | 0.2 | 0.7 | Medial |
| Great Barrier Reef | -15.28 | 145.75 | 0.5 | 0.4 | 0.5 | 0.2 | 0.6 | Medial |
| Great Barrier Reef | -15.28 | 145.75 | 0.5 | 0.4 | 0.5 | 0.2 | 0.6 | Medial |
| Great Barrier Reef | -15.25 | 145.58 | 0.6 | 0.4 | 0.6 | 0.2 | 0.7 | Medial |
| Great Barrier Reef | -15.24 | 145.54 | 0.6 | 0.4 | 0.5 | 0.2 | 0.7 | Medial |
| Great Barrier Reef | -15.24 | 145.54 | 0.6 | 0.4 | 0.5 | 0.2 | 0.7 | Medial |
| Great Barrier Reef | -15.24 | 145.72 | 0.4 | 0.4 | 0.5 | 0.2 | 0.6 | Medial |
| Great Barrier Reef | -15.21 | 145.56 | 0.6 | 0.4 | 0.5 | 0.2 | 0.6 | Medial |
| Great Barrier Reef | -15.21 | 145.56 | 0.6 | 0.4 | 0.5 | 0.2 | 0.6 | Medial |
| Great Barrier Reef | -15.21 | 145.56 | 0.6 | 0.4 | 0.5 | 0.2 | 0.6 | Medial |
| Great Barrier Reef | -15.20 | 145.74 | 0.4 | 0.4 | 0.4 | 0.2 | 0.5 | Medial |
| Polynesia | -15.20 | -147.70 | 0.5 | 0.9 | 0.6 | 0.0 | 0.6 | Medial |
| Great Barrier Reef | -15.20 | 145.62 | 0.6 | 0.4 | 0.5 | 0.2 | 0.6 | Medial |
| Polynesia | -15.17 | -147.67 | 0.5 | 0.9 | 0.6 | 0.0 | 0.6 | Medial |
| Great Barrier Reef | -15.17 | 145.50 | 0.6 | 0.4 | 0.6 | 0.2 | 0.7 | Medial |
| Great Barrier Reef | -15.16 | 145.27 | 0.8 | 0.3 | 0.6 | 0.3 | 0.7 | High |
| Great Barrier Reef | -15.14 | 145.58 | 0.6 | 0.4 | 0.5 | 0.2 | 0.6 | Medial |
| Great Barrier Reef | -15.14 | 145.58 | 0.6 | 0.4 | 0.5 | 0.2 | 0.6 | Medial |
| Great Barrier Reef | -15.14 | 145.32 | 0.7 | 0.3 | 0.6 | 0.3 | 0.7 | High |
| Great Barrier Reef | -15.14 | 145.70 | 0.5 | 0.4 | 0.5 | 0.2 | 0.6 | Medial |
| Great Barrier Reef | -15.11 | 145.42 | 0.6 | 0.3 | 0.5 | 0.2 | 0.6 | Medial |
| Great Barrier Reef | -15.10 | 145.38 | 0.6 | 0.3 | 0.6 | 0.2 | 0.7 | High |
| Great Barrier Reef | -15.08 | 145.72 | 0.5 | 0.4 | 0.5 | 0.2 | 0.6 | Medial |
| Great Barrier Reef | -15.08 | 145.72 | 0.5 | 0.4 | 0.5 | 0.2 | 0.6 | Medial |
| Great Barrier Reef | -15.05 | 145.57 | 0.6 | 0.4 | 0.5 | 0.2 | 0.6 | Medial |
| Great Barrier Reef | -15.05 | 145.53 | 0.4 | 0.4 | 0.4 | 0.2 | 0.6 | Medial |
| Great Barrier Reef | -15.05 | 145.57 | 0.6 | 0.4 | 0.5 | 0.2 | 0.6 | Medial |
| Great Barrier Reef | -15.02 | 145.44 | 0.6 | 0.4 | 0.5 | 0.2 | 0.6 | Medial |
| Great Barrier Reef | -15.02 | 145.44 | 0.6 | 0.4 | 0.5 | 0.2 | 0.6 | Medial |
| Great Barrier Reef | -15.01 | 145.57 | 0.6 | 0.4 | 0.5 | 0.2 | 0.6 | Medial |
| Polynesia | -15.00 | -148.17 | 0.5 | 1.0 | 0.6 | 0.0 | 0.6 | Medial |
| Polynesia | -15.00 | -147.60 | 0.5 | 0.9 | 0.6 | 0.0 | 0.6 | Medial |
| Western Indian Ocean | -15.00 | 50.50 | 0.3 | 0.4 | 0.4 | 0.3 | 0.6 | Medial |
| Great Barrier Reef | -14.99 | 145.70 | 0.4 | 0.4 | 0.4 | 0.2 | 0.5 | Medial |
| Great Barrier Reef | -14.99 | 145.70 | 0.4 | 0.4 | 0.4 | 0.2 | 0.5 | Medial |
| Great Barrier Reef | -14.99 | 145.70 | 0.4 | 0.4 | 0.4 | 0.2 | 0.5 | Medial |
| Polynesia | -14.99 | -147.60 | 0.5 | 0.9 | 0.6 | 0.0 | 0.6 | Medial |
| Polynesia | -14.95 | -147.80 | 0.6 | 0.9 | 0.7 | 0.0 | 0.8 | High |
| Great Barrier Reef | -14.94 | 145.48 | 0.5 | 0.4 | 0.5 | 0.2 | 0.6 | Medial |
| Great Barrier Reef | -14.94 | 145.49 | 0.5 | 0.4 | 0.5 | 0.2 | 0.6 | Medial |
| Great Barrier Reef | -14.93 | 145.68 | 0.5 | 0.4 | 0.5 | 0.2 | 0.6 | Medial |
| Great Barrier Reef | -14.92 | 145.69 | 0.5 | 0.4 | 0.5 | 0.2 | 0.6 | Medial |
| Great Barrier Reef | -14.92 | 145.51 | 0.4 | 0.4 | 0.4 | 0.2 | 0.5 | Medial |
| Great Barrier Reef | -14.90 | 145.48 | 0.6 | 0.4 | 0.5 | 0.2 | 0.6 | Medial |
| Great Barrier Reef | -14.88 | 145.56 | 0.4 | 0.4 | 0.5 | 0.2 | 0.6 | Medial |
| Great Barrier Reef | -14.88 | 145.56 | 0.4 | 0.4 | 0.5 | 0.2 | 0.6 | Medial |
| Great Barrier Reef | -14.87 | 145.53 | 0.6 | 0.4 | 0.6 | 0.2 | 0.7 | Medial |
| Great Barrier Reef | -14.85 | 145.48 | 0.6 | 0.4 | 0.5 | 0.2 | 0.6 | Medial |
| Great Barrier Reef | -14.85 | 145.27 | 0.6 | 0.4 | 0.5 | 0.3 | 0.7 | High |
| Great Barrier Reef | -14.85 | 145.27 | 0.6 | 0.4 | 0.5 | 0.3 | 0.7 | High |
| Great Barrier Reef | -14.85 | 145.27 | 0.6 | 0.4 | 0.5 | 0.3 | 0.7 | High |
| Great Barrier Reef | -14.85 | 145.27 | 0.6 | 0.4 | 0.5 | 0.3 | 0.7 | High |
| Great Barrier Reef | -14.83 | 145.70 | 0.4 | 0.4 | 0.5 | 0.2 | 0.6 | Medial |
| Great Barrier Reef | -14.82 | 145.52 | 0.6 | 0.4 | 0.6 | 0.2 | 0.7 | Medial |
| Great Barrier Reef | -14.82 | 145.52 | 0.6 | 0.4 | 0.6 | 0.2 | 0.7 | Medial |
| Great Barrier Reef | -14.81 | 145.52 | 0.6 | 0.4 | 0.6 | 0.2 | 0.7 | Medial |
| Great Barrier Reef | -14.81 | 145.52 | 0.6 | 0.4 | 0.6 | 0.2 | 0.7 | Medial |
| Great Barrier Reef | -14.80 | 145.72 | 0.5 | 0.4 | 0.5 | 0.2 | 0.6 | Medial |
| Great Barrier Reef | -14.79 | 145.59 | 0.5 | 0.4 | 0.5 | 0.2 | 0.6 | Medial |
| Great Barrier Reef | -14.79 | 145.35 | 0.5 | 0.4 | 0.5 | 0.2 | 0.6 | Medial |
| Great Barrier Reef | -14.76 | 145.38 | 0.6 | 0.4 | 0.5 | 0.2 | 0.6 | Medial |
| South East Asia | -14.75 | 99.26 | 0.5 | 0.8 | 0.6 | 0.1 | 0.6 | Medial |
| Great Barrier Reef | -14.75 | 145.52 | 0.6 | 0.4 | 0.6 | 0.2 | 0.7 | High |
| Great Barrier Reef | -14.74 | 145.51 | 0.6 | 0.4 | 0.6 | 0.2 | 0.7 | High |
| Great Barrier Reef | -14.74 | 145.51 | 0.6 | 0.4 | 0.6 | 0.2 | 0.7 | High |
| Great Barrier Reef | -14.74 | 145.51 | 0.6 | 0.4 | 0.6 | 0.2 | 0.7 | High |
| Great Barrier Reef | -14.74 | 145.08 | 0.7 | 0.4 | 0.6 | 0.3 | 0.7 | High |
| Great Barrier Reef | -14.74 | 145.20 | 0.4 | 0.4 | 0.5 | 0.3 | 0.6 | Medial |
| Great Barrier Reef | -14.73 | 145.19 | 0.4 | 0.4 | 0.5 | 0.3 | 0.6 | Medial |
| Great Barrier Reef | -14.73 | 145.10 | 0.7 | 0.4 | 0.6 | 0.3 | 0.7 | High |
| Great Barrier Reef | -14.73 | 145.19 | 0.4 | 0.4 | 0.5 | 0.3 | 0.6 | Medial |
| Great Barrier Reef | -14.73 | 145.18 | 0.4 | 0.4 | 0.5 | 0.3 | 0.6 | Medial |
| Great Barrier Reef | -14.72 | 145.16 | 0.4 | 0.4 | 0.5 | 0.3 | 0.6 | Medial |
| Great Barrier Reef | -14.72 | 145.19 | 0.4 | 0.4 | 0.5 | 0.3 | 0.6 | Medial |
| Great Barrier Reef | -14.71 | 145.38 | 0.5 | 0.4 | 0.5 | 0.2 | 0.6 | Medial |
| Great Barrier Reef | -14.71 | 144.99 | 0.5 | 0.3 | 0.5 | 0.3 | 0.7 | Medial |
| Great Barrier Reef | -14.71 | 145.38 | 0.5 | 0.4 | 0.5 | 0.2 | 0.6 | Medial |
| Great Barrier Reef | -14.70 | 145.20 | 0.4 | 0.4 | 0.4 | 0.3 | 0.6 | Medial |
| Great Barrier Reef | -14.69 | 145.47 | 0.5 | 0.4 | 0.5 | 0.2 | 0.6 | Medial |
| Great Barrier Reef | -14.69 | 145.45 | 0.5 | 0.4 | 0.5 | 0.2 | 0.6 | Medial |
| Great Barrier Reef | -14.68 | 145.70 | 0.5 | 0.4 | 0.5 | 0.2 | 0.6 | Medial |
| Great Barrier Reef | -14.68 | 145.47 | 0.5 | 0.4 | 0.5 | 0.2 | 0.6 | Medial |
| Great Barrier Reef | -14.66 | 145.65 | 0.4 | 0.4 | 0.5 | 0.2 | 0.6 | Medial |
| Great Barrier Reef | -14.66 | 145.45 | 0.6 | 0.3 | 0.6 | 0.2 | 0.6 | Medial |
| Polynesia | -14.66 | -145.18 | 0.5 | 0.9 | 0.7 | 0.1 | 0.7 | High |
| Great Barrier Reef | -14.66 | 145.47 | 0.5 | 0.3 | 0.5 | 0.2 | 0.6 | Medial |
| Great Barrier Reef | -14.66 | 145.25 | 0.4 | 0.4 | 0.5 | 0.2 | 0.6 | Medial |
| Great Barrier Reef | -14.66 | 145.25 | 0.4 | 0.4 | 0.5 | 0.2 | 0.6 | Medial |
| Great Barrier Reef | -14.65 | 145.49 | 0.5 | 0.3 | 0.5 | 0.2 | 0.6 | Medial |
| Great Barrier Reef | -14.65 | 145.45 | 0.6 | 0.3 | 0.6 | 0.2 | 0.6 | Medial |
| Great Barrier Reef | -14.65 | 145.49 | 0.5 | 0.3 | 0.5 | 0.2 | 0.6 | Medial |
| Great Barrier Reef | -14.65 | 145.65 | 0.4 | 0.4 | 0.5 | 0.2 | 0.6 | Medial |
| Great Barrier Reef | -14.63 | 145.48 | 0.5 | 0.3 | 0.5 | 0.2 | 0.6 | Medial |
| Great Barrier Reef | -14.63 | 145.65 | 0.4 | 0.4 | 0.4 | 0.2 | 0.5 | Medial |
| Great Barrier Reef | -14.61 | 144.94 | 0.5 | 0.4 | 0.5 | 0.3 | 0.6 | Medial |
| Great Barrier Reef | -14.60 | 145.62 | 0.3 | 0.4 | 0.4 | 0.2 | 0.5 | Medial |
| Polynesia | -14.58 | -145.22 | 0.5 | 0.9 | 0.6 | 0.0 | 0.6 | Medial |
| Great Barrier Reef | -14.57 | 145.62 | 0.3 | 0.4 | 0.4 | 0.2 | 0.5 | Medial |
| Great Barrier Reef | -14.55 | 145.58 | 0.3 | 0.4 | 0.4 | 0.2 | 0.5 | Medial |
| Great Barrier Reef | -14.55 | 145.59 | 0.3 | 0.4 | 0.4 | 0.2 | 0.5 | Medial |
| Great Barrier Reef | -14.55 | 145.59 | 0.3 | 0.4 | 0.4 | 0.2 | 0.5 | Medial |
| Great Barrier Reef | -14.54 | 144.99 | 0.6 | 0.4 | 0.6 | 0.3 | 0.7 | High |
| Great Barrier Reef | -14.53 | 145.59 | 0.4 | 0.4 | 0.4 | 0.2 | 0.6 | Medial |
| Great Barrier Reef | -14.52 | 144.97 | 0.6 | 0.4 | 0.6 | 0.3 | 0.7 | High |
| Great Barrier Reef | -14.51 | 145.16 | 0.4 | 0.4 | 0.5 | 0.3 | 0.6 | Medial |
| Western Indian Ocean | -14.50 | 41.00 | 0.5 | 0.5 | 0.5 | 0.2 | 0.6 | Medial |
| Great Barrier Reef | -14.50 | 145.54 | 0.5 | 0.4 | 0.5 | 0.2 | 0.6 | Medial |
| Great Barrier Reef | -14.50 | 145.54 | 0.5 | 0.4 | 0.5 | 0.2 | 0.6 | Medial |
| Great Barrier Reef | -14.50 | 144.97 | 0.5 | 0.4 | 0.5 | 0.3 | 0.7 | Medial |
| Great Barrier Reef | -14.48 | 144.96 | 0.5 | 0.4 | 0.5 | 0.3 | 0.7 | Medial |
| Great Barrier Reef | -14.47 | 145.03 | 0.5 | 0.4 | 0.5 | 0.3 | 0.7 | Medial |
| Great Barrier Reef | -14.45 | 145.48 | 0.4 | 0.4 | 0.5 | 0.2 | 0.6 | Medial |
| Great Barrier Reef | -14.45 | 145.22 | 0.4 | 0.4 | 0.5 | 0.2 | 0.6 | Medial |
| Great Barrier Reef | -14.45 | 145.48 | 0.4 | 0.4 | 0.5 | 0.2 | 0.6 | Medial |
| Great Barrier Reef | -14.45 | 145.41 | 0.5 | 0.4 | 0.5 | 0.2 | 0.6 | Medial |
| Great Barrier Reef | -14.44 | 145.41 | 0.5 | 0.4 | 0.5 | 0.2 | 0.6 | Medial |
| Great Barrier Reef | -14.43 | 144.89 | 0.5 | 0.4 | 0.5 | 0.3 | 0.7 | High |
| Great Barrier Reef | -14.43 | 144.81 | 0.4 | 0.4 | 0.5 | 0.3 | 0.6 | Medial |
| Great Barrier Reef | -14.42 | 145.10 | 0.5 | 0.4 | 0.5 | 0.3 | 0.6 | Medial |
| Great Barrier Reef | -14.42 | 145.32 | 0.4 | 0.4 | 0.4 | 0.2 | 0.5 | Medial |
| Great Barrier Reef | -14.42 | 145.32 | 0.4 | 0.4 | 0.4 | 0.2 | 0.5 | Medial |
| Great Barrier Reef | -14.42 | 144.95 | 0.6 | 0.4 | 0.6 | 0.3 | 0.7 | High |
| Great Barrier Reef | -14.41 | 144.95 | 0.6 | 0.4 | 0.6 | 0.3 | 0.7 | High |
| Polynesia | -14.40 | -145.86 | 0.5 | 0.9 | 0.7 | 0.7 | 0.9 | Severe |
| Great Barrier Reef | -14.36 | 144.76 | 0.5 | 0.4 | 0.5 | 0.3 | 0.6 | Medial |
| Polynesia | -14.34 | -170.79 | 0.6 | 0.9 | 0.7 | 0.0 | 0.7 | High |
| Polynesia | -14.33 | -170.83 | 0.6 | 0.9 | 0.7 | 0.0 | 0.7 | High |
| Polynesia | -14.33 | -170.71 | 0.6 | 0.9 | 0.7 | 0.0 | 0.7 | High |
| Polynesia | -14.33 | -170.70 | 0.6 | 0.9 | 0.7 | 0.0 | 0.7 | High |
| Great Barrier Reef | -14.32 | 144.86 | 0.4 | 0.4 | 0.4 | 0.3 | 0.6 | Medial |
| Polynesia | -14.32 | -170.68 | 0.6 | 0.9 | 0.7 | 0.0 | 0.7 | High |
| Great Barrier Reef | -14.32 | 144.74 | 0.6 | 0.4 | 0.6 | 0.3 | 0.7 | High |
| Great Barrier Reef | -14.31 | 144.79 | 0.6 | 0.4 | 0.6 | 0.3 | 0.7 | High |
| Polynesia | -14.30 | -178.81 | 0.7 | 0.9 | 0.8 | 0.0 | 0.8 | High |
| Great Barrier Reef | -14.29 | 144.69 | 0.6 | 0.4 | 0.6 | 0.3 | 0.7 | High |
| Polynesia | -14.29 | -170.68 | 0.6 | 0.9 | 0.7 | 0.0 | 0.7 | High |
| Polynesia | -14.28 | -170.67 | 0.6 | 0.9 | 0.7 | 0.0 | 0.7 | High |
| Polynesia | -14.28 | -170.67 | 0.6 | 0.9 | 0.7 | 0.0 | 0.7 | High |
| Polynesia | -14.28 | -170.68 | 0.6 | 0.9 | 0.7 | 0.0 | 0.7 | High |
| Polynesia | -14.28 | -170.58 | 0.6 | 0.9 | 0.7 | 0.0 | 0.7 | High |
| Polynesia | -14.26 | -170.59 | 0.6 | 0.9 | 0.7 | 0.0 | 0.7 | High |
| Great Barrier Reef | -14.22 | 144.67 | 0.6 | 0.4 | 0.5 | 0.3 | 0.7 | Medial |
| Great Barrier Reef | -14.19 | 144.26 | 0.6 | 0.3 | 0.6 | 0.8 | 0.9 | Severe |
| Great Barrier Reef | -14.18 | 144.65 | 0.5 | 0.4 | 0.5 | 0.3 | 0.6 | Medial |
| Great Barrier Reef | -14.18 | 144.22 | 0.6 | 0.3 | 0.6 | 1.0 | 1.0 | Severe |
| Great Barrier Reef | -14.17 | 144.28 | 0.4 | 0.3 | 0.4 | 0.8 | 0.9 | Severe |
| Great Barrier Reef | -14.17 | 144.24 | 0.6 | 0.3 | 0.5 | 0.8 | 0.9 | Severe |
| Great Barrier Reef | -14.15 | 144.49 | 0.5 | 0.3 | 0.5 | 0.5 | 0.8 | High |
| Polynesia | -14.13 | -169.63 | 0.6 | 0.9 | 0.7 | 0.0 | 0.7 | High |
| Great Barrier Reef | -14.13 | 144.00 | 0.6 | 0.3 | 0.5 | 0.9 | 1.0 | Severe |
| Great Barrier Reef | -14.12 | 144.52 | 0.5 | 0.4 | 0.5 | 0.4 | 0.7 | High |
| Polynesia | -14.11 | -169.65 | 0.6 | 0.9 | 0.7 | 0.0 | 0.7 | High |
| Great Barrier Reef | -14.10 | 144.32 | 0.5 | 0.4 | 0.5 | 0.9 | 1.0 | Severe |
| Western Australia | -14.08 | 121.85 | 0.9 | 0.6 | 0.9 | 0.5 | 0.9 | Severe |
| Great Barrier Reef | -14.08 | 143.91 | 0.6 | 0.3 | 0.5 | 0.9 | 0.9 | Severe |
| Great Barrier Reef | -14.07 | 144.24 | 0.5 | 0.3 | 0.5 | 1.0 | 1.0 | Severe |
| Great Barrier Reef | -14.06 | 143.86 | 0.6 | 0.3 | 0.6 | 0.8 | 0.9 | Severe |
| Western Australia | -14.06 | 121.96 | 0.9 | 0.6 | 0.8 | 0.3 | 0.9 | Severe |
| Great Barrier Reef | -14.01 | 143.98 | 0.6 | 0.3 | 0.6 | 1.0 | 1.0 | Severe |
| Polynesia | -14.00 | -171.00 | 0.6 | 0.9 | 0.7 | 0.0 | 0.7 | High |
| Great Barrier Reef | -13.98 | 144.11 | 0.5 | 0.4 | 0.5 | 1.0 | 1.0 | Severe |
| Great Barrier Reef | -13.96 | 143.84 | 0.5 | 0.3 | 0.5 | 0.9 | 1.0 | Severe |
| Great Barrier Reef | -13.94 | 143.75 | 0.5 | 0.3 | 0.5 | 0.8 | 0.9 | Severe |
| Polynesia | -13.93 | -171.55 | 0.7 | 0.9 | 0.7 | 0.0 | 0.7 | High |
| Western Indian Ocean | -13.92 | 47.67 | 0.5 | 0.5 | 0.6 | 0.3 | 0.7 | High |
| Great Barrier Reef | -13.92 | 143.84 | 0.6 | 0.3 | 0.5 | 0.9 | 1.0 | Severe |
| Great Barrier Reef | -13.91 | 143.95 | 0.5 | 0.4 | 0.5 | 1.0 | 1.0 | Severe |
| Great Barrier Reef | -13.87 | 143.71 | 0.6 | 0.3 | 0.6 | 0.9 | 0.9 | Severe |
| Polynesia | -13.83 | -171.79 | 0.7 | 0.9 | 0.8 | 0.1 | 0.8 | High |
| Polynesia | -13.82 | -171.73 | 0.7 | 0.9 | 0.7 | 0.0 | 0.8 | High |
| Great Barrier Reef | -13.77 | 143.64 | 0.6 | 0.3 | 0.6 | 0.9 | 0.9 | Severe |
| Great Barrier Reef | -13.74 | 143.91 | 0.5 | 0.3 | 0.5 | 1.0 | 1.0 | Severe |
| Western Australia | -13.69 | 122.08 | 0.9 | 0.6 | 0.9 | 0.3 | 0.9 | Severe |
| Western Australia | -13.67 | 122.00 | 0.9 | 0.6 | 0.9 | 0.9 | 1.0 | Severe |
| Great Barrier Reef | -13.67 | 143.69 | 0.7 | 0.3 | 0.6 | 0.9 | 1.0 | Severe |
| Great Barrier Reef | -13.65 | 143.73 | 0.6 | 0.3 | 0.6 | 1.0 | 1.0 | Severe |
| Western Australia | -13.65 | 122.03 | 0.9 | 0.6 | 0.9 | 0.9 | 1.0 | Severe |
| Great Barrier Reef | -13.64 | 143.92 | 0.5 | 0.4 | 0.5 | 1.0 | 1.0 | Severe |
| Great Barrier Reef | -13.56 | 143.84 | 0.6 | 0.4 | 0.6 | 1.0 | 1.0 | Severe |
| Great Barrier Reef | -13.55 | 143.84 | 0.6 | 0.4 | 0.6 | 1.0 | 1.0 | Severe |
| Great Barrier Reef | -13.53 | 143.80 | 0.6 | 0.3 | 0.6 | 1.0 | 1.0 | Severe |
| Western Indian Ocean | -13.50 | 47.50 | 0.4 | 0.5 | 0.5 | 0.1 | 0.6 | Medial |
| Western Indian Ocean | -13.49 | 48.24 | 0.5 | 0.5 | 0.6 | 0.7 | 0.9 | Severe |
| Western Indian Ocean | -13.49 | 48.24 | 0.5 | 0.5 | 0.6 | 0.7 | 0.9 | Severe |
| Great Barrier Reef | -13.44 | 143.98 | 0.5 | 0.4 | 0.5 | 1.0 | 1.0 | Severe |
| Great Barrier Reef | -13.40 | 143.97 | 0.5 | 0.4 | 0.5 | 0.9 | 1.0 | Severe |
| Great Barrier Reef | -13.37 | 143.96 | 0.5 | 0.4 | 0.5 | 1.0 | 1.0 | Severe |
| Great Barrier Reef | -13.33 | 143.78 | 0.6 | 0.4 | 0.6 | 1.0 | 1.0 | Severe |
| Western Indian Ocean | -13.33 | 45.83 | 0.6 | 0.5 | 0.6 | 0.1 | 0.7 | Medial |
| Great Barrier Reef | -13.32 | 143.73 | 0.6 | 0.4 | 0.6 | 1.0 | 1.0 | Severe |
| Western Indian Ocean | -13.32 | 45.70 | 0.6 | 0.5 | 0.6 | 0.1 | 0.7 | High |
| Western Indian Ocean | -13.32 | 45.82 | 0.6 | 0.5 | 0.6 | 0.1 | 0.7 | Medial |
| Great Barrier Reef | -13.30 | 143.68 | 0.6 | 0.4 | 0.6 | 1.0 | 1.0 | Severe |
| Great Barrier Reef | -13.28 | 143.60 | 0.8 | 0.4 | 0.7 | 1.0 | 1.0 | Severe |
| Great Barrier Reef | -13.27 | 143.94 | 0.5 | 0.4 | 0.5 | 1.0 | 1.0 | Severe |
| Western Indian Ocean | -13.19 | 48.28 | 0.6 | 0.5 | 0.6 | 0.6 | 0.9 | Severe |
| Great Barrier Reef | -13.18 | 143.57 | 0.7 | 0.4 | 0.6 | 1.0 | 1.0 | Severe |
| Western Indian Ocean | -13.18 | 48.23 | 0.5 | 0.5 | 0.6 | 0.7 | 0.9 | Severe |
| Great Barrier Reef | -13.17 | 143.76 | 0.5 | 0.4 | 0.5 | 1.0 | 1.0 | Severe |
| Great Barrier Reef | -13.17 | 143.66 | 0.6 | 0.4 | 0.6 | 1.0 | 1.0 | Severe |
| Great Barrier Reef | -13.14 | 143.95 | 0.4 | 0.4 | 0.5 | 0.9 | 1.0 | Severe |
| Western Indian Ocean | -13.09 | 45.34 | 0.6 | 0.5 | 0.6 | 0.2 | 0.7 | High |
| Great Barrier Reef | -13.08 | 143.91 | 0.5 | 0.4 | 0.5 | 1.0 | 1.0 | Severe |
| Western Indian Ocean | -13.07 | 45.11 | 0.4 | 0.5 | 0.5 | 0.3 | 0.7 | High |
| Great Barrier Reef | -13.06 | 143.88 | 0.5 | 0.4 | 0.5 | 1.0 | 1.0 | Severe |
| Great Barrier Reef | -13.05 | 143.87 | 0.4 | 0.4 | 0.5 | 1.0 | 1.0 | Severe |
| Great Barrier Reef | -13.04 | 143.87 | 0.5 | 0.4 | 0.5 | 1.0 | 1.0 | Severe |
| Great Barrier Reef | -13.04 | 143.87 | 0.5 | 0.4 | 0.5 | 1.0 | 1.0 | Severe |
| Western Indian Ocean | -13.04 | 48.55 | 0.5 | 0.5 | 0.5 | 0.6 | 0.8 | High |
| Western Indian Ocean | -13.03 | 48.53 | 0.5 | 0.5 | 0.5 | 0.6 | 0.8 | High |
| Great Barrier Reef | -13.02 | 143.85 | 0.5 | 0.4 | 0.5 | 1.0 | 1.0 | Severe |
| Western Indian Ocean | -13.00 | 40.50 | 0.9 | 0.5 | 0.8 | 0.5 | 0.9 | Severe |
| Western Indian Ocean | -13.00 | 45.50 | 0.5 | 0.5 | 0.6 | 0.1 | 0.6 | Medial |
| Great Barrier Reef | -13.00 | 143.85 | 0.5 | 0.4 | 0.6 | 1.0 | 1.0 | Severe |
| Great Barrier Reef | -12.99 | 143.61 | 0.5 | 0.4 | 0.5 | 1.0 | 1.0 | Severe |
| Western Indian Ocean | -12.98 | 44.98 | 0.5 | 0.5 | 0.6 | 0.8 | 0.9 | Severe |
| Western Indian Ocean | -12.97 | 45.08 | 0.5 | 0.5 | 0.6 | 0.4 | 0.8 | High |
| Western Indian Ocean | -12.96 | 40.56 | 0.8 | 0.5 | 0.8 | 0.5 | 0.9 | Severe |
| Western Indian Ocean | -12.96 | 40.58 | 0.8 | 0.5 | 0.7 | 0.5 | 0.9 | Severe |
| Great Barrier Reef | -12.95 | 143.69 | 0.6 | 0.4 | 0.5 | 1.0 | 1.0 | Severe |
| Great Barrier Reef | -12.95 | 143.82 | 0.5 | 0.4 | 0.5 | 1.0 | 1.0 | Severe |
| Great Barrier Reef | -12.94 | 143.82 | 0.5 | 0.4 | 0.5 | 1.0 | 1.0 | Severe |
| Western Indian Ocean | -12.94 | 48.54 | 0.5 | 0.5 | 0.5 | 0.5 | 0.8 | High |
| Great Barrier Reef | -12.93 | 143.66 | 0.6 | 0.4 | 0.6 | 1.0 | 1.0 | Severe |
| Great Barrier Reef | -12.89 | 143.60 | 0.6 | 0.4 | 0.6 | 1.0 | 1.0 | Severe |
| Western Indian Ocean | -12.88 | 44.95 | 0.4 | 0.5 | 0.5 | 0.2 | 0.6 | Medial |
| Great Barrier Reef | -12.88 | 143.60 | 0.6 | 0.4 | 0.6 | 1.0 | 1.0 | Severe |
| Western Indian Ocean | -12.87 | 45.28 | 0.4 | 0.5 | 0.5 | 0.2 | 0.6 | Medial |
| Western Indian Ocean | -12.87 | 45.27 | 0.4 | 0.5 | 0.5 | 0.2 | 0.6 | Medial |
| Western Indian Ocean | -12.87 | 45.27 | 0.4 | 0.5 | 0.5 | 0.2 | 0.6 | Medial |
| Western Indian Ocean | -12.85 | 48.57 | 0.5 | 0.5 | 0.5 | 0.6 | 0.8 | High |
| Great Barrier Reef | -12.83 | 143.84 | 0.4 | 0.4 | 0.5 | 1.0 | 1.0 | Severe |
| Western Indian Ocean | -12.82 | 48.59 | 0.4 | 0.5 | 0.5 | 0.6 | 0.8 | High |
| Western Indian Ocean | -12.82 | 48.64 | 0.5 | 0.5 | 0.6 | 0.6 | 0.8 | Severe |
| Great Barrier Reef | -12.81 | 143.61 | 0.6 | 0.4 | 0.6 | 1.0 | 1.0 | Severe |
| Great Barrier Reef | -12.78 | 143.49 | 0.5 | 0.4 | 0.5 | 1.0 | 1.0 | Severe |
| Great Barrier Reef | -12.78 | 143.41 | 0.8 | 0.4 | 0.7 | 1.0 | 1.0 | Severe |
| Great Barrier Reef | -12.78 | 143.47 | 0.5 | 0.4 | 0.5 | 1.0 | 1.0 | Severe |
| Great Barrier Reef | -12.77 | 143.61 | 0.6 | 0.4 | 0.5 | 1.0 | 1.0 | Severe |
| Great Barrier Reef | -12.77 | 143.61 | 0.6 | 0.4 | 0.5 | 1.0 | 1.0 | Severe |
| Great Barrier Reef | -12.75 | 143.40 | 0.8 | 0.4 | 0.7 | 1.0 | 1.0 | Severe |
| Great Barrier Reef | -12.75 | 143.40 | 0.8 | 0.4 | 0.7 | 1.0 | 1.0 | Severe |
| Great Barrier Reef | -12.71 | 143.61 | 0.5 | 0.4 | 0.5 | 1.0 | 1.0 | Severe |
| Great Barrier Reef | -12.71 | 143.61 | 0.5 | 0.4 | 0.5 | 1.0 | 1.0 | Severe |
| Western Indian Ocean | -12.70 | 45.26 | 0.6 | 0.5 | 0.6 | 0.2 | 0.7 | High |
| Western Indian Ocean | -12.70 | 45.20 | 0.6 | 0.5 | 0.6 | 0.3 | 0.7 | High |
| Western Indian Ocean | -12.69 | 45.17 | 0.5 | 0.5 | 0.6 | 0.3 | 0.7 | High |
| Western Indian Ocean | -12.69 | 45.04 | 0.4 | 0.5 | 0.5 | 0.5 | 0.8 | High |
| Western Indian Ocean | -12.68 | 44.96 | 0.4 | 0.5 | 0.5 | 0.8 | 0.9 | Severe |
| South East Asia | -12.66 | 98.29 | 0.6 | 0.9 | 0.7 | 0.1 | 0.7 | High |
| Western Indian Ocean | -12.65 | 45.13 | 0.4 | 0.5 | 0.5 | 0.3 | 0.7 | High |
| Great Barrier Reef | -12.64 | 143.56 | 0.4 | 0.4 | 0.5 | 1.0 | 1.0 | Severe |
| Great Barrier Reef | -12.58 | 143.55 | 0.4 | 0.4 | 0.5 | 1.0 | 1.0 | Severe |
| Western Indian Ocean | -12.58 | 44.93 | 0.5 | 0.5 | 0.6 | 0.2 | 0.6 | Medial |
| Western Indian Ocean | -12.57 | 45.17 | 0.5 | 0.5 | 0.6 | 0.2 | 0.7 | Medial |
| Great Barrier Reef | -12.56 | 143.53 | 0.5 | 0.4 | 0.5 | 1.0 | 1.0 | Severe |
| Great Barrier Reef | -12.53 | 143.53 | 0.5 | 0.4 | 0.5 | 1.0 | 1.0 | Severe |
| Western Australia | -12.53 | 123.54 | 0.7 | 0.6 | 0.8 | 0.4 | 0.9 | Severe |
| Great Barrier Reef | -12.52 | 143.39 | 0.6 | 0.4 | 0.6 | 0.9 | 1.0 | Severe |
| Western Indian Ocean | -12.50 | 40.64 | 0.6 | 0.5 | 0.7 | 0.7 | 0.9 | Severe |
| Great Barrier Reef | -12.48 | 143.43 | 0.6 | 0.4 | 0.6 | 1.0 | 1.0 | Severe |
| Great Barrier Reef | -12.45 | 143.50 | 0.5 | 0.4 | 0.5 | 1.0 | 1.0 | Severe |
| Great Barrier Reef | -12.42 | 143.50 | 0.5 | 0.4 | 0.5 | 1.0 | 1.0 | Severe |
| Great Barrier Reef | -12.38 | 143.49 | 0.6 | 0.4 | 0.6 | 1.0 | 1.0 | Severe |
| Great Barrier Reef | -12.38 | 143.54 | 0.6 | 0.4 | 0.6 | 1.0 | 1.0 | Severe |
| Great Barrier Reef | -12.32 | 143.33 | 0.7 | 0.4 | 0.6 | 1.0 | 1.0 | Severe |
| Great Barrier Reef | -12.31 | 143.43 | 0.7 | 0.4 | 0.6 | 1.0 | 1.0 | Severe |
| Western Indian Ocean | -12.30 | 43.80 | 0.6 | 0.5 | 0.7 | 0.1 | 0.7 | High |
| Great Barrier Reef | -12.30 | 143.42 | 0.7 | 0.4 | 0.7 | 1.0 | 1.0 | Severe |
| Western Indian Ocean | -12.28 | 40.62 | 0.7 | 0.5 | 0.6 | 0.4 | 0.8 | High |
| Western Indian Ocean | -12.28 | 40.60 | 0.7 | 0.5 | 0.7 | 0.4 | 0.8 | Severe |
| Western Indian Ocean | -12.28 | 43.75 | 0.6 | 0.5 | 0.7 | 0.1 | 0.7 | High |
| Great Barrier Reef | -12.27 | 143.92 | 0.6 | 0.4 | 0.6 | 0.9 | 1.0 | Severe |
| Great Barrier Reef | -12.27 | 143.30 | 0.7 | 0.4 | 0.6 | 1.0 | 1.0 | Severe |
| Western Indian Ocean | -12.27 | 40.62 | 0.7 | 0.5 | 0.6 | 0.4 | 0.8 | High |
| Western Australia | -12.27 | 123.00 | 0.7 | 0.6 | 0.8 | 0.5 | 0.9 | Severe |
| Great Barrier Reef | -12.25 | 143.24 | 0.5 | 0.4 | 0.5 | 1.0 | 1.0 | Severe |
| Great Barrier Reef | -12.25 | 143.28 | 0.5 | 0.4 | 0.5 | 1.0 | 1.0 | Severe |
| Great Barrier Reef | -12.24 | 143.21 | 0.4 | 0.4 | 0.5 | 1.0 | 1.0 | Severe |
| Great Barrier Reef | -12.24 | 143.51 | 0.5 | 0.4 | 0.5 | 1.0 | 1.0 | Severe |
| Great Barrier Reef | -12.23 | 143.28 | 0.5 | 0.4 | 0.5 | 1.0 | 1.0 | Severe |
| Great Barrier Reef | -12.23 | 143.29 | 0.5 | 0.4 | 0.5 | 1.0 | 1.0 | Severe |
| Great Barrier Reef | -12.22 | 143.28 | 0.5 | 0.4 | 0.5 | 1.0 | 1.0 | Severe |
| Western Australia | -12.17 | 122.83 | 0.7 | 0.6 | 0.8 | 0.2 | 0.8 | Severe |
| South East Asia | -12.16 | 97.02 | 0.7 | 0.9 | 0.7 | 0.1 | 0.8 | High |
| South East Asia | -12.16 | 96.82 | 0.6 | 0.9 | 0.7 | 0.1 | 0.7 | High |
| South East Asia | -12.15 | 96.83 | 0.6 | 0.9 | 0.7 | 0.1 | 0.7 | High |
| Great Barrier Reef | -12.14 | 143.37 | 0.6 | 0.4 | 0.6 | 1.0 | 1.0 | Severe |
| Great Barrier Reef | -12.14 | 143.37 | 0.6 | 0.4 | 0.6 | 1.0 | 1.0 | Severe |
| Great Barrier Reef | -12.14 | 143.27 | 0.5 | 0.4 | 0.5 | 1.0 | 1.0 | Severe |
| Great Barrier Reef | -12.14 | 143.28 | 0.5 | 0.4 | 0.5 | 1.0 | 1.0 | Severe |
| South East Asia | -12.14 | 96.92 | 0.7 | 0.9 | 0.7 | 0.1 | 0.8 | High |
| South East Asia | -12.14 | 96.92 | 0.7 | 0.9 | 0.7 | 0.1 | 0.8 | High |
| South East Asia | -12.13 | 96.90 | 0.7 | 0.9 | 0.7 | 0.1 | 0.8 | High |
| South East Asia | -12.13 | 96.83 | 0.6 | 0.9 | 0.7 | 0.1 | 0.7 | High |
| Great Barrier Reef | -12.13 | 143.66 | 0.5 | 0.4 | 0.5 | 1.0 | 1.0 | Severe |
| Great Barrier Reef | -12.13 | 143.22 | 0.7 | 0.4 | 0.6 | 1.0 | 1.0 | Severe |
| South East Asia | -12.11 | 96.87 | 0.6 | 0.9 | 0.7 | 0.1 | 0.7 | High |
| South East Asia | -12.10 | 96.90 | 0.6 | 0.9 | 0.7 | 0.1 | 0.7 | High |
| Great Barrier Reef | -12.10 | 143.48 | 0.5 | 0.4 | 0.6 | 1.0 | 1.0 | Severe |
| Western Indian Ocean | -12.10 | 43.80 | 0.6 | 0.5 | 0.6 | 0.1 | 0.7 | High |
| Great Barrier Reef | -12.10 | 143.26 | 0.6 | 0.4 | 0.6 | 1.0 | 1.0 | Severe |
| South East Asia | -12.10 | 96.85 | 0.6 | 0.9 | 0.7 | 0.1 | 0.7 | High |
| Great Barrier Reef | -12.10 | 143.27 | 0.6 | 0.4 | 0.6 | 1.0 | 1.0 | Severe |
| South East Asia | -12.09 | 96.84 | 0.6 | 0.9 | 0.7 | 0.1 | 0.7 | High |
| South East Asia | -12.09 | 96.84 | 0.6 | 0.9 | 0.7 | 0.1 | 0.7 | High |
| South East Asia | -12.09 | 96.85 | 0.6 | 0.9 | 0.7 | 0.1 | 0.7 | High |
| South East Asia | -12.08 | 96.75 | 0.6 | 0.9 | 0.7 | 0.1 | 0.7 | High |
| Great Barrier Reef | -12.08 | 143.97 | 0.6 | 0.4 | 0.6 | 0.8 | 0.9 | Severe |
| Great Barrier Reef | -12.03 | 143.30 | 0.6 | 0.4 | 0.6 | 1.0 | 1.0 | Severe |
| Great Barrier Reef | -12.03 | 143.30 | 0.6 | 0.4 | 0.6 | 1.0 | 1.0 | Severe |
| Western Indian Ocean | -12.00 | 40.00 | 0.7 | 0.5 | 0.7 | 0.4 | 0.8 | High |
| Western Indian Ocean | -12.00 | 40.02 | 0.7 | 0.5 | 0.7 | 0.4 | 0.8 | High |
| Western Indian Ocean | -12.00 | 43.50 | 0.5 | 0.5 | 0.6 | 0.1 | 0.6 | Medial |
| Western Australia | -11.99 | 123.41 | 0.6 | 0.6 | 0.7 | 0.3 | 0.8 | High |
| Great Barrier Reef | -11.99 | 143.26 | 0.6 | 0.4 | 0.6 | 1.0 | 1.0 | Severe |
| Great Barrier Reef | -11.99 | 143.26 | 0.6 | 0.4 | 0.6 | 1.0 | 1.0 | Severe |
| Great Barrier Reef | -11.97 | 143.28 | 0.6 | 0.4 | 0.6 | 1.0 | 1.0 | Severe |
| Great Barrier Reef | -11.96 | 143.27 | 0.6 | 0.4 | 0.6 | 1.0 | 1.0 | Severe |
| Great Barrier Reef | -11.95 | 143.27 | 0.6 | 0.4 | 0.6 | 1.0 | 1.0 | Severe |
| Great Barrier Reef | -11.95 | 143.27 | 0.7 | 0.4 | 0.7 | 1.0 | 1.0 | Severe |
| Great Barrier Reef | -11.95 | 143.99 | 0.6 | 0.4 | 0.6 | 0.9 | 0.9 | Severe |
| Great Barrier Reef | -11.93 | 143.21 | 0.7 | 0.4 | 0.6 | 1.0 | 1.0 | Severe |
| Great Barrier Reef | -11.91 | 143.49 | 0.4 | 0.4 | 0.5 | 1.0 | 1.0 | Severe |
| Great Barrier Reef | -11.90 | 143.11 | 0.7 | 0.4 | 0.7 | 1.0 | 1.0 | Severe |
| Great Barrier Reef | -11.89 | 143.11 | 0.7 | 0.4 | 0.7 | 1.0 | 1.0 | Severe |
| Great Barrier Reef | -11.87 | 143.30 | 0.7 | 0.4 | 0.7 | 1.0 | 1.0 | Severe |
| Great Barrier Reef | -11.87 | 143.30 | 0.7 | 0.4 | 0.7 | 1.0 | 1.0 | Severe |
| Great Barrier Reef | -11.87 | 143.29 | 0.7 | 0.4 | 0.7 | 1.0 | 1.0 | Severe |
| Western Indian Ocean | -11.86 | 40.55 | 0.7 | 0.5 | 0.7 | 0.4 | 0.8 | High |
| Great Barrier Reef | -11.84 | 142.94 | 0.8 | 0.4 | 0.7 | 1.0 | 1.0 | Severe |
| Western Indian Ocean | -11.84 | 40.57 | 0.5 | 0.5 | 0.6 | 0.4 | 0.7 | High |
| Great Barrier Reef | -11.82 | 143.36 | 0.7 | 0.4 | 0.6 | 1.0 | 1.0 | Severe |
| South East Asia | -11.82 | 96.82 | 0.7 | 0.9 | 0.7 | 0.1 | 0.8 | High |
| South East Asia | -11.82 | 96.82 | 0.7 | 0.9 | 0.7 | 0.1 | 0.8 | High |
| Great Barrier Reef | -11.80 | 142.94 | 0.8 | 0.4 | 0.7 | 1.0 | 1.0 | Severe |
| Great Barrier Reef | -11.79 | 143.08 | 0.7 | 0.4 | 0.7 | 1.0 | 1.0 | Severe |
| Great Barrier Reef | -11.77 | 142.94 | 0.7 | 0.4 | 0.7 | 1.0 | 1.0 | Severe |
| Great Barrier Reef | -11.77 | 143.08 | 0.7 | 0.4 | 0.7 | 1.0 | 1.0 | Severe |
| Great Barrier Reef | -11.77 | 143.09 | 0.7 | 0.4 | 0.7 | 1.0 | 1.0 | Severe |
| Great Barrier Reef | -11.77 | 143.10 | 0.7 | 0.4 | 0.7 | 1.0 | 1.0 | Severe |
| Great Barrier Reef | -11.76 | 142.97 | 0.7 | 0.4 | 0.7 | 1.0 | 1.0 | Severe |
| Great Barrier Reef | -11.73 | 142.99 | 0.7 | 0.4 | 0.6 | 1.0 | 1.0 | Severe |
| Great Barrier Reef | -11.71 | 143.19 | 0.7 | 0.4 | 0.7 | 1.0 | 1.0 | Severe |
| Western Indian Ocean | -11.70 | 40.60 | 0.5 | 0.5 | 0.6 | 0.4 | 0.7 | High |
| Western Indian Ocean | -11.70 | 40.60 | 0.5 | 0.5 | 0.6 | 0.4 | 0.7 | High |
| Great Barrier Reef | -11.60 | 142.94 | 0.7 | 0.4 | 0.6 | 1.0 | 1.0 | Severe |
| Great Barrier Reef | -11.60 | 143.01 | 0.7 | 0.4 | 0.7 | 1.0 | 1.0 | Severe |
| Great Barrier Reef | -11.59 | 144.04 | 0.5 | 0.4 | 0.6 | 1.0 | 1.0 | Severe |
| Great Barrier Reef | -11.55 | 143.02 | 0.7 | 0.4 | 0.7 | 1.0 | 1.0 | Severe |
| Western Indian Ocean | -11.53 | 40.63 | 0.6 | 0.5 | 0.6 | 0.4 | 0.8 | High |
| Great Barrier Reef | -11.52 | 143.36 | 0.6 | 0.4 | 0.6 | 1.0 | 1.0 | Severe |
| Great Barrier Reef | -11.51 | 142.87 | 0.7 | 0.4 | 0.7 | 1.0 | 1.0 | Severe |
| Great Barrier Reef | -11.50 | 143.03 | 0.7 | 0.4 | 0.7 | 1.0 | 1.0 | Severe |
| Western Indian Ocean | -11.49 | 43.40 | 0.6 | 0.5 | 0.7 | 0.4 | 0.8 | High |
| Great Barrier Reef | -11.49 | 143.04 | 0.7 | 0.4 | 0.7 | 1.0 | 1.0 | Severe |
| Great Barrier Reef | -11.49 | 143.04 | 0.7 | 0.4 | 0.7 | 1.0 | 1.0 | Severe |
| Great Barrier Reef | -11.47 | 143.16 | 0.7 | 0.4 | 0.7 | 1.0 | 1.0 | Severe |
| Great Barrier Reef | -11.46 | 143.04 | 0.7 | 0.4 | 0.6 | 1.0 | 1.0 | Severe |
| Great Barrier Reef | -11.45 | 143.04 | 0.7 | 0.4 | 0.7 | 1.0 | 1.0 | Severe |
| Great Barrier Reef | -11.45 | 143.76 | 0.5 | 0.4 | 0.6 | 1.0 | 1.0 | Severe |
| Great Barrier Reef | -11.43 | 143.17 | 0.7 | 0.4 | 0.7 | 1.0 | 1.0 | Severe |
| Great Barrier Reef | -11.43 | 144.01 | 0.6 | 0.4 | 0.6 | 1.0 | 1.0 | Severe |
| Great Barrier Reef | -11.40 | 143.96 | 0.7 | 0.4 | 0.7 | 1.0 | 1.0 | Severe |
| Great Barrier Reef | -11.39 | 143.07 | 0.6 | 0.4 | 0.6 | 1.0 | 1.0 | Severe |
| Great Barrier Reef | -11.39 | 143.07 | 0.6 | 0.4 | 0.6 | 1.0 | 1.0 | Severe |
| Great Barrier Reef | -11.39 | 142.97 | 0.6 | 0.4 | 0.6 | 1.0 | 1.0 | Severe |
| Great Barrier Reef | -11.38 | 143.04 | 0.6 | 0.4 | 0.6 | 1.0 | 1.0 | Severe |
| Great Barrier Reef | -11.38 | 142.97 | 0.6 | 0.4 | 0.6 | 1.0 | 1.0 | Severe |
| Great Barrier Reef | -11.26 | 142.87 | 0.7 | 0.4 | 0.7 | 1.0 | 1.0 | Severe |
| Great Barrier Reef | -11.26 | 142.87 | 0.7 | 0.4 | 0.7 | 1.0 | 1.0 | Severe |
| Great Barrier Reef | -11.25 | 142.93 | 0.6 | 0.4 | 0.6 | 1.0 | 1.0 | Severe |
| Great Barrier Reef | -11.25 | 142.93 | 0.6 | 0.4 | 0.6 | 1.0 | 1.0 | Severe |
| Great Barrier Reef | -11.24 | 143.00 | 0.6 | 0.4 | 0.6 | 1.0 | 1.0 | Severe |
| Great Barrier Reef | -11.24 | 143.00 | 0.6 | 0.4 | 0.6 | 1.0 | 1.0 | Severe |
| Western Indian Ocean | -11.21 | 40.67 | 0.6 | 0.5 | 0.6 | 0.4 | 0.8 | High |
| Great Barrier Reef | -11.18 | 143.03 | 0.6 | 0.4 | 0.6 | 1.0 | 1.0 | Severe |
| Great Barrier Reef | -11.18 | 143.03 | 0.6 | 0.4 | 0.6 | 1.0 | 1.0 | Severe |
| Great Barrier Reef | -11.17 | 143.10 | 0.6 | 0.4 | 0.6 | 1.0 | 1.0 | Severe |
| Great Barrier Reef | -11.17 | 143.10 | 0.6 | 0.4 | 0.6 | 1.0 | 1.0 | Severe |
| Great Barrier Reef | -11.11 | 143.02 | 0.6 | 0.4 | 0.6 | 1.0 | 1.0 | Severe |
| Great Barrier Reef | -11.11 | 143.03 | 0.6 | 0.4 | 0.6 | 1.0 | 1.0 | Severe |
| Great Barrier Reef | -11.07 | 143.09 | 0.7 | 0.4 | 0.6 | 1.0 | 1.0 | Severe |
| Great Barrier Reef | -11.07 | 143.09 | 0.7 | 0.4 | 0.6 | 1.0 | 1.0 | Severe |
| Western Indian Ocean | -11.04 | 40.70 | 0.5 | 0.5 | 0.6 | 0.3 | 0.7 | High |
| Western Indian Ocean | -11.04 | 40.70 | 0.5 | 0.5 | 0.6 | 0.3 | 0.7 | High |
| Western Indian Ocean | -11.03 | 40.64 | 0.6 | 0.5 | 0.6 | 0.4 | 0.8 | High |
| Great Barrier Reef | -11.01 | 142.99 | 0.6 | 0.4 | 0.6 | 1.0 | 1.0 | Severe |
| Western Indian Ocean | -11.01 | 40.61 | 0.6 | 0.5 | 0.6 | 0.4 | 0.8 | High |
| Great Barrier Reef | -11.01 | 142.99 | 0.6 | 0.4 | 0.6 | 1.0 | 1.0 | Severe |
| Western Indian Ocean | -11.00 | 40.69 | 0.6 | 0.5 | 0.7 | 0.4 | 0.8 | High |
| Western Indian Ocean | -10.99 | 40.71 | 0.5 | 0.5 | 0.6 | 0.3 | 0.7 | High |
| Western Indian Ocean | -10.91 | 40.67 | 0.6 | 0.5 | 0.7 | 0.4 | 0.8 | High |
| Great Barrier Reef | -10.90 | 142.78 | 0.6 | 0.4 | 0.6 | 1.0 | 1.0 | Severe |
| Great Barrier Reef | -10.89 | 142.70 | 0.6 | 0.4 | 0.6 | 1.0 | 1.0 | Severe |
| Great Barrier Reef | -10.82 | 142.76 | 0.6 | 0.4 | 0.6 | 1.0 | 1.0 | Severe |
| Great Barrier Reef | -10.82 | 142.77 | 0.6 | 0.4 | 0.6 | 1.0 | 1.0 | Severe |
| Great Barrier Reef | -10.80 | 142.98 | 0.7 | 0.4 | 0.7 | 1.0 | 1.0 | Severe |
| Great Barrier Reef | -10.70 | 142.73 | 0.7 | 0.4 | 0.7 | 1.0 | 1.0 | Severe |
| Great Barrier Reef | -10.70 | 142.73 | 0.7 | 0.4 | 0.7 | 1.0 | 1.0 | Severe |
| Great Barrier Reef | -10.68 | 142.74 | 0.7 | 0.4 | 0.7 | 1.0 | 1.0 | Severe |
| South East Asia | -10.50 | 105.51 | 0.6 | 0.8 | 0.7 | 0.1 | 0.7 | High |
| South East Asia | -10.42 | 105.67 | 0.7 | 0.7 | 0.8 | 0.1 | 0.8 | High |
| South East Asia | -10.40 | 105.68 | 0.7 | 0.7 | 0.8 | 0.1 | 0.8 | High |
| Western Indian Ocean | -10.30 | 40.39 | 0.7 | 0.5 | 0.7 | 0.4 | 0.8 | High |
| Western Indian Ocean | -10.30 | 40.36 | 0.8 | 0.5 | 0.7 | 0.4 | 0.9 | Severe |
| Western Indian Ocean | -10.26 | 40.38 | 0.7 | 0.5 | 0.7 | 0.4 | 0.8 | Severe |
| Western Indian Ocean | -10.21 | 51.33 | 0.4 | 0.4 | 0.5 | 0.2 | 0.6 | Medial |
| Western Indian Ocean | -10.19 | 40.21 | 0.4 | 0.5 | 0.5 | 0.4 | 0.7 | High |
| Western Indian Ocean | -10.19 | 40.22 | 0.4 | 0.5 | 0.5 | 0.4 | 0.7 | High |
| Great Barrier Reef | -10.18 | 151.19 | 0.8 | 0.6 | 0.8 | 0.3 | 0.9 | Severe |
| Great Barrier Reef | -10.17 | 161.95 | 0.8 | 0.7 | 0.8 | 0.2 | 0.8 | Severe |
| Western Indian Ocean | -10.00 | 40.02 | 0.2 | 0.5 | 0.4 | 0.2 | 0.6 | Medial |
| Western Indian Ocean | -10.00 | 40.03 | 0.2 | 0.5 | 0.4 | 0.2 | 0.6 | Medial |
| Western Indian Ocean | -10.00 | 40.05 | 0.4 | 0.5 | 0.5 | 0.2 | 0.6 | Medial |
| Western Indian Ocean | -10.00 | 40.07 | 0.4 | 0.5 | 0.5 | 0.2 | 0.6 | Medial |
| Great Barrier Reef | -9.92 | 151.06 | 0.8 | 0.6 | 0.8 | 0.2 | 0.8 | Severe |
| Western Indian Ocean | -9.67 | 47.10 | 0.5 | 0.4 | 0.6 | 0.2 | 0.7 | Medial |
| Great Barrier Reef | -9.60 | 147.22 | 0.7 | 0.5 | 0.7 | 0.6 | 0.9 | Severe |
| Western Indian Ocean | -9.54 | 51.00 | 0.6 | 0.4 | 0.6 | 1.0 | 1.0 | Severe |
| Western Indian Ocean | -9.50 | 46.20 | 0.5 | 0.4 | 0.5 | 0.2 | 0.7 | Medial |
| Western Indian Ocean | -9.50 | 46.50 | 0.5 | 0.4 | 0.6 | 0.2 | 0.7 | Medial |
| Western Indian Ocean | -9.50 | 55.50 | 0.7 | 0.6 | 0.7 | 0.2 | 0.8 | High |
| Western Indian Ocean | -9.40 | 46.20 | 0.7 | 0.4 | 0.7 | 0.7 | 0.9 | Severe |
| Western Indian Ocean | -9.38 | 51.05 | 0.7 | 0.4 | 0.7 | 1.0 | 1.0 | Severe |
| Western Indian Ocean | -9.33 | 50.73 | 0.6 | 0.4 | 0.6 | 0.2 | 0.7 | High |
| Western Indian Ocean | -9.32 | 51.72 | 0.5 | 0.4 | 0.6 | 0.2 | 0.7 | High |
| Western Indian Ocean | -9.31 | 50.71 | 0.6 | 0.4 | 0.6 | 0.3 | 0.7 | High |
| Western Indian Ocean | -9.25 | 51.04 | 0.6 | 0.4 | 0.6 | 1.0 | 1.0 | Severe |
| Western Indian Ocean | -9.21 | 51.04 | 0.5 | 0.4 | 0.6 | 0.3 | 0.7 | High |
| Great Barrier Reef | -9.21 | 150.59 | 0.7 | 0.6 | 0.7 | 0.4 | 0.8 | Severe |
| Great Barrier Reef | -9.14 | 149.34 | 0.8 | 0.8 | 0.8 | 0.3 | 0.9 | Severe |
| Great Barrier Reef | -9.01 | 160.10 | 0.9 | 0.9 | 0.9 | 0.3 | 0.9 | Severe |
| Great Barrier Reef | -9.00 | 159.25 | 0.8 | 0.8 | 0.9 | 0.3 | 0.9 | Severe |
| Central Indian Ocean | -8.78 | 81.25 | 0.7 | 0.8 | 0.8 | 0.1 | 0.8 | High |
| Central Indian Ocean | -8.73 | 81.18 | 0.8 | 0.8 | 0.8 | 0.1 | 0.8 | Severe |
| South East Asia | -8.72 | 115.94 | 0.6 | 0.7 | 0.8 | 0.7 | 0.9 | Severe |
| Western Indian Ocean | -8.72 | 39.52 | 0.3 | 0.5 | 0.4 | 0.7 | 0.9 | Severe |
| Central Indian Ocean | -8.68 | 81.34 | 0.8 | 0.9 | 0.8 | 0.1 | 0.8 | Severe |
| South East Asia | -8.67 | 115.47 | 0.9 | 0.7 | 0.9 | 0.5 | 0.9 | Severe |
| South East Asia | -8.66 | 115.46 | 0.9 | 0.7 | 0.9 | 0.5 | 0.9 | Severe |
| Polynesia | -8.50 | 179.18 | 0.7 | 0.9 | 0.8 | 0.0 | 0.8 | Severe |
| Great Barrier Reef | -8.49 | 158.13 | 0.8 | 0.8 | 0.8 | 0.2 | 0.9 | Severe |
| Western Indian Ocean | -8.42 | 39.26 | 0.6 | 0.5 | 0.6 | 0.8 | 0.9 | Severe |
| South East Asia | -8.38 | 116.05 | 0.8 | 0.8 | 0.9 | 0.4 | 0.9 | Severe |
| South East Asia | -8.33 | 116.06 | 0.8 | 0.8 | 0.9 | 0.6 | 1.0 | Severe |
| South East Asia | -8.26 | 116.69 | 0.6 | 0.8 | 0.7 | 0.2 | 0.8 | High |
| Western Indian Ocean | -8.15 | 39.65 | 0.4 | 0.5 | 0.5 | 0.8 | 0.9 | Severe |
| South East Asia | -8.12 | 114.63 | 0.8 | 0.8 | 0.9 | 0.3 | 0.9 | Severe |
| Western Indian Ocean | -8.12 | 39.70 | 0.5 | 0.5 | 0.6 | 0.8 | 0.9 | Severe |
| South East Asia | -8.10 | 115.06 | 0.9 | 0.8 | 0.9 | 0.2 | 0.9 | Severe |
| South East Asia | -8.10 | 114.90 | 0.7 | 0.8 | 0.8 | 0.4 | 0.9 | Severe |
| Western Indian Ocean | -8.08 | 39.60 | 0.4 | 0.5 | 0.5 | 0.8 | 0.9 | Severe |
| Great Barrier Reef | -8.07 | 156.80 | 0.8 | 0.9 | 0.9 | 0.5 | 0.9 | Severe |
| Western Indian Ocean | -8.05 | 39.60 | 0.4 | 0.5 | 0.5 | 0.8 | 0.9 | Severe |
| Western Indian Ocean | -7.98 | 39.78 | 0.7 | 0.5 | 0.7 | 0.3 | 0.8 | High |
| Western Indian Ocean | -7.97 | 39.78 | 0.7 | 0.5 | 0.7 | 0.3 | 0.8 | High |
| Western Indian Ocean | -7.83 | 39.83 | 0.6 | 0.5 | 0.6 | 0.8 | 0.9 | Severe |
| Western Indian Ocean | -7.67 | 40.67 | 0.5 | 0.5 | 0.6 | 0.1 | 0.6 | Medial |
| Central Indian Ocean | -7.50 | 72.50 | 0.7 | 0.8 | 0.8 | 0.2 | 0.8 | Severe |
| Central Indian Ocean | -7.37 | 72.42 | 0.7 | 0.8 | 0.8 | 0.2 | 0.8 | Severe |
| Western Indian Ocean | -7.03 | 52.76 | 0.4 | 0.5 | 0.5 | 0.3 | 0.7 | High |
| Western Indian Ocean | -7.01 | 52.73 | 0.4 | 0.5 | 0.5 | 0.3 | 0.7 | High |
| Western Indian Ocean | -7.01 | 52.73 | 0.5 | 0.5 | 0.6 | 0.4 | 0.7 | High |
| Central Indian Ocean | -7.00 | 71.00 | 0.8 | 0.8 | 0.9 | 0.2 | 0.9 | Severe |
| Western Indian Ocean | -6.70 | 39.26 | 0.6 | 0.5 | 0.6 | 0.6 | 0.9 | Severe |
| Western Indian Ocean | -6.68 | 39.25 | 0.5 | 0.5 | 0.5 | 0.6 | 0.8 | High |
| Western Indian Ocean | -6.67 | 39.22 | 0.7 | 0.5 | 0.7 | 0.6 | 0.9 | Severe |
| Central Indian Ocean | -6.66 | 71.35 | 0.6 | 0.9 | 0.8 | 0.4 | 0.9 | Severe |
| Western Indian Ocean | -6.66 | 39.25 | 0.5 | 0.5 | 0.5 | 0.6 | 0.8 | High |
| Western Indian Ocean | -6.65 | 39.50 | 0.4 | 0.5 | 0.5 | 0.5 | 0.7 | High |
| Western Indian Ocean | -6.43 | 39.30 | 0.4 | 0.5 | 0.5 | 0.8 | 0.9 | Severe |
| Western Indian Ocean | -6.43 | 39.35 | 0.5 | 0.5 | 0.6 | 0.8 | 0.9 | Severe |
| Western Indian Ocean | -6.35 | 39.23 | 0.6 | 0.5 | 0.6 | 0.7 | 0.9 | Severe |
| Western Indian Ocean | -6.30 | 39.20 | 0.6 | 0.5 | 0.6 | 0.7 | 0.9 | Severe |
| Western Indian Ocean | -6.27 | 39.18 | 0.6 | 0.4 | 0.6 | 0.6 | 0.9 | Severe |
| Western Indian Ocean | -6.25 | 39.17 | 0.6 | 0.4 | 0.6 | 0.6 | 0.9 | Severe |
| Central Indian Ocean | -6.23 | 72.00 | 0.5 | 0.9 | 0.7 | 0.3 | 0.8 | High |
| Western Indian Ocean | -6.23 | 39.17 | 0.7 | 0.4 | 0.7 | 0.6 | 0.9 | Severe |
| Western Indian Ocean | -6.19 | 39.13 | 0.6 | 0.5 | 0.7 | 0.6 | 0.9 | Severe |
| Western Indian Ocean | -6.13 | 39.17 | 0.6 | 0.5 | 0.6 | 0.6 | 0.9 | Severe |
| Central Indian Ocean | -6.10 | 71.73 | 0.5 | 0.9 | 0.7 | 0.2 | 0.8 | High |
| Western Indian Ocean | -6.08 | 39.08 | 0.6 | 0.5 | 0.6 | 0.6 | 0.9 | Severe |
| Western Indian Ocean | -6.07 | 39.11 | 0.6 | 0.5 | 0.7 | 0.6 | 0.9 | Severe |
| Western Indian Ocean | -6.07 | 39.10 | 0.6 | 0.5 | 0.6 | 0.6 | 0.9 | Severe |
| Western Indian Ocean | -6.00 | 39.02 | 0.7 | 0.5 | 0.7 | 0.6 | 0.9 | Severe |
| Western Indian Ocean | -6.00 | 39.55 | 0.6 | 0.5 | 0.6 | 0.3 | 0.7 | High |
| South East Asia | -5.90 | 110.50 | 0.8 | 1.0 | 0.8 | 0.5 | 0.9 | Severe |
| South East Asia | -5.87 | 106.62 | 0.5 | 0.9 | 0.6 | 1.0 | 1.0 | Severe |
| South East Asia | -5.87 | 110.33 | 0.7 | 1.0 | 0.8 | 0.5 | 0.9 | Severe |
| South East Asia | -5.83 | 106.63 | 0.5 | 0.9 | 0.7 | 0.9 | 1.0 | Severe |
| Western Indian Ocean | -5.80 | 39.50 | 0.6 | 0.5 | 0.6 | 0.5 | 0.8 | High |
| Western Indian Ocean | -5.65 | 39.10 | 0.5 | 0.5 | 0.6 | 0.6 | 0.8 | Severe |
| South East Asia | -5.52 | 106.48 | 0.5 | 0.9 | 0.6 | 1.0 | 1.0 | Severe |
| Western Indian Ocean | -5.50 | 39.07 | 0.6 | 0.5 | 0.6 | 0.7 | 0.9 | Severe |
| Central Indian Ocean | -5.48 | 72.48 | 0.6 | 0.9 | 0.8 | 0.2 | 0.8 | High |
| South East Asia | -5.48 | 106.40 | 0.6 | 0.9 | 0.7 | 1.0 | 1.0 | Severe |
| Central Indian Ocean | -5.48 | 72.48 | 0.6 | 0.9 | 0.8 | 0.2 | 0.8 | High |
| Central Indian Ocean | -5.35 | 71.75 | 0.5 | 0.9 | 0.6 | 0.2 | 0.7 | High |
| Central Indian Ocean | -5.35 | 72.24 | 0.6 | 0.9 | 0.7 | 0.2 | 0.7 | High |
| Western Indian Ocean | -5.28 | 39.63 | 0.6 | 0.5 | 0.6 | 0.8 | 0.9 | Severe |
| Great Barrier Reef | -5.26 | 151.02 | 0.8 | 0.9 | 0.8 | 0.2 | 0.9 | Severe |
| Central Indian Ocean | -5.25 | 72.47 | 0.6 | 0.9 | 0.7 | 0.3 | 0.8 | Severe |
| Great Barrier Reef | -5.24 | 151.02 | 0.8 | 0.9 | 0.8 | 0.2 | 0.9 | Severe |
| Western Indian Ocean | -5.23 | 39.60 | 0.6 | 0.5 | 0.6 | 0.9 | 1.0 | Severe |
| Great Barrier Reef | -5.20 | 150.52 | 0.8 | 0.9 | 0.9 | 0.2 | 0.9 | Severe |
| Western Indian Ocean | -5.15 | 39.35 | 0.5 | 0.5 | 0.6 | 0.7 | 0.9 | Severe |
| Great Barrier Reef | -5.15 | 150.57 | 0.8 | 0.9 | 0.9 | 0.2 | 0.9 | Severe |
| Western Indian Ocean | -5.14 | 39.35 | 0.5 | 0.5 | 0.5 | 0.7 | 0.9 | Severe |
| Western Indian Ocean | -5.14 | 39.35 | 0.5 | 0.5 | 0.5 | 0.7 | 0.9 | Severe |
| Great Barrier Reef | -4.90 | 151.06 | 0.8 | 0.9 | 0.9 | 0.3 | 0.9 | Severe |
| South East Asia | -4.80 | 119.30 | 0.8 | 1.0 | 0.8 | 0.7 | 1.0 | Severe |
| Western Indian Ocean | -4.77 | 55.50 | 0.5 | 0.7 | 0.6 | 0.5 | 0.8 | High |
| Western Indian Ocean | -4.71 | 39.37 | 0.6 | 0.5 | 0.6 | 0.9 | 1.0 | Severe |
| Western Indian Ocean | -4.71 | 39.37 | 0.6 | 0.5 | 0.6 | 0.9 | 1.0 | Severe |
| Western Indian Ocean | -4.63 | 55.37 | 0.5 | 0.7 | 0.6 | 0.4 | 0.8 | High |
| Western Indian Ocean | -4.62 | 55.37 | 0.5 | 0.7 | 0.6 | 0.4 | 0.8 | High |
| Western Indian Ocean | -4.50 | 55.50 | 0.5 | 0.7 | 0.6 | 0.4 | 0.8 | High |
| Western Indian Ocean | -4.45 | 55.32 | 0.4 | 0.7 | 0.6 | 0.4 | 0.8 | High |
| Western Indian Ocean | -4.43 | 55.32 | 0.5 | 0.7 | 0.6 | 0.4 | 0.8 | High |
| Western Indian Ocean | -4.41 | 55.26 | 0.5 | 0.7 | 0.6 | 0.4 | 0.8 | High |
| Western Indian Ocean | -4.40 | 55.24 | 0.5 | 0.7 | 0.6 | 0.4 | 0.8 | High |
| Western Indian Ocean | -4.39 | 55.29 | 0.4 | 0.7 | 0.6 | 0.4 | 0.7 | High |
| Western Indian Ocean | -4.38 | 55.22 | 0.5 | 0.7 | 0.6 | 0.4 | 0.7 | High |
| Western Indian Ocean | -4.37 | 55.30 | 0.4 | 0.7 | 0.6 | 0.4 | 0.7 | High |
| Western Indian Ocean | -4.37 | 55.31 | 0.4 | 0.7 | 0.6 | 0.4 | 0.7 | High |
| Western Indian Ocean | -4.36 | 55.25 | 0.5 | 0.7 | 0.6 | 0.4 | 0.8 | High |
| Western Indian Ocean | -4.36 | 55.30 | 0.4 | 0.7 | 0.6 | 0.4 | 0.7 | High |
| Western Indian Ocean | -4.35 | 55.63 | 0.5 | 0.8 | 0.6 | 0.5 | 0.8 | High |
| Western Indian Ocean | -4.35 | 55.65 | 0.6 | 0.8 | 0.7 | 0.5 | 0.8 | Severe |
| Western Indian Ocean | -4.35 | 55.83 | 0.5 | 0.8 | 0.6 | 0.4 | 0.8 | High |
| Western Indian Ocean | -4.33 | 55.87 | 0.6 | 0.8 | 0.7 | 0.4 | 0.8 | High |
| Western Indian Ocean | -4.33 | 55.28 | 0.4 | 0.7 | 0.6 | 0.4 | 0.7 | High |
| Western Indian Ocean | -4.30 | 55.72 | 0.5 | 0.8 | 0.7 | 0.4 | 0.8 | High |
| Western Indian Ocean | -4.30 | 55.92 | 0.5 | 0.8 | 0.6 | 0.4 | 0.8 | High |
| Western Indian Ocean | -4.28 | 55.87 | 0.5 | 0.8 | 0.6 | 0.4 | 0.8 | High |
| Western Indian Ocean | -4.27 | 55.73 | 0.4 | 0.8 | 0.6 | 0.4 | 0.7 | High |
| Western Indian Ocean | -4.21 | 55.42 | 0.5 | 0.7 | 0.6 | 0.3 | 0.7 | High |
| Western Indian Ocean | -4.20 | 55.39 | 0.4 | 0.7 | 0.6 | 0.4 | 0.7 | High |
| Western Indian Ocean | -4.20 | 55.40 | 0.4 | 0.7 | 0.6 | 0.4 | 0.7 | High |
| Western Indian Ocean | -4.20 | 55.41 | 0.4 | 0.7 | 0.6 | 0.4 | 0.7 | High |
| Western Indian Ocean | -4.19 | 55.40 | 0.4 | 0.7 | 0.6 | 0.4 | 0.7 | High |
| Western Indian Ocean | -4.19 | 55.41 | 0.4 | 0.7 | 0.6 | 0.4 | 0.7 | High |
| Western Indian Ocean | -4.19 | 55.45 | 0.5 | 0.7 | 0.6 | 0.3 | 0.7 | High |
| Western Indian Ocean | -4.18 | 55.44 | 0.5 | 0.7 | 0.6 | 0.3 | 0.7 | High |
| Western Indian Ocean | -4.17 | 55.43 | 0.4 | 0.7 | 0.6 | 0.3 | 0.7 | High |
| Western Indian Ocean | -4.00 | 55.02 | 0.5 | 0.7 | 0.6 | 0.4 | 0.8 | High |
| Western Indian Ocean | -4.00 | 55.05 | 0.5 | 0.7 | 0.6 | 0.4 | 0.7 | High |
| Western Indian Ocean | -4.00 | 55.07 | 0.5 | 0.7 | 0.6 | 0.3 | 0.7 | High |
| Western Indian Ocean | -4.00 | 55.08 | 0.5 | 0.7 | 0.6 | 0.3 | 0.7 | High |
| Western Indian Ocean | -4.00 | 55.12 | 0.5 | 0.7 | 0.6 | 0.3 | 0.7 | High |
| Western Indian Ocean | -4.00 | 55.13 | 0.5 | 0.7 | 0.6 | 0.3 | 0.7 | High |
| Polynesia | -4.00 | 173.00 | 0.8 | 0.8 | 0.9 | 0.1 | 0.9 | Severe |
| Western Indian Ocean | -3.59 | 39.93 | 0.8 | 0.6 | 0.7 | 0.6 | 0.9 | Severe |
| Western Indian Ocean | -3.48 | 39.95 | 0.6 | 0.5 | 0.6 | 0.6 | 0.8 | Severe |
| Western Indian Ocean | -3.37 | 40.48 | 0.5 | 0.6 | 0.6 | 0.8 | 0.9 | Severe |
| Western Indian Ocean | -3.23 | 40.67 | 0.5 | 0.6 | 0.6 | 0.8 | 0.9 | Severe |
| South East Asia | -2.63 | 99.91 | 0.9 | 0.9 | 0.9 | 0.3 | 0.9 | Severe |
| Western Indian Ocean | -2.50 | 45.50 | 0.8 | 0.6 | 0.8 | 0.4 | 0.9 | Severe |
| Western Indian Ocean | -2.25 | 41.06 | 0.5 | 0.6 | 0.6 | 0.9 | 1.0 | Severe |
| South East Asia | -2.22 | 99.51 | 0.9 | 1.0 | 0.9 | 0.4 | 0.9 | Severe |
| Western Indian Ocean | -1.99 | 41.38 | 0.6 | 0.6 | 0.7 | 0.9 | 1.0 | Severe |
| South East Asia | -1.96 | 99.46 | 0.9 | 1.0 | 0.9 | 0.3 | 0.9 | Severe |
| Eastern Pacific | -1.59 | -80.87 | 0.9 | 0.3 | 0.6 | 0.8 | 0.9 | Severe |
| Eastern Pacific | -1.28 | -81.07 | 0.9 | 0.3 | 0.6 | 0.8 | 0.9 | Severe |
| Eastern Pacific | -1.22 | -90.50 | 0.9 | 0.1 | 0.7 | 0.6 | 0.9 | Severe |
| Eastern Pacific | -1.22 | -90.39 | 0.9 | 0.1 | 0.7 | 0.5 | 0.8 | Severe |
| Eastern Pacific | -1.22 | -90.30 | 0.9 | 0.1 | 0.7 | 0.5 | 0.8 | Severe |
| Eastern Pacific | -0.89 | -90.41 | 0.8 | 0.1 | 0.7 | 0.6 | 0.9 | Severe |
| Eastern Pacific | -0.80 | -90.00 | 0.9 | 0.1 | 0.7 | 0.5 | 0.8 | Severe |
| Eastern Pacific | -0.80 | -90.10 | 0.9 | 0.1 | 0.7 | 0.6 | 0.9 | Severe |
| Eastern Pacific | -0.75 | -90.30 | 0.9 | 0.1 | 0.7 | 0.7 | 0.9 | Severe |
| Eastern Pacific | -0.75 | -90.77 | 0.9 | 0.1 | 0.7 | 0.7 | 0.9 | Severe |
| Eastern Pacific | -0.56 | -90.17 | 0.9 | 0.1 | 0.7 | 0.6 | 0.9 | Severe |
| Eastern Pacific | -0.50 | -90.20 | 0.9 | 0.1 | 0.7 | 0.5 | 0.8 | Severe |
| South East Asia | -0.33 | 122.00 | 0.8 | 0.9 | 0.9 | 0.1 | 0.9 | Severe |
| Eastern Pacific | -0.28 | -90.52 | 0.9 | 0.1 | 0.7 | 0.6 | 0.9 | Severe |
| Eastern Pacific | -0.27 | -91.10 | 0.8 | 0.1 | 0.7 | 0.6 | 0.9 | Severe |
| Brazilian province | 0.00 | -45.00 | 0.2 | 0.9 | 0.5 | 0.6 | 0.8 | Severe |
| Central Indian Ocean | 0.00 | 73.00 | 0.6 | 1.0 | 0.7 | 0.1 | 0.7 | High |
| Central Indian Ocean | 0.00 | 73.02 | 0.6 | 1.0 | 0.7 | 0.1 | 0.7 | High |
| Central Indian Ocean | 0.00 | 73.03 | 0.6 | 1.0 | 0.7 | 0.1 | 0.7 | High |
| Central Indian Ocean | 0.00 | 73.05 | 0.5 | 1.0 | 0.7 | 0.1 | 0.7 | High |
| Central Indian Ocean | 0.00 | 73.08 | 0.5 | 1.0 | 0.6 | 0.1 | 0.7 | High |
| Central Pacific | 0.22 | -176.52 | 1.0 | 0.7 | 0.9 | 0.3 | 0.9 | Severe |
| Eastern Pacific | 0.27 | -91.63 | 0.9 | 0.2 | 0.7 | 0.6 | 0.9 | Severe |
| Eastern Pacific | 0.40 | -90.40 | 0.9 | 0.2 | 0.7 | 0.4 | 0.8 | Severe |
| Eastern Pacific | 0.50 | -90.43 | 0.9 | 0.2 | 0.7 | 0.4 | 0.8 | Severe |
| Central Indian Ocean | 0.70 | 73.18 | 0.5 | 1.0 | 0.7 | 0.6 | 0.9 | Severe |
| Eastern Pacific | 0.77 | -90.40 | 0.9 | 0.2 | 0.7 | 0.4 | 0.8 | Severe |
| Central Pacific | 0.80 | -176.63 | 1.0 | 0.8 | 0.9 | 0.2 | 0.9 | Severe |
| South East Asia | 0.83 | 97.00 | 0.9 | 0.9 | 0.9 | 0.2 | 0.9 | Severe |
| South East Asia | 1.14 | 122.35 | 0.7 | 0.8 | 0.8 | 0.2 | 0.9 | Severe |
| South East Asia | 1.61 | 124.73 | 0.9 | 0.8 | 0.9 | 0.6 | 1.0 | Severe |
| South East Asia | 1.61 | 124.74 | 0.9 | 0.8 | 0.9 | 0.6 | 1.0 | Severe |
| South East Asia | 1.81 | 125.16 | 0.8 | 0.9 | 0.9 | 0.3 | 0.9 | Severe |
| South East Asia | 1.90 | 109.77 | 0.6 | 0.8 | 0.7 | 0.8 | 1.0 | Severe |
| Central Indian Ocean | 2.00 | 73.00 | 0.7 | 0.9 | 0.8 | 0.2 | 0.9 | Severe |
| Central Indian Ocean | 2.00 | 73.02 | 0.7 | 0.9 | 0.8 | 0.2 | 0.9 | Severe |
| South East Asia | 2.12 | 96.67 | 0.9 | 0.9 | 0.9 | 0.2 | 1.0 | Severe |
| South East Asia | 2.28 | 104.11 | 0.5 | 0.8 | 0.7 | 0.9 | 1.0 | Severe |
| South East Asia | 2.30 | 97.39 | 0.9 | 0.9 | 0.9 | 0.6 | 1.0 | Severe |
| South East Asia | 2.48 | 104.51 | 0.6 | 0.8 | 0.7 | 0.5 | 0.8 | Severe |
| Central Indian Ocean | 2.50 | 73.00 | 0.7 | 0.9 | 0.8 | 0.6 | 0.9 | Severe |
| South East Asia | 2.61 | 95.87 | 0.9 | 0.9 | 0.9 | 0.6 | 1.0 | Severe |
| South East Asia | 2.89 | 104.07 | 0.6 | 0.8 | 0.7 | 0.8 | 0.9 | Severe |
| South East Asia | 2.90 | 131.80 | 0.7 | 0.9 | 0.8 | 0.5 | 0.9 | Severe |
| South East Asia | 2.92 | 104.11 | 0.5 | 0.8 | 0.7 | 0.7 | 0.9 | Severe |
| Eastern Pacific | 2.95 | -78.21 | 0.9 | 0.8 | 0.8 | 0.8 | 1.0 | Severe |
| Eastern Pacific | 2.96 | -78.18 | 0.9 | 0.9 | 0.8 | 0.9 | 1.0 | Severe |
| Eastern Pacific | 2.97 | -78.17 | 0.9 | 0.9 | 0.8 | 0.9 | 1.0 | Severe |
| Eastern Pacific | 2.98 | -78.17 | 0.9 | 0.9 | 0.8 | 0.9 | 1.0 | Severe |
| Eastern Pacific | 2.98 | -78.20 | 0.9 | 0.8 | 0.8 | 0.8 | 1.0 | Severe |
| Eastern Pacific | 3.00 | -78.16 | 0.9 | 0.9 | 0.8 | 0.9 | 1.0 | Severe |
| Central Indian Ocean | 3.00 | 72.02 | 0.7 | 0.9 | 0.8 | 0.2 | 0.8 | Severe |
| Central Indian Ocean | 3.00 | 72.03 | 0.7 | 0.9 | 0.8 | 0.2 | 0.8 | Severe |
| Central Indian Ocean | 3.00 | 72.05 | 0.7 | 0.9 | 0.8 | 0.2 | 0.8 | Severe |
| Central Indian Ocean | 3.00 | 72.07 | 0.7 | 0.9 | 0.8 | 0.2 | 0.8 | Severe |
| Central Indian Ocean | 3.00 | 72.08 | 0.7 | 0.9 | 0.8 | 0.2 | 0.8 | Severe |
| Central Indian Ocean | 3.00 | 72.12 | 0.6 | 0.9 | 0.7 | 0.2 | 0.8 | High |
| Central Indian Ocean | 3.00 | 72.13 | 0.6 | 0.9 | 0.7 | 0.2 | 0.8 | High |
| Central Indian Ocean | 3.00 | 72.15 | 0.6 | 0.9 | 0.7 | 0.2 | 0.8 | High |
| Central Indian Ocean | 3.00 | 72.18 | 0.5 | 0.9 | 0.7 | 0.3 | 0.8 | High |
| Central Indian Ocean | 3.00 | 72.20 | 0.5 | 0.9 | 0.7 | 0.3 | 0.8 | High |
| Central Indian Ocean | 3.00 | 72.22 | 0.6 | 0.9 | 0.8 | 0.3 | 0.8 | Severe |
| Central Indian Ocean | 3.00 | 72.23 | 0.6 | 0.9 | 0.8 | 0.3 | 0.8 | Severe |
| Central Indian Ocean | 3.00 | 73.00 | 0.6 | 0.9 | 0.7 | 0.5 | 0.9 | Severe |
| Central Indian Ocean | 3.00 | 73.03 | 0.6 | 0.9 | 0.7 | 0.5 | 0.9 | Severe |
| Central Indian Ocean | 3.00 | 73.50 | 0.7 | 1.0 | 0.8 | 0.6 | 0.9 | Severe |
| South East Asia | 3.00 | 131.17 | 0.7 | 0.9 | 0.8 | 0.2 | 0.9 | Severe |
| Central Indian Ocean | 3.20 | 72.92 | 0.6 | 0.9 | 0.7 | 0.5 | 0.9 | Severe |
| Central Indian Ocean | 3.23 | 72.93 | 0.6 | 0.9 | 0.7 | 0.5 | 0.9 | Severe |
| Central Indian Ocean | 3.25 | 73.00 | 0.5 | 0.9 | 0.7 | 0.5 | 0.8 | Severe |
| Eastern Pacific | 3.30 | -78.00 | 0.9 | 0.8 | 0.8 | 0.6 | 0.9 | Severe |
| Central Indian Ocean | 3.43 | 73.42 | 0.5 | 1.0 | 0.7 | 0.7 | 0.9 | Severe |
| Central Indian Ocean | 3.43 | 73.45 | 0.5 | 1.0 | 0.7 | 0.7 | 0.9 | Severe |
| Central Indian Ocean | 3.45 | 73.75 | 0.7 | 1.0 | 0.8 | 0.7 | 0.9 | Severe |
| Central Indian Ocean | 3.48 | 73.48 | 0.6 | 1.0 | 0.7 | 0.6 | 0.9 | Severe |
| Central Indian Ocean | 3.50 | 73.00 | 0.6 | 0.9 | 0.7 | 0.5 | 0.9 | Severe |
| Central Indian Ocean | 3.50 | 73.50 | 0.6 | 1.0 | 0.7 | 0.6 | 0.9 | Severe |
| Central Indian Ocean | 3.63 | 72.17 | 0.5 | 0.9 | 0.7 | 0.2 | 0.8 | High |
| Central Indian Ocean | 3.63 | 72.18 | 0.5 | 0.9 | 0.7 | 0.2 | 0.8 | High |
| Central Indian Ocean | 3.63 | 72.20 | 0.5 | 0.9 | 0.7 | 0.2 | 0.8 | High |
| Central Indian Ocean | 3.63 | 72.22 | 0.5 | 0.9 | 0.6 | 0.2 | 0.7 | High |
| Central Indian Ocean | 3.63 | 72.23 | 0.5 | 0.9 | 0.6 | 0.2 | 0.7 | High |
| Central Indian Ocean | 3.64 | 72.93 | 0.5 | 0.9 | 0.6 | 0.8 | 0.9 | Severe |
| Central Indian Ocean | 3.70 | 73.12 | 0.6 | 0.9 | 0.7 | 0.4 | 0.8 | Severe |
| Central Indian Ocean | 3.79 | 72.71 | 0.6 | 0.9 | 0.7 | 0.8 | 0.9 | Severe |
| Central Indian Ocean | 3.82 | 73.42 | 0.6 | 1.0 | 0.7 | 0.8 | 0.9 | Severe |
| Central Indian Ocean | 3.88 | 72.84 | 0.6 | 0.9 | 0.7 | 0.8 | 0.9 | Severe |
| Eastern Pacific | 3.90 | -81.60 | 0.9 | 0.8 | 0.8 | 0.5 | 0.9 | Severe |
| Central Indian Ocean | 3.98 | 73.43 | 0.5 | 0.9 | 0.7 | 0.8 | 0.9 | Severe |
| Central Indian Ocean | 4.00 | 72.05 | 0.6 | 0.9 | 0.7 | 0.2 | 0.8 | High |
| Central Indian Ocean | 4.00 | 72.82 | 0.5 | 0.9 | 0.7 | 0.8 | 0.9 | Severe |
| Central Indian Ocean | 4.00 | 73.02 | 0.5 | 0.9 | 0.7 | 0.5 | 0.8 | Severe |
| Central Indian Ocean | 4.00 | 73.03 | 0.5 | 0.9 | 0.7 | 0.5 | 0.8 | Severe |
| Central Indian Ocean | 4.00 | 73.05 | 0.5 | 0.9 | 0.6 | 0.5 | 0.8 | High |
| Central Indian Ocean | 4.00 | 73.07 | 0.5 | 0.9 | 0.6 | 0.5 | 0.8 | High |
| Central Indian Ocean | 4.00 | 73.08 | 0.5 | 0.9 | 0.6 | 0.4 | 0.8 | High |
| Central Indian Ocean | 4.00 | 73.10 | 0.5 | 0.9 | 0.6 | 0.4 | 0.8 | High |
| Central Indian Ocean | 4.00 | 73.12 | 0.5 | 0.9 | 0.6 | 0.4 | 0.8 | High |
| Central Indian Ocean | 4.00 | 73.13 | 0.5 | 0.9 | 0.7 | 0.4 | 0.8 | High |
| Central Indian Ocean | 4.01 | 72.81 | 0.5 | 0.9 | 0.7 | 0.8 | 0.9 | Severe |
| Eastern Pacific | 4.01 | -77.63 | 0.9 | 0.8 | 0.8 | 0.9 | 1.0 | Severe |
| Central Indian Ocean | 4.12 | 73.47 | 0.4 | 0.9 | 0.6 | 0.8 | 0.9 | Severe |
| Central Indian Ocean | 4.17 | 73.50 | 0.6 | 0.9 | 0.7 | 0.8 | 0.9 | Severe |
| Central Indian Ocean | 4.17 | 72.83 | 0.5 | 0.9 | 0.7 | 0.8 | 0.9 | Severe |
| Central Indian Ocean | 4.20 | 73.40 | 0.5 | 0.9 | 0.7 | 0.8 | 0.9 | Severe |
| Central Indian Ocean | 4.20 | 73.50 | 0.5 | 0.9 | 0.7 | 0.8 | 0.9 | Severe |
| Central Indian Ocean | 4.21 | 73.05 | 0.6 | 0.9 | 0.7 | 0.4 | 0.8 | Severe |
| Central Indian Ocean | 4.26 | 72.98 | 0.5 | 0.9 | 0.7 | 0.4 | 0.8 | High |
| Central Indian Ocean | 4.26 | 72.99 | 0.5 | 0.9 | 0.7 | 0.4 | 0.8 | High |
| Central Indian Ocean | 4.26 | 73.00 | 0.5 | 0.9 | 0.6 | 0.4 | 0.8 | High |
| Central Indian Ocean | 4.27 | 73.00 | 0.5 | 0.9 | 0.6 | 0.4 | 0.8 | High |
| Central Indian Ocean | 4.27 | 73.01 | 0.5 | 0.9 | 0.6 | 0.4 | 0.8 | High |
| Central Indian Ocean | 4.27 | 72.77 | 0.4 | 0.9 | 0.6 | 0.9 | 0.9 | Severe |
| Central Indian Ocean | 4.28 | 73.47 | 0.5 | 0.9 | 0.6 | 0.8 | 0.9 | Severe |
| Central Indian Ocean | 4.29 | 73.36 | 0.5 | 0.9 | 0.7 | 0.9 | 1.0 | Severe |
| Central Indian Ocean | 4.30 | 73.35 | 0.5 | 0.9 | 0.7 | 0.9 | 1.0 | Severe |
| Central Indian Ocean | 4.30 | 73.43 | 0.4 | 0.9 | 0.6 | 0.8 | 0.9 | Severe |
| Central Indian Ocean | 4.30 | 73.43 | 0.5 | 0.9 | 0.6 | 0.8 | 0.9 | Severe |
| Central Indian Ocean | 4.30 | 73.48 | 0.5 | 0.9 | 0.6 | 0.8 | 0.9 | Severe |
| Central Indian Ocean | 4.30 | 73.50 | 0.5 | 0.9 | 0.6 | 0.8 | 0.9 | Severe |
| Central Indian Ocean | 4.32 | 73.58 | 0.5 | 0.9 | 0.7 | 0.7 | 0.9 | Severe |
| Central Indian Ocean | 4.33 | 73.37 | 0.5 | 0.9 | 0.6 | 0.9 | 1.0 | Severe |
| Central Indian Ocean | 4.34 | 73.84 | 0.5 | 0.9 | 0.7 | 0.3 | 0.8 | High |
| Central Indian Ocean | 4.36 | 72.98 | 0.7 | 0.9 | 0.8 | 0.4 | 0.9 | Severe |
| Central Indian Ocean | 4.42 | 73.50 | 0.5 | 0.9 | 0.6 | 0.7 | 0.9 | Severe |
| Central Indian Ocean | 4.49 | 73.47 | 0.5 | 0.9 | 0.7 | 0.7 | 0.9 | Severe |
| Central Indian Ocean | 4.49 | 73.59 | 0.5 | 0.9 | 0.7 | 0.6 | 0.9 | Severe |
| Central Indian Ocean | 4.49 | 73.01 | 0.6 | 0.9 | 0.8 | 0.5 | 0.9 | Severe |
| South East Asia | 4.49 | 118.61 | 0.9 | 0.8 | 0.9 | 0.7 | 1.0 | Severe |
| Central Indian Ocean | 4.50 | 73.00 | 0.6 | 0.9 | 0.8 | 0.5 | 0.9 | Severe |
| Central Indian Ocean | 4.56 | 73.64 | 0.5 | 0.9 | 0.7 | 0.5 | 0.8 | Severe |
| South East Asia | 4.57 | 118.76 | 0.7 | 0.8 | 0.8 | 0.6 | 0.9 | Severe |
| Central Indian Ocean | 4.61 | 73.62 | 0.4 | 0.9 | 0.6 | 0.4 | 0.8 | High |
| Central Indian Ocean | 4.61 | 73.84 | 0.6 | 0.9 | 0.7 | 0.3 | 0.8 | Severe |
| South East Asia | 4.62 | 118.78 | 0.6 | 0.8 | 0.8 | 0.5 | 0.9 | Severe |
| South East Asia | 4.65 | 118.83 | 0.6 | 0.8 | 0.7 | 0.5 | 0.9 | Severe |
| Central Indian Ocean | 4.74 | 73.50 | 0.6 | 0.9 | 0.7 | 0.6 | 0.9 | Severe |
| Central Indian Ocean | 5.00 | 73.02 | 0.5 | 0.9 | 0.7 | 0.8 | 0.9 | Severe |
| Central Indian Ocean | 5.00 | 73.03 | 0.5 | 0.9 | 0.7 | 0.8 | 0.9 | Severe |
| Central Indian Ocean | 5.00 | 73.05 | 0.6 | 0.9 | 0.7 | 0.8 | 0.9 | Severe |
| Central Indian Ocean | 5.00 | 73.07 | 0.6 | 0.9 | 0.7 | 0.8 | 0.9 | Severe |
| Micronesia | 5.00 | 170.00 | 0.7 | 0.9 | 0.8 | 0.0 | 0.8 | High |
| Central Indian Ocean | 5.02 | 72.92 | 0.5 | 0.9 | 0.7 | 0.8 | 0.9 | Severe |
| Central Indian Ocean | 5.30 | 73.33 | 0.5 | 0.9 | 0.7 | 0.4 | 0.8 | Severe |
| Central Indian Ocean | 5.30 | 73.34 | 0.5 | 0.9 | 0.7 | 0.4 | 0.8 | Severe |
| Central Indian Ocean | 5.31 | 73.32 | 0.5 | 0.9 | 0.7 | 0.4 | 0.8 | Severe |
| Central Indian Ocean | 5.32 | 73.33 | 0.5 | 0.9 | 0.7 | 0.4 | 0.8 | Severe |
| Central Indian Ocean | 5.40 | 73.60 | 0.5 | 0.9 | 0.7 | 0.5 | 0.8 | Severe |
| Central Indian Ocean | 5.50 | 73.50 | 0.4 | 0.9 | 0.6 | 0.5 | 0.8 | High |
| South East Asia | 5.54 | 125.22 | 0.7 | 0.8 | 0.8 | 0.1 | 0.8 | High |
| South East Asia | 5.58 | 124.87 | 0.7 | 0.8 | 0.8 | 0.1 | 0.8 | Severe |
| Central Indian Ocean | 5.60 | 73.40 | 0.6 | 0.9 | 0.7 | 0.5 | 0.8 | Severe |
| Central Indian Ocean | 5.60 | 79.90 | 0.6 | 0.9 | 0.7 | 0.7 | 0.9 | Severe |
| South East Asia | 5.73 | 103.00 | 0.6 | 0.7 | 0.7 | 0.5 | 0.8 | Severe |
| South East Asia | 5.74 | 103.03 | 0.6 | 0.7 | 0.7 | 0.4 | 0.8 | Severe |
| South East Asia | 5.77 | 103.06 | 0.6 | 0.7 | 0.7 | 0.4 | 0.8 | High |
| South East Asia | 5.78 | 103.00 | 0.7 | 0.7 | 0.8 | 0.4 | 0.9 | Severe |
| South East Asia | 5.85 | 118.33 | 0.9 | 0.9 | 0.9 | 0.9 | 1.0 | Severe |
| South East Asia | 5.85 | 95.27 | 0.7 | 0.9 | 0.7 | 0.5 | 0.9 | Severe |
| Central Pacific | 5.87 | -162.10 | 0.6 | 0.9 | 0.7 | 0.4 | 0.8 | Severe |
| Central Pacific | 5.87 | -162.07 | 0.6 | 0.9 | 0.7 | 0.4 | 0.8 | Severe |
| Central Indian Ocean | 5.90 | 80.00 | 0.5 | 0.9 | 0.7 | 0.8 | 0.9 | Severe |
| Central Indian Ocean | 5.90 | 80.90 | 0.5 | 1.0 | 0.7 | 1.0 | 1.0 | Severe |
| South East Asia | 5.90 | 102.78 | 0.6 | 0.8 | 0.7 | 0.5 | 0.8 | Severe |
| South East Asia | 5.91 | 95.22 | 0.6 | 0.9 | 0.7 | 0.5 | 0.8 | Severe |
| South East Asia | 5.92 | 102.76 | 0.6 | 0.8 | 0.7 | 0.5 | 0.8 | Severe |
| South East Asia | 5.96 | 102.76 | 0.6 | 0.8 | 0.7 | 0.5 | 0.9 | Severe |
| South East Asia | 5.97 | 116.00 | 0.7 | 0.8 | 0.8 | 0.8 | 1.0 | Severe |
| Micronesia | 5.99 | 169.60 | 0.5 | 0.9 | 0.6 | 0.1 | 0.7 | High |
| South East Asia | 6.00 | 96.00 | 0.8 | 0.9 | 0.9 | 0.3 | 0.9 | Severe |
| South East Asia | 6.00 | 116.03 | 0.7 | 0.8 | 0.8 | 0.8 | 1.0 | Severe |
| South East Asia | 6.02 | 116.03 | 0.7 | 0.8 | 0.8 | 0.8 | 1.0 | Severe |
| South East Asia | 6.04 | 99.93 | 0.7 | 0.8 | 0.8 | 1.0 | 1.0 | Severe |
| South East Asia | 6.05 | 100.04 | 0.8 | 0.8 | 0.8 | 1.0 | 1.0 | Severe |
| South East Asia | 6.05 | 100.04 | 0.8 | 0.8 | 0.8 | 1.0 | 1.0 | Severe |
| South East Asia | 6.06 | 100.04 | 0.8 | 0.8 | 0.8 | 1.0 | 1.0 | Severe |
| South East Asia | 6.06 | 100.04 | 0.8 | 0.8 | 0.8 | 1.0 | 1.0 | Severe |
| South East Asia | 6.06 | 100.04 | 0.8 | 0.8 | 0.8 | 1.0 | 1.0 | Severe |
| South East Asia | 6.07 | 100.06 | 0.8 | 0.8 | 0.8 | 1.0 | 1.0 | Severe |
| South East Asia | 6.07 | 100.05 | 0.8 | 0.8 | 0.8 | 1.0 | 1.0 | Severe |
| Central Indian Ocean | 6.10 | 81.29 | 0.6 | 1.0 | 0.7 | 1.0 | 1.0 | Severe |
| South East Asia | 6.27 | 124.13 | 0.7 | 0.8 | 0.8 | 0.1 | 0.8 | High |
| Central Pacific | 6.40 | -162.40 | 0.4 | 0.9 | 0.6 | 0.1 | 0.7 | Medial |
| Eastern Pacific | 6.40 | -77.42 | 0.8 | 0.7 | 0.8 | 0.8 | 0.9 | Severe |
| South East Asia | 6.44 | 99.69 | 0.7 | 0.9 | 0.8 | 1.0 | 1.0 | Severe |
| Central Indian Ocean | 6.50 | 73.00 | 0.5 | 0.9 | 0.7 | 0.7 | 0.9 | Severe |
| South East Asia | 6.50 | 99.30 | 0.8 | 0.8 | 0.9 | 0.7 | 1.0 | Severe |
| South East Asia | 6.50 | 99.30 | 0.8 | 0.8 | 0.9 | 0.7 | 1.0 | Severe |
| South East Asia | 6.53 | 99.28 | 0.8 | 0.8 | 0.8 | 0.7 | 0.9 | Severe |
| South East Asia | 6.53 | 99.28 | 0.8 | 0.8 | 0.8 | 0.7 | 0.9 | Severe |
| South East Asia | 6.63 | 122.47 | 0.8 | 0.8 | 0.8 | 0.4 | 0.9 | Severe |
| Central Indian Ocean | 6.85 | 79.70 | 0.6 | 0.9 | 0.7 | 0.7 | 0.9 | Severe |
| Central Indian Ocean | 6.90 | 72.00 | 0.5 | 1.0 | 0.6 | 0.2 | 0.7 | High |
| Caribbean | 7.00 | -74.00 | 0.7 | 0.9 | 0.8 | 0.6 | 0.9 | Severe |
| Central Indian Ocean | 7.00 | 79.50 | 0.6 | 0.9 | 0.7 | 0.7 | 0.9 | Severe |
| Central Indian Ocean | 7.00 | 79.70 | 0.5 | 0.9 | 0.6 | 0.8 | 0.9 | Severe |
| South East Asia | 7.00 | 94.00 | 0.6 | 0.9 | 0.7 | 0.3 | 0.8 | High |
| South East Asia | 7.00 | 98.02 | 0.7 | 0.8 | 0.8 | 0.2 | 0.8 | Severe |
| South East Asia | 7.00 | 98.03 | 0.7 | 0.8 | 0.8 | 0.2 | 0.8 | Severe |
| Micronesia | 7.00 | 134.22 | 0.8 | 0.9 | 0.9 | 0.8 | 1.0 | Severe |
| Micronesia | 7.01 | 171.37 | 0.5 | 0.9 | 0.7 | 0.0 | 0.7 | High |
| Micronesia | 7.08 | 171.39 | 0.4 | 0.9 | 0.6 | 0.2 | 0.7 | Medial |
| Micronesia | 7.08 | 171.14 | 0.4 | 0.9 | 0.6 | 0.6 | 0.8 | Severe |
| Micronesia | 7.09 | 171.20 | 0.4 | 0.9 | 0.6 | 0.2 | 0.7 | High |
| Micronesia | 7.10 | 171.16 | 0.4 | 0.9 | 0.6 | 0.6 | 0.8 | Severe |
| Micronesia | 7.11 | 134.25 | 0.8 | 0.9 | 0.9 | 0.4 | 0.9 | Severe |
| Micronesia | 7.11 | 134.26 | 0.8 | 0.9 | 0.9 | 0.4 | 0.9 | Severe |
| Micronesia | 7.13 | 134.23 | 0.9 | 0.9 | 0.9 | 0.3 | 0.9 | Severe |
| Micronesia | 7.14 | 171.21 | 0.4 | 0.9 | 0.6 | 0.2 | 0.7 | High |
| Micronesia | 7.16 | 171.07 | 0.5 | 0.9 | 0.7 | 0.5 | 0.8 | Severe |
| Micronesia | 7.16 | 134.35 | 0.8 | 0.9 | 0.8 | 0.5 | 0.9 | Severe |
| Micronesia | 7.19 | 134.37 | 0.8 | 0.9 | 0.8 | 0.5 | 0.9 | Severe |
| Micronesia | 7.21 | 134.23 | 0.8 | 0.9 | 0.9 | 0.4 | 0.9 | Severe |
| Micronesia | 7.25 | 134.25 | 0.8 | 0.9 | 0.9 | 0.7 | 1.0 | Severe |
| Micronesia | 7.26 | 134.38 | 0.8 | 0.9 | 0.9 | 0.6 | 0.9 | Severe |
| Micronesia | 7.27 | 134.35 | 0.8 | 0.9 | 0.9 | 0.6 | 0.9 | Severe |
| Micronesia | 7.28 | 134.23 | 0.8 | 0.9 | 0.9 | 0.7 | 1.0 | Severe |
| Eastern Pacific | 7.30 | -81.80 | 0.7 | 0.9 | 0.7 | 0.8 | 0.9 | Severe |
| Micronesia | 7.30 | 134.35 | 0.8 | 0.9 | 0.8 | 0.6 | 0.9 | Severe |
| Micronesia | 7.31 | 134.49 | 0.8 | 0.9 | 0.9 | 0.6 | 0.9 | Severe |
| Micronesia | 7.32 | 134.23 | 0.8 | 0.9 | 0.9 | 0.7 | 1.0 | Severe |
| Micronesia | 7.32 | 134.51 | 0.8 | 0.9 | 0.9 | 0.6 | 0.9 | Severe |
| Micronesia | 7.33 | 134.50 | 0.8 | 0.9 | 0.9 | 0.6 | 0.9 | Severe |
| Micronesia | 7.35 | 134.45 | 0.8 | 0.9 | 0.9 | 0.6 | 1.0 | Severe |
| Micronesia | 7.43 | 134.72 | 0.8 | 0.9 | 0.9 | 0.0 | 0.9 | Severe |
| Micronesia | 7.44 | 134.30 | 0.8 | 0.9 | 0.8 | 0.3 | 0.9 | Severe |
| Central Indian Ocean | 7.46 | 81.89 | 0.4 | 1.0 | 0.6 | 0.5 | 0.8 | High |
| Eastern Pacific | 7.48 | -82.25 | 0.6 | 0.9 | 0.7 | 0.7 | 0.9 | Severe |
| Micronesia | 7.50 | 134.50 | 0.9 | 0.9 | 0.9 | 0.9 | 1.0 | Severe |
| South East Asia | 7.51 | 93.50 | 0.6 | 0.9 | 0.7 | 0.4 | 0.8 | High |
| South East Asia | 7.54 | 98.39 | 0.8 | 0.8 | 0.8 | 0.6 | 0.9 | Severe |
| Micronesia | 7.55 | 134.47 | 0.9 | 0.7 | 0.9 | 1.0 | 1.0 | Severe |
| Micronesia | 7.57 | 134.70 | 0.9 | 0.9 | 0.9 | 0.0 | 0.9 | Severe |
| South East Asia | 7.60 | 98.38 | 0.8 | 0.8 | 0.8 | 0.6 | 0.9 | Severe |
| South East Asia | 7.61 | 98.39 | 0.8 | 0.8 | 0.8 | 0.7 | 1.0 | Severe |
| South East Asia | 7.62 | 98.38 | 0.8 | 0.8 | 0.8 | 0.7 | 1.0 | Severe |
| South East Asia | 7.67 | 98.77 | 0.8 | 0.8 | 0.8 | 0.7 | 1.0 | Severe |
| South East Asia | 7.68 | 98.59 | 0.8 | 0.8 | 0.8 | 0.7 | 0.9 | Severe |
| Eastern Pacific | 7.70 | -82.06 | 0.6 | 0.9 | 0.7 | 0.8 | 1.0 | Severe |
| Micronesia | 7.75 | 134.58 | 0.8 | 0.9 | 0.8 | 0.4 | 0.9 | Severe |
| Eastern Pacific | 7.79 | -81.76 | 0.6 | 0.9 | 0.7 | 0.8 | 0.9 | Severe |
| Eastern Pacific | 7.80 | -81.75 | 0.6 | 0.9 | 0.7 | 0.8 | 0.9 | Severe |
| Eastern Pacific | 7.80 | -80.10 | 0.8 | 0.8 | 0.8 | 0.9 | 1.0 | Severe |
| Central Indian Ocean | 7.80 | 79.60 | 0.4 | 0.9 | 0.6 | 0.9 | 0.9 | Severe |
| Eastern Pacific | 7.82 | -81.77 | 0.6 | 0.9 | 0.7 | 0.8 | 0.9 | Severe |
| Eastern Pacific | 7.85 | -82.46 | 0.6 | 0.9 | 0.7 | 0.8 | 1.0 | Severe |
| Eastern Pacific | 7.94 | -82.05 | 0.6 | 0.9 | 0.7 | 1.0 | 1.0 | Severe |
| Eastern Pacific | 7.95 | -82.02 | 0.6 | 0.9 | 0.7 | 0.9 | 1.0 | Severe |
| Eastern Pacific | 8.00 | -82.33 | 0.5 | 0.9 | 0.6 | 0.9 | 1.0 | Severe |
| South East Asia | 8.10 | 98.30 | 0.7 | 0.8 | 0.7 | 0.4 | 0.8 | Severe |
| Eastern Pacific | 8.10 | -81.90 | 0.6 | 0.9 | 0.7 | 0.9 | 1.0 | Severe |
| South East Asia | 8.10 | 98.30 | 0.7 | 0.8 | 0.7 | 0.4 | 0.8 | Severe |
| South East Asia | 8.10 | 98.30 | 0.7 | 0.8 | 0.7 | 0.4 | 0.8 | Severe |
| Micronesia | 8.15 | 134.62 | 0.8 | 0.9 | 0.9 | 0.5 | 0.9 | Severe |
| Micronesia | 8.16 | 134.63 | 0.8 | 0.9 | 0.9 | 0.5 | 0.9 | Severe |
| Micronesia | 8.17 | 134.63 | 0.8 | 0.9 | 0.9 | 0.5 | 0.9 | Severe |
| Micronesia | 8.17 | 134.65 | 0.7 | 0.9 | 0.8 | 0.1 | 0.8 | Severe |
| Eastern Pacific | 8.25 | -80.37 | 0.7 | 0.8 | 0.8 | 0.9 | 1.0 | Severe |
| Central Indian Ocean | 8.41 | 79.75 | 0.4 | 0.9 | 0.6 | 1.0 | 1.0 | Severe |
| Central Indian Ocean | 8.43 | 79.70 | 0.4 | 0.9 | 0.6 | 1.0 | 1.0 | Severe |
| South East Asia | 8.47 | 97.66 | 0.7 | 0.8 | 0.8 | 0.4 | 0.8 | Severe |
| Central Indian Ocean | 8.50 | 73.00 | 0.5 | 0.9 | 0.6 | 0.3 | 0.7 | High |
| Central Indian Ocean | 8.50 | 79.50 | 0.5 | 0.9 | 0.6 | 0.9 | 1.0 | Severe |
| Central Indian Ocean | 8.50 | 81.50 | 0.3 | 0.9 | 0.5 | 0.5 | 0.7 | High |
| South East Asia | 8.50 | 93.50 | 0.5 | 0.9 | 0.7 | 0.3 | 0.8 | High |
| South East Asia | 8.50 | 97.67 | 0.6 | 0.8 | 0.7 | 0.3 | 0.8 | Severe |
| South East Asia | 8.51 | 97.76 | 0.7 | 0.8 | 0.8 | 0.3 | 0.8 | Severe |
| South East Asia | 8.52 | 97.85 | 0.8 | 0.8 | 0.8 | 0.3 | 0.9 | Severe |
| Central Indian Ocean | 8.52 | 81.32 | 0.5 | 0.9 | 0.6 | 0.8 | 0.9 | Severe |
| South East Asia | 8.58 | 97.77 | 0.7 | 0.8 | 0.8 | 0.3 | 0.8 | Severe |
| South East Asia | 8.59 | 97.56 | 0.7 | 0.8 | 0.8 | 0.3 | 0.8 | Severe |
| Micronesia | 8.61 | 150.39 | 0.7 | 1.0 | 0.8 | 0.3 | 0.9 | Severe |
| Eastern Pacific | 8.62 | -79.05 | 0.8 | 0.8 | 0.8 | 1.0 | 1.0 | Severe |
| Eastern Pacific | 8.63 | -79.08 | 0.8 | 0.8 | 0.8 | 1.0 | 1.0 | Severe |
| Eastern Pacific | 8.63 | -79.01 | 0.8 | 0.8 | 0.8 | 1.0 | 1.0 | Severe |
| Eastern Pacific | 8.63 | -79.07 | 0.8 | 0.8 | 0.8 | 1.0 | 1.0 | Severe |
| South East Asia | 8.64 | 111.92 | 0.5 | 0.8 | 0.6 | 0.2 | 0.7 | High |
| South East Asia | 8.65 | 106.67 | 0.6 | 0.5 | 0.6 | 1.0 | 1.0 | Severe |
| South East Asia | 8.66 | 97.65 | 0.8 | 0.8 | 0.8 | 0.3 | 0.9 | Severe |
| South East Asia | 8.66 | 97.64 | 0.8 | 0.8 | 0.8 | 0.3 | 0.9 | Severe |
| South East Asia | 8.66 | 97.65 | 0.7 | 0.8 | 0.8 | 0.3 | 0.9 | Severe |
| South East Asia | 8.66 | 97.69 | 0.7 | 0.8 | 0.8 | 0.3 | 0.9 | Severe |
| South East Asia | 8.67 | 106.67 | 0.8 | 0.5 | 0.7 | 1.0 | 1.0 | Severe |
| South East Asia | 8.67 | 106.56 | 0.7 | 0.5 | 0.7 | 1.0 | 1.0 | Severe |
| South East Asia | 8.68 | 97.66 | 0.7 | 0.8 | 0.8 | 0.3 | 0.9 | Severe |
| Eastern Pacific | 8.69 | -87.01 | 0.9 | 0.7 | 0.9 | 0.5 | 0.9 | Severe |
| South East Asia | 8.71 | 106.66 | 0.7 | 0.5 | 0.7 | 1.0 | 1.0 | Severe |
| South East Asia | 8.71 | 106.65 | 0.7 | 0.5 | 0.7 | 1.0 | 1.0 | Severe |
| Central Indian Ocean | 8.71 | 81.20 | 0.4 | 0.9 | 0.6 | 0.6 | 0.8 | High |
| Eastern Pacific | 8.72 | -83.88 | 0.7 | 0.9 | 0.8 | 0.7 | 0.9 | Severe |
| Eastern Pacific | 8.72 | -83.87 | 0.7 | 0.9 | 0.8 | 0.7 | 0.9 | Severe |
| Eastern Pacific | 8.72 | -83.88 | 0.7 | 0.9 | 0.8 | 0.7 | 0.9 | Severe |
| South East Asia | 8.72 | 119.80 | 0.7 | 0.8 | 0.7 | 0.2 | 0.8 | High |
| Eastern Pacific | 8.73 | -83.90 | 0.7 | 0.9 | 0.7 | 0.7 | 0.9 | Severe |
| Eastern Pacific | 8.73 | -83.89 | 0.7 | 0.9 | 0.8 | 0.7 | 0.9 | Severe |
| Eastern Pacific | 8.73 | -83.88 | 0.7 | 0.9 | 0.8 | 0.7 | 0.9 | Severe |
| Eastern Pacific | 8.73 | -83.88 | 0.7 | 0.9 | 0.8 | 0.7 | 0.9 | Severe |
| South East Asia | 8.74 | 97.77 | 0.7 | 0.8 | 0.8 | 0.3 | 0.8 | Severe |
| South East Asia | 8.75 | 97.78 | 0.7 | 0.8 | 0.8 | 0.3 | 0.8 | Severe |
| South East Asia | 8.76 | 97.74 | 0.7 | 0.8 | 0.8 | 0.3 | 0.9 | Severe |
| Eastern Pacific | 8.78 | -79.53 | 0.8 | 0.8 | 0.8 | 1.0 | 1.0 | Severe |
| Eastern Pacific | 8.80 | -79.57 | 0.9 | 0.8 | 0.8 | 1.0 | 1.0 | Severe |
| Central Indian Ocean | 8.87 | 78.65 | 0.4 | 0.9 | 0.6 | 1.0 | 1.0 | Severe |
| South East Asia | 8.89 | 97.77 | 0.7 | 0.8 | 0.8 | 0.3 | 0.8 | Severe |
| South East Asia | 8.90 | 97.89 | 0.6 | 0.8 | 0.7 | 0.3 | 0.8 | High |
| South East Asia | 8.90 | 97.88 | 0.6 | 0.8 | 0.7 | 0.3 | 0.8 | High |
| South East Asia | 8.94 | 106.95 | 0.6 | 0.4 | 0.6 | 1.0 | 1.0 | Severe |
| South East Asia | 8.99 | 106.91 | 0.6 | 0.4 | 0.6 | 1.0 | 1.0 | Severe |
| Eastern Pacific | 9.00 | -85.00 | 0.8 | 0.8 | 0.8 | 0.4 | 0.9 | Severe |
| Central Indian Ocean | 9.00 | 79.00 | 0.4 | 0.9 | 0.6 | 0.9 | 1.0 | Severe |
| South East Asia | 9.00 | 124.00 | 0.8 | 0.9 | 0.8 | 0.2 | 0.9 | Severe |
| South East Asia | 9.07 | 123.27 | 0.7 | 0.9 | 0.7 | 0.3 | 0.8 | Severe |
| South East Asia | 9.08 | 123.27 | 0.7 | 0.9 | 0.7 | 0.3 | 0.8 | Severe |
| South East Asia | 9.08 | 123.27 | 0.7 | 0.9 | 0.7 | 0.3 | 0.8 | Severe |
| South East Asia | 9.08 | 123.27 | 0.7 | 0.9 | 0.7 | 0.3 | 0.8 | Severe |
| South East Asia | 9.09 | 118.23 | 0.5 | 0.9 | 0.6 | 0.6 | 0.8 | Severe |
| Caribbean | 9.11 | -81.82 | 0.9 | 1.0 | 0.8 | 0.8 | 1.0 | Severe |
| Caribbean | 9.11 | -81.08 | 0.8 | 1.0 | 0.8 | 0.3 | 0.9 | Severe |
| Caribbean | 9.11 | -81.82 | 0.9 | 1.0 | 0.8 | 0.8 | 1.0 | Severe |
| Caribbean | 9.19 | -82.08 | 0.9 | 1.0 | 0.9 | 0.8 | 1.0 | Severe |
| Micronesia | 9.20 | 167.66 | 0.3 | 0.9 | 0.6 | 0.0 | 0.6 | Medial |
| South East Asia | 9.20 | 123.34 | 0.7 | 0.9 | 0.8 | 0.2 | 0.8 | Severe |
| Central Indian Ocean | 9.23 | 79.20 | 0.7 | 0.9 | 0.8 | 0.8 | 0.9 | Severe |
| Caribbean | 9.24 | -82.11 | 0.9 | 1.0 | 0.9 | 0.8 | 1.0 | Severe |
| Caribbean | 9.25 | -82.03 | 0.9 | 1.0 | 0.8 | 0.8 | 1.0 | Severe |
| Caribbean | 9.27 | -82.12 | 0.9 | 1.0 | 0.9 | 0.8 | 1.0 | Severe |
| Caribbean | 9.27 | -82.12 | 0.9 | 1.0 | 0.9 | 0.8 | 1.0 | Severe |
| Caribbean | 9.27 | -82.01 | 0.9 | 1.0 | 0.8 | 0.8 | 1.0 | Severe |
| Caribbean | 9.27 | -82.07 | 0.9 | 1.0 | 0.8 | 0.8 | 1.0 | Severe |
| Central Indian Ocean | 9.29 | 79.17 | 0.6 | 0.9 | 0.7 | 0.9 | 1.0 | Severe |
| Central Indian Ocean | 9.29 | 79.14 | 0.7 | 0.9 | 0.8 | 0.9 | 1.0 | Severe |
| Central Indian Ocean | 9.29 | 79.14 | 0.7 | 0.9 | 0.8 | 0.9 | 1.0 | Severe |
| Central Indian Ocean | 9.29 | 79.19 | 0.6 | 0.9 | 0.7 | 0.9 | 1.0 | Severe |
| Central Indian Ocean | 9.29 | 79.33 | 0.5 | 0.9 | 0.7 | 0.9 | 1.0 | Severe |
| Central Indian Ocean | 9.29 | 79.25 | 0.7 | 0.9 | 0.8 | 1.0 | 1.0 | Severe |
| Caribbean | 9.30 | -82.26 | 0.9 | 1.0 | 0.9 | 0.8 | 1.0 | Severe |
| Central Indian Ocean | 9.33 | 79.32 | 0.5 | 0.9 | 0.7 | 0.9 | 1.0 | Severe |
| Micronesia | 9.34 | 167.45 | 0.4 | 0.9 | 0.6 | 0.0 | 0.6 | Medial |
| South East Asia | 9.37 | 97.87 | 0.7 | 0.8 | 0.7 | 0.4 | 0.9 | Severe |
| South East Asia | 9.38 | 97.88 | 0.7 | 0.8 | 0.7 | 0.4 | 0.9 | Severe |
| South East Asia | 9.39 | 97.89 | 0.7 | 0.8 | 0.7 | 0.4 | 0.9 | Severe |
| South East Asia | 9.40 | 97.90 | 0.7 | 0.8 | 0.7 | 0.4 | 0.9 | Severe |
| Caribbean | 9.42 | -82.32 | 0.9 | 1.0 | 0.8 | 0.8 | 1.0 | Severe |
| Caribbean | 9.42 | -82.32 | 0.9 | 1.0 | 0.8 | 0.8 | 1.0 | Severe |
| Caribbean | 9.43 | -78.59 | 0.8 | 0.9 | 0.8 | 0.3 | 0.8 | Severe |
| South East Asia | 9.43 | 98.07 | 0.6 | 0.8 | 0.7 | 0.5 | 0.8 | Severe |
| South East Asia | 9.43 | 123.95 | 0.8 | 0.9 | 0.8 | 0.4 | 0.9 | Severe |
| Caribbean | 9.46 | -78.73 | 0.6 | 0.9 | 0.7 | 0.3 | 0.8 | High |
| Caribbean | 9.47 | -78.92 | 0.7 | 0.9 | 0.7 | 0.5 | 0.8 | Severe |
| Caribbean | 9.48 | -78.93 | 0.7 | 0.9 | 0.7 | 0.5 | 0.9 | Severe |
| Caribbean | 9.48 | -78.82 | 0.7 | 0.9 | 0.7 | 0.4 | 0.8 | Severe |
| Caribbean | 9.49 | -78.88 | 0.7 | 0.9 | 0.7 | 0.5 | 0.8 | Severe |
| Caribbean | 9.49 | -78.73 | 0.6 | 0.9 | 0.7 | 0.3 | 0.8 | High |
| South East Asia | 9.49 | 98.01 | 0.6 | 0.8 | 0.7 | 0.4 | 0.8 | Severe |
| Caribbean | 9.49 | -78.96 | 0.6 | 0.9 | 0.7 | 0.5 | 0.8 | Severe |
| Caribbean | 9.50 | -78.72 | 0.7 | 0.9 | 0.7 | 0.3 | 0.8 | High |
| Central Indian Ocean | 9.50 | 79.50 | 0.5 | 0.9 | 0.7 | 0.9 | 1.0 | Severe |
| South East Asia | 9.50 | 93.00 | 0.6 | 0.9 | 0.7 | 0.3 | 0.8 | High |
| South East Asia | 9.50 | 97.90 | 0.8 | 0.9 | 0.8 | 0.4 | 0.9 | Severe |
| Caribbean | 9.51 | -78.80 | 0.6 | 0.9 | 0.6 | 0.4 | 0.8 | High |
| Caribbean | 9.52 | -78.70 | 0.6 | 0.9 | 0.7 | 0.3 | 0.8 | High |
| Caribbean | 9.52 | -78.04 | 0.7 | 0.9 | 0.7 | 0.2 | 0.8 | High |
| South East Asia | 9.52 | 123.68 | 0.8 | 0.8 | 0.8 | 0.3 | 0.9 | Severe |
| Caribbean | 9.52 | -78.66 | 0.7 | 0.9 | 0.7 | 0.3 | 0.8 | High |
| South East Asia | 9.52 | 97.91 | 0.8 | 0.9 | 0.8 | 0.4 | 0.9 | Severe |
| Caribbean | 9.52 | -78.65 | 0.7 | 0.9 | 0.7 | 0.3 | 0.8 | High |
| South East Asia | 9.53 | 98.09 | 0.5 | 0.9 | 0.7 | 0.5 | 0.8 | Severe |
| South East Asia | 9.53 | 97.89 | 0.8 | 0.9 | 0.8 | 0.4 | 0.9 | Severe |
| South East Asia | 9.53 | 98.11 | 0.5 | 0.9 | 0.7 | 0.5 | 0.8 | Severe |
| South East Asia | 9.53 | 98.05 | 0.6 | 0.9 | 0.7 | 0.5 | 0.8 | Severe |
| South East Asia | 9.54 | 97.91 | 0.7 | 0.8 | 0.7 | 0.4 | 0.9 | Severe |
| Caribbean | 9.54 | -78.90 | 0.7 | 0.9 | 0.7 | 0.5 | 0.8 | Severe |
| Caribbean | 9.54 | -78.90 | 0.7 | 0.9 | 0.7 | 0.5 | 0.8 | Severe |
| Caribbean | 9.55 | -78.91 | 0.7 | 0.9 | 0.7 | 0.5 | 0.8 | Severe |
| Caribbean | 9.55 | -78.98 | 0.6 | 0.9 | 0.6 | 0.4 | 0.8 | High |
| Caribbean | 9.55 | -78.87 | 0.6 | 0.9 | 0.7 | 0.4 | 0.8 | High |
| Caribbean | 9.55 | -78.87 | 0.6 | 0.9 | 0.7 | 0.4 | 0.8 | High |
| Caribbean | 9.55 | -78.95 | 0.6 | 0.9 | 0.7 | 0.5 | 0.8 | Severe |
| South East Asia | 9.55 | 98.00 | 0.7 | 0.8 | 0.7 | 0.5 | 0.9 | Severe |
| Caribbean | 9.55 | -78.94 | 0.6 | 0.9 | 0.7 | 0.5 | 0.8 | Severe |
| South East Asia | 9.56 | 123.66 | 0.8 | 0.8 | 0.8 | 0.2 | 0.9 | Severe |
| Caribbean | 9.56 | -78.88 | 0.7 | 0.9 | 0.7 | 0.5 | 0.8 | Severe |
| Caribbean | 9.56 | -78.87 | 0.6 | 0.9 | 0.7 | 0.4 | 0.8 | High |
| Caribbean | 9.56 | -78.88 | 0.6 | 0.9 | 0.7 | 0.4 | 0.8 | High |
| Caribbean | 9.56 | -78.88 | 0.6 | 0.9 | 0.7 | 0.4 | 0.8 | High |
| Caribbean | 9.56 | -78.84 | 0.6 | 0.9 | 0.7 | 0.4 | 0.8 | High |
| Caribbean | 9.57 | -78.85 | 0.6 | 0.9 | 0.7 | 0.4 | 0.8 | High |
| South East Asia | 9.57 | 98.02 | 0.7 | 0.8 | 0.7 | 0.5 | 0.9 | Severe |
| South East Asia | 9.57 | 98.13 | 0.6 | 0.9 | 0.7 | 0.5 | 0.8 | Severe |
| Caribbean | 9.57 | -78.87 | 0.6 | 0.9 | 0.7 | 0.4 | 0.8 | High |
| Caribbean | 9.58 | -78.68 | 0.6 | 0.9 | 0.7 | 0.3 | 0.8 | High |
| Caribbean | 9.58 | -78.87 | 0.6 | 0.9 | 0.7 | 0.4 | 0.8 | High |
| South East Asia | 9.58 | 98.06 | 0.5 | 0.9 | 0.7 | 0.5 | 0.8 | Severe |
| South East Asia | 9.59 | 97.99 | 0.7 | 0.9 | 0.8 | 0.5 | 0.9 | Severe |
| Caribbean | 9.59 | -78.77 | 0.6 | 0.9 | 0.7 | 0.3 | 0.8 | High |
| Caribbean | 9.60 | -78.78 | 0.6 | 0.9 | 0.7 | 0.3 | 0.8 | High |
| South East Asia | 9.60 | 98.00 | 0.6 | 0.9 | 0.7 | 0.5 | 0.8 | Severe |
| South East Asia | 9.63 | 98.03 | 0.6 | 0.9 | 0.7 | 0.5 | 0.8 | Severe |
| Caribbean | 9.63 | -82.68 | 0.8 | 1.0 | 0.8 | 0.8 | 1.0 | Severe |
| South East Asia | 9.64 | 97.86 | 0.7 | 0.8 | 0.7 | 0.4 | 0.8 | Severe |
| South East Asia | 9.65 | 98.10 | 0.5 | 0.9 | 0.7 | 0.5 | 0.8 | Severe |
| Caribbean | 9.65 | -74.67 | 0.6 | 0.8 | 0.7 | 0.6 | 0.9 | Severe |
| South East Asia | 9.67 | 123.55 | 0.8 | 0.7 | 0.9 | 0.2 | 0.9 | Severe |
| Caribbean | 9.75 | -75.83 | 0.6 | 0.9 | 0.7 | 0.6 | 0.9 | Severe |
| South East Asia | 9.76 | 123.74 | 0.8 | 0.8 | 0.9 | 0.3 | 0.9 | Severe |
| South East Asia | 9.78 | 98.11 | 0.6 | 0.9 | 0.7 | 0.5 | 0.8 | Severe |
| South East Asia | 9.80 | 119.39 | 0.7 | 0.8 | 0.8 | 0.2 | 0.8 | Severe |
| Caribbean | 9.80 | -75.82 | 0.7 | 0.9 | 0.7 | 0.7 | 0.9 | Severe |
| South East Asia | 9.83 | 98.11 | 0.5 | 0.9 | 0.6 | 0.5 | 0.8 | Severe |
| South East Asia | 9.87 | 122.37 | 0.6 | 0.7 | 0.7 | 0.3 | 0.8 | Severe |
| Eastern Pacific | 9.92 | -84.17 | 0.6 | 0.8 | 0.7 | 0.4 | 0.8 | High |
| Caribbean | 9.92 | -82.80 | 0.9 | 1.0 | 0.8 | 0.7 | 1.0 | Severe |
| South East Asia | 9.94 | 99.98 | 0.7 | 0.9 | 0.7 | 0.4 | 0.9 | Severe |
| Caribbean | 10.00 | -82.50 | 0.9 | 1.0 | 0.8 | 0.1 | 0.9 | Severe |
| South East Asia | 10.05 | 99.84 | 0.8 | 0.9 | 0.8 | 0.5 | 0.9 | Severe |
| South East Asia | 10.05 | 99.84 | 0.8 | 0.9 | 0.8 | 0.5 | 0.9 | Severe |
| South East Asia | 10.07 | 99.84 | 0.7 | 0.9 | 0.8 | 0.5 | 0.9 | Severe |
| Caribbean | 10.10 | -76.00 | 0.7 | 0.9 | 0.8 | 0.3 | 0.8 | Severe |
| South East Asia | 10.12 | 99.83 | 0.7 | 0.9 | 0.7 | 0.4 | 0.8 | Severe |
| Caribbean | 10.14 | -75.74 | 0.7 | 0.9 | 0.8 | 0.8 | 0.9 | Severe |
| Caribbean | 10.15 | -75.75 | 0.7 | 0.9 | 0.8 | 0.8 | 0.9 | Severe |
| South East Asia | 10.16 | 98.04 | 0.6 | 0.8 | 0.6 | 0.6 | 0.8 | Severe |
| South East Asia | 10.16 | 98.04 | 0.6 | 0.8 | 0.6 | 0.6 | 0.8 | Severe |
| South East Asia | 10.16 | 98.14 | 0.5 | 0.8 | 0.6 | 0.6 | 0.9 | Severe |
| Caribbean | 10.16 | -75.74 | 0.7 | 0.9 | 0.8 | 0.8 | 1.0 | Severe |
| Caribbean | 10.17 | -75.77 | 0.7 | 0.9 | 0.8 | 0.8 | 1.0 | Severe |
| Caribbean | 10.17 | -75.75 | 0.7 | 0.9 | 0.8 | 0.8 | 1.0 | Severe |
| Caribbean | 10.17 | -75.74 | 0.7 | 0.9 | 0.8 | 0.8 | 1.0 | Severe |
| South East Asia | 10.23 | 98.35 | 0.6 | 0.8 | 0.6 | 0.7 | 0.9 | Severe |
| Caribbean | 10.23 | -66.05 | 0.4 | 0.7 | 0.5 | 0.4 | 0.7 | High |
| South East Asia | 10.27 | 103.12 | 0.6 | 0.9 | 0.7 | 0.6 | 0.9 | Severe |
| South East Asia | 10.31 | 125.38 | 0.7 | 0.9 | 0.7 | 0.1 | 0.8 | High |
| Central Indian Ocean | 10.33 | 72.63 | 0.3 | 0.9 | 0.5 | 0.3 | 0.7 | High |
| South East Asia | 10.34 | 122.60 | 0.8 | 0.7 | 0.8 | 0.9 | 1.0 | Severe |
| South East Asia | 10.38 | 122.60 | 0.8 | 0.7 | 0.8 | 0.9 | 1.0 | Severe |
| South East Asia | 10.40 | 119.17 | 0.9 | 0.9 | 0.9 | 0.3 | 0.9 | Severe |
| Middle East | 10.43 | 44.97 | 0.9 | 0.4 | 0.8 | 0.9 | 1.0 | Severe |
| South East Asia | 10.47 | 98.22 | 0.7 | 0.8 | 0.7 | 0.7 | 0.9 | Severe |
| South East Asia | 10.47 | 119.17 | 0.7 | 0.9 | 0.8 | 0.3 | 0.8 | Severe |
| Eastern Pacific | 10.48 | -85.87 | 0.8 | 0.7 | 0.8 | 1.0 | 1.0 | Severe |
| South East Asia | 10.48 | 119.25 | 0.8 | 0.9 | 0.8 | 0.3 | 0.9 | Severe |
| South East Asia | 10.49 | 119.14 | 0.7 | 0.9 | 0.8 | 0.3 | 0.8 | Severe |
| South East Asia | 10.52 | 119.14 | 0.8 | 0.9 | 0.8 | 0.3 | 0.9 | Severe |
| Central Indian Ocean | 10.58 | 72.63 | 0.5 | 0.9 | 0.6 | 0.3 | 0.8 | High |
| South East Asia | 10.68 | 98.18 | 0.7 | 0.8 | 0.7 | 0.7 | 0.9 | Severe |
| South East Asia | 10.68 | 103.28 | 0.7 | 0.9 | 0.7 | 0.9 | 1.0 | Severe |
| Caribbean | 10.78 | -68.31 | 0.8 | 0.6 | 0.8 | 0.5 | 0.9 | Severe |
| South East Asia | 10.80 | 103.20 | 0.8 | 0.9 | 0.8 | 0.9 | 1.0 | Severe |
| South East Asia | 10.80 | 103.18 | 0.8 | 0.9 | 0.8 | 0.9 | 1.0 | Severe |
| Caribbean | 10.81 | -68.14 | 0.5 | 0.6 | 0.6 | 0.5 | 0.8 | High |
| South East Asia | 10.82 | 103.18 | 0.8 | 0.9 | 0.8 | 0.9 | 1.0 | Severe |
| South East Asia | 10.83 | 103.17 | 0.7 | 0.9 | 0.7 | 0.9 | 1.0 | Severe |
| South East Asia | 10.83 | 103.17 | 0.7 | 0.9 | 0.7 | 0.9 | 1.0 | Severe |
| Eastern Pacific | 10.85 | -85.95 | 0.7 | 0.7 | 0.8 | 0.9 | 1.0 | Severe |
| Caribbean | 10.85 | -68.21 | 0.7 | 0.6 | 0.7 | 0.5 | 0.8 | Severe |
| South East Asia | 10.87 | 97.92 | 0.7 | 0.8 | 0.7 | 0.7 | 0.9 | Severe |
| South East Asia | 10.88 | 120.80 | 0.7 | 0.8 | 0.7 | 0.4 | 0.9 | Severe |
| South East Asia | 10.88 | 103.08 | 0.6 | 0.9 | 0.7 | 0.8 | 1.0 | Severe |
| Caribbean | 10.89 | -68.22 | 0.6 | 0.5 | 0.6 | 0.5 | 0.8 | High |
| South East Asia | 10.97 | 98.14 | 0.7 | 0.8 | 0.7 | 0.7 | 0.9 | Severe |
| Central Indian Ocean | 10.98 | 73.00 | 0.4 | 0.9 | 0.6 | 0.4 | 0.7 | High |
| South East Asia | 11.00 | 114.00 | 0.6 | 0.9 | 0.7 | 0.1 | 0.7 | High |
| South East Asia | 11.05 | 103.07 | 0.8 | ? | 0.8 | 0.9 | 1.0 | Severe |
| South East Asia | 11.05 | 103.07 | 0.8 | ? | 0.8 | 0.9 | 1.0 | Severe |
| South East Asia | 11.05 | 103.08 | 0.8 | ? | 0.8 | 0.9 | 1.0 | Severe |
| South East Asia | 11.05 | 98.22 | 0.6 | 0.8 | 0.7 | 0.9 | 1.0 | Severe |
| Central Indian Ocean | 11.13 | 72.73 | 0.4 | 0.9 | 0.5 | 0.6 | 0.8 | High |
| Caribbean | 11.17 | -60.86 | 0.3 | 0.9 | 0.5 | 1.0 | 1.0 | Severe |
| Caribbean | 11.18 | -60.88 | 0.2 | 0.8 | 0.4 | 1.0 | 1.0 | Severe |
| South East Asia | 11.18 | 119.39 | 0.8 | 0.9 | 0.8 | 0.4 | 0.9 | Severe |
| South East Asia | 11.22 | 92.60 | 0.7 | 0.9 | 0.8 | 0.4 | 0.8 | Severe |
| Central Indian Ocean | 11.42 | 72.83 | 0.4 | 0.9 | 0.6 | 0.3 | 0.7 | High |
| South East Asia | 11.73 | 92.88 | 0.7 | 0.8 | 0.7 | 0.4 | 0.8 | Severe |
| Caribbean | 11.74 | -66.85 | 0.4 | 0.6 | 0.5 | 0.7 | 0.9 | Severe |
| Caribbean | 11.74 | -66.86 | 0.4 | 0.6 | 0.5 | 0.7 | 0.9 | Severe |
| Caribbean | 11.77 | -66.71 | 0.4 | 0.8 | 0.5 | 0.9 | 1.0 | Severe |
| Caribbean | 11.79 | -66.79 | 0.5 | 0.6 | 0.5 | 0.5 | 0.8 | High |
| Caribbean | 11.79 | -66.89 | 0.4 | 0.5 | 0.5 | 0.7 | 0.9 | Severe |
| Caribbean | 11.80 | -66.88 | 0.4 | 0.5 | 0.4 | 0.5 | 0.7 | High |
| South East Asia | 11.84 | 121.90 | 0.7 | 0.7 | 0.8 | 0.5 | 0.9 | Severe |
| Caribbean | 11.88 | -66.90 | 0.3 | 0.5 | 0.4 | 0.4 | 0.7 | High |
| Caribbean | 11.88 | -66.69 | 0.4 | 0.6 | 0.5 | 0.4 | 0.7 | High |
| Caribbean | 11.91 | -66.59 | 0.3 | 0.6 | 0.4 | 0.4 | 0.6 | Medial |
| Caribbean | 11.95 | -66.62 | 0.3 | 0.6 | 0.4 | 0.3 | 0.6 | Medial |
| Caribbean | 12.00 | -67.00 | 0.2 | 0.4 | 0.3 | 0.3 | 0.5 | Medial |
| Middle East | 12.00 | 43.50 | 0.9 | 0.4 | 0.8 | 0.9 | 1.0 | Severe |
| Middle East | 12.00 | 46.50 | 0.9 | 0.4 | 0.8 | 0.7 | 0.9 | Severe |
| Middle East | 12.00 | 54.02 | 0.6 | 0.4 | 0.6 | 0.9 | 0.9 | Severe |
| Middle East | 12.02 | 46.98 | 0.9 | 0.4 | 0.8 | 0.6 | 0.9 | Severe |
| Caribbean | 12.02 | -68.75 | 0.5 | 0.2 | 0.5 | 0.2 | 0.6 | Medial |
| Caribbean | 12.05 | -68.87 | 0.6 | 0.2 | 0.5 | 0.2 | 0.6 | Medial |
| Caribbean | 12.10 | -68.97 | 0.6 | 0.2 | 0.5 | 0.2 | 0.6 | Medial |
| Middle East | 12.13 | 53.18 | 0.5 | 0.6 | 0.5 | 0.9 | 0.9 | Severe |
| South East Asia | 12.15 | 122.21 | 0.7 | 0.7 | 0.7 | 0.3 | 0.8 | High |
| South East Asia | 12.16 | 109.31 | 0.9 | 0.6 | 0.8 | 0.7 | 0.9 | Severe |
| Caribbean | 12.17 | -68.33 | 0.4 | 0.2 | 0.4 | 0.3 | 0.6 | Medial |
| Caribbean | 12.17 | -68.33 | 0.4 | 0.2 | 0.4 | 0.3 | 0.6 | Medial |
| Caribbean | 12.18 | -83.03 | 0.7 | 1.0 | 0.7 | 0.4 | 0.8 | Severe |
| Caribbean | 12.19 | -83.07 | 0.7 | 1.0 | 0.8 | 0.4 | 0.9 | Severe |
| Caribbean | 12.19 | -83.03 | 0.7 | 1.0 | 0.7 | 0.4 | 0.8 | Severe |
| Caribbean | 12.20 | -83.07 | 0.7 | 1.0 | 0.8 | 0.4 | 0.9 | Severe |
| Caribbean | 12.20 | -83.06 | 0.7 | 1.0 | 0.7 | 0.4 | 0.8 | Severe |
| Caribbean | 12.24 | -68.24 | 0.6 | 0.2 | 0.6 | 0.3 | 0.7 | High |
| Caribbean | 12.26 | -68.43 | 0.2 | 0.2 | 0.3 | 0.2 | 0.5 | Medial |
| Caribbean | 12.30 | -82.97 | 0.7 | 1.0 | 0.7 | 0.4 | 0.8 | Severe |
| Caribbean | 12.31 | -82.99 | 0.7 | 1.0 | 0.7 | 0.4 | 0.8 | High |
| South East Asia | 12.31 | 122.20 | 0.7 | 0.7 | 0.7 | 0.3 | 0.8 | High |
| Caribbean | 12.32 | -82.98 | 0.7 | 1.0 | 0.7 | 0.4 | 0.8 | High |
| South East Asia | 12.37 | 122.20 | 0.7 | 0.7 | 0.8 | 0.3 | 0.8 | Severe |
| -81.65 | Isla de San Andr?s(Caribbean Sea) | 12.47 | 0.6 | 1.0 | 0.6 | 0.1 | 0.7 | High |
| Caribbean | 12.50 | -81.74 | 0.6 | 1.0 | 0.7 | 0.1 | 0.7 | High |
| South East Asia | 12.50 | 93.50 | 0.6 | 0.8 | 0.7 | 0.2 | 0.8 | High |
| Caribbean | 12.52 | -81.73 | 0.6 | 1.0 | 0.7 | 0.3 | 0.8 | High |
| Caribbean | 12.53 | -81.72 | 0.7 | 1.0 | 0.7 | 0.3 | 0.8 | High |
| Caribbean | 12.53 | -81.70 | 0.7 | 1.0 | 0.7 | 0.3 | 0.8 | High |
| Caribbean | 12.54 | -81.74 | 0.7 | 1.0 | 0.7 | 0.3 | 0.8 | High |
| South East Asia | 12.55 | 101.43 | 0.8 | 0.8 | 0.8 | 1.0 | 1.0 | Severe |
| Caribbean | 12.60 | -81.70 | 0.7 | 1.0 | 0.7 | 0.1 | 0.7 | High |
| Caribbean | 12.63 | -61.35 | 0.3 | 0.9 | 0.5 | 0.7 | 0.8 | Severe |
| Caribbean | 12.63 | -61.35 | 0.3 | 0.9 | 0.5 | 0.7 | 0.8 | Severe |
| Caribbean | 12.63 | -61.35 | 0.3 | 0.9 | 0.5 | 0.7 | 0.8 | Severe |
| Caribbean | 12.64 | -61.35 | 0.3 | 0.9 | 0.5 | 0.7 | 0.8 | Severe |
| Caribbean | 12.64 | -61.35 | 0.3 | 0.9 | 0.5 | 0.7 | 0.8 | Severe |
| South East Asia | 12.64 | 100.85 | 0.6 | 0.9 | 0.7 | 1.0 | 1.0 | Severe |
| Middle East | 12.78 | 54.35 | 0.4 | 0.4 | 0.5 | 0.8 | 0.9 | Severe |
| South East Asia | 12.87 | 100.67 | 0.6 | 0.9 | 0.7 | 1.0 | 1.0 | Severe |
| Middle East | 12.88 | 54.23 | 0.4 | 0.5 | 0.5 | 0.8 | 0.9 | Severe |
| South East Asia | 12.95 | 100.67 | 0.6 | 0.9 | 0.7 | 1.0 | 1.0 | Severe |
| Middle East | 13.00 | 46.50 | 0.9 | 0.4 | 0.9 | 0.7 | 1.0 | Severe |
| Caribbean | 13.04 | -59.52 | 0.3 | 0.9 | 0.5 | 0.4 | 0.7 | High |
| South East Asia | 13.14 | 100.71 | 0.6 | 0.8 | 0.7 | 1.0 | 1.0 | Severe |
| Micronesia | 13.43 | 144.65 | 0.6 | 0.8 | 0.7 | 0.0 | 0.7 | High |
| Micronesia | 13.48 | 144.69 | 0.7 | 0.7 | 0.7 | 0.0 | 0.7 | High |
| South East Asia | 13.50 | 109.33 | 0.8 | 0.4 | 0.7 | 0.7 | 0.9 | Severe |
| South East Asia | 13.51 | 109.33 | 0.8 | 0.4 | 0.7 | 0.7 | 0.9 | Severe |
| Caribbean | 13.51 | -61.40 | 0.3 | 0.9 | 0.4 | 0.5 | 0.7 | High |
| South East Asia | 13.53 | 120.93 | 0.7 | 0.8 | 0.7 | 0.6 | 0.9 | Severe |
| South East Asia | 13.54 | 120.76 | 0.7 | 0.8 | 0.7 | 0.5 | 0.9 | Severe |
| Micronesia | 13.60 | 144.83 | 0.8 | 0.7 | 0.8 | 0.0 | 0.8 | High |
| South East Asia | 13.69 | 120.87 | 0.9 | 0.8 | 0.8 | 0.5 | 0.9 | Severe |
| South East Asia | 13.72 | 120.85 | 0.9 | 0.8 | 0.8 | 0.5 | 0.9 | Severe |
| South East Asia | 13.72 | 120.87 | 0.9 | 0.8 | 0.8 | 0.5 | 0.9 | Severe |
| South East Asia | 13.79 | 120.68 | 0.9 | 0.8 | 0.9 | 0.6 | 0.9 | Severe |
| Middle East | 14.00 | 42.50 | 0.7 | 0.2 | 0.7 | 0.9 | 1.0 | Severe |
| South East Asia | 14.00 | 109.50 | 0.7 | 0.4 | 0.7 | 0.5 | 0.8 | Severe |
| Caribbean | 14.40 | -61.20 | 0.2 | 0.9 | 0.4 | 0.4 | 0.6 | Medial |
| South East Asia | 14.45 | 122.03 | 0.8 | 0.7 | 0.8 | 0.3 | 0.8 | Severe |
| Micronesia | 14.50 | 145.20 | 0.6 | 0.9 | 0.7 | 0.0 | 0.7 | High |
| Micronesia | 14.55 | 145.55 | 0.6 | 0.9 | 0.7 | 0.0 | 0.7 | High |
| South East Asia | 14.82 | 121.89 | 0.7 | 0.8 | 0.7 | 0.3 | 0.8 | Severe |
| South East Asia | 14.86 | 122.22 | 0.7 | 0.8 | 0.7 | 0.2 | 0.8 | High |
| Micronesia | 15.20 | 145.70 | 0.7 | 0.7 | 0.7 | 0.6 | 0.9 | Severe |
| Micronesia | 15.22 | 145.68 | 0.6 | 0.7 | 0.7 | 0.2 | 0.8 | High |
| Micronesia | 15.23 | 145.69 | 0.6 | 0.7 | 0.7 | 0.2 | 0.8 | High |
| Caribbean | 15.93 | -86.40 | 0.6 | 0.9 | 0.6 | 0.4 | 0.8 | High |
| Caribbean | 15.97 | -86.45 | 0.6 | 0.9 | 0.6 | 0.4 | 0.8 | High |
| Eastern Pacific | 16.00 | -95.00 | 0.7 | 0.6 | 0.7 | 1.0 | 1.0 | Severe |
| Micronesia | 16.02 | 146.07 | 0.4 | 0.9 | 0.6 | 0.0 | 0.6 | Medial |
| Caribbean | 16.18 | -88.57 | 0.7 | 0.9 | 0.7 | 0.9 | 1.0 | Severe |
| Caribbean | 16.20 | -88.35 | 0.7 | 0.9 | 0.7 | 0.9 | 1.0 | Severe |
| Caribbean | 16.21 | -88.18 | 0.6 | 0.9 | 0.7 | 0.9 | 1.0 | Severe |
| Caribbean | 16.25 | -61.83 | 0.2 | 0.6 | 0.3 | 0.2 | 0.4 | Medial |
| South East Asia | 16.31 | 119.77 | 0.7 | 0.6 | 0.7 | 0.1 | 0.7 | High |
| Caribbean | 16.33 | -88.17 | 0.7 | 0.9 | 0.7 | 1.0 | 1.0 | Severe |
| Caribbean | 16.33 | -86.47 | 0.6 | 0.9 | 0.6 | 0.2 | 0.7 | High |
| Caribbean | 16.37 | -61.65 | 0.4 | 0.5 | 0.5 | 0.3 | 0.6 | Medial |
| Caribbean | 16.43 | -88.20 | 0.7 | 0.9 | 0.7 | 1.0 | 1.0 | Severe |
| Caribbean | 16.45 | -88.02 | 0.6 | 0.9 | 0.7 | 0.7 | 0.9 | Severe |
| South East Asia | 16.45 | 119.92 | 0.8 | 0.6 | 0.8 | 0.4 | 0.9 | Severe |
| Caribbean | 16.47 | -88.28 | 0.7 | 0.9 | 0.7 | 1.0 | 1.0 | Severe |
| Middle East | 16.50 | 41.50 | 0.5 | 0.3 | 0.6 | 0.8 | 0.9 | Severe |
| Caribbean | 16.53 | -87.97 | 0.6 | 0.9 | 0.6 | 0.4 | 0.8 | High |
| Caribbean | 16.53 | -87.98 | 0.7 | 0.9 | 0.7 | 0.9 | 1.0 | Severe |
| Caribbean | 16.60 | -88.23 | 0.7 | 0.9 | 0.7 | 1.0 | 1.0 | Severe |
| Caribbean | 16.60 | -88.20 | 0.7 | 0.9 | 0.7 | 1.0 | 1.0 | Severe |
| Caribbean | 16.62 | -88.07 | 0.6 | 0.9 | 0.7 | 1.0 | 1.0 | Severe |
| Caribbean | 16.62 | -88.23 | 0.7 | 0.9 | 0.7 | 1.0 | 1.0 | Severe |
| Caribbean | 16.63 | -88.13 | 0.7 | 0.9 | 0.7 | 1.0 | 1.0 | Severe |
| Caribbean | 16.66 | -88.19 | 0.7 | 0.9 | 0.7 | 1.0 | 1.0 | Severe |
| Caribbean | 16.67 | -88.17 | 0.7 | 0.9 | 0.7 | 1.0 | 1.0 | Severe |
| Caribbean | 16.71 | -88.17 | 0.7 | 0.9 | 0.7 | 1.0 | 1.0 | Severe |
| Caribbean | 16.71 | -87.85 | 0.6 | 0.9 | 0.6 | 0.7 | 0.9 | Severe |
| Caribbean | 16.72 | -88.07 | 0.7 | 0.9 | 0.7 | 1.0 | 1.0 | Severe |
| Caribbean | 16.72 | -88.07 | 0.7 | 0.9 | 0.7 | 1.0 | 1.0 | Severe |
| Caribbean | 16.73 | -87.89 | 0.6 | 0.9 | 0.6 | 0.7 | 0.9 | Severe |
| Caribbean | 16.73 | -87.89 | 0.6 | 0.9 | 0.6 | 0.7 | 0.9 | Severe |
| Caribbean | 16.73 | -87.77 | 0.6 | 0.9 | 0.6 | 0.2 | 0.7 | High |
| Caribbean | 16.74 | -87.81 | 0.6 | 0.9 | 0.6 | 0.8 | 0.9 | Severe |
| Caribbean | 16.74 | -87.88 | 0.6 | 0.9 | 0.6 | 0.7 | 0.9 | Severe |
| Caribbean | 16.74 | -87.88 | 0.6 | 0.9 | 0.6 | 0.7 | 0.9 | Severe |
| Caribbean | 16.76 | -88.14 | 0.7 | 0.9 | 0.7 | 1.0 | 1.0 | Severe |
| Caribbean | 16.77 | -88.16 | 0.7 | 0.9 | 0.7 | 1.0 | 1.0 | Severe |
| Caribbean | 16.77 | -88.07 | 0.7 | 0.9 | 0.7 | 1.0 | 1.0 | Severe |
| Caribbean | 16.78 | -88.08 | 0.7 | 0.9 | 0.7 | 1.0 | 1.0 | Severe |
| Central Pacific | 16.78 | -169.51 | 0.1 | 0.8 | 0.3 | 0.0 | 0.3 | Low |
| Caribbean | 16.79 | -87.69 | 0.5 | 0.9 | 0.6 | 0.2 | 0.7 | High |
| Caribbean | 16.79 | -87.73 | 0.6 | 0.9 | 0.6 | 0.2 | 0.7 | High |
| Caribbean | 16.80 | -88.08 | 0.7 | 0.9 | 0.7 | 1.0 | 1.0 | Severe |
| Caribbean | 16.80 | -88.08 | 0.7 | 0.9 | 0.7 | 1.0 | 1.0 | Severe |
| Caribbean | 16.80 | -87.77 | 0.7 | 0.9 | 0.7 | 0.3 | 0.8 | High |
| Caribbean | 16.81 | -87.69 | 0.5 | 0.9 | 0.6 | 0.2 | 0.7 | High |
| Caribbean | 16.81 | -88.06 | 0.6 | 0.9 | 0.6 | 1.0 | 1.0 | Severe |
| Caribbean | 16.81 | -88.08 | 0.7 | 0.9 | 0.7 | 1.0 | 1.0 | Severe |
| Caribbean | 16.81 | -87.78 | 0.7 | 0.9 | 0.7 | 0.3 | 0.8 | High |
| Caribbean | 16.87 | -88.12 | 0.6 | 0.9 | 0.7 | 1.0 | 1.0 | Severe |
| Caribbean | 16.88 | -88.05 | 0.7 | 0.9 | 0.7 | 1.0 | 1.0 | Severe |
| Caribbean | 16.91 | -87.80 | 0.6 | 0.9 | 0.6 | 0.3 | 0.7 | High |
| Caribbean | 16.91 | -87.80 | 0.6 | 0.9 | 0.6 | 0.3 | 0.7 | High |
| Caribbean | 16.91 | -87.70 | 0.5 | 0.9 | 0.6 | 0.2 | 0.6 | Medial |
| Caribbean | 16.92 | -81.70 | 0.3 | 1.0 | 0.4 | 0.1 | 0.5 | Medial |
| Caribbean | 16.92 | -88.05 | 0.6 | 0.9 | 0.6 | 1.0 | 1.0 | Severe |
| Middle East | 16.99 | 55.18 | 0.5 | 0.4 | 0.5 | 1.0 | 1.0 | Severe |
| Caribbean | 17.00 | -88.01 | 0.5 | 0.9 | 0.6 | 1.0 | 1.0 | Severe |
| Caribbean | 17.09 | -88.00 | 0.5 | 0.9 | 0.6 | 1.0 | 1.0 | Severe |
| Caribbean | 17.14 | -87.60 | 0.4 | 0.9 | 0.5 | 0.2 | 0.6 | Medial |
| Caribbean | 17.15 | -87.57 | 0.5 | 0.9 | 0.5 | 0.2 | 0.6 | Medial |
| Caribbean | 17.16 | -87.91 | 0.5 | 0.9 | 0.5 | 0.7 | 0.9 | Severe |
| Caribbean | 17.17 | -87.59 | 0.5 | 0.9 | 0.5 | 0.5 | 0.8 | High |
| Caribbean | 17.18 | -88.08 | 0.7 | 0.9 | 0.7 | 1.0 | 1.0 | Severe |
| Caribbean | 17.19 | -87.59 | 0.4 | 0.9 | 0.5 | 0.7 | 0.8 | Severe |
| Caribbean | 17.20 | -87.53 | 0.4 | 0.9 | 0.5 | 0.2 | 0.6 | Medial |
| Caribbean | 17.22 | -87.61 | 0.4 | 0.9 | 0.5 | 0.7 | 0.8 | Severe |
| Caribbean | 17.22 | -87.53 | 0.4 | 0.9 | 0.5 | 0.2 | 0.6 | Medial |
| Caribbean | 17.22 | -87.61 | 0.4 | 0.9 | 0.5 | 0.7 | 0.8 | Severe |
| Caribbean | 17.22 | -87.61 | 0.4 | 0.9 | 0.5 | 0.7 | 0.8 | Severe |
| Caribbean | 17.23 | -87.61 | 0.4 | 0.9 | 0.5 | 0.7 | 0.8 | Severe |
| Caribbean | 17.23 | -87.60 | 0.4 | 0.9 | 0.5 | 0.7 | 0.8 | Severe |
| Caribbean | 17.25 | -87.53 | 0.4 | 0.9 | 0.5 | 0.2 | 0.6 | Medial |
| Caribbean | 17.27 | -87.82 | 0.6 | 0.9 | 0.6 | 0.4 | 0.8 | High |
| Caribbean | 17.28 | -88.04 | 0.6 | 0.9 | 0.6 | 1.0 | 1.0 | Severe |
| Caribbean | 17.30 | -87.50 | 0.4 | 0.9 | 0.5 | 0.2 | 0.6 | Medial |
| Caribbean | 17.32 | -88.00 | 0.5 | 0.9 | 0.6 | 1.0 | 1.0 | Severe |
| Caribbean | 17.33 | -88.03 | 0.6 | 0.9 | 0.6 | 1.0 | 1.0 | Severe |
| Caribbean | 17.34 | -87.54 | 0.5 | 0.9 | 0.5 | 0.3 | 0.7 | Medial |
| Caribbean | 17.34 | -87.95 | 0.5 | 0.9 | 0.6 | 1.0 | 1.0 | Severe |
| Caribbean | 17.35 | -88.04 | 0.6 | 0.9 | 0.6 | 1.0 | 1.0 | Severe |
| Caribbean | 17.35 | -88.03 | 0.6 | 0.9 | 0.6 | 1.0 | 1.0 | Severe |
| Caribbean | 17.36 | -87.95 | 0.5 | 0.9 | 0.6 | 1.0 | 1.0 | Severe |
| Caribbean | 17.39 | -87.94 | 0.6 | 0.9 | 0.6 | 0.9 | 1.0 | Severe |
| Caribbean | 17.39 | -87.94 | 0.6 | 0.9 | 0.6 | 0.9 | 1.0 | Severe |
| Caribbean | 17.41 | -87.55 | 0.4 | 0.9 | 0.5 | 0.4 | 0.7 | High |
| Caribbean | 17.46 | -63.22 | 0.2 | 0.5 | 0.3 | 0.4 | 0.6 | Medial |
| Caribbean | 17.49 | -63.23 | 0.2 | 0.5 | 0.3 | 0.4 | 0.6 | Medial |
| Caribbean | 17.49 | -63.52 | 0.2 | 0.5 | 0.4 | 0.3 | 0.6 | Medial |
| Caribbean | 17.49 | -88.05 | 0.6 | 0.9 | 0.6 | 1.0 | 1.0 | Severe |
| Caribbean | 17.50 | -87.85 | 0.5 | 0.9 | 0.5 | 0.5 | 0.8 | High |
| Caribbean | 17.55 | -87.82 | 0.5 | 0.9 | 0.5 | 0.3 | 0.7 | High |
| Caribbean | 17.57 | -87.74 | 0.4 | 0.9 | 0.5 | 0.5 | 0.8 | High |
| Caribbean | 17.59 | -87.74 | 0.4 | 0.9 | 0.5 | 0.5 | 0.7 | High |
| Caribbean | 17.59 | -87.75 | 0.4 | 0.9 | 0.5 | 0.5 | 0.7 | High |
| Caribbean | 17.62 | -63.26 | 0.2 | 0.5 | 0.4 | 0.2 | 0.5 | Medial |
| Caribbean | 17.62 | -63.23 | 0.3 | 0.5 | 0.4 | 0.2 | 0.5 | Medial |
| Caribbean | 17.62 | -63.26 | 0.3 | 0.5 | 0.4 | 0.2 | 0.5 | Medial |
| Caribbean | 17.63 | -63.26 | 0.3 | 0.5 | 0.4 | 0.2 | 0.5 | Medial |
| Caribbean | 17.63 | -63.26 | 0.3 | 0.5 | 0.4 | 0.2 | 0.5 | Medial |
| Caribbean | 17.63 | -63.22 | 0.3 | 0.5 | 0.4 | 0.1 | 0.4 | Medial |
| Caribbean | 17.63 | -63.26 | 0.3 | 0.5 | 0.4 | 0.2 | 0.5 | Medial |
| Caribbean | 17.64 | -63.25 | 0.3 | 0.5 | 0.4 | 0.2 | 0.5 | Medial |
| Caribbean | 17.65 | -63.23 | 0.3 | 0.5 | 0.4 | 0.1 | 0.4 | Medial |
| Caribbean | 17.67 | -88.02 | 0.5 | 0.9 | 0.5 | 1.0 | 1.0 | Severe |
| Caribbean | 17.67 | -88.01 | 0.5 | 0.9 | 0.5 | 1.0 | 1.0 | Severe |
| Caribbean | 17.70 | -64.90 | 0.3 | 0.5 | 0.4 | 0.6 | 0.8 | High |
| Caribbean | 17.76 | -64.66 | 0.6 | 0.5 | 0.6 | 0.2 | 0.7 | Medial |
| Caribbean | 17.77 | -64.69 | 0.6 | 0.5 | 0.6 | 0.2 | 0.7 | High |
| Caribbean | 17.79 | -64.63 | 0.4 | 0.5 | 0.4 | 0.7 | 0.8 | Severe |
| Caribbean | 17.79 | -64.63 | 0.4 | 0.5 | 0.4 | 0.7 | 0.8 | Severe |
| Caribbean | 17.79 | -64.60 | 0.4 | 0.5 | 0.4 | 0.7 | 0.8 | Severe |
| Caribbean | 17.86 | -88.00 | 0.7 | 0.9 | 0.7 | 1.0 | 1.0 | Severe |
| Caribbean | 17.93 | -79.81 | 0.2 | 0.7 | 0.4 | 0.0 | 0.5 | Medial |
| Caribbean | 17.94 | -87.94 | 0.7 | 0.9 | 0.7 | 1.0 | 1.0 | Severe |
| Caribbean | 17.99 | -67.31 | 0.2 | 0.5 | 0.3 | 0.4 | 0.6 | Medial |
| Caribbean | 17.99 | -63.06 | 0.3 | 0.4 | 0.4 | 0.3 | 0.6 | Medial |
| Eastern Pacific | 18.00 | -111.50 | 0.7 | 0.6 | 0.6 | 0.1 | 0.7 | High |
| Caribbean | 18.00 | -87.90 | 0.6 | 0.9 | 0.6 | 0.6 | 0.8 | Severe |
| Caribbean | 18.01 | -63.01 | 0.3 | 0.4 | 0.4 | 0.2 | 0.5 | Medial |
| Caribbean | 18.03 | -87.87 | 0.5 | 0.9 | 0.5 | 0.4 | 0.7 | High |
| Caribbean | 18.03 | -87.88 | 0.5 | 0.9 | 0.5 | 0.4 | 0.7 | High |
| Caribbean | 18.21 | -87.83 | 0.5 | 0.9 | 0.5 | 0.3 | 0.7 | Medial |
| Caribbean | 18.22 | -87.83 | 0.5 | 0.9 | 0.5 | 0.3 | 0.7 | Medial |
| Caribbean | 18.23 | -87.83 | 0.5 | 0.9 | 0.5 | 0.3 | 0.7 | Medial |
| Caribbean | 18.23 | -87.83 | 0.5 | 0.9 | 0.5 | 0.3 | 0.7 | Medial |
| Caribbean | 18.24 | -87.83 | 0.5 | 0.9 | 0.5 | 0.3 | 0.7 | Medial |
| Caribbean | 18.26 | -65.59 | 0.4 | 0.5 | 0.4 | 0.3 | 0.6 | Medial |
| Caribbean | 18.26 | -78.37 | 0.3 | 0.6 | 0.4 | 0.1 | 0.4 | Medial |
| Caribbean | 18.27 | -76.69 | 0.5 | 0.6 | 0.5 | 0.1 | 0.5 | Medial |
| Caribbean | 18.27 | -76.71 | 0.5 | 0.6 | 0.5 | 0.1 | 0.5 | Medial |
| Caribbean | 18.29 | -78.37 | 0.3 | 0.6 | 0.4 | 0.1 | 0.4 | Medial |
| Caribbean | 18.29 | -65.25 | 0.3 | 0.5 | 0.4 | 0.4 | 0.6 | Medial |
| Caribbean | 18.29 | -65.25 | 0.3 | 0.5 | 0.4 | 0.4 | 0.6 | Medial |
| Caribbean | 18.29 | -65.24 | 0.3 | 0.5 | 0.4 | 0.4 | 0.6 | Medial |
| Caribbean | 18.30 | -65.23 | 0.3 | 0.5 | 0.4 | 0.4 | 0.6 | Medial |
| Caribbean | 18.30 | -65.60 | 0.4 | 0.5 | 0.5 | 0.3 | 0.6 | Medial |
| Caribbean | 18.30 | -78.36 | 0.3 | 0.6 | 0.4 | 0.1 | 0.4 | Medial |
| Caribbean | 18.31 | -65.66 | 0.2 | 0.4 | 0.3 | 0.3 | 0.6 | Medial |
| Caribbean | 18.31 | -78.35 | 0.5 | 0.6 | 0.5 | 0.1 | 0.6 | Medial |
| Caribbean | 18.32 | -65.23 | 0.3 | 0.5 | 0.4 | 0.4 | 0.6 | Medial |
| Caribbean | 18.32 | -64.99 | 0.3 | 0.5 | 0.4 | 0.2 | 0.5 | Medial |
| Caribbean | 18.33 | -65.33 | 0.2 | 0.5 | 0.3 | 0.3 | 0.6 | Medial |
| Caribbean | 18.34 | -65.57 | 0.2 | 0.4 | 0.3 | 0.3 | 0.6 | Medial |
| Caribbean | 18.34 | -65.31 | 0.2 | 0.5 | 0.3 | 0.3 | 0.6 | Medial |
| Caribbean | 18.34 | -64.75 | 0.5 | 0.5 | 0.5 | 0.3 | 0.6 | Medial |
| Caribbean | 18.34 | -78.36 | 0.3 | 0.6 | 0.4 | 0.1 | 0.4 | Medial |
| Caribbean | 18.35 | -65.58 | 0.2 | 0.4 | 0.3 | 0.3 | 0.6 | Medial |
| Caribbean | 18.35 | -65.50 | 0.2 | 0.4 | 0.3 | 0.3 | 0.6 | Medial |
| Caribbean | 18.35 | -64.58 | 0.3 | 0.5 | 0.4 | 0.3 | 0.6 | Medial |
| Caribbean | 18.35 | -78.35 | 0.3 | 0.6 | 0.4 | 0.5 | 0.7 | High |
| Caribbean | 18.35 | -76.85 | 0.4 | 0.6 | 0.4 | 0.1 | 0.5 | Medial |
| Caribbean | 18.35 | -65.57 | 0.2 | 0.4 | 0.3 | 0.3 | 0.6 | Medial |
| Caribbean | 18.36 | -65.53 | 0.2 | 0.4 | 0.3 | 0.3 | 0.5 | Medial |
| Caribbean | 18.37 | -64.99 | 0.2 | 0.5 | 0.4 | 0.2 | 0.5 | Medial |
| Caribbean | 18.37 | -76.87 | 0.4 | 0.6 | 0.4 | 0.0 | 0.5 | Medial |
| Caribbean | 18.38 | -78.31 | 0.4 | 0.6 | 0.4 | 0.0 | 0.4 | Medial |
| Caribbean | 18.38 | -65.57 | 0.2 | 0.4 | 0.3 | 0.3 | 0.5 | Medial |
| Caribbean | 18.38 | -78.31 | 0.4 | 0.6 | 0.4 | 0.0 | 0.4 | Medial |
| Caribbean | 18.38 | -64.48 | 0.3 | 0.5 | 0.4 | 0.3 | 0.5 | Medial |
| Central Pacific | 18.38 | -154.63 | 0.1 | 0.8 | 0.3 | 0.0 | 0.3 | Low |
| Caribbean | 18.39 | -78.28 | 0.4 | 0.6 | 0.5 | 0.1 | 0.5 | Medial |
| Caribbean | 18.41 | -87.41 | 0.2 | 0.9 | 0.4 | 0.2 | 0.5 | Medial |
| Caribbean | 18.41 | -87.43 | 0.2 | 0.9 | 0.4 | 0.2 | 0.5 | Medial |
| Caribbean | 18.41 | -76.98 | 0.5 | 0.6 | 0.5 | 0.0 | 0.6 | Medial |
| Caribbean | 18.41 | -76.95 | 0.5 | 0.6 | 0.5 | 0.0 | 0.6 | Medial |
| Caribbean | 18.41 | -78.27 | 0.3 | 0.5 | 0.4 | 0.0 | 0.4 | Medial |
| Caribbean | 18.41 | -77.10 | 0.4 | 0.6 | 0.5 | 0.0 | 0.5 | Medial |
| Caribbean | 18.42 | -77.01 | 0.4 | 0.6 | 0.4 | 0.0 | 0.4 | Medial |
| Caribbean | 18.42 | -87.42 | 0.3 | 0.9 | 0.4 | 0.2 | 0.5 | Medial |
| Caribbean | 18.42 | -77.08 | 0.4 | 0.6 | 0.5 | 0.0 | 0.5 | Medial |
| Caribbean | 18.42 | -77.12 | 0.4 | 0.6 | 0.5 | 0.0 | 0.5 | Medial |
| Caribbean | 18.42 | -77.03 | 0.4 | 0.6 | 0.4 | 0.0 | 0.4 | Medial |
| Caribbean | 18.42 | -77.03 | 0.4 | 0.6 | 0.4 | 0.0 | 0.4 | Medial |
| Caribbean | 18.43 | -78.25 | 0.3 | 0.6 | 0.4 | 0.0 | 0.4 | Medial |
| Caribbean | 18.43 | -64.52 | 0.3 | 0.5 | 0.4 | 0.3 | 0.6 | Medial |
| Caribbean | 18.44 | -77.21 | 0.5 | 0.6 | 0.5 | 0.0 | 0.5 | Medial |
| Caribbean | 18.45 | -77.21 | 0.5 | 0.6 | 0.5 | 0.0 | 0.5 | Medial |
| Caribbean | 18.45 | -64.63 | 0.4 | 0.5 | 0.5 | 0.3 | 0.6 | Medial |
| Caribbean | 18.45 | -77.97 | 0.7 | 0.6 | 0.7 | 0.2 | 0.8 | High |
| Caribbean | 18.46 | -78.01 | 0.4 | 0.6 | 0.5 | 0.2 | 0.6 | Medial |
| Caribbean | 18.46 | -87.43 | 0.3 | 0.9 | 0.5 | 0.2 | 0.6 | Medial |
| Caribbean | 18.46 | -78.08 | 0.4 | 0.6 | 0.5 | 0.1 | 0.5 | Medial |
| Caribbean | 18.46 | -77.36 | 0.4 | 0.6 | 0.5 | 0.0 | 0.5 | Medial |
| Caribbean | 18.46 | -78.13 | 0.4 | 0.6 | 0.5 | 0.1 | 0.5 | Medial |
| Caribbean | 18.47 | -78.13 | 0.4 | 0.6 | 0.5 | 0.1 | 0.5 | Medial |
| Caribbean | 18.47 | -77.39 | 0.5 | 0.6 | 0.5 | 0.0 | 0.6 | Medial |
| Caribbean | 18.47 | -78.12 | 0.4 | 0.6 | 0.5 | 0.1 | 0.5 | Medial |
| Caribbean | 18.47 | -77.32 | 0.5 | 0.6 | 0.5 | 0.0 | 0.5 | Medial |
| Caribbean | 18.47 | -77.27 | 0.4 | 0.6 | 0.5 | 0.0 | 0.5 | Medial |
| Caribbean | 18.47 | -77.41 | 0.5 | 0.6 | 0.5 | 0.0 | 0.6 | Medial |
| Caribbean | 18.48 | -77.45 | 0.5 | 0.6 | 0.5 | 0.0 | 0.6 | Medial |
| Caribbean | 18.48 | -77.33 | 0.5 | 0.6 | 0.5 | 0.0 | 0.5 | Medial |
| Caribbean | 18.48 | -77.50 | 0.6 | 0.6 | 0.6 | 0.0 | 0.6 | Medial |
| Caribbean | 18.50 | -87.76 | 0.4 | 0.9 | 0.5 | 0.2 | 0.6 | Medial |
| Caribbean | 18.50 | -64.50 | 0.3 | 0.5 | 0.4 | 0.4 | 0.6 | Medial |
| Caribbean | 18.51 | -77.92 | 0.3 | 0.6 | 0.4 | 0.1 | 0.5 | Medial |
| Caribbean | 18.51 | -77.92 | 0.3 | 0.6 | 0.4 | 0.1 | 0.5 | Medial |
| Caribbean | 18.51 | -87.75 | 0.5 | 0.9 | 0.5 | 0.3 | 0.6 | Medial |
| Caribbean | 18.51 | -87.32 | 0.3 | 0.9 | 0.5 | 0.2 | 0.5 | Medial |
| Caribbean | 18.51 | -77.64 | 0.4 | 0.6 | 0.5 | 0.0 | 0.5 | Medial |
| Caribbean | 18.52 | -87.75 | 0.5 | 0.9 | 0.5 | 0.3 | 0.6 | Medial |
| Caribbean | 18.52 | -87.32 | 0.3 | 0.9 | 0.5 | 0.2 | 0.6 | Medial |
| Caribbean | 18.52 | -77.74 | 0.5 | 0.5 | 0.5 | 0.0 | 0.6 | Medial |
| Caribbean | 18.52 | -77.88 | 0.5 | 0.6 | 0.6 | 0.0 | 0.6 | Medial |
| Caribbean | 18.53 | -87.43 | 0.3 | 0.9 | 0.5 | 0.2 | 0.5 | Medial |
| Caribbean | 18.53 | -77.86 | 0.5 | 0.6 | 0.6 | 0.0 | 0.6 | Medial |
| Caribbean | 18.53 | -77.82 | 0.5 | 0.6 | 0.6 | 0.0 | 0.6 | Medial |
| Caribbean | 18.56 | -87.30 | 0.3 | 0.9 | 0.5 | 0.2 | 0.5 | Medial |
| Caribbean | 18.57 | -87.29 | 0.3 | 0.9 | 0.5 | 0.2 | 0.5 | Medial |
| Caribbean | 18.61 | -87.25 | 0.2 | 0.9 | 0.4 | 0.1 | 0.5 | Medial |
| Caribbean | 18.66 | -64.26 | 0.4 | 0.5 | 0.5 | 0.4 | 0.7 | Medial |
| Caribbean | 18.66 | -87.23 | 0.3 | 0.9 | 0.5 | 0.1 | 0.5 | Medial |
| Caribbean | 18.67 | -87.71 | 0.2 | 0.9 | 0.4 | 0.2 | 0.5 | Medial |
| Caribbean | 18.68 | -87.71 | 0.4 | 0.9 | 0.5 | 0.4 | 0.6 | Medial |
| Caribbean | 18.68 | -87.71 | 0.4 | 0.9 | 0.5 | 0.4 | 0.6 | Medial |
| Caribbean | 18.68 | -87.71 | 0.4 | 0.9 | 0.5 | 0.4 | 0.6 | Medial |
| Caribbean | 18.69 | -87.71 | 0.4 | 0.9 | 0.5 | 0.4 | 0.6 | Medial |
| Caribbean | 18.69 | -87.71 | 0.4 | 0.9 | 0.5 | 0.4 | 0.6 | Medial |
| Caribbean | 18.69 | -87.71 | 0.4 | 0.9 | 0.5 | 0.4 | 0.6 | Medial |
| Caribbean | 18.71 | -87.70 | 0.4 | 0.9 | 0.5 | 0.4 | 0.6 | Medial |
| Caribbean | 18.71 | -87.71 | 0.4 | 0.9 | 0.5 | 0.4 | 0.6 | Medial |
| Caribbean | 18.71 | -87.71 | 0.4 | 0.9 | 0.5 | 0.4 | 0.6 | Medial |
| Caribbean | 18.72 | -87.70 | 0.4 | 0.9 | 0.5 | 0.4 | 0.6 | Medial |
| Caribbean | 18.72 | -87.70 | 0.4 | 0.9 | 0.5 | 0.4 | 0.6 | Medial |
| Caribbean | 18.72 | -87.70 | 0.4 | 0.9 | 0.5 | 0.4 | 0.6 | Medial |
| Caribbean | 18.72 | -87.70 | 0.4 | 0.9 | 0.5 | 0.4 | 0.6 | Medial |
| Caribbean | 18.72 | -87.60 | 0.2 | 0.9 | 0.4 | 0.1 | 0.4 | Medial |
| Caribbean | 18.73 | -64.32 | 0.3 | 0.5 | 0.4 | 0.1 | 0.5 | Medial |
| Caribbean | 18.75 | -87.33 | 0.3 | 0.9 | 0.4 | 0.1 | 0.5 | Medial |
| Caribbean | 18.77 | -87.33 | 0.3 | 0.9 | 0.4 | 0.1 | 0.5 | Medial |
| Caribbean | 18.77 | -87.31 | 0.3 | 0.9 | 0.5 | 0.1 | 0.5 | Medial |
| Caribbean | 18.81 | -87.36 | 0.3 | 0.9 | 0.5 | 0.1 | 0.5 | Medial |
| Eastern Pacific | 18.84 | -111.08 | 0.8 | 0.4 | 0.6 | 0.1 | 0.7 | High |
| Caribbean | 18.89 | -87.63 | 0.3 | 0.9 | 0.4 | 0.1 | 0.5 | Medial |
| Caribbean | 18.90 | -87.62 | 0.4 | 0.9 | 0.5 | 0.6 | 0.8 | High |
| Caribbean | 18.91 | -87.62 | 0.4 | 0.9 | 0.5 | 0.6 | 0.8 | High |
| Caribbean | 19.00 | -87.57 | 0.3 | 0.9 | 0.4 | 0.7 | 0.8 | High |
| Caribbean | 19.14 | -87.54 | 0.2 | 0.9 | 0.3 | 0.7 | 0.8 | High |
| Caribbean | 19.15 | -87.54 | 0.2 | 0.9 | 0.3 | 0.7 | 0.8 | High |
| Caribbean | 19.15 | -87.53 | 0.2 | 0.9 | 0.3 | 0.7 | 0.8 | High |
| Caribbean | 19.17 | -69.53 | 0.7 | 0.4 | 0.6 | 1.0 | 1.0 | Severe |
| Caribbean | 19.23 | -96.02 | 0.5 | 0.3 | 0.4 | 0.7 | 0.8 | Severe |
| Caribbean | 19.29 | -81.15 | 0.3 | 0.8 | 0.5 | 0.0 | 0.5 | Medial |
| Caribbean | 19.29 | -81.20 | 0.3 | 0.8 | 0.4 | 0.0 | 0.5 | Medial |
| Caribbean | 19.32 | -81.23 | 0.3 | 0.8 | 0.4 | 0.0 | 0.5 | Medial |
| Caribbean | 19.32 | -81.07 | 0.3 | 0.8 | 0.4 | 0.0 | 0.5 | Medial |
| Caribbean | 19.33 | -81.08 | 0.3 | 0.8 | 0.4 | 0.0 | 0.5 | Medial |
| Caribbean | 19.34 | -81.07 | 0.3 | 0.8 | 0.4 | 0.0 | 0.5 | Medial |
| Caribbean | 19.35 | -81.20 | 0.3 | 0.8 | 0.5 | 0.0 | 0.5 | Medial |
| Caribbean | 19.38 | -81.29 | 0.4 | 0.8 | 0.5 | 0.0 | 0.5 | Medial |
| Caribbean | 19.40 | -81.36 | 0.4 | 0.8 | 0.5 | 0.0 | 0.5 | Medial |
| Caribbean | 19.50 | -87.30 | 0.2 | 0.9 | 0.4 | 0.1 | 0.4 | Medial |
| Middle East | 19.50 | 40.50 | 0.4 | 0.5 | 0.5 | 0.8 | 0.9 | Severe |
| Caribbean | 19.53 | -80.67 | 0.3 | 0.8 | 0.5 | 0.0 | 0.5 | Medial |
| Caribbean | 19.66 | -80.07 | 0.4 | 0.7 | 0.5 | 0.0 | 0.5 | Medial |
| Caribbean | 19.66 | -80.09 | 0.4 | 0.7 | 0.5 | 0.0 | 0.5 | Medial |
| Caribbean | 19.66 | -80.11 | 0.4 | 0.7 | 0.5 | 0.0 | 0.5 | Medial |
| Caribbean | 19.67 | -80.09 | 0.4 | 0.7 | 0.5 | 0.0 | 0.5 | Medial |
| Caribbean | 19.67 | -80.04 | 0.3 | 0.7 | 0.4 | 0.0 | 0.4 | Medial |
| Caribbean | 19.68 | -80.03 | 0.3 | 0.7 | 0.4 | 0.0 | 0.4 | Medial |
| Caribbean | 19.68 | -80.09 | 0.4 | 0.7 | 0.5 | 0.0 | 0.5 | Medial |
| Caribbean | 19.68 | -80.02 | 0.3 | 0.7 | 0.5 | 0.0 | 0.5 | Medial |
| Caribbean | 19.69 | -80.08 | 0.4 | 0.7 | 0.5 | 0.0 | 0.5 | Medial |
| Caribbean | 19.69 | -80.07 | 0.4 | 0.7 | 0.5 | 0.0 | 0.5 | Medial |
| Caribbean | 19.69 | -80.07 | 0.4 | 0.7 | 0.5 | 0.0 | 0.5 | Medial |
| Caribbean | 19.69 | -80.07 | 0.4 | 0.7 | 0.5 | 0.0 | 0.5 | Medial |
| Caribbean | 19.70 | -79.80 | 0.5 | 0.6 | 0.5 | 0.0 | 0.6 | Medial |
| Caribbean | 19.73 | -80.05 | 0.3 | 0.7 | 0.4 | 0.0 | 0.4 | Medial |
| Caribbean | 19.76 | -80.06 | 0.3 | 0.7 | 0.4 | 0.0 | 0.4 | Medial |
| Caribbean | 19.78 | -80.01 | 0.3 | 0.7 | 0.4 | 0.0 | 0.4 | Medial |
| Caribbean | 19.79 | -87.43 | 0.2 | 0.9 | 0.4 | 0.4 | 0.6 | Medial |
| Caribbean | 19.79 | -87.43 | 0.2 | 0.9 | 0.4 | 0.4 | 0.6 | Medial |
| Caribbean | 19.87 | -71.30 | 0.5 | 0.6 | 0.5 | 0.1 | 0.5 | Medial |
| Caribbean | 19.88 | -71.32 | 0.5 | 0.6 | 0.5 | 0.1 | 0.5 | Medial |
| Caribbean | 19.89 | -79.95 | 0.3 | 0.6 | 0.4 | 0.0 | 0.4 | Medial |
| Caribbean | 19.89 | -79.86 | 0.3 | 0.6 | 0.4 | 0.0 | 0.4 | Medial |
| Caribbean | 19.90 | -79.90 | 0.3 | 0.6 | 0.4 | 0.0 | 0.4 | Medial |
| Caribbean | 19.90 | -80.14 | 0.3 | 0.7 | 0.5 | 0.0 | 0.5 | Medial |
| Caribbean | 19.91 | -71.60 | 0.5 | 0.6 | 0.5 | 0.7 | 0.8 | Severe |
| Caribbean | 19.92 | -71.62 | 0.5 | 0.6 | 0.5 | 0.7 | 0.8 | Severe |
| Caribbean | 19.92 | -71.60 | 0.5 | 0.6 | 0.5 | 0.7 | 0.8 | Severe |
| Caribbean | 19.92 | -71.59 | 0.5 | 0.6 | 0.5 | 0.7 | 0.8 | Severe |
| Caribbean | 19.92 | -71.59 | 0.5 | 0.6 | 0.5 | 0.8 | 0.9 | Severe |
| Caribbean | 19.92 | -71.58 | 0.5 | 0.6 | 0.5 | 0.8 | 0.9 | Severe |
| Caribbean | 19.93 | -79.92 | 0.3 | 0.6 | 0.4 | 0.0 | 0.4 | Medial |
| Caribbean | 19.93 | -87.43 | 0.3 | 0.8 | 0.4 | 0.2 | 0.5 | Medial |
| Caribbean | 19.93 | -87.43 | 0.3 | 0.8 | 0.4 | 0.2 | 0.5 | Medial |
| Caribbean | 19.93 | -87.43 | 0.3 | 0.8 | 0.4 | 0.2 | 0.5 | Medial |
| Caribbean | 19.94 | -80.02 | 0.3 | 0.6 | 0.4 | 0.0 | 0.5 | Medial |
| Caribbean | 19.96 | -87.45 | 0.3 | 0.8 | 0.4 | 0.1 | 0.5 | Medial |
| Middle East | 20.26 | 58.42 | 0.4 | 0.5 | 0.4 | 1.0 | 1.0 | Severe |
| Caribbean | 20.27 | -87.22 | 0.2 | 0.8 | 0.3 | 0.0 | 0.3 | Low |
| Caribbean | 20.50 | -87.05 | 0.2 | 0.8 | 0.3 | 0.4 | 0.6 | Medial |
| Eastern Pacific | 20.53 | -105.32 | 0.4 | 0.1 | 0.4 | 1.0 | 1.0 | Severe |
| Caribbean | 20.53 | -87.01 | 0.2 | 0.8 | 0.3 | 0.4 | 0.6 | Medial |
| Micronesia | 20.55 | 144.89 | 0.5 | 0.6 | 0.5 | 0.0 | 0.5 | Medial |
| Caribbean | 20.57 | -78.20 | 0.4 | 0.6 | 0.5 | 0.9 | 0.9 | Severe |
| Caribbean | 20.58 | -78.12 | 0.4 | 0.6 | 0.5 | 0.9 | 0.9 | Severe |
| Caribbean | 20.59 | -78.11 | 0.4 | 0.6 | 0.5 | 0.9 | 0.9 | Severe |
| Caribbean | 20.59 | -78.47 | 0.5 | 0.6 | 0.5 | 0.8 | 0.9 | Severe |
| Caribbean | 20.60 | -78.18 | 0.4 | 0.6 | 0.5 | 0.9 | 0.9 | Severe |
| Caribbean | 20.61 | -78.28 | 0.4 | 0.6 | 0.5 | 0.9 | 0.9 | Severe |
| Caribbean | 20.61 | -78.25 | 0.4 | 0.6 | 0.4 | 0.9 | 0.9 | Severe |
| Caribbean | 20.64 | -78.70 | 0.4 | 0.6 | 0.5 | 0.7 | 0.8 | High |
| Caribbean | 20.64 | -78.68 | 0.4 | 0.6 | 0.5 | 0.7 | 0.8 | High |
| Caribbean | 20.64 | -78.66 | 0.4 | 0.6 | 0.5 | 0.7 | 0.8 | Severe |
| Caribbean | 20.81 | -78.85 | 0.6 | 0.6 | 0.6 | 0.7 | 0.9 | Severe |
| Caribbean | 20.82 | -78.97 | 0.4 | 0.6 | 0.4 | 0.1 | 0.5 | Medial |
| Caribbean | 20.82 | -78.97 | 0.4 | 0.6 | 0.4 | 0.1 | 0.5 | Medial |
| Caribbean | 20.90 | -79.10 | 0.4 | 0.6 | 0.5 | 0.1 | 0.5 | Medial |
| Caribbean | 20.91 | -79.12 | 0.4 | 0.6 | 0.5 | 0.1 | 0.5 | Medial |
| Caribbean | 20.97 | -79.20 | 0.4 | 0.6 | 0.5 | 0.2 | 0.6 | Medial |
| Caribbean | 20.97 | -79.21 | 0.4 | 0.6 | 0.5 | 0.2 | 0.6 | Medial |
| Caribbean | 20.97 | -79.20 | 0.4 | 0.6 | 0.5 | 0.2 | 0.6 | Medial |
| Caribbean | 20.97 | -79.22 | 0.4 | 0.6 | 0.5 | 0.2 | 0.6 | Medial |
| Caribbean | 20.99 | -70.78 | 0.2 | 0.6 | 0.3 | 0.1 | 0.4 | Medial |
| Central Pacific | 21.00 | -156.68 | 0.3 | 0.8 | 0.4 | 0.4 | 0.6 | Medial |
| Central Pacific | 21.02 | -156.67 | 0.2 | 0.8 | 0.2 | 0.1 | 0.3 | Low |
| Caribbean | 21.02 | -70.82 | 0.2 | 0.6 | 0.3 | 0.1 | 0.4 | Medial |
| Caribbean | 21.06 | -77.08 | 0.3 | 0.4 | 0.3 | 0.1 | 0.4 | Medial |
| Caribbean | 21.06 | -79.42 | 0.3 | 0.5 | 0.4 | 0.1 | 0.5 | Medial |
| Caribbean | 21.07 | -79.41 | 0.3 | 0.5 | 0.4 | 0.1 | 0.5 | Medial |
| Caribbean | 21.07 | -79.43 | 0.3 | 0.5 | 0.4 | 0.1 | 0.5 | Medial |
| Caribbean | 21.07 | -79.41 | 0.3 | 0.5 | 0.4 | 0.1 | 0.5 | Medial |
| Caribbean | 21.08 | -79.47 | 0.3 | 0.5 | 0.4 | 0.3 | 0.6 | Medial |
| Caribbean | 21.08 | -79.97 | 0.3 | 0.6 | 0.4 | 0.0 | 0.4 | Medial |
| Caribbean | 21.08 | -79.47 | 0.3 | 0.5 | 0.4 | 0.3 | 0.6 | Medial |
| Caribbean | 21.22 | -79.56 | 0.3 | 0.5 | 0.4 | 0.7 | 0.8 | Severe |
| Caribbean | 21.22 | -79.57 | 0.3 | 0.5 | 0.4 | 0.7 | 0.8 | Severe |
| Caribbean | 21.30 | -79.59 | 0.3 | 0.5 | 0.4 | 0.8 | 0.8 | Severe |
| Caribbean | 21.30 | -71.22 | 0.3 | 0.6 | 0.4 | 0.2 | 0.5 | Medial |
| Caribbean | 21.31 | -71.62 | 0.3 | 0.6 | 0.4 | 0.4 | 0.6 | Medial |
| Caribbean | 21.33 | -90.16 | 0.4 | 0.3 | 0.4 | 0.6 | 0.8 | High |
| Caribbean | 21.33 | -79.58 | 0.4 | 0.5 | 0.4 | 0.7 | 0.8 | Severe |
| Caribbean | 21.37 | -71.60 | 0.3 | 0.6 | 0.4 | 0.8 | 0.8 | Severe |
| Caribbean | 21.37 | -71.20 | 0.3 | 0.6 | 0.3 | 0.1 | 0.4 | Medial |
| Caribbean | 21.39 | -72.15 | 0.4 | 0.6 | 0.4 | 0.3 | 0.6 | Medial |
| Caribbean | 21.39 | -71.17 | 0.3 | 0.6 | 0.4 | 0.1 | 0.4 | Medial |
| Caribbean | 21.40 | -71.12 | 0.2 | 0.6 | 0.3 | 0.2 | 0.5 | Medial |
| Caribbean | 21.44 | -71.15 | 0.3 | 0.6 | 0.4 | 0.1 | 0.5 | Medial |
| Caribbean | 21.45 | -79.65 | 0.3 | 0.5 | 0.3 | 0.8 | 0.8 | Severe |
| Caribbean | 21.46 | -71.16 | 0.2 | 0.6 | 0.3 | 0.1 | 0.4 | Medial |
| Caribbean | 21.47 | -71.55 | 0.2 | 0.6 | 0.3 | 0.9 | 1.0 | Severe |
| Caribbean | 21.48 | -71.15 | 0.2 | 0.6 | 0.3 | 0.1 | 0.4 | Medial |
| Caribbean | 21.49 | -72.22 | 0.3 | 0.6 | 0.4 | 0.2 | 0.5 | Medial |
| Caribbean | 21.50 | -81.25 | 0.3 | 0.6 | 0.3 | 0.1 | 0.4 | Medial |
| Caribbean | 21.52 | -82.10 | 0.3 | 0.6 | 0.4 | 0.8 | 0.9 | Severe |
| Caribbean | 21.52 | -71.13 | 0.2 | 0.6 | 0.3 | 0.4 | 0.6 | Medial |
| Caribbean | 21.53 | -82.37 | 0.3 | 0.6 | 0.4 | 0.6 | 0.8 | High |
| Caribbean | 21.54 | -82.37 | 0.6 | 0.6 | 0.5 | 0.8 | 0.9 | Severe |
| Caribbean | 21.54 | -71.11 | 0.2 | 0.6 | 0.3 | 0.0 | 0.4 | Medial |
| Caribbean | 21.54 | -82.44 | 0.4 | 0.6 | 0.5 | 0.7 | 0.9 | Severe |
| Caribbean | 21.54 | -82.45 | 0.4 | 0.6 | 0.5 | 0.7 | 0.9 | Severe |
| Caribbean | 21.55 | -71.46 | 0.3 | 0.6 | 0.4 | 0.1 | 0.5 | Medial |
| Caribbean | 21.55 | -82.29 | 0.5 | 0.6 | 0.5 | 0.8 | 0.9 | Severe |
| Caribbean | 21.55 | -82.33 | 0.5 | 0.6 | 0.5 | 0.8 | 0.9 | Severe |
| Caribbean | 21.55 | -82.34 | 0.5 | 0.6 | 0.5 | 0.8 | 0.9 | Severe |
| Caribbean | 21.55 | -72.97 | 0.5 | 0.6 | 0.5 | 0.0 | 0.5 | Medial |
| Caribbean | 21.56 | -82.31 | 0.5 | 0.6 | 0.5 | 0.8 | 0.9 | Severe |
| Caribbean | 21.56 | -82.30 | 0.5 | 0.6 | 0.5 | 0.8 | 0.9 | Severe |
| Caribbean | 21.56 | -82.32 | 0.5 | 0.6 | 0.5 | 0.8 | 0.9 | Severe |
| Caribbean | 21.57 | -81.64 | 0.3 | 0.6 | 0.4 | 0.4 | 0.6 | Medial |
| Caribbean | 21.57 | -81.97 | 0.4 | 0.6 | 0.5 | 0.4 | 0.7 | High |
| Caribbean | 21.57 | -81.67 | 0.3 | 0.6 | 0.4 | 0.4 | 0.6 | Medial |
| Caribbean | 21.58 | -81.68 | 0.3 | 0.6 | 0.4 | 0.4 | 0.6 | Medial |
| Caribbean | 21.58 | -81.59 | 0.4 | 0.6 | 0.4 | 1.0 | 1.0 | Severe |
| Caribbean | 21.58 | -81.64 | 0.4 | 0.6 | 0.4 | 1.0 | 1.0 | Severe |
| Caribbean | 21.58 | -81.63 | 0.4 | 0.6 | 0.4 | 1.0 | 1.0 | Severe |
| Caribbean | 21.59 | -81.59 | 0.4 | 0.6 | 0.4 | 1.0 | 1.0 | Severe |
| Caribbean | 21.59 | -81.96 | 0.3 | 0.6 | 0.4 | 0.9 | 0.9 | Severe |
| Caribbean | 21.60 | -81.93 | 0.3 | 0.6 | 0.4 | 0.9 | 0.9 | Severe |
| Caribbean | 21.61 | -83.10 | 0.6 | 0.6 | 0.6 | 1.0 | 1.0 | Severe |
| Caribbean | 21.61 | -81.30 | 0.3 | 0.6 | 0.4 | 0.4 | 0.6 | Medial |
| Caribbean | 21.61 | -83.20 | 0.3 | 0.6 | 0.4 | 0.4 | 0.6 | Medial |
| Caribbean | 21.62 | -83.19 | 0.3 | 0.6 | 0.4 | 0.4 | 0.6 | Medial |
| Caribbean | 21.65 | -72.48 | 0.4 | 0.6 | 0.4 | 0.1 | 0.5 | Medial |
| Caribbean | 21.65 | -79.93 | 0.3 | 0.5 | 0.3 | 0.7 | 0.8 | Severe |
| Caribbean | 21.66 | -79.93 | 0.3 | 0.5 | 0.3 | 0.7 | 0.8 | Severe |
| Caribbean | 21.66 | -72.47 | 0.4 | 0.6 | 0.4 | 0.1 | 0.5 | Medial |
| Caribbean | 21.67 | -72.48 | 0.4 | 0.6 | 0.4 | 0.1 | 0.5 | Medial |
| Caribbean | 21.67 | -79.94 | 0.2 | 0.5 | 0.3 | 0.8 | 0.8 | Severe |
| Caribbean | 21.67 | -79.95 | 0.2 | 0.5 | 0.3 | 0.8 | 0.8 | Severe |
| Caribbean | 21.69 | -79.98 | 0.2 | 0.5 | 0.3 | 0.8 | 0.8 | Severe |
| Caribbean | 21.74 | -81.10 | 0.3 | 0.6 | 0.4 | 0.7 | 0.8 | High |
| Caribbean | 21.75 | -84.50 | 0.3 | 0.6 | 0.4 | 0.2 | 0.5 | Medial |
| Caribbean | 21.78 | -81.18 | 0.3 | 0.6 | 0.4 | 0.4 | 0.7 | Medial |
| Caribbean | 21.80 | -81.17 | 0.3 | 0.6 | 0.4 | 0.4 | 0.7 | Medial |
| Caribbean | 21.80 | -84.52 | 0.3 | 0.6 | 0.4 | 0.1 | 0.4 | Medial |
| Caribbean | 21.80 | -84.52 | 0.3 | 0.6 | 0.4 | 0.1 | 0.4 | Medial |
| Caribbean | 21.80 | -72.20 | 0.5 | 0.6 | 0.5 | 0.1 | 0.6 | Medial |
| Caribbean | 21.80 | -81.20 | 0.3 | 0.6 | 0.4 | 0.4 | 0.6 | Medial |
| Caribbean | 21.81 | -81.20 | 0.3 | 0.6 | 0.4 | 0.4 | 0.6 | Medial |
| Caribbean | 21.81 | -72.23 | 0.5 | 0.6 | 0.5 | 0.1 | 0.6 | Medial |
| Caribbean | 21.81 | -84.51 | 0.3 | 0.6 | 0.4 | 0.1 | 0.4 | Medial |
| Caribbean | 21.81 | -72.19 | 0.5 | 0.6 | 0.5 | 0.1 | 0.6 | Medial |
| Caribbean | 21.82 | -72.18 | 0.5 | 0.6 | 0.5 | 0.1 | 0.6 | Medial |
| Caribbean | 21.83 | -72.17 | 0.5 | 0.6 | 0.5 | 0.1 | 0.6 | Medial |
| Caribbean | 21.84 | -81.25 | 0.3 | 0.6 | 0.4 | 0.5 | 0.7 | High |
| Caribbean | 21.85 | -81.29 | 0.3 | 0.6 | 0.4 | 0.8 | 0.9 | Severe |
| Caribbean | 21.85 | -81.28 | 0.3 | 0.6 | 0.4 | 0.8 | 0.9 | Severe |
| Caribbean | 21.86 | -81.33 | 0.3 | 0.6 | 0.3 | 0.8 | 0.9 | Severe |
| Caribbean | 21.86 | -81.32 | 0.3 | 0.6 | 0.3 | 0.8 | 0.9 | Severe |
| Caribbean | 21.89 | -81.40 | 0.3 | 0.6 | 0.3 | 0.8 | 0.9 | Severe |
| Caribbean | 21.89 | -81.42 | 0.3 | 0.6 | 0.3 | 0.8 | 0.9 | Severe |
| Caribbean | 21.90 | -81.43 | 0.3 | 0.6 | 0.3 | 0.8 | 0.9 | Severe |
| Caribbean | 21.90 | -81.42 | 0.3 | 0.6 | 0.3 | 0.8 | 0.9 | Severe |
| Caribbean | 21.91 | -72.12 | 0.3 | 0.6 | 0.4 | 0.2 | 0.5 | Medial |
| Caribbean | 21.91 | -81.43 | 0.3 | 0.6 | 0.3 | 0.8 | 0.9 | Severe |
| Caribbean | 21.92 | -81.43 | 0.3 | 0.6 | 0.3 | 0.8 | 0.9 | Severe |
| Caribbean | 21.96 | -81.47 | 0.3 | 0.6 | 0.3 | 1.0 | 1.0 | Severe |
| Caribbean | 21.97 | -81.48 | 0.4 | 0.6 | 0.4 | 1.0 | 1.0 | Severe |
| Caribbean | 21.99 | -81.48 | 0.3 | 0.6 | 0.3 | 1.0 | 1.0 | Severe |
| Caribbean | 22.00 | -79.00 | 0.4 | 0.4 | 0.4 | 0.2 | 0.5 | Medial |
| Caribbean | 22.00 | -78.00 | 0.8 | 0.4 | 0.7 | 0.2 | 0.7 | High |
| Caribbean | 22.02 | -81.51 | 0.3 | 0.6 | 0.3 | 1.0 | 1.0 | Severe |
| Caribbean | 22.02 | -81.52 | 0.3 | 0.6 | 0.3 | 1.0 | 1.0 | Severe |
| Caribbean | 22.03 | -80.43 | 0.2 | 0.5 | 0.3 | 0.1 | 0.4 | Medial |
| Caribbean | 22.04 | -81.51 | 0.3 | 0.6 | 0.3 | 1.0 | 1.0 | Severe |
| Caribbean | 22.04 | -81.51 | 0.3 | 0.6 | 0.3 | 1.0 | 1.0 | Severe |
| Caribbean | 22.09 | -81.57 | 0.5 | 0.6 | 0.4 | 1.0 | 1.0 | Severe |
| Caribbean | 22.10 | -81.52 | 0.3 | 0.6 | 0.3 | 1.0 | 1.0 | Severe |
| Caribbean | 22.10 | -81.20 | 0.4 | 0.5 | 0.4 | 1.0 | 1.0 | Severe |
| Caribbean | 22.11 | -81.48 | 0.3 | 0.5 | 0.3 | 1.0 | 1.0 | Severe |
| Caribbean | 22.12 | -81.50 | 0.3 | 0.5 | 0.3 | 1.0 | 1.0 | Severe |
| Caribbean | 22.15 | -81.50 | 0.3 | 0.5 | 0.3 | 1.0 | 1.0 | Severe |
| Caribbean | 22.55 | -78.04 | 0.2 | 0.4 | 0.2 | 0.9 | 0.9 | Severe |
| Middle East | 22.55 | 39.03 | 0.7 | 0.3 | 0.6 | 0.5 | 0.8 | High |
| Middle East | 22.56 | 69.33 | 0.5 | 0.3 | 0.4 | 1.0 | 1.0 | Severe |
| Caribbean | 22.56 | -78.46 | 0.5 | 0.4 | 0.5 | 0.3 | 0.6 | Medial |
| Middle East | 22.60 | 38.82 | 0.5 | 0.3 | 0.5 | 0.4 | 0.7 | High |
| Caribbean | 22.63 | -78.65 | 0.3 | 0.4 | 0.3 | 0.3 | 0.5 | Medial |
| Caribbean | 22.88 | -83.55 | 0.4 | 0.5 | 0.4 | 0.4 | 0.6 | Medial |
| Caribbean | 22.88 | -83.57 | 0.4 | 0.5 | 0.4 | 0.4 | 0.6 | Medial |
| Caribbean | 22.91 | -79.70 | 0.4 | 0.4 | 0.4 | 0.6 | 0.8 | High |
| Caribbean | 22.92 | -83.48 | 0.3 | 0.5 | 0.3 | 0.5 | 0.6 | Medial |
| Caribbean | 22.94 | -79.75 | 0.3 | 0.4 | 0.3 | 0.7 | 0.8 | Severe |
| Caribbean | 22.94 | -79.75 | 0.3 | 0.4 | 0.3 | 0.7 | 0.8 | Severe |
| Caribbean | 23.10 | -80.12 | 0.4 | 0.3 | 0.3 | 0.4 | 0.6 | Medial |
| Caribbean | 23.13 | -73.75 | 0.2 | 0.4 | 0.3 | 0.0 | 0.3 | Low |
| Caribbean | 23.13 | -81.40 | 0.3 | 0.3 | 0.3 | 0.1 | 0.3 | Low |
| Caribbean | 23.15 | -82.41 | 0.4 | 0.5 | 0.4 | 0.1 | 0.5 | Medial |
| Caribbean | 23.21 | -80.47 | 0.3 | 0.3 | 0.3 | 0.6 | 0.7 | High |
| Caribbean | 23.22 | -80.47 | 0.3 | 0.3 | 0.3 | 0.6 | 0.7 | High |
| Caribbean | 23.24 | -80.59 | 0.4 | 0.3 | 0.4 | 0.1 | 0.5 | Medial |
| Caribbean | 23.24 | -80.59 | 0.4 | 0.3 | 0.4 | 0.1 | 0.5 | Medial |
| Caribbean | 23.27 | -77.40 | 0.4 | 0.3 | 0.4 | 0.8 | 0.9 | Severe |
| Caribbean | 23.29 | -80.90 | 0.4 | 0.3 | 0.3 | 0.1 | 0.4 | Medial |
| Caribbean | 23.29 | -80.90 | 0.4 | 0.3 | 0.3 | 0.1 | 0.4 | Medial |
| Caribbean | 23.29 | -80.96 | 0.5 | 0.3 | 0.4 | 0.9 | 1.0 | Severe |
| Caribbean | 23.29 | -80.96 | 0.2 | 0.3 | 0.2 | 0.1 | 0.3 | Low |
| Eastern Pacific | 23.40 | -109.40 | 0.6 | 0.0 | 0.4 | 0.6 | 0.8 | High |
| Middle East | 23.56 | 69.08 | 0.6 | 0.2 | 0.5 | 1.0 | 1.0 | Severe |
| Caribbean | 23.67 | -76.20 | 0.4 | 0.3 | 0.4 | 0.7 | 0.8 | Severe |
| Caribbean | 23.68 | -77.37 | 0.2 | 0.3 | 0.2 | 0.5 | 0.6 | Medial |
| Caribbean | 23.70 | -77.38 | 0.2 | 0.3 | 0.2 | 0.4 | 0.5 | Medial |
| Caribbean | 23.71 | -77.38 | 0.2 | 0.3 | 0.2 | 0.4 | 0.5 | Medial |
| Caribbean | 23.71 | -77.38 | 0.2 | 0.3 | 0.2 | 0.4 | 0.5 | Medial |
| Caribbean | 23.72 | -76.25 | 0.5 | 0.3 | 0.4 | 0.8 | 0.9 | Severe |
| Caribbean | 23.74 | -76.07 | 0.2 | 0.3 | 0.3 | 0.1 | 0.3 | Low |
| Caribbean | 23.74 | -76.01 | 0.2 | 0.3 | 0.2 | 0.1 | 0.3 | Low |
| Caribbean | 23.74 | -76.05 | 0.2 | 0.3 | 0.3 | 0.1 | 0.3 | Low |
| Caribbean | 23.76 | -76.12 | 0.4 | 0.3 | 0.4 | 0.1 | 0.4 | Medial |
| Caribbean | 23.78 | -77.42 | 0.2 | 0.3 | 0.2 | 0.3 | 0.5 | Medial |
| Caribbean | 23.78 | -77.42 | 0.2 | 0.3 | 0.2 | 0.3 | 0.5 | Medial |
| Caribbean | 23.79 | -76.14 | 0.3 | 0.3 | 0.3 | 0.1 | 0.3 | Low |
| Caribbean | 23.79 | -76.14 | 0.3 | 0.3 | 0.3 | 0.1 | 0.3 | Low |
| Caribbean | 23.79 | -77.43 | 0.2 | 0.3 | 0.2 | 0.3 | 0.5 | Medial |
| Caribbean | 23.80 | -76.14 | 0.3 | 0.3 | 0.3 | 0.1 | 0.3 | Low |
| Caribbean | 23.83 | -75.12 | 0.2 | 0.3 | 0.2 | 0.0 | 0.3 | Low |
| Middle East | 23.85 | 58.10 | 0.7 | 0.1 | 0.6 | 0.9 | 1.0 | Severe |
| Caribbean | 23.95 | -77.39 | 0.2 | 0.3 | 0.2 | 0.2 | 0.3 | Low |
| Caribbean | 23.95 | -74.55 | 0.3 | 0.4 | 0.3 | 0.0 | 0.3 | Low |
| Caribbean | 23.95 | -74.54 | 0.3 | 0.4 | 0.3 | 0.0 | 0.3 | Low |
| Caribbean | 24.00 | -74.56 | 0.3 | 0.4 | 0.3 | 0.0 | 0.3 | Low |
| Caribbean | 24.00 | -74.60 | 0.3 | 0.3 | 0.2 | 0.0 | 0.2 | Low |
| Caribbean | 24.03 | -74.54 | 0.3 | 0.4 | 0.3 | 0.0 | 0.3 | Low |
| Caribbean | 24.04 | -74.54 | 0.3 | 0.4 | 0.3 | 0.0 | 0.3 | Low |
| Caribbean | 24.14 | -74.46 | 0.3 | 0.3 | 0.3 | 0.0 | 0.3 | Low |
| Caribbean | 24.14 | -74.45 | 0.3 | 0.3 | 0.3 | 0.0 | 0.3 | Low |
| Caribbean | 24.15 | -74.47 | 0.3 | 0.3 | 0.3 | 0.0 | 0.3 | Low |
| Caribbean | 24.16 | -74.48 | 0.3 | 0.3 | 0.3 | 0.0 | 0.3 | Low |
| Caribbean | 24.16 | -74.46 | 0.3 | 0.3 | 0.3 | 0.0 | 0.3 | Low |
| Caribbean | 24.17 | -74.48 | 0.2 | 0.3 | 0.2 | 0.1 | 0.3 | Low |
| Middle East | 24.20 | 36.80 | 0.5 | 0.3 | 0.4 | 0.2 | 0.5 | Medial |
| Caribbean | 24.29 | -77.65 | 0.4 | 0.3 | 0.3 | 0.2 | 0.5 | Medial |
| South East Asia | 24.30 | 124.10 | 0.5 | 0.2 | 0.4 | 0.3 | 0.6 | Medial |
| Caribbean | 24.30 | -77.65 | 0.4 | 0.3 | 0.3 | 0.2 | 0.5 | Medial |
| Caribbean | 24.30 | -77.65 | 0.4 | 0.3 | 0.3 | 0.2 | 0.5 | Medial |
| Caribbean | 24.30 | -77.65 | 0.4 | 0.3 | 0.3 | 0.2 | 0.5 | Medial |
| Caribbean | 24.31 | -77.66 | 0.3 | 0.3 | 0.2 | 0.2 | 0.4 | Medial |
| Caribbean | 24.33 | -74.46 | 0.2 | 0.3 | 0.2 | 0.0 | 0.2 | Low |
| Caribbean | 24.34 | -77.67 | 0.3 | 0.3 | 0.2 | 0.2 | 0.4 | Medial |
| South East Asia | 24.35 | 124.11 | 0.6 | 0.2 | 0.4 | 0.3 | 0.6 | Medial |
| South East Asia | 24.40 | 124.00 | 0.5 | 0.2 | 0.4 | 0.4 | 0.6 | Medial |
| Caribbean | 24.44 | -77.70 | 0.5 | 0.3 | 0.4 | 0.2 | 0.5 | Medial |
| Caribbean | 24.44 | -77.70 | 0.5 | 0.3 | 0.4 | 0.2 | 0.5 | Medial |
| Caribbean | 24.45 | -81.88 | 0.3 | 0.3 | 0.3 | 0.6 | 0.7 | High |
| Caribbean | 24.48 | -81.89 | 0.3 | 0.3 | 0.3 | 0.6 | 0.7 | High |
| Caribbean | 24.49 | -81.66 | 0.4 | 0.2 | 0.3 | 0.7 | 0.8 | High |
| Middle East | 24.49 | 52.98 | 0.6 | 0.1 | 0.5 | 0.9 | 0.9 | Severe |
| Caribbean | 24.49 | -81.68 | 0.4 | 0.2 | 0.3 | 0.7 | 0.8 | High |
| Caribbean | 24.49 | -81.66 | 0.4 | 0.2 | 0.3 | 0.7 | 0.8 | High |
| Caribbean | 24.49 | -81.66 | 0.4 | 0.2 | 0.3 | 0.7 | 0.8 | High |
| Caribbean | 24.49 | -81.72 | 0.4 | 0.3 | 0.3 | 0.7 | 0.8 | High |
| Caribbean | 24.49 | -81.71 | 0.4 | 0.3 | 0.3 | 0.7 | 0.8 | High |
| Caribbean | 24.49 | -81.71 | 0.4 | 0.3 | 0.3 | 0.7 | 0.8 | High |
| Caribbean | 24.50 | -81.70 | 0.4 | 0.3 | 0.3 | 0.7 | 0.8 | High |
| Caribbean | 24.50 | -76.71 | 0.2 | 0.3 | 0.2 | 0.7 | 0.7 | High |
| Eastern Pacific | 24.50 | -110.40 | 0.4 | 0.1 | 0.3 | 0.9 | 0.9 | Severe |
| Caribbean | 24.50 | -81.05 | 0.3 | 0.2 | 0.3 | 0.2 | 0.4 | Medial |
| Middle East | 24.50 | 58.50 | 0.7 | 0.1 | 0.6 | 0.9 | 1.0 | Severe |
| Caribbean | 24.51 | -81.56 | 0.4 | 0.2 | 0.3 | 0.4 | 0.6 | Medial |
| Caribbean | 24.52 | -76.65 | 0.1 | 0.3 | 0.2 | 0.2 | 0.3 | Low |
| Caribbean | 24.53 | -77.69 | 0.1 | 0.3 | 0.2 | 0.2 | 0.3 | Low |
| Caribbean | 24.53 | -81.58 | 0.4 | 0.2 | 0.3 | 0.8 | 0.8 | Severe |
| Caribbean | 24.54 | -81.83 | 0.4 | 0.3 | 0.4 | 0.7 | 0.8 | Severe |
| Caribbean | 24.54 | -77.68 | 0.1 | 0.3 | 0.2 | 0.2 | 0.3 | Low |
| Caribbean | 24.54 | -77.68 | 0.1 | 0.3 | 0.2 | 0.2 | 0.3 | Low |
| Caribbean | 24.55 | -82.05 | 0.4 | 0.3 | 0.3 | 0.6 | 0.8 | High |
| Caribbean | 24.55 | -81.67 | 0.4 | 0.2 | 0.4 | 0.7 | 0.8 | High |
| Caribbean | 24.55 | -81.41 | 0.4 | 0.2 | 0.3 | 0.5 | 0.6 | Medial |
| Caribbean | 24.55 | -81.59 | 0.4 | 0.2 | 0.3 | 0.8 | 0.8 | Severe |
| Caribbean | 24.56 | -81.78 | 0.5 | 0.3 | 0.4 | 0.7 | 0.8 | Severe |
| Caribbean | 24.56 | -81.51 | 0.4 | 0.2 | 0.3 | 0.7 | 0.8 | High |
| Caribbean | 24.56 | -81.40 | 0.4 | 0.2 | 0.3 | 0.5 | 0.6 | Medial |
| Caribbean | 24.56 | -81.39 | 0.4 | 0.2 | 0.3 | 0.5 | 0.6 | Medial |
| Caribbean | 24.57 | -75.93 | 0.2 | 0.3 | 0.2 | 0.4 | 0.5 | Medial |
| Caribbean | 24.57 | -75.93 | 0.2 | 0.3 | 0.2 | 0.4 | 0.5 | Medial |
| Caribbean | 24.59 | -76.81 | 0.2 | 0.3 | 0.3 | 0.9 | 0.9 | Severe |
| Caribbean | 24.60 | -77.69 | 0.2 | 0.3 | 0.2 | 0.4 | 0.5 | Medial |
| Caribbean | 24.61 | -82.95 | 0.4 | 0.3 | 0.3 | 0.4 | 0.6 | Medial |
| Caribbean | 24.61 | -82.94 | 0.4 | 0.3 | 0.3 | 0.4 | 0.6 | Medial |
| Caribbean | 24.62 | -82.92 | 0.4 | 0.3 | 0.3 | 0.4 | 0.6 | Medial |
| Caribbean | 24.63 | -77.69 | 0.1 | 0.3 | 0.2 | 0.2 | 0.3 | Low |
| Caribbean | 24.63 | -77.69 | 0.1 | 0.3 | 0.2 | 0.2 | 0.3 | Low |
| Caribbean | 24.63 | -82.90 | 0.4 | 0.3 | 0.3 | 0.4 | 0.6 | Medial |
| Caribbean | 24.63 | -82.92 | 0.4 | 0.3 | 0.3 | 0.4 | 0.6 | Medial |
| Caribbean | 24.63 | -82.87 | 0.4 | 0.3 | 0.3 | 0.5 | 0.7 | High |
| Caribbean | 24.63 | -82.86 | 0.4 | 0.3 | 0.3 | 0.5 | 0.7 | High |
| Caribbean | 24.63 | -81.11 | 0.4 | 0.3 | 0.3 | 0.9 | 0.9 | Severe |
| Caribbean | 24.63 | -82.95 | 0.4 | 0.3 | 0.3 | 0.4 | 0.6 | Medial |
| Caribbean | 24.63 | -77.69 | 0.1 | 0.3 | 0.2 | 0.2 | 0.3 | Low |
| Caribbean | 24.63 | -81.11 | 0.4 | 0.3 | 0.3 | 0.9 | 0.9 | Severe |
| Caribbean | 24.63 | -82.92 | 0.4 | 0.3 | 0.3 | 0.4 | 0.6 | Medial |
| Caribbean | 24.63 | -82.91 | 0.4 | 0.3 | 0.3 | 0.4 | 0.6 | Medial |
| Caribbean | 24.64 | -82.92 | 0.4 | 0.3 | 0.3 | 0.4 | 0.6 | Medial |
| Caribbean | 24.64 | -82.92 | 0.4 | 0.3 | 0.3 | 0.4 | 0.6 | Medial |
| Caribbean | 24.64 | -82.92 | 0.4 | 0.3 | 0.3 | 0.4 | 0.6 | Medial |
| Caribbean | 24.64 | -82.93 | 0.4 | 0.3 | 0.3 | 0.4 | 0.6 | Medial |
| Caribbean | 24.65 | -82.87 | 0.4 | 0.3 | 0.3 | 0.5 | 0.7 | High |
| Caribbean | 24.65 | -76.81 | 0.3 | 0.3 | 0.2 | 0.9 | 0.9 | Severe |
| Caribbean | 24.67 | -82.87 | 0.4 | 0.3 | 0.3 | 0.6 | 0.7 | High |
| Caribbean | 24.67 | -82.89 | 0.4 | 0.3 | 0.3 | 0.5 | 0.7 | Medial |
| Caribbean | 24.67 | -82.81 | 0.4 | 0.3 | 0.3 | 0.6 | 0.7 | High |
| Caribbean | 24.68 | -80.98 | 0.3 | 0.2 | 0.3 | 0.9 | 0.9 | Severe |
| Caribbean | 24.68 | -80.99 | 0.3 | 0.2 | 0.3 | 0.9 | 0.9 | Severe |
| Caribbean | 24.68 | -80.95 | 0.3 | 0.2 | 0.3 | 0.9 | 0.9 | Severe |
| Caribbean | 24.69 | -82.91 | 0.4 | 0.3 | 0.3 | 0.5 | 0.7 | Medial |
| Caribbean | 24.69 | -80.95 | 0.3 | 0.2 | 0.3 | 0.9 | 0.9 | Severe |
| Caribbean | 24.70 | -82.77 | 0.4 | 0.2 | 0.3 | 0.7 | 0.8 | High |
| Caribbean | 24.70 | -82.82 | 0.4 | 0.2 | 0.3 | 0.6 | 0.8 | High |
| Caribbean | 24.72 | -80.86 | 0.4 | 0.2 | 0.3 | 1.0 | 1.0 | Severe |
| Caribbean | 24.73 | -82.80 | 0.4 | 0.2 | 0.3 | 0.6 | 0.8 | High |
| Caribbean | 24.75 | -77.78 | 0.3 | 0.3 | 0.3 | 0.2 | 0.4 | Medial |
| Caribbean | 24.75 | -77.78 | 0.2 | 0.3 | 0.2 | 0.2 | 0.3 | Low |
| Caribbean | 24.75 | -77.81 | 0.3 | 0.3 | 0.3 | 0.2 | 0.4 | Medial |
| Caribbean | 24.77 | -80.75 | 0.4 | 0.2 | 0.3 | 0.8 | 0.9 | Severe |
| Caribbean | 24.77 | -77.80 | 0.3 | 0.3 | 0.3 | 0.2 | 0.4 | Medial |
| Caribbean | 24.77 | -77.81 | 0.3 | 0.3 | 0.3 | 0.2 | 0.4 | Medial |
| Caribbean | 24.77 | -80.77 | 0.4 | 0.2 | 0.3 | 0.8 | 0.9 | Severe |
| Eastern Pacific | 24.78 | -110.48 | 0.4 | 0.1 | 0.3 | 0.9 | 0.9 | Severe |
| Caribbean | 24.79 | -80.89 | 0.6 | 0.2 | 0.4 | 1.0 | 1.0 | Severe |
| Caribbean | 24.81 | -80.82 | 0.5 | 0.2 | 0.4 | 1.0 | 1.0 | Severe |
| Caribbean | 24.84 | -80.64 | 0.4 | 0.2 | 0.3 | 0.7 | 0.8 | High |
| Caribbean | 24.84 | -80.73 | 0.5 | 0.2 | 0.3 | 1.0 | 1.0 | Severe |
| Caribbean | 24.84 | -77.86 | 0.2 | 0.3 | 0.2 | 0.2 | 0.4 | Medial |
| Caribbean | 24.84 | -77.86 | 0.2 | 0.3 | 0.2 | 0.2 | 0.4 | Medial |
| Caribbean | 24.84 | -77.86 | 0.2 | 0.3 | 0.2 | 0.2 | 0.4 | Medial |
| Caribbean | 24.84 | -77.86 | 0.2 | 0.3 | 0.2 | 0.2 | 0.4 | Medial |
| Caribbean | 24.85 | -77.86 | 0.2 | 0.3 | 0.2 | 0.2 | 0.4 | Medial |
| Eastern Pacific | 24.90 | -110.60 | 0.6 | 0.1 | 0.4 | 0.9 | 1.0 | Severe |
| Caribbean | 24.90 | -80.54 | 0.3 | 0.2 | 0.3 | 0.5 | 0.7 | Medial |
| Caribbean | 24.90 | -80.62 | 0.4 | 0.2 | 0.3 | 0.9 | 0.9 | Severe |
| Caribbean | 24.91 | -80.51 | 0.3 | 0.2 | 0.3 | 0.5 | 0.7 | Medial |
| Caribbean | 24.91 | -80.62 | 0.4 | 0.2 | 0.3 | 0.9 | 0.9 | Severe |
| Caribbean | 24.93 | -80.51 | 0.4 | 0.2 | 0.4 | 0.8 | 0.9 | Severe |
| Caribbean | 24.93 | -80.55 | 0.4 | 0.2 | 0.4 | 0.8 | 0.9 | Severe |
| Caribbean | 24.94 | -80.56 | 0.5 | 0.2 | 0.4 | 1.0 | 1.0 | Severe |
| Caribbean | 24.94 | -80.48 | 0.4 | 0.2 | 0.3 | 0.7 | 0.8 | High |
| Caribbean | 24.95 | -80.46 | 0.4 | 0.2 | 0.3 | 0.7 | 0.8 | High |
| Caribbean | 24.95 | -80.50 | 0.4 | 0.2 | 0.3 | 0.7 | 0.8 | High |
| Caribbean | 24.99 | -80.42 | 0.3 | 0.2 | 0.3 | 0.6 | 0.7 | High |
| Caribbean | 24.99 | -80.42 | 0.4 | 0.2 | 0.3 | 0.8 | 0.8 | Severe |
| Caribbean | 25.00 | -80.37 | 0.3 | 0.2 | 0.3 | 0.3 | 0.5 | Medial |
| Caribbean | 25.00 | -78.20 | 0.5 | 0.3 | 0.4 | 0.7 | 0.8 | High |
| Middle East | 25.00 | 53.50 | 0.7 | 0.1 | 0.5 | 0.9 | 1.0 | Severe |
| Caribbean | 25.01 | -80.37 | 0.3 | 0.2 | 0.3 | 0.7 | 0.8 | High |
| Caribbean | 25.01 | -80.41 | 0.4 | 0.2 | 0.3 | 0.8 | 0.9 | Severe |
| Caribbean | 25.02 | -80.37 | 0.3 | 0.2 | 0.3 | 0.7 | 0.8 | High |
| Caribbean | 25.03 | -80.35 | 0.3 | 0.2 | 0.3 | 0.7 | 0.8 | High |
| Caribbean | 25.04 | -80.36 | 0.3 | 0.2 | 0.3 | 0.7 | 0.8 | High |
| Caribbean | 25.04 | -80.38 | 0.4 | 0.2 | 0.3 | 0.8 | 0.9 | Severe |
| Caribbean | 25.04 | -80.36 | 0.3 | 0.2 | 0.3 | 0.7 | 0.8 | High |
| Caribbean | 25.06 | -77.94 | 0.3 | 0.3 | 0.3 | 0.3 | 0.5 | Medial |
| Caribbean | 25.07 | -80.42 | 0.4 | 0.2 | 0.3 | 0.8 | 0.9 | Severe |
| Middle East | 25.09 | 55.14 | 0.8 | 0.0 | 0.6 | 0.9 | 1.0 | Severe |
| Caribbean | 25.10 | -77.96 | 0.3 | 0.3 | 0.2 | 0.3 | 0.5 | Medial |
| Caribbean | 25.11 | -80.36 | 0.4 | 0.2 | 0.3 | 0.8 | 0.9 | Severe |
| Caribbean | 25.12 | -80.32 | 0.3 | 0.2 | 0.3 | 0.8 | 0.9 | Severe |
| Caribbean | 25.12 | -80.31 | 0.3 | 0.2 | 0.3 | 0.8 | 0.9 | Severe |
| Caribbean | 25.13 | -77.98 | 0.3 | 0.3 | 0.2 | 0.3 | 0.5 | Medial |
| Middle East | 25.13 | 54.91 | 0.5 | 0.0 | 0.4 | 0.9 | 0.9 | Severe |
| Caribbean | 25.14 | -77.99 | 0.2 | 0.3 | 0.2 | 0.3 | 0.5 | Medial |
| Caribbean | 25.15 | -80.28 | 0.3 | 0.2 | 0.3 | 0.8 | 0.9 | Severe |
| Caribbean | 25.15 | -80.28 | 0.3 | 0.2 | 0.3 | 0.8 | 0.9 | Severe |
| Caribbean | 25.16 | -78.00 | 0.2 | 0.3 | 0.2 | 0.3 | 0.5 | Medial |
| Caribbean | 25.16 | -80.24 | 0.3 | 0.2 | 0.2 | 0.4 | 0.6 | Medial |
| Caribbean | 25.16 | -80.28 | 0.3 | 0.2 | 0.3 | 0.8 | 0.9 | Severe |
| Caribbean | 25.17 | -80.26 | 0.3 | 0.2 | 0.3 | 0.8 | 0.9 | Severe |
| Caribbean | 25.19 | -77.02 | 0.2 | 0.3 | 0.2 | 0.4 | 0.5 | Medial |
| Caribbean | 25.20 | -80.22 | 0.3 | 0.2 | 0.2 | 0.7 | 0.8 | High |
| Caribbean | 25.20 | -80.17 | 0.3 | 0.2 | 0.3 | 0.4 | 0.6 | Medial |
| Middle East | 25.21 | 54.23 | 0.7 | 0.1 | 0.5 | 0.9 | 1.0 | Severe |
| Caribbean | 25.22 | -78.03 | 0.3 | 0.3 | 0.2 | 0.4 | 0.5 | Medial |
| Caribbean | 25.31 | -78.09 | 0.2 | 0.3 | 0.2 | 0.4 | 0.5 | Medial |
| Caribbean | 25.31 | -78.09 | 0.2 | 0.3 | 0.2 | 0.4 | 0.5 | Medial |
| Caribbean | 25.37 | -80.14 | 0.3 | 0.2 | 0.3 | 0.8 | 0.9 | Severe |
| Caribbean | 25.39 | -80.17 | 0.4 | 0.2 | 0.3 | 0.8 | 0.9 | Severe |
| Caribbean | 25.41 | -77.92 | 0.3 | 0.3 | 0.2 | 0.6 | 0.7 | High |
| Central Pacific | 25.42 | -170.57 | 0.4 | 0.4 | 0.2 | 0.4 | 0.5 | Medial |
| Caribbean | 25.46 | -80.17 | 0.4 | 0.2 | 0.3 | 1.0 | 1.0 | Severe |
| Caribbean | 25.47 | -80.11 | 0.3 | 0.2 | 0.3 | 0.6 | 0.7 | High |
| Caribbean | 25.48 | -80.15 | 0.3 | 0.2 | 0.3 | 1.0 | 1.0 | Severe |
| Caribbean | 25.50 | -80.20 | 0.5 | 0.2 | 0.3 | 1.0 | 1.0 | Severe |
| Caribbean | 25.50 | -76.80 | 0.3 | 0.3 | 0.2 | 0.2 | 0.4 | Medial |
| Caribbean | 25.57 | -80.22 | 0.5 | 0.2 | 0.4 | 1.0 | 1.0 | Severe |
| Caribbean | 25.59 | -80.10 | 0.3 | 0.2 | 0.3 | 0.6 | 0.7 | High |
| Middle East | 25.92 | 50.70 | 0.9 | 0.1 | 0.6 | 0.9 | 1.0 | Severe |
| Middle East | 25.99 | 50.88 | 0.8 | 0.0 | 0.6 | 0.9 | 1.0 | Severe |
| Middle East | 26.00 | 51.50 | 0.9 | 0.0 | 0.6 | 0.9 | 0.9 | Severe |
| Middle East | 26.00 | 56.00 | 0.6 | 0.1 | 0.5 | 1.0 | 1.0 | Severe |
| Caribbean | 26.01 | -80.10 | 0.3 | 0.2 | 0.2 | 0.6 | 0.7 | High |
| Caribbean | 26.01 | -80.10 | 0.3 | 0.2 | 0.2 | 0.6 | 0.7 | High |
| Middle East | 26.04 | 50.89 | 0.9 | 0.0 | 0.6 | 0.9 | 1.0 | Severe |
| Caribbean | 26.06 | -76.95 | 0.2 | 0.3 | 0.2 | 0.0 | 0.2 | Low |
| South East Asia | 26.07 | 127.07 | 0.6 | 0.1 | 0.4 | 0.1 | 0.4 | Medial |
| Middle East | 26.08 | 50.77 | 0.8 | 0.0 | 0.6 | 0.9 | 0.9 | Severe |
| Middle East | 26.08 | 50.76 | 0.8 | 0.0 | 0.6 | 0.9 | 0.9 | Severe |
| Middle East | 26.08 | 50.75 | 0.8 | 0.0 | 0.6 | 0.9 | 0.9 | Severe |
| Middle East | 26.13 | 50.68 | 0.9 | 0.0 | 0.6 | 0.9 | 0.9 | Severe |
| Middle East | 26.15 | 50.70 | 0.9 | 0.0 | 0.6 | 0.9 | 0.9 | Severe |
| Middle East | 26.24 | 55.14 | 0.5 | 0.1 | 0.4 | 1.0 | 1.0 | Severe |
| Middle East | 26.25 | 50.93 | 0.8 | 0.0 | 0.6 | 0.9 | 0.9 | Severe |
| Middle East | 26.25 | 56.33 | 0.8 | 0.1 | 0.6 | 1.0 | 1.0 | Severe |
| Middle East | 26.25 | 50.99 | 0.8 | 0.0 | 0.6 | 0.9 | 0.9 | Severe |
| Middle East | 26.28 | 50.94 | 0.8 | 0.0 | 0.6 | 0.9 | 0.9 | Severe |
| Middle East | 26.28 | 50.64 | 0.9 | 0.0 | 0.6 | 0.9 | 0.9 | Severe |
| Middle East | 26.29 | 50.80 | 0.8 | 0.0 | 0.6 | 0.9 | 0.9 | Severe |
| Middle East | 26.29 | 50.96 | 0.8 | 0.0 | 0.6 | 0.9 | 0.9 | Severe |
| Middle East | 26.30 | 53.40 | 0.6 | 0.1 | 0.4 | 0.9 | 1.0 | Severe |
| Middle East | 26.34 | 50.41 | 0.9 | 0.0 | 0.6 | 0.9 | 0.9 | Severe |
| Caribbean | 26.35 | -76.98 | 0.2 | 0.3 | 0.2 | 0.2 | 0.3 | Low |
| Caribbean | 26.36 | -76.98 | 0.2 | 0.3 | 0.2 | 0.2 | 0.3 | Low |
| South East Asia | 26.38 | 128.00 | 0.4 | 0.1 | 0.3 | 0.2 | 0.4 | Medial |
| Caribbean | 26.40 | -76.99 | 0.2 | 0.3 | 0.2 | 0.3 | 0.4 | Medial |
| South East Asia | 26.44 | 127.77 | 0.6 | 0.1 | 0.4 | 0.2 | 0.5 | Medial |
| South East Asia | 26.45 | 127.77 | 0.6 | 0.1 | 0.4 | 0.2 | 0.5 | Medial |
| Middle East | 26.50 | 55.50 | 0.5 | 0.1 | 0.4 | 1.0 | 1.0 | Severe |
| Caribbean | 26.53 | -76.90 | 0.2 | 0.3 | 0.1 | 0.1 | 0.2 | Low |
| Caribbean | 26.54 | -76.95 | 0.2 | 0.3 | 0.1 | 0.1 | 0.3 | Low |
| Middle East | 26.58 | 53.94 | 0.9 | 0.1 | 0.6 | 1.0 | 1.0 | Severe |
| Caribbean | 26.58 | -76.96 | 0.2 | 0.2 | 0.2 | 0.5 | 0.5 | Medial |
| Middle East | 26.60 | 50.80 | 0.8 | 0.0 | 0.5 | 0.9 | 0.9 | Severe |
| Caribbean | 26.60 | -76.98 | 0.2 | 0.2 | 0.2 | 0.2 | 0.3 | Low |
| South East Asia | 26.63 | 127.87 | 0.5 | 0.1 | 0.3 | 0.2 | 0.4 | Medial |
| South East Asia | 26.64 | 127.86 | 0.4 | 0.1 | 0.3 | 0.2 | 0.4 | Medial |
| South East Asia | 26.64 | 127.86 | 0.4 | 0.1 | 0.3 | 0.2 | 0.4 | Medial |
| Caribbean | 26.64 | -77.04 | 0.2 | 0.2 | 0.2 | 0.1 | 0.3 | Low |
| Middle East | 26.70 | 53.62 | 0.8 | 0.1 | 0.6 | 1.0 | 1.0 | Severe |
| Caribbean | 26.71 | -80.01 | 0.2 | 0.2 | 0.2 | 0.2 | 0.4 | Medial |
| Caribbean | 26.74 | -77.30 | 0.3 | 0.2 | 0.2 | 0.2 | 0.4 | Medial |
| Middle East | 26.89 | 50.97 | 0.7 | 0.0 | 0.5 | 0.9 | 0.9 | Severe |
| Middle East | 26.89 | 51.06 | 0.7 | 0.0 | 0.5 | 0.9 | 0.9 | Severe |
| Eastern Pacific | 26.90 | -111.90 | 0.4 | 0.1 | 0.3 | 1.0 | 1.0 | Severe |
| Middle East | 26.90 | 50.94 | 0.7 | 0.0 | 0.5 | 0.9 | 0.9 | Severe |
| Middle East | 26.95 | 51.04 | 0.7 | 0.0 | 0.5 | 0.9 | 0.9 | Severe |
| Micronesia | 27.00 | 135.80 | 0.6 | 0.0 | 0.4 | 0.1 | 0.4 | Medial |
| Micronesia | 27.00 | 142.17 | 0.5 | 0.1 | 0.3 | 0.1 | 0.4 | Medial |
| South East Asia | 27.05 | 128.42 | 0.4 | 0.1 | 0.3 | 0.2 | 0.4 | Medial |
| Caribbean | 27.26 | -78.38 | 0.3 | 0.2 | 0.2 | 0.5 | 0.6 | Medial |
| Caribbean | 27.26 | -78.40 | 0.3 | 0.2 | 0.3 | 0.6 | 0.7 | High |
| Middle East | 27.50 | 33.50 | 0.6 | 0.1 | 0.4 | 0.7 | 0.8 | Severe |
| Middle East | 27.50 | 49.70 | 0.7 | 0.1 | 0.5 | 0.8 | 0.9 | Severe |
| Middle East | 27.50 | 50.00 | 0.7 | 0.1 | 0.5 | 0.8 | 0.9 | Severe |
| Central Pacific | 27.83 | -175.83 | 0.5 | 0.2 | 0.3 | 0.1 | 0.4 | Medial |
| Caribbean | 27.87 | -93.82 | 0.3 | 0.1 | 0.2 | 0.4 | 0.5 | Medial |
| Caribbean | 27.87 | -93.82 | 0.3 | 0.1 | 0.2 | 0.4 | 0.5 | Medial |
| Caribbean | 27.88 | -93.82 | 0.3 | 0.1 | 0.2 | 0.4 | 0.5 | Medial |
| Central Pacific | 27.89 | -175.89 | 0.5 | 0.2 | 0.3 | 0.1 | 0.4 | Medial |
| Caribbean | 27.91 | -93.59 | 0.3 | 0.1 | 0.2 | 0.4 | 0.5 | Medial |
| Caribbean | 27.92 | -93.61 | 0.3 | 0.1 | 0.2 | 0.4 | 0.5 | Medial |
| Caribbean | 27.92 | -93.60 | 0.3 | 0.1 | 0.2 | 0.4 | 0.5 | Medial |
| Caribbean | 27.92 | -93.81 | 0.3 | 0.1 | 0.2 | 0.4 | 0.5 | Medial |
| Caribbean | 27.92 | -93.61 | 0.3 | 0.1 | 0.2 | 0.4 | 0.5 | Medial |
| South East Asia | 28.00 | 129.33 | 0.4 | 0.1 | 0.2 | 0.1 | 0.3 | Low |
| Central Pacific | 28.20 | -177.35 | 0.4 | 0.2 | 0.3 | 0.2 | 0.4 | Medial |
| Micronesia | 28.43 | 178.33 | 0.5 | 0.2 | 0.3 | 0.0 | 0.3 | Low |
| Middle East | 28.63 | 48.65 | 0.7 | 0.0 | 0.4 | 0.8 | 0.9 | Severe |
| Middle East | 29.00 | 48.50 | 0.7 | 0.0 | 0.4 | 0.9 | 0.9 | Severe |
| Middle East | 29.07 | 48.49 | 0.7 | 0.1 | 0.4 | 0.9 | 0.9 | Severe |
| Middle East | 29.50 | 49.00 | 0.7 | 0.1 | 0.5 | 0.8 | 0.9 | Severe |
| South East Asia | 30.00 | 130.00 | 0.5 | 0.1 | 0.3 | 0.4 | 0.6 | Medial |
| South East Asia | 30.27 | 130.42 | 0.6 | 0.1 | 0.4 | 0.3 | 0.5 | Medial |
| Middle East | 32.24 | 34.76 | 0.2 | 0.1 | 0.1 | 0.4 | 0.4 | Medial |
| Caribbean | 32.25 | -64.85 | 0.2 | 0.1 | 0.1 | 0.1 | 0.2 | Low |
| Caribbean | 32.32 | -64.79 | 0.3 | 0.1 | 0.2 | 0.1 | 0.3 | Low |
| Caribbean | 32.33 | -64.83 | 0.3 | 0.1 | 0.2 | 0.1 | 0.3 | Low |
| Caribbean | 32.43 | -64.85 | 0.3 | 0.1 | 0.2 | 0.1 | 0.3 | Low |
| Caribbean | 32.43 | -64.65 | 0.3 | 0.1 | 0.2 | 0.1 | 0.3 | Low |
| Caribbean | 32.47 | -64.80 | 0.3 | 0.1 | 0.2 | 0.1 | 0.3 | Low |
| Caribbean | 32.47 | -64.84 | 0.3 | 0.1 | 0.2 | 0.2 | 0.3 | Low |
| Caribbean | 32.50 | -64.76 | 0.2 | 0.1 | 0.1 | 0.1 | 0.2 | Low |
| South East Asia | 33.30 | 129.18 | 0.7 | 0.1 | 0.5 | 0.7 | 0.9 | Severe |
